# Global, regional, and national burden of chronic respiratory diseases and associated risk factors, 1990–2019: Results from the Global Burden of Disease Study 2019

**DOI:** 10.3389/fmed.2023.1066804

**Published:** 2023-03-28

**Authors:** Xiang Chen, Cheng-Wei Zhou, Yang-Yang Fu, Yao-Zhe Li, Lei Chen, Qing-Wei Zhang, Yan-Fan Chen

**Affiliations:** ^1^Key Laboratory of Heart and Lung, Division of Pulmonary Medicine, The First Affiliated Hospital of Wenzhou Medical University, Wenzhou, China; ^2^Pulmonary and Critical Care Medicine, The Third Affiliated Hospital of Wenzhou Medical University, Wenzhou, China; ^3^NHC Key Laboratory of Digestive Diseases (Renji Hospital, Shanghai Jiaotong University School of Medicine), Division of Gastroenterology and Hepatology, Shanghai Institute of Digestive Disease, Renji Hospital, School of Medicine, Shanghai Jiao Tong University, Shanghai, China

**Keywords:** chronic respiratory diseases, Global Burden of Diseases, Injuries, and Risk Factors Study, sociodemographic index, risk factor, epidemiology

## Abstract

**Background:**

The burden of chronic respiratory diseases has changed over the three decades. This study aims to describe the spatiotemporal trends of prevalence, mortality, and disability-adjusted life years (DALY) due to chronic respiratory diseases (CRDs) worldwide during 1990–2019 using data from the Global Burden of Disease Study 2019 (GBD 2019).

**Methods:**

The prevalence, mortality, and DALY attributable to CRDs and risk factors from 1990 to 2019 were estimated. We also assessed the driving factors and potentiality for improvement with decomposition and frontier analyses, respectively.

**Results:**

In 2019, 454.56 [95% uncertainty interval (UI): 417.35–499.14] million individuals worldwide had a CRD, showing a 39·8% increase compared with 1990. Deaths due to CRDs were 3.97 (95%UI: 3.58–4.30) million, and DALY in 2019 was 103.53 (95%UI: 94.79–112.27) million. Declines by average annual percent change (AAPC) were observed in age-standardized prevalence rates (ASPR) (0.64% decrease), age-standardized mortality rates (ASMR) (1.92%), and age-standardized DALY rates (ASDR) (1.72%) globally and in 5 socio-demographic index (SDI) regions. Decomposition analyses represented that the increase in overall CRDs DALY was driven by aging and population growth. However, chronic obstructive pulmonary disease (COPD) was the leading driver of increased DALY worldwide. Frontier analyses witnessed significant improvement opportunities at all levels of the development spectrum. Smoking remained a leading risk factor (RF) for mortality and DALY, although it showed a downward trend. Air pollution, a growing factor especially in relatively low SDI regions, deserves our attention.

**Conclusion:**

Our study clarified that CRDs remain the leading causes of prevalence, mortality, and DALY worldwide, with growth in absolute numbers but declines in several age-standardized estimators since 1990. The estimated contribution of risk factors to mortality and DALY demands the need for urgent measures to improve them.

**Systematic review registration:**

http://ghdx.healthdata.org/gbd-results-tool.

## Background

Chronic respiratory diseases (CRDs) are chronic, commonly occurring, and persistent diseases affecting the airways and the lung and are among the leading causes of morbidity and mortality globally. It was estimated that 4 million people died prematurely yearly worldwide (1) and that costs of about €380 billion were spent for the care of CRD patients among European Union states in 2019 (1), thus posing a major public health problem. CRDs mainly include chronic obstructive pulmonary disease (COPD), asthma, interstitial lung disease and pulmonary sarcoidosis (ILD and PS), and pneumoconiosis. Although COVID-19 is still spreading globally, the prevention and treatment of non-communicable diseases (NCDs) cannot be compromised (2). CRDs are nonnegligible contributors to the rising burden of NCDs worldwide (3). These types of CRDs are associated with socioeconomic development, demographic trends, and risk factors (RFs), including smoking, air pollution, and non-optimal temperature. Over the past 30 years, the aging population has increased rapidly (4). Moreover, economic shifts, sociodemographic development, and risk factors have changed suddenly over the decades (5). Consequently, the situation of CRDs has altered drastically.

Some preceding analyses of disease burden from CRDs were published. For instance, a systematic analysis for GBD 2017 performed by Li et al. (5) reported mortality, DALY, and associated ASR; however, this study lacks details about analyses on the prevalence and age-standardized prevalence rates (ASPR). Another systematic analysis for GBD 2017 by GBD CRDs Collaborators (1) reviewed the prevalence and ASPR, but its analysis was limited to the GBD region, and the association with the sociodemographic index (SDI) was not further analyzed. A relatively recent article by Gan et al. (6) on GBD 2019 discussed the incidence, mortality, and corresponding age-standardized rates (ASR), excluding national-level data. Meanwhile, previous studies (7–11) also analyzed the burden of COPD, asthma, ILD and PS, and pneumoconiosis separately but failed to show a comparative analysis.

To make progress based on previous research, the study aimed to (1) update and illustrate adequately the geographical and time trends of prevalence, mortality, and DALY due to CRDs, by subtype, age, sex, and SDI; (2) introduce innovatively new research methods (decomposition analysis and frontier analysis) for further discussion; and (3) analyze risk factor changes in different SDI and GBD regions in order to adjust control policies dynamically and achieve the health-related Sustainable Development Goals (SDGs) by 2030 (12).

## Methods

### Overview

GBD 2019 is the recent data that reports the trends and levels of the epidemiology of different global injuries and diseases. The methods used in GBD 2019 have been described in earlier reports (13, 14). In our study, we used a systematic review to illustrate the global, regional, and national burden of chronic respiratory diseases and associated risk factors, 1990–2019.

### Data source and definition

The data on prevalence, mortality, DALY, ASR (ASPR, ASMR, and age-standardized DALY rates [ASDR]) of CRDs in 204 countries and territories during 1990–2019 were obtained from the Global Burden of Disease Study 2019 (http://ghdx.healthdata.org/gbd-results-tool). Five categories of chronic respiratory diseases—COPD, pneumoconiosis, asthma, ILD and PS, and other CRDs—were identified based on the International Classification of Diseases and Injuries-10 (ICD-10) diagnostic codes. Covariates included the SDI and risk factors.

The SDI is a composite indicator of income per capita, average educational attainment, and total fertility rates. Index values range from 0 (lowest income, the worst average educational attainment, and highest fertility) to 1 (highest income, the best average educational attainment, and lowest fertility). Countries and territories were classified as regions with high, high-middle, middle, low-middle, or low SDI. The cutoff values used to determine regions for analysis were computed using country-level estimates of the SDI for the year 2019, excluding countries with populations of <1 million.

Risk factors were defined in the comparative risk assessment framework of the GBD 2019, which includes environmental and occupational risks (household air pollution from solid fuels, ambient particulate matter pollution, and ambient ozone pollution), behavioral risks (tobacco, including smoking and secondhand smoke), and metabolic risks (high body mass index). The risk factor hierarchy and accompanying definitions of exposure were clarified in the previous study.

### Statistical analyses

The standardized methods of the GBD 2019 have been extensively reported (13, 15). Prevalence, mortality, and DALY from CRDs, such as COPD, pneumoconiosis, asthma, ILD and PS, and others, were estimated for 204 countries and territories, matched by age and sex, from 1990 to 2019 using DisMod-MR 2.1, a Bayesian meta-regression tool. All estimates were derived from the mean of 1,000 draws, and 95% uncertainty intervals (UI) were determined using the 2.5th and 97.5th percentiles of the ordered draws. A Spearman's rank-order correlation was utilized to evaluate the strength and direction of the association between the SDI and age-standardized rates (ASMR and ASDR), the change in the SDI between 1990 and 2019 (the ratio of the 2019 index to the 1990 index), and the average annual percentage change (AAPC) of ASR during 1990–2019. To assess the magnitude and direction of trends in the ASR of CRDs over time, we adopted the JoinPoint software (version 4.9.0.0) and calculated the AAPC and the corresponding 95% confidence interval (CI) by joinpoint regression analysis. By comparing AAPC with 0, we ascertained whether the variation trend in different sections is statistically significant. A detailed description of the decomposition assessment and frontier analysis is described in Supplementary Method.

### Patient and public involvement

There was no patient or public involvement in the study. No patients were evaluated in the study.

## Results

### Disease burden at the global, regional, and national levels

#### Global level

This study identified 454.56 (95%UI: 417.35–499.14) million individuals with CRDs in 2019, with an ASPR of 5,789.16 (95%UI: 5,290.68–6,418.14) (Table 1, Supplementary Tables 1, 2). The rate of prevalence increased by 39.8% (Supplementary Table 2). Nevertheless, the APSR declined by an average of 0.64% (95%CI: −0.70 to −0.58) annually from 1990 to 2019, and this phenomenon was applicable in both sexes and all SDI regions (Table 1, Supplementary Tables 1, 2). It was found that CRDs were responsible for 3.97 (95%UI: 3.58–4.30) million deaths in 2019 globally, with an ASMR of 51.28 (95%UI: 45.90–55.51), whose AAPC decreased by 1.92 (95%CI: −2.00 to −1.84) (Table 1, Supplementary Tables 7, 8). In addition, DALY was 103.53 (95%UI: 94.79–112.27) million in 2019, with an ASR of 1,293.74 (95%UI: 1,182.99–1,403.57) that reduced by 1.72% (95%CI: −1.78 to −1.65) (Table 1, Supplementary Tables 14, 15).

**Table 1:** Prevalence, mortality, and DALYs of chronic pulmonary disease in 2019 for both sexes, all SDI quintiles and all regions, with AAPC from 1990 and 2019.

**GBD data**	**Prevalence (95% UI)**			**Mortality (95% UI)**			**DALYs (95% UI)**		
	**NO. (95% UI)**	**Age-standardized prevalence per 1,00,000 population (95% UI)**	**AAPC % (95% CI) 1990–2019**	**NO. (95% UI)**	**Age-standardized mortality per 1,00,000 population (95% UI)**	**AAPC %** **(95% CI)** **1990–2019**	**NO. (95% UI)**	**Age-standardized DALYs per 1,00,000 population (95% UI)**	**AAPC % (95% CI) 1990–2019**
Global	454,557,390 (417,354,403 to 499,144,380)	5,789.16 (5,290.68 to 6,418.14)	−0.64 (−0.7 to −0.58)	3,974,315 (3,581,757 to 4,303,823)	51.28 (45.9 to 55.51)	−1.92 (−2 to −1.84)	103,533,107 (94,792,077 to 112,266,452)	1,293.74 (1,182.99 to 1,403.57)	−1.72 (−1.78 to −1.65)
COPD	212,335,951 (200,422,146 to 225,097,834)	2,638.2 (2,492.17 to 2,796.14)	−0.34 (−0.4 to −0.28)	3,280,636 (2,902,855 to 3,572,367)	42.52 (37.63 to 46.31)	−1.93 (−2.03 to −1.84)	74,432,367 (68,204,127 to 80,193,347)	926.08 (848.76 to 997.67)	−1.82 (−1.9 to −1.74)
Asthma	262,405,182 (224,047,914 to 309,452,681)	3,415.53 (2,898.92 to 4,066.2)	−0.91 (−1.04 to −0.79)	461,069 (366,580 to 559,006)	5.8 (4.62 to 7.03)	−2.47 (−2.55 to −2.39)	21,550,977 (17,141,587 to 26,971,997)	273.63 (216.71 to 343.38)	−1.94 (−2.07 to −1.81)
ILD and PS	4,710,180 (4,020,397 to 5,401,700)	57.62 (49.42 to 65.67)	0.33 (0.25 to 0.4)	169,833 (118,756 to 204,802)	2.17 (1.5 to 2.62)	0.73 (0.61 to 0.84)	3,770,894 (2,864,234 to 4468319)	46.45 (35.12 to 54.98)	0.44 (0.3 to 0.58)
Pneumoconiosis	3,072,550 (2,596,999 to 3,596,518)	36.78 (31.1 to 43.08)	−0.38 (−0.71 to −0.04)	23,015 (20,348 to 26,159)	0.29 (0.26 to 0.33)	−2.6 (−2.74 to −2.46)	919,077 (761,478 to 1,116,127)	11.1 (9.23 to 13.45)	−2.03 (−2.19 to −1.87)
Other	/	/	/	39,761 (31,085 to 46,581)	0.5 (0.39 to 0.59)	−0.65 (−0.8 to −0.49)	2,859,792 (246,1295 to 3,217,791)	36.48 (31.41 to 41.1)	0.45 (0.4 to 0.51)
**Sex**									
Female	231,152,957 (212,559,495 to 252,660,333)	5,695.91 (5,209.31 to 6,282.15)	−0.67 (−0.73 to −0.62)	1,740,667 (1,456,918 to 1,961,313)	39.73 (33.24 to 44.75)	−1.92 (−2 to −1.84)	4,652,6316 (41,119,608 to 51,370,913)	1,093.03 (965.72 to 1,208.96)	−1.63 (−1.7 to −1.56)
Male	223,404,433 (203,679,747 to 248,407,883)	5,907.65 (5,382.04 to 6,581.16)	−0.63 (−0.71 to −0.55)	2,233,647 (2,029,782 to 2,452,532)	66.72 (60.55 to 73.06)	−1.97 (−2.06 to −1.89)	57,006,791 (51,895,441 to 62,641,862)	1,538.69 (1,399.47 to 1,690.3)	−1.82 (−1.9 to −1.74)
**SDI**									
High SDI	111,890,796 (103,862,564 to 120,447,230)	9,110.33 (8,211.2 to 10,240.43)	−0.49 (−0.59 to −0.4)	525,019 (453,715 to 559,024)	24.64 (21.49 to 26.07)	−0.73 (−0.8 to −0.66)	253,581 (220,120 to 281,835)	924.07 (797.2 to 1,067.03)	−0.63 (−0.68 to −0.57)
High–middle SDI	86,144,403 (78,876,826 to 94,478,706)	5,296.89 (4,736.23 to 6,016.11)	−0.83 (−0.94 to −0.72)	651,323 (580,032 to 772,576)	33.22 (29.45 to 39.31)	−3.2 (−3.42 to −2.97)	343,436 (298,812 to 389,741)	837 (750.92 to 948.78)	−2.67 (−2.88 to −2.46)
Middle SDI	123,815,506 (112,658,899 to 138,000,392)	5,264.17 (4,747.44 to 5,910.84)	−0.36 (−0.44 to −0.28)	1,206,232 (1,062,900 to 1,340,546)	59.82 (52.3 to 66.61)	−2.83 (−2.98 to −2.67)	790,676 (693,913 to 895,867)	1,316.93 (1,199.96 to 1,449.4)	−2.53 (−2.65 to −2.42)
Low–middle SDI	82,481,524 (75,628,261 to 91,033,633)	5,417.1 (5,005.46 to 5,903.14)	−0.28 (−0.35 to −0.21)	1,226,245 (1,031,957 to 1,380,038)	107.29 (90.1 to 120.82)	−1.36 (−1.57 to −1.15)	882,039 (739,773 to 1,015,616)	2,314.24 (2,029.67 to 2,562.08)	−1.44 (−1.68 to −1.2)
Low SDI	49,928,256 (43,435,735 to 58,192,038)	5,549.6 (5,101.56 to 6,120.66)	−0.27 (−0.31 to −0.23)	363,857 (310,934 to 403,593)	87.82 (74.17 to 97.67)	−0.95 (−1.16 to −0.73)	587,801 (462,330 to 721,639)	2,048.37 (1,802.01 to 2,247.14)	−1.05 (−1.21 to −0.89)
**Region**									
High–income Asia Pacific	13,524,396 (12,384,865 to 14,875,991)	5,181.21 (4,471.8 to 6,125.97)	−1.81 (−1.96 to −1.67)	72,143 (59,578 to 81,146)	12.31 (10.4 to 13.74)	−2.37 (−2.52 to −2.22)	296,69 (25,177 to 35,634)	468.37 (392.53 to 561.9)	−2.07 (−2.19 to −1.95)
High–income North America	52,638,477 (49,184,741 to 56,555,381)	12,449.57 (11,351.77 to 13,803.52)	0.38 (0.09 to 0.67)	243,409 (196,086 to 260,316)	36.24 (29.59 to 38.58)	0.47 (0.41 to 0.53)	124,425 (100,864 to 136,499)	1,374 (1,180.95 to 1,570.24)	0.16 (0.03 to 0.3)
Western Europe	49,616,742 (45,577,274 to 54,278,252)	8,449.61 (7,477.19 to 9,614.06)	−0.9 (−1.1 to −0.71)	244,861 (210,219 to 263,528)	22.45 (19.62 to 24.03)	−1.11 (−1.21 to −1)	88,544 (76,270 to 108,721)	769.63 (660.74 to 900.99)	−1.06 (−1.16 to −0.97)
Australasia	3,596,396 (3,240,398 to 4,002,542)	10,936.44 (9,497.47 to 12,696.54)	−1.02 (−1.29 to −0.75)	13,599 (11,348 to 15,144)	24.82 (20.92 to 27.5)	−1.33 (−1.44 to −1.21)	7,133 (6,172 to 8,387)	946.08 (793.37 to 1,128.11)	−1.31 (−1.38 to −1.24)
Andean Latin America	3,434,185 (2,812,614 to 4,232,116)	5,519.29 (4,546.85 to 6,761.35)	−0.59 (−0.72 to −0.46)	14,209 (11,012 to 17,013)	26.84 (20.79 to 32.1)	−0.74 (−0.97 to −0.51)	20,159 (15,929 to 25,834)	680.12 (559.51 to 811.82)	−1.45 (−1.6 to −1.29)
Tropical Latin America	15,686,977 (13,554,279 to 18,289,360)	7,279.67 (6,181.33 to 8,633.32)	−0.69 (−0.77 to −0.6)	77,698 (69,788 to 86,968)	34.1 (30.5 to 38.25)	−1.65 (−1.81 to −1.49)	67,690 (57,936 to 78,207)	909.91 (808.6 to 1,037.21)	−1.47 (−1.6 to −1.33)
Central Latin America	12,802,204 (11,151,611 to 14,835,688)	5,309.65 (4,606.64 to 6,148.31)	−0.57 (−0.63 to −0.5)	74,582 (62,845 to 85,574)	33.69 (28.27 to 38.66)	−0.8 (−0.97 to −0.64)	82,170 (68,279 to 95,942)	784.43 (686.77 to 890.61)	−0.97 (−1.1 to −0.83)
Southern Latin America	6,289,607 (5,692,879 to 7,053,892)	8,716.12 (7,771.03 to 1,0037.53)	0.06 (−0.02 to 0.13)	27,893 (24,150 to 30,844)	32.56 (28.24 to 35.95)	−0.04 (−0.36 to 0.28)	21,955 (19,087 to 25,705)	942.1 (817.88 to 1,086.82)	−0.2 (−0.3 to −0.1)
Caribbean	3,643,997 (3,236,341 to 4,150,963)	7,804.39 (6,858.66 to 8,982.39)	−0.23 (−0.27 to −0.18)	13,766 (11,412 to 16,209)	26.87 (22.23 to 31.67)	−0.05 (−0.21 to 0.11)	19,939 (13,568 to 31,132)	960.2 (797.86 to 1,118.46)	−0.35 (−0.42 to −0.28)
Central Europe	9,408,225 (8,600,853 to 10,356,267)	6,405.17 (5,664.26 to 7,297.92)	−0.72 (−0.81 to −0.64)	42,773 (3,7191 to 49,286)	19.12 (16.63 to 22.05)	−2.22 (−2.37 to −2.06)	20,066 (16,782 to 23,799)	678.01 (583.53 to 787.39)	−1.57 (−1.64 to −1.5)
Eastern Europe	10,862,111 (9,826,188 to 11,974,988)	4,453.83 (3,918.48 to 5,167.66)	−1.59 (−1.66 to −1.51)	56,496 (49,473 to 71,897)	16.23 (14.22 to 20.72)	−2.9 (−3.65 to −2.14)	35,005 (29,509 to 42,466)	537.15 (467.11 to 633.55)	−2.4 (−2.85 to −1.94)
Central Asia	3,671,866 (3,309,535 to 4,163,424)	4,412.99 (4,024.71 to 4,927.71)	−0.53 (-0.58 to −0.47)	22,237 (20,056 to 26,149)	39.41 (35.47 to 46.52)	−0.62 (−1.03 to −0.21)	33,594 (28,232 to 44,025)	937.22 (847.05 to 1,056.72)	−0.99 (−1.24 to −0.73)
North Africa and Middle East	31,776,197 (28,349,827 to 36,022,813)	5,891.24 (5,320.98 to 6,584.28)	0.09 (0.05 to 0.12)	128,513 (110,781 to 144,351)	36.1 (30.9 to 40.31)	−1.38 (−1.51 to −1.25)	171,495 (146,350 to 197,712)	1,033.42 (906.68 to 1,149.27)	−1.03 (−1.13 to −0.94)
South Asia	81,003,958 (74,252,202 to 88,513,792)	5,366.27 (4,957.78 to 5,815.77)	−0.24 (−0.42 to −0.07)	1,363,402 (1,125,162 to 1,563,756)	118.75 (97.56 to 135.84)	−1.49 (−1.72 to −1.26)	964,077 (799,166 to 1,127,515)	2,559.27 (2,206.93 to 2,879.03)	−1.4 (−1.55 to −1.24)
Southeast Asia	35,926,312 (32,695,055 to 39,797,804)	5,651.09 (5,157.94 to 6,262.32)	−0.13 (−0.21 to −0.06)	267,847 (233,250 to 296,152)	53.72 (46.49 to 59.45)	−1.83 (−1.92 to −1.74)	250,854 (210,194 to 313,559)	1,383.12 (1,235.32 to 1,511.65)	−1.57 (−1.64 to −1.51)
East Asia	72,603,642 (66,122,037 to 79,486,904)	4,352.33 (3,867.67 to 4,978.69)	−0.72 (−0.89 to −0.55)	1,126,946 (969,591 to 1,362,127)	67.48 (57.8 to 82.27)	−4.15 (−4.49 to −3.81)	350,308 (285,959 to 410,212)	1,270.89 (1,120.48 to 1,470.61)	−3.87 (−4.1 to −3.63)
Oceania	699,604 (640,666 to 766,013)	6,504.63 (6,095.2 to 6,939.03)	−0.52 (−0.55 to −0.48)	8,940 (7142 to 11103)	166.28 (133.34 to 202.63)	−0.68 (−0.71 to −0.66)	18,209 (10,600 to 34,090)	3,677.62 (3,020.87 to 4,477.19)	−0.72 (−0.77 to −0.67)
Western Sub-Saharan Africa	17,677,345 (15,043,894 to 21,372,019)	4,581.4 (4,126.53 to 5,168.81)	−0.57 (−0.7 to −0.43)	64,223 (54,089 to 74,359)	39.14 (33.48 to 44.62)	−1.15 (−1.21 to −1.09)	292,020 (221,970 to 364,887)	1,128.76 (986.27 to 1,272.71)	−1.02 (−1.1 to −0.95)
Eastern Sub-Saharan Africa	20,536,229 (17,359,042 to 24,732,550)	5,486.86 (4,919.39 to 6,242.77)	−0.57 (−0.62 to −0.52)	60,212 (51,794 to 69,611)	42.4 (36.86 to 48.27)	−1.39 (−1.43 to −1.35)	170,760 (126,011 to 223,638)	1,231.52 (1,082.84 to 1,392.41)	−1.37 (−1.41 to −1.32)
Central Sub-Saharan Africa	5,276,142 (4,515,014 to 6,211,222)	4,876.92 (4,434.13 to 5,402.1)	−0.26 (−0.31 to −0.22)	26,972 (18,531 to 40,753)	65.8 (44.51 to 104.69)	−0.99 (−1.09 to −0.89)	54,480 (40,954 to 69,999)	1,625 (1,229.32 to 2,246.8)	−1.01 (−1.11 to −0.91)
Southern Sub-Saharan Africa	3,882,778 (3,173,435 to 4,588,692)	5,523.82 (4,620.04 to 6,421.33)	−0.28 (−0.58 to 0.01)	23,593 (21,463 to 26,023)	49.21 (44.37 to 54.24)	−0.79 (−1.11 to −0.47)	37,240 (32,037 to 44,600)	1,387.64 (1,263.76 to 1,520.81)	−0.94 (−1.13 to −0.74)

The table was generated from data available at http://ghdx.healthdata.org/gbd-results-tool.

GBD, Global Burden of Disease; DALY, disability-adjusted life-year; UI, uncertainty interval; AAPC, average annual percent change; CI, confidence interval.

#### Regional level

In 2019, the ASPR of CRDs was found to be the highest in high-income countries like North America (12,449.57 [95%UI: 11,351.77–13,803.52]) and Australasia (10,936.44 [95%UI: 9,497.47–12,696.54]) (Table 1, Supplementary Tables 1, 2), whereas East Asia (4,352.33 [95%UI: 3,867.67–4,978.69]) and Central Asia (4,412.99 [95%UI: 4,024.71–4,927.71]) had the lowest ASPR (Table 1, Supplementary Tables 1, 2). The ASMR in 2019 was found to be the highest in Oceania (166.28 [95%UI: 133.34–202.63]) and South Asia (118.75 [95%UI: 97.56–135.84]), whereas high-income Asia Pacific (12.31 [95%UI: 10.4–13.74]), Eastern Europe (16.23 [95%UI: 14.22–20.72]), and Central Europe (19.12 [95%UI: 16.63 to 22.05]) had the lowest rates (Table 1, Supplementary Tables 7, 8). Meanwhile, the region with the highest ASDR in 2019 was Oceania (161.79[95%UI: 90.64–280.06]), and the region with the lowest rate was high-income Asia Pacific (14.99 [95%UI: 12.56 to 17.57]) (Table 1, Supplementary Tables 14, 15).

During the three decades, the AAPC in ASPR of CRDs was different across GBD regions, with high-income Asia Pacific (−1.81%[95%CI: −1.96 to −1.67]), Eastern Europe (−1.59% [95%CI: −1.66 to −1.51]), and Australasia (−1.02% [95%CI: −1.29 to −0.75]) having significant decreasing trends (Table 1, Supplementary Tables 1, 2). In contrast, North Africa and the Middle East (0.09% [95%CI: 0.05–0.12]) and Southern Latin America (0.06% [95%CI: −0.02 to 0.13]) had weak increasing trends (Table 1, Supplementary Tables 1, 2). Regions with the largest decreasing AAPC trends in ASMR over the past decades included East Asia (−4.15% [95%CI: (−4.49 to −3.81]) and Eastern Europe (−2.90% [95%CI: −3.65 to −2.14]). In contrast, a substantially increasing trend was witnessed in high-income North America (0.47% [95%CI: 0.41–0.53]) (Table 1, Supplementary Tables 7, 8). The AAPC of ASDR of high-income North America (0.16% [95%CI: 0.03 to 0.3]) and East Asia (−3.87%[95%CI: −4.1 to −3.63]) during the period was at the poles of increasing and decreasing trends (Table 1, Supplementary Tables 14, 15).

#### National level

**Figure 1:**
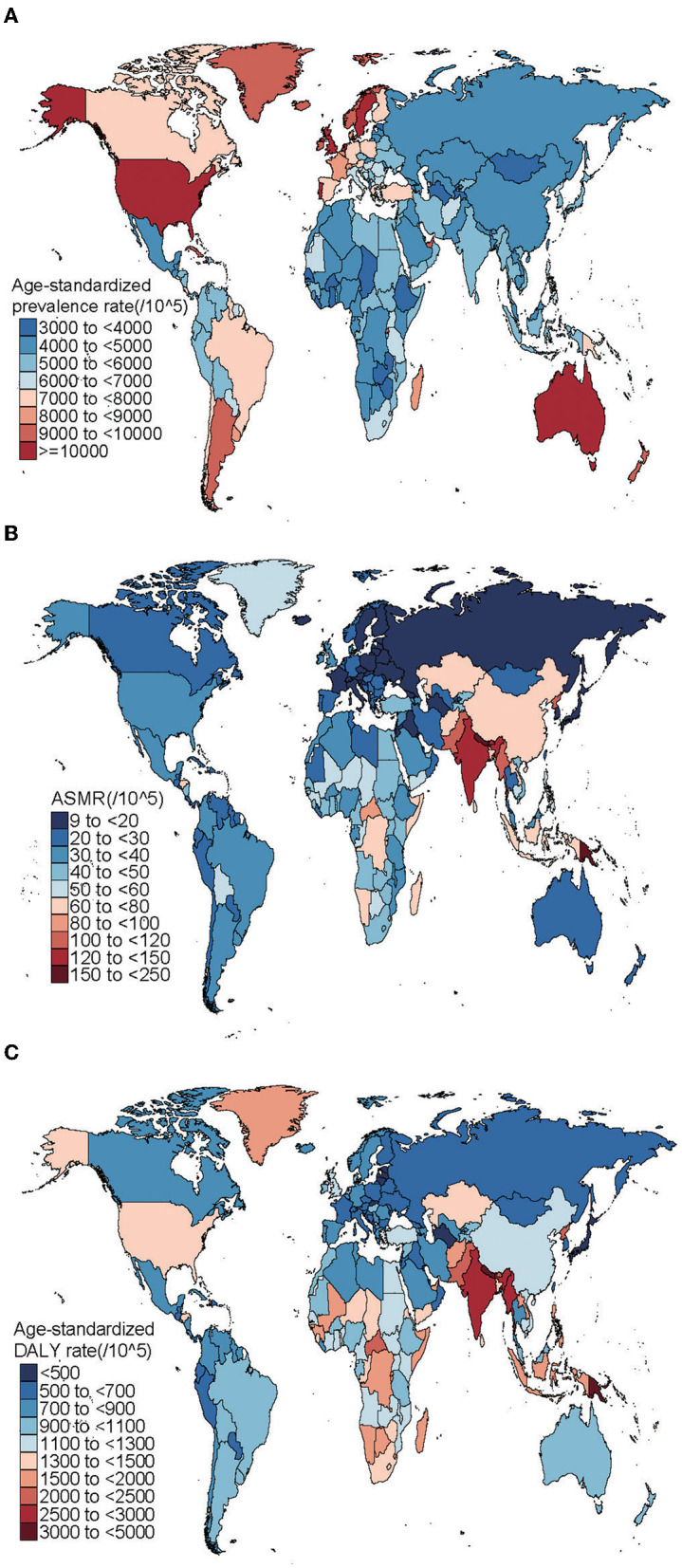
Global ASPR **(A)**, ASMR **(B)**, and ASDR **(C)** of CRDs in 204 countries and territories in 2019. ASPR, age-standardized prevalence rate; ASMR, age-standardized mortality rate; ASDR, age-standardized DALY rate; DALY, disability-adjusted life year; CRDs, chronic respiratory diseases.

In 2019, the ASPR of CRDs varied notably between countries, such that the USA (13,030.37 [95%UI: 11,908.32–14,412.04]), the UK (12,151.98 [95%UI: 10,750.52–13,846.11]), and Australia (11,253.26 [95%UI: 9,736.76–13,143.42]) had the three highest rates of all countries (Figure 1A, Supplementary Table 2). By comparison, countries with the lowest rates were Turkmenistan (3,297.51 [95%UI: 2,839.46–3,828.59]), Mongolia (3,331.34 [95%UI: 2,978.47 to 3,764.9]), and Estonia (3,429.92 [95%UI: 3,017.72–3,934.08]) (Figure 1A, Supplementary Table 2). Meanwhile, the results were observed for ASMR in 2019, with Nepal (231.2 [95%UI: 175.79–270.35]), Papua New Guinea (209.49 [95%UI: 162.01–259.45]), and Solomon Islands (145.87 [95%UI: 118.53–169.97]) producing the highest rates and Montenegro (9.32 [95%UI: 7.48–10.91]), Latvia (9.92 [95%UI: 7.94–13.53]), Estonia (10.27 [95%UI:8.01–13.09]), and Singapore (10.58 [95%UI: 8.82–14.07]) produced the lowest rates (Figure 1B, Supplementary Table 8). Papua New Guinea (4,452.56 [95%UI: 3,566.00–5,534.57]) and Nepal (4,339.27 [95%UI: 3,410.62–5,078.79]) had the two highest ASDR among all countries in 2019. Conversely, countries with the lowest rates were Estonia (354.15 [95%UI: 293.97–425.71]), Montenegro (374.22 [95%UI: 302.81–459.92]), and Latvia (390.72 [95%UI: 320.13–483.54]) (Figure 1C, Supplementary Table 15).

The AAPC in ASPR altered significantly between countries over decades, with Japan (−2.16% [95%CI: −2.31 to −2.02]), New Zealand (−2% [95%CI: −2.13 to −1.88]), and Singapore (−1.92% [95%CI: −2.04 to −1.8]) having the largest decreases. In contrast, Omen (1.09% [95%CI: 0.94 to 1.25]) and Saudi Arabia (0.93% [95% CI:0.77–1.09]) had increasing trends (Supplementary Table 2). The AAPC in ASMR also altered across countries. The largest decreases existed in Singapore (−5.85% [95%CI: −6.17 to −5.53]) and Turkmenistan (−5.16% [95%CI: −5.82 to −4.50]). Instead, the largest increases were found in Belize (1.3% [95%CI: 0.69–1.92]), Nicaragua (1.1% [95%CI: 0.64–1.55]), and Cuba (0.99% [95%CI: 0.71–1.27]) (Supplementary Table 8). Notably, the largest decreases in ASDR were found in Turkmenistan (−4.16% [95%CI: −4.71 to −3.60]), Singapore (−4.00% [95%CI: −4.20 to −3.80]), and China (−3.93% [95%CI: −4.17 to −3.68]). In contrast, Belize (0.70% [95%CI: 0.42–0.99]), Cuba (0.40% [95%CI: 0.27–0.54]), and Kazakhstan (0.37% [95% CI: −0.14 to 0.88]) had largest increasing trends (Supplementary Table 15).

The data on prevalence, mortality, and DALY of overall CRDs, COPD, asthma, pneumoconiosis, ILD and PS, and other CRDs at the global, regional, and national levels can be seen in Supplementary Tables 1–6 prevalence; Supplementary Tables 7–13 deaths; Supplementary Tables 14–20 DALY; Supplementary Figures 1–5 national.

### Disease burden by age, sex, year, and SDI

The detailed description is described in Supplementary material.

### Decomposition analysis by epidemiology drivers and cause of CRDs

**Figure 2:**
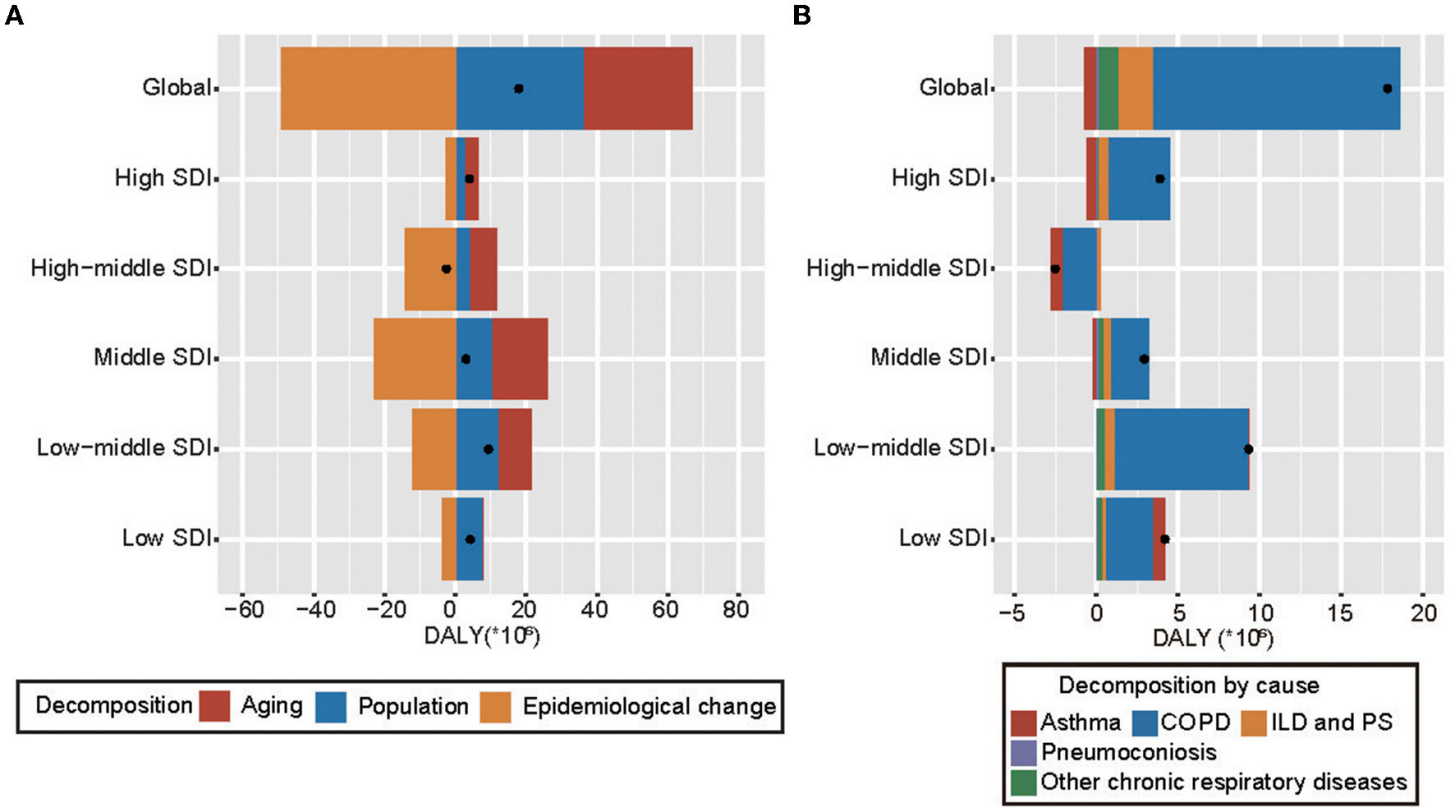
Changes in CRDs DALYs according to global population-level determinants **(A)** and 5 causes **(B)**, by SDI (1990–2019). **(A)** Changes in CRDs DALYs according to global population-level determinants and by SDI (1990–2019). Global population-level determinants include aging, population growth, and epidemiological change. The black dot represents the overall value of change contributed by all 3 components. For each component, the magnitude of a positive value indicates a corresponding increase in CRDs DALYs attributed to the component; the magnitude of a negative value indicates a corresponding decrease in CRDs DALYs attributed to the related component. **(B)** Changes in CRDs DALYs according to the global 5 causes and by SDI (1990–2019). The black dot represents the overall value of change contributed by all 5 causes. For each component, the magnitude of a positive value indicates a corresponding increase in CRDs DALYs attributed to the component; the magnitude of a negative value indicates a corresponding decrease in CRDs DALYs attributed to the related component. CRDs, chronic respiratory diseases; COPD, chronic obstructive pulmonary disease; ILD and PS, interstitial lung disease and pulmonary sarcoidosis; SDI, sociodemographic index; DALY, disability-adjusted life year.

A decomposition analysis of raw DALY was carried out in order to explore to what extent the factors, such as aging, population growth, and epidemiologic changes, shaped CRDs epidemiology (1990–2019) (Supplementary Table 21). Overall, there was an increase in CRDs DALY worldwide and in SDI regions, except in high-middle SDI, and it was most prominent in the low-middle SDI region, which showed the largest increase (Figure 2A). Globally, population growth and aging contributed to 202.17 and 172.91%, respectively, to the increased burden (Supplementary Table 18). The aging was the most prominent, contributing to 532.71% in the middle-SDI region; this decreased to 102.28% in the low-middle-, 96.64% in the high-, and 5.73% in the low-SDI regions, and even negative contribution was found in the high-middle SDI region (−297.03%). The contribution of population growth was similar [increase: middle-SDI (354.31%), low-SDI (179.16%), low-middle SDI (127.01%), high SDI (68.79%); decrease: high-middle SDI (−156.99%)]. The epidemiologic changes have decreased globally, and it was least pronounced in high- and low-SDI regions and more evident in middle-, high-middle-, and low-middle-SDI regions (Figure 2A, Supplementary Table 21). Decomposition analysis in GBD regions exhibited substantial heterogeneity. In particular, although most GBD regions revealed a decrease in epidemiologic changes, there was a GBD region (high-income North America) that showed a significant deviation from the normal trend (Supplementary Table 18). Likewise, as for aging, western, eastern, and central sub-Saharan Africa exhibited deviations and showed decreases. Central Europe and Eastern Europe showed a decreasing trend in population growth, contrary to the general trend.

Decomposition analyses on the five causes of CRDs were also carried out (Supplementary Table 21). From 1990 to 2019, COPD was the primary driver of increased DALY worldwide and in SDI regions, except in high-middle SDI (Figure 2B). Globally, CRDs due to COPD and ILD and PS contributed 85.19 and 11.66%, respectively. While asthma presented as a decreasing factor, contributing −4.32%. Interestingly, the high-middle SDI region showed a downward trend inversely, with COPD and asthma both playing important roles in this decline, contributing 78.89 and 31.89%, respectively. However, in other SDI regions, COPD existed as a growing force. In the increasing SDI regions, the contribution of COPD was the lowest in the low-SDI region (68.69%) and highest in the high-SDI region (97.73%) (Figure 2B and Supplementary Table 21). A similar dual function was seen in asthma. In GBD regions, COPD was still the primary driver of change in CRDs DALY (Supplementary Table 21), but its correlative contribution varied greatly geographically: it was high in high-income Asia Pacific (226.37%) and Western Europe (197.28%), and least pronounced in Central Europe (20.84%).

### Frontier analysis of CRDs

**Figure 3:**
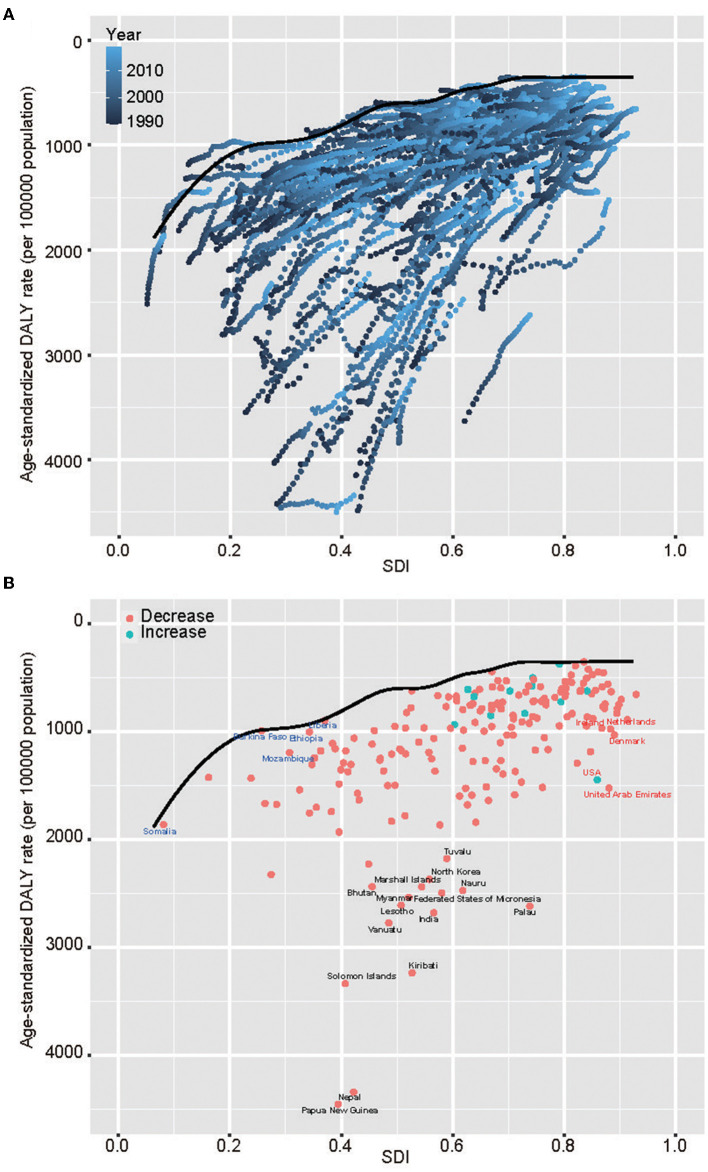
Frontier analysis based upon CRDs ASDR and SDI over decades (1990–2019) and in 2019. **(A)** Frontier analysis based upon CRDs ASDR and SDI (1990–2019). The color scale showed the years from 1990 (dark blue) to 2019 (light blue). The solid black color was used to delineate the frontier. **(B)** Frontier analysis based upon CRDs ASDR and SDI in 2019. The solid black color was used to delineate the frontier, and dots were used to represent countries and territories. The top 15 countries with the largest effective difference were labeled in black; examples of frontier countries with low SDI (< 0.5) and low effective difference are labeled in blue (e.g., Somalia, Burkina, Ethiopia, Mozambique, Liberia), and examples of countries and territories with high SDI (>0.85) and relatively high effective difference were labeled in red (e.g., the USA, Ireland, the Netherlands, Denmark, the UAE). Red dots showed a decrease in CRDs ASDR; blue dots showed an increase in CRDs ASDR from 1990 to 2019.

A frontier analysis was carried out based on ASDR and SDI using data (1990–2019) in order to acquire a better realization of the potential improvement in CRDs DALY rates, which are potentially achievable considering the national development status (Figure 3 and Supplementary Table 22). The top 15 countries with the highest effective difference from the frontier (range of effective difference: 3,599.56–1,657.5) included Papua New Guinea, Nepal, Kiribati, Solomon Islands, Palau, Vanuatu, India, Lesotho, Nauru, Federated States of Micronesia, Myanmar, Marshall Islands, North Korea, Bhutan, and Tuvalu. Compared to other countries, these owned disproportionally higher CRDs DALY rates with comparable sociodemographic resources. The frontier countries with low SDI (<0.5) and low effective difference included Somalia, Burkina, Ethiopia, Mozambique, and Liberia. The USA, Ireland, the Netherlands, Denmark, and the UAE were the examples with high SDI (>0.85) and relatively high effective differences at their development level.

### Risk factors

**Figure 4:**
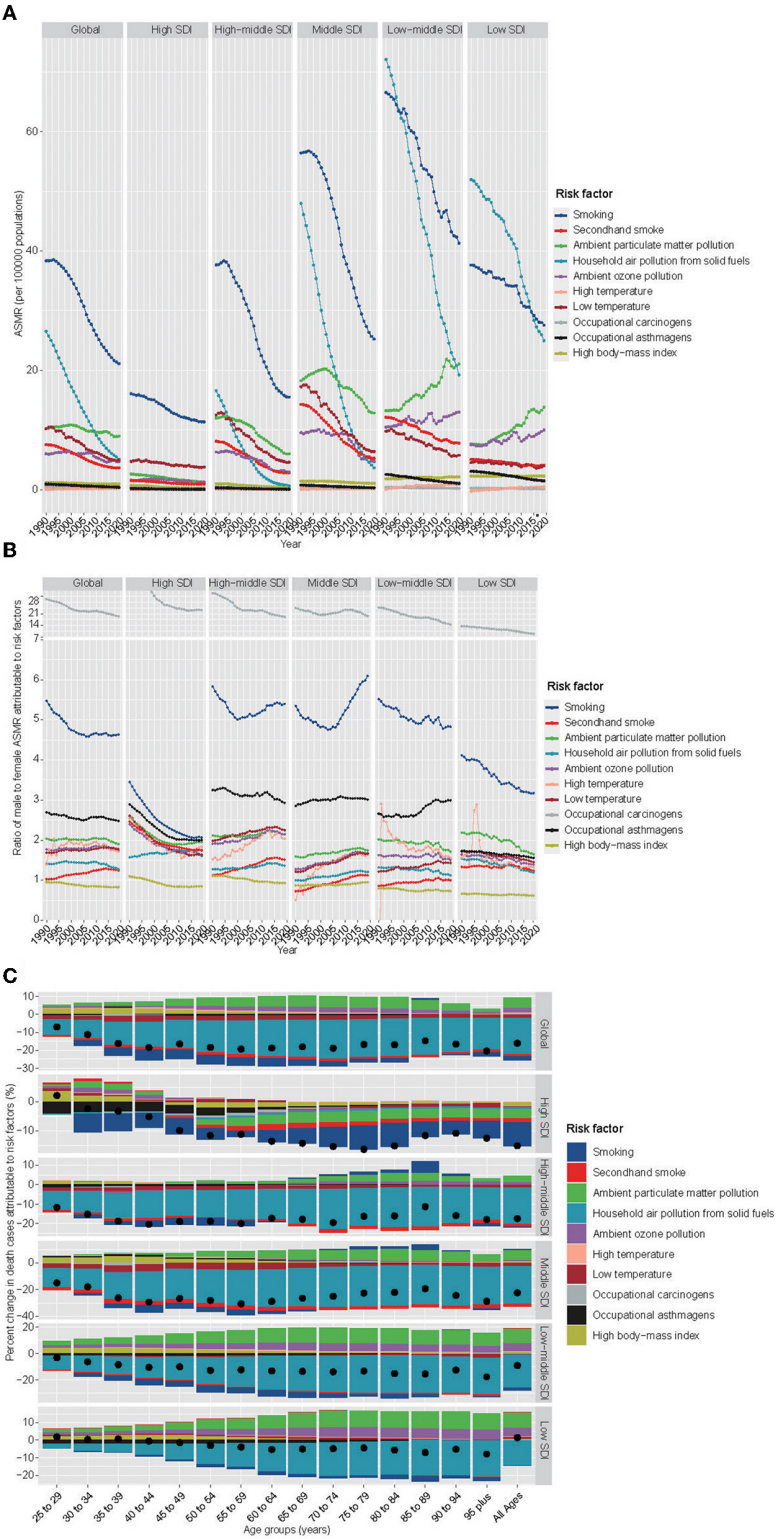
Prominent contribution of RFs to CRDs-related deaths by SDI, sex, and age groups. **(A)** The ASMR attributable to main risk factors by SDI region (1990–2019). **(B)** Male-to-female ratios of ASMR attributable to RFs (1990–2019). **(C)** The percentage changes in mortality attributable to RFs by age group and SDI region (1990–2019). The black dot represents the overall value of change contributed by all RFs. RF, risk factor; CRDs, chronic respiratory diseases; ASMR, age-standardized mortality rate; SDI, sociodemographic index.

Smoking remained the leading factor in 2019 globally in comparison to the top-ranking RFs for ASMR in 1990. Meanwhile, household air pollution from solid fuels (HAPSF) lost its second place in 1990 and fell to third place in 2019, and ambient particulate matter pollution (APMP) took its second place. Generally, the ASMR attributable to RFs, except high temperature, decreased over three decades (Figure 4A). In all regions covering five SDI quintiles (1990–2019), smoking was the top one RF in accord with the globe, though, which rapidly decreased especially in high-middle-, middle-, and low-middle-SDI regions (Figure 4A). Contrary to the overall downtrend, the ASMR attributable to some RFs contradicted the trend. For instance, the ASMR attributable to APMP, ambient ozone pollution (AOP), and high temperature played increasingly important roles, especially in low- and low-middle-SDI regions. Fortunately, the ASMR attributable to HAPSF decreased dramatically in all SDI regions, except in the high-SDI region, where it was insignificant at first and slightly decreased. Globally, the male had a higher ASMR, which could be attributed to all the above-mentioned RFs, except high BMI (ratio <1). The sex ratio for occupational carcinogens (>19) was much higher than other RFs, indicating that occupational carcinogen was a non-negligible source of the sex-dependent differences (Figure 4B). The role of smoking came second; however, there were downtrends of the ratios globally and SDI regionally, except in high-middle and middle-SDI regions. Meanwhile, the gaps enlarged in high-middle and middle-SDI regions among some RFs (secondhand smoke, APMP, HAPSF, AOP, and high and low temperature). It is worth noting that secondhand smoke did not only affect female but also male more, with uptrends of ratios (apart from high SDI) indicating that the sex gap may further widen.

We know that the overall percentage changes in death cases attributable to RFs were on the decline in most age groups and SDI regions over decades (Figure 4C). The RFs of smoking in the high SDI region and HAPSF in the other SDI regions played a crucial role in reducing trends. However, some RFs were on the rise. At the global level, APMP, AOP, high body mass index (BMI), and high temperature by all age groups were increased RFs sequentially. High BMI played a larger part in the youth periods. There was heterogeneity in the RFs contributing to a deterioration in different SDI regions. For example, compared with the general situation, high temperature in the high-SDI and secondhand smoke in the low- and low-middle SDI regions played unique roles.

The situation of the predominant contribution of RFs to CRDs-related DALY is similar to deaths approximately (Supplementary Figure 12). Some differences include (1) smoking was not the most significant RF for ASDR in low SDI regions until 2018, surpassing the RF of HAPSF and (2) except that the ratio of high-BMI was <1 in middle- and low-middle SDI regions, so was the factor of secondhand smoke.

The detailed percentages of mortality and DALY owing to CRDs attributable to RFs for 5 SDI regions and 21 GBD regions and attributable to RFs by age for both sexes in 1990 and 2019 are described in Supplementary Figures 12, 13.

## Discussion

Our results showed increasing trends for the number of prevalence, deaths, and DALY due to CRDs. The most likely causes of this trend were population growth and aging. On the contrary, the ASPR, ASMR, and ASDR decreased, which can be owed to gradually improving treatments and interventions. However, ILD and PS showed increasing trends for ASPR, ASMR, and ASDR (10), in contrast to other types, which may attribute to the increasing awareness and diagnosis level.

Interestingly, Australasia with the second highest ASPR and the third fastest decline in AAPC showed that although its base value was large, the situation improved significantly. The virtuous circle may exist in Eastern Europe with the second lowest ASMR and the second most significant decrease in AAPC. High-income North America was the only region with weak growth in ASDR AAPC, contrary to other regions with downward trends. Meanwhile, an uptrend of ASMR in high-income North America was also witnessed. Therefore, there was heterogeneity in the performance of each GBD region, which requires local measures to solve the problem.

Consistent with previous studies (16–18), China or India tended to be number one in absolute numbers of prevalence, mortality, and DALY due to their huge population base. When the data on mortality and DALY were age-standardized, Nepal's and Papua New Guinea's top two spots were highlighted. As for ASPR, developed countries such as the USA, the UK, and Australia led the way. Prevalence was still very high in developed countries, but their ASMR and ASDR were relatively low due to good healthcare systems. However, this was contrary in most developing countries (e.g., Nepal). For example, Singapore's efforts and gains were seen in the past 30 years, with decreased AAPC of all rates (ASPR, ASMR, and ASDR). China's efforts were laudable concurrently (16, 19), although its absolute value was still high and the AAPC of ASDR dropped significantly in the top three.

Fortunately, the overall trends of CRDs decreased from 1990 to 2019, regardless of SDI and ASR. These were due to the efforts of the WHO and various countries in prevention and treatment. When it came to specific SDI regions and disease levels, it was not always perfect. In the low SDI region, the ASPR of COPD continued to increase over the past few years (5, 9). But of greater concern was the overall increase in ASR of ILD and PS in any SDI regions, obtained from this study and previous studies (10). The level of SDI is related to population health directly, and the huge gap between poverty and wealth in many parts has an effect on health equity (20). This deserves our attention and targeted assistance.

Positive correlations were found between the SDI and ASPR of overall CRDs, COPD, asthma, and ILD and PS. However, the ASMR and ASDR of CRDs, COPD, and asthma were negatively associated with the SDI. It was found that countries with a high SDI showed lower ASMR and ASDR even with higher ASPR, reflecting more accessibility to medical care and healthcare services. As for COPD, countries with low SDI have to reverse the uptrend, while countries with high SDI have to control the huge cases.

Although there were general downward trends of ASDR AAPC in the globe and SDI regions, our decomposition analyses based on the raw value of increased DALY also obtained corresponding gains. The overall increase was actuated by aging and population growth in large part, but epidemiologic changes played negative roles. There were always some regions that deviate from the general trend. Overall, the globe and most SDI regions showed raw DALY growth, except in the high-middle SDI region, a real double-reduction region (raw value and AAPC). Similarly, we developed decomposition analyses by the five causes. Globally, COPD was the leading driver of increased DALY, but asthma was present as the only decreasing factor. But COPD and asthma played different roles in different SDI regions. COPD is attributed to a decline in high-middle SDI (9), and asthma is boosted with uptrends in low and low-middle SDI regions. These regions certainly deserve more attention where all causes were drivers for increased DALYs, showing a general inability to cope with CRDs (21).

As for national development status, a frontier analysis was built using data from ASDR and SDI. Despite our analysis highlighting a formidable challenge, this study provided a more optimistic evaluation, suggesting unrealized opportunities to narrow the DALYs differences. Notably, those countries with low SDI existed as frontier countries, which showed surprisingly outstanding performances despite limited resources and conditions and deserved to be set up as exemplars of optimization of health outcomes in a low-resource environment. On the contrary, some high SDI countries underperformed (e.g., the USA). Future work should be undertaken to identify drivers of success in exemplar countries and forces hampering progress in laggard countries; addressing this knowledge gap will likely be useful in taking efforts to alleviate the burden.

Although many measures and policy actions have been taken within and across countries (22, 23), the leading cause of CRDs mortality and DALY was smoking globally in GBD 2019. Smoking not only affects the health of smokers but also endangers other lives due to secondhand smoke (24). New assessments on the effects of exposure to secondhand smoke (25), and of new smoking forms (e.g., electronic cigarettes) (26), deserve heightened public awareness. Meanwhile, as for air pollution in ASMRs, APMP and HAPSF were the second and third causes, switching the order over decades. With regard to HAPSF, the percentage of ASMR and ASDR surpassed smoking in low SDI regions in the study, consistent with previous studies (27, 28). The burning of solid fuels (29–31), high population, and limited medical support in countries with lower SDI have led to an uptrend in APMP (31, 32). In this study, the ASMR and ASDR attributable to high temperature increased over decades. The low temperature showed a decrease in ASMR and ASDR, although its percentage was still higher. The shift might be attributed to climate change (20, 33). Previous studies have shown that high BMI had a great effect on CRDs (e.g., COPD and asthma) (34). High BMI played a larger part in the young and middle-aged periods in this study.

Prior to this study, previously published articles clarified the disease burden to some extent. A systematic analysis (Global Burden of Disease Study 2017) performed by Li et al. (5) focused on mortality, DALY, and risk factors, without the prevalence analysis unfortunately. Some GBD CRDs Collaborators (1) co-created another systematic analysis (GBD 2017), which included the prevalence component, but its analysis was limited to the GBD region, and the association with SDI was not further analyzed. But these were all based on GBD 2017, the recent data being GBD 2019. GBD 2019 updates and expands beyond GBD 2017 in many ways (15). Gan et al. (6) performed a relatively recent article derived from GBD 2019, which only discussed incidence and mortality without the national level, as well as the association with SDI, sex, and age. This article emphasizes again that smoking is the most harmful to CRDs, but this argument is almost universally familiar, and the analysis of dynamic changes of other risk factors was inadequate. Compared with Hui Gan's research, our study supplemented the information on prevalence and DALY details and emphasized the increasing importance of air pollution in causing CRDs. The main causes of air pollution included APMP and HAPSF. In the context of the obvious improvement of ASMR and ASDR of smoking and HAPSF, APMP became more prominent in ASMR and ASDR, especially in low-middle and low SDI regions, even showing an upward trend. This needs to be focused in the future. Meanwhile, the analysis limited to a certain subtype (COPD, asthma, ILD, and so on) was relatively limited (7–11). These studies were not able to show a comparative change in trends among subtypes of CRDs. In this study, decomposition analysis was used to demonstrate the changes in DALY number and show clearly that the burden of asthma was significantly improved compared to others. Decomposition analysis also reflected both aging and population growth in the increasing weight of changes in DALY number, except in high-middle SDI region. Frontier analysis of ASDR was introduced innovatively to show the potential for progress in the country with various SDI level, given the current conditions.

Although there is some progress in our study, the common limitation of GBD study specific to chronic respiratory diseases was inevitable (35, 36). First, CRD diagnoses require clear diagnostic criteria, which are often not standardized worldwide. If estimates were all recorded in a standard procedure, uncertainty could be diminished (35). Second, CRDs definition is highly dependent on medical expertise and equipment, especially for ILD and PS, and it is likely that a significant source of geographic heterogeneity is the lack of adequate diagnosis in underdeveloped regions (5). Additionally, statistics systems and civil registration are pivotal sources of vital statistics for mortality rates (37), but the population coverage with these systems has been disappointing (5). These aspects deserve further attention in future GBD construction.

## Conclusion

This study revealed that the ASR (ASPR, ASMR, and ASDR) decreased over three decades, with aging and population growth, nevertheless, the number of global prevalence, mortality, and DALY from CRDs continued to increase from 1990 to 2019. Meanwhile, geographical heterogeneity can be witnessed in the CRDs burden, which was closely relevant to the SDI level. Smoking and air pollution were the most predominant risk factors for mortality and DALY, equally important in other risk factors (non-optimal temperature, occupational exposure, and high BMI). No matter where we are, we all have to receive more attention with supportive policies and strive forward, in order to have a better future.

## Data availability statement

The original contributions presented in the study are included in the article/Supplementary material, further inquiries can be directed to the corresponding authors.

## Ethics statement

The GBD study's protocol has been approved by the Research Ethics Board at the University of Washington. The GBD shall be conducted in full compliance with the University of Washington policies and procedures, as well as applicable federal, state, and local laws. All methods were carried out in accordance with Guidelines for Accurate and Transparent Health Estimates Reporting (GATHER) guidelines for reporting health estimates.

## Author contributions

XC, Y-FC, Q-WZ, and C-WZ conceived and designed the study, drafted the manuscript, and revised the scientific and factual content of the manuscript. XC, Q-WZ, Y-YF, Y-ZL, and LC collected and analyzed the data. All authors read and approved the final manuscript.
