# Supplementary material for: Global, regional, and national burden of chronic respiratory diseases and associated risk factors, 1990–2019: Results from the Global Burden of Disease Study 2019
Source: Front Med (Lausanne). 2023 Mar 28;10:1066804. doi: 10.3389/fmed.2023.1066804 (PMC10088372; doi:10.3389/fmed.2023.1066804)
Supplement: Supplementary file 1 [file Data_Sheet_1.ZIP › Additional file 1.Supplemental Material.docx]

**Supplemental Methods**

**Data source and definition**

Our study on the burden of chronic respiratory diseases (CRDs) and risk factors did not involve human subjects, and the data were retrieved from the Global Health Data Exchange GBD Results Tool (http://ghdx.healthdata.org/gbd-results-tool). The GBD 2019, conducted by the Institute of Health Metrics and Evaluation(IHME), estimated prevalence of exposure and attributable deaths and DALYs for 23 age groups; males, females, and both sexes combined; with risk factors of 369 diseases in 204 countries from 1990 to 2019(1, 2). GBD 2019 complies with the Guidelines for Accurate and Transparent Health Estimates Reporting (GATHER) statement(3).

According to the GBD Data Dictionary, CRDs include the following five categories: chronic obstructive pulmonary disease(COPD), asthma, interstitial lung disease and pulmonary sarcoidosis(ILD&PS), pneumoconiosis, and other chronic respiratory diseases. COPD(4) is a common, preventable, and treatable disease that is characterized by persistent respiratory symptoms and airflow limitation that is due to airway and/or alveolar abnormalities usually caused by significant exposure to noxious particles or gases. Asthma(5) is a heterogeneous disease defined by the history of respiratory symptoms (e.g., wheeze, shortness of breath, chest tightness, and cough) that vary over time and in intensity, together with variable expiratory airflow limitation. Airflow limitation may later become persistent. Interstitial lung diseases (ILDs)(6), also known as diffuse parenchymal lung diseases, are a group of diverse disorders affecting the pulmonary interstitium, composed of alveolar epithelium, pulmonary capillary endothelium, basement membrane, and perivascular and perilymphatic tissues, including all forms of ILD and pulmonary sarcoidosis(PS), while pneumoconiosis is not included here. Pneumoconiosis(7) is inclusive of a group of serious occupational diseases associated with the inhalation of mineral dusts and corresponding reactions of lung tissues, including asbestosis, coal workers’ pneumoconiosis, silicosis, and other types of pneumoconiosis. The other CRDs(8) category comprised obstructive sleep apnoea; vasomotor and allergic rhinitis; chronic rhinitis; nasopharyngitis and pharyngitis; chronic sinusitis; nasal polyposis; other and unspecified disorders of the nose and nasal sinuses; chronic diseases of the tonsils and adenoids; chronic laryngitis and laryngotracheitis; diseases of the vocal cords and larynx, not elsewhere classified; other diseases of the upper respiratory tract; airway disease due to specific organic dust; hypersensitivity pneumonitis due to organic dust; respiratory conditions due to inhalation of chemicals, gases, fumes, and vapours; respiratory conditions due to other external agents; respiratory conditions due to other specified external agents; pulmonary eosinophilia, not elsewhere classified; pleural effusion in conditions classified elsewhere; pleural plaque with presence of asbestos; and pleural plaque without asbestos. Notably, infectious diseases such as tuberculosis were not included, even though it is one of the most prevalent respiratory conditions.

The Socio-demographic index(SDI) is a composite indicator of income per capita, average educational attainment, and total fertility rates(9). Index values range from 0 (lowest income, fewest years of schooling, and highest fertility) to 1 (highest income, most years of schooling, and lowest fertility). Countries and territories were classified as regions with a high, high-middle, middle, low-middle, or low SDI. The cut-off values used to determine regions for analysis were computed using country level estimates of the SDI for the year 2019, excluding countries with populations less than one million.

Risk factors were defined in the comparative risk assessment framework of the GBD 2019, which includes environmental and occupational risks (household air pollution from solid fuels, ambient particulate matter, ambient ozone pollution), behavioural risks (tobacco, including smoking and secondhand smoke), and metabolic risks (high body mass index). The risk factor hierarchy and accompanying definitions of exposure were clarified in the previous study(10).

**Measures of burden**

Measures of burden at the global, regional, and national levels included prevalence, mortality and DALYs due to CRDs. The prevalence of CRDs was estimated by location, age, sex, and year using DisMod-MR version 2.1, an updated Bayesian regression analytical tool used in GBD to estimate non-fatal health outcomes. Death due to CRDs was estimated by the GBD using cause of death ensemble model. DALYs were calculated through summation of years lived with disability and years of life lost. The years lived with disability is a measure of disease burden that represents the years lived with CRDs; it considers the duration of time living with the disease and related disability weights to reflect the underlying severity of CRDs. The years of life lost represented the years lost due to premature mortality caused by CRDs. All measures are reported as raw values and age-standardized rates per 100,000 population, where age standardization was based on the World Health Organization world population standard age structure. Crude estimates provide the real situation, which is useful for policy makers and practicing physicians, whereas age-standardized estimates allow comparisons over time and between countries after adjusting for the differences in the age structure and population growth of the population for a specific year(8). The estimates of GBD for a disease burden are described in terms of 95% UIs, with the actual value of a parameter at 95% probability. UIs consider the variance in the estimation of the parameter, as well as the uncertainty in model selection, data collection, and other sources in the process of parameter estimation. Average annual percentage change(AAPC) of age standardized rates is described in 95% confidence interval(CI), obtained from the linear model.

**Statistical analyses**

**Decomposition analysis:**

The analytic approach to identify the additive contribution of the effect of the differences in factors in 2 populations to the difference in their overall value. CRDs DALY by age structure, population growth, and epidemiologic changes. Similarly, decomposition of CRDs DALY by the 5 causes allows the quantification of the contribution of each cause to the overall CRDs DALY.

We first used the decomposition methodology of Das Gupta(11-13) to decompose CRDs DALYs by population age structure, population growth, and epidemiologic changes. The number of DALYs at each location was obtained from the following formula: DALY _ay, py, ey_ = $\sum_{i=1}^{20} ($a _i, y_ * p _y_ * e _i, y_)

Where DALY _ay, py, ey_ represented DALYs based on the factors of age structure, population, and DALYs rate for specific year y; a _i_ _y_ represents the proportion of population for the age category i of the 20 age categories in given year y; p _y_ represents the total population in given year y; and e _i, y_ represents DALYs rate given age category i in year y. The contribution of each factor to the change in DALYs from 1990 to 2019 was defined by the effect of one factor changing while the other factors were held constant. For example, the effect of age structure was calculated as:

[(DALY _a2016, p1990, e1990_ + DALY _a2016, p2016, e2016_)/3+ (DALY _a2016, p1990, e2016_ + DALY _a2016, p2016, e1990_)/6] - [(DALY _a1990, p2016, e2016_ + DALY _a1990, p1990, e1990_)/3+ (DALY _a1990, p2016b, e1990_ + DALY _a1990, p1990, e2016_)/6]

We additionally decomposed the changes in CRD DALYs numbers due to COPD, pneumoconiosis, asthma, ILD and PS and other CRD. The all-cause CRD DAYs change equals to the sum of change in CRD DALYs due to these 5 causes proportion of change attributable to specific cause was calculated as the quotient of the change of cause-specific CRD DALYs to the change of all-cause CRD DALYs.

**Frontier analysis**

In order to evaluate the relationship between burden of CRD and socio-demographic development, we applied a frontier analysis as a quantitative methodology to identify the lowest potentially achievable age-standardized DALYs rate on the basis of development status as measured by the Socio-demographic Index (SDI). The DALYs frontier pinpoints the minimum DALYs that could be attained for every country or territory given its SDI. Distance from the frontier is termed effective difference; a large effective difference from the frontier suggests there may be unrealized opportunities for gains or improvement (reduction in CRD DALYs) that should be possible based on the country or territory’s place on the development spectrum. A data envelope analysis, which allows for the delineation of non-linear frontiers, utilizing the free disposal hull method was developed to produce a frontier for age-adjusted CRD DALYs by SDI(4) using data from 1990-2019. In order to account for uncertainty, we used 100 bootstrapped samples of the data, randomly sampling with replacement from all countries and territories in all years. Mean CRD DALYs at each SDI value from the bootstrapped samples was computed. LOESS regression with local polynomial degree of 1 and span of 0.2 was then developed to generate a smoothed frontier(14). To exclude influence of outliers, super-efficient countries were excluded in the generation of the frontier(14). To understand the relationship of age-standardized CRD DALYs rates vis-à-vis the frontier in 2019, we calculated the effective difference (the absolute distance from the frontier) using 2019 SDI and age-standardized CRD DALYs rate data point for each country or territory. Countries or territories with lower DALYs than the frontiers were assigned a zero distance.

**Supplemental Result**

**Sex and Age Patterns**

The age-specific prevalence (figure S6) and the age-sex-specific prevalence (figure S7) of each CRD in 2019 were highly variable. Before 50 years old, asthma predominated in overall prevalence, and then COPD began to dominate. Asthma prevalence peaked at 5-9 years of age among children, dropping to a trough period（25-29 age group）, and then continued to rise with age. Asthma prevalence was greater among boys than among girls up to 19 years of age, however, from age 20 years onwards, asthma was consistently higher in females. Since childhood, COPD increased monotonically, while the contributions of ILD&PS and other CRDs decreased relative to all CRDs (figure S6). Interestingly, similar to asthma, up to a certain age (COPD-39; Asthma-19; ILD&PS-64; Pneumoconiosis-29), female dominated in ASPR of all types of CRDs, and then male became the majority.(figure S7). Meanwhile, the age-specific mortality/DALY(figure S6), as well as the age-sex-specific mortality/DALY (figure S7), of each CRD in 2019 were varied in some ways. COPD had dominated in the trend of mortality in both sexes, accounting for an increasing proportion with increasing age. Asthma, ILD&PS, and other CRDs also increased with age approximately, but the overall proportions were limited. Male dominated across all ages in ASMR of COPD and pneumoconiosis in 2019. In male individuals and overall population, the ASDR in 2019 increased with age up to the 85-89 age group, with a declining trend observed up to the oldest groups. This peak could not be seen in the female population curve.

**CRDs Burden by SDI and Year**

Associations between the SDI and ASRs of prevalence, mortality and DALY due to overall CRDs, COPD, asthma, ILD&PS, pneumoconiosis and others from 1990 to 2019 are shown in figure S8. For overall CRDs, the ASRs of three categories showed a downward trend between 1990 and 2019 globally. The trends in 5 SDI regions were consistent with the overall trend according to AAPC (Table 1.Table S1-2,Table S7-S8,Table S14-15). For COPD, asthma and pneumoconiosis, downtrend between the rates and year was seen, apart from the uptrend of prevalence of COPD in low SDI region (AAPC 0.05[95% UI 0.02-0.09]). Nevertheless, the rates of ILD&PS showing increasing trends from 1990 to 2019 in all SDI regions(Table S1,S5,S7,S9,S14,S18). Positive correlations were seen in CRDs, COPD, Asthma, ILD&PS(P＜0.001) between the ASRs and SDI. For pneumoconiosis, a positive correlation between ASMR and SDI was seen, however, with no statistical significance(P=0.333). The ASMR of CRDs, COPD and asthma were negatively associated with the SDI (P＜0.001). Similarly, the relation between the SDI and ASDR resembled those between the SDI and ASMR of the three types (CRDs, COPD and Asthma). Unlike those diseases, ILD&PS showed a positive correlation between SDI and rates (ASMR&ASDR), with no statistical significance(P=0.273/0.308) (Figure S9-11).

**Risk factors**

Meanwhile, the percent of attributable mortality and DALY for risk factors in Globe, 5 SDI regions and 21 GBD regions in 1990 and 2019 can be seen (Figure S13). The data from the figure was very informative, and at least we get these things out of it:(1) As time went by, the percent of smoking and secondhand smoke decreased in most SDI and GBD region;(2) Ambient particulate matter pollution shared increased in percent of risk factors, however, household air pollution from solid fuels was on the decline; (3)Even with the change of times, the risk factor proportion of smoking was still not as high as the percent of household air pollution from solid fuels in low SDI region(e.g., most Sub−Saharan Africa) .(4) Low temperatures apparently caused more mortality and DALY than high temperatures. In addition, the percentage in 1990/2019 revealed various patterns across age group(Figure S14). The highest percent of attributable deaths for smoking was found in 65-69 years (1990) and 70-74 years (2019), respectively. As for DALY, the peak was seen in 70-74 years in both 1990 and 2019. It was difficult to specify the exact age, however, there was a peak of percentage in 70 years approximately. These age peaks could also be seen for other risk factors, however, the peaks age of each risk factor were varied.

**Supplemental Discussion**

To investigate the factors explaining the changes in prevalence, mortality and DALY due to CRDs from 2019, the effects of sex and age were examined. Since childhood, COPD increased monotonically in ASRs in 2019 roughly, while the contributions of asthma, ILD&PS, and other CRDs decreased relatively. Exactly, COPD, the most pervasive CRDs and the leading contributor, increased in rank from 11 to 6th in DALYs among all causes from 1990 to 2019(1, 8). In ASPR of CRDs, male and female dominance shifted after a certain age period. These tended to be dominated by female in the early periods and male in the later periods, with differences in specific switching ages. Asthma might accounted for the majority of CRDs in youth(15), and females were the most likely to suffer from asthma. Male dominated across all ages in ASMR of COPD and pneumoconiosis in 2019. This was consistent with previous knowledge that males were more likely to have a history of smoking and relative occupational exposure, whose variation could be partially explained by the variations in the summary exposure values between the sexes(16).And males with COPD have worse survival might explain the higher disease burden to some extent, with possible support for phenotypic differences between sexes(16).As for other types, male and female dominance alternated dynamically with age in ASMR and ASDR.

**Supplemental References**

S1. Collaborators GDaI. Global burden of 369 diseases and injuries in 204 countries and territories, 1990-2019: a systematic analysis for the Global Burden of Disease Study 2019. Lancet (London, England). 2020;396(10258):1204-22.

s2. Collaborators GRF. Global burden of 87 risk factors in 204 countries and territories, 1990-2019: a systematic analysis for the Global Burden of Disease Study 2019. Lancet (London, England). 2020;396(10258):1223-49.

s3. Stevens G, Alkema L, Black R, Boerma J, Collins G, Ezzati M, et al. Guidelines for Accurate and Transparent Health Estimates Reporting: the GATHER statement. Lancet (London, England). 2016;388(10062):e19-e23.

s4. Global strategy for the diagnosis，management and prevention of chronic obstructive pulmonary disease 2022 report[EB/OL] (2021-11-15）<https://goldcopd.org/gold-reports/>.

s5. Reddel H, Bacharier L, Bateman E, Brightling C, Brusselle G, Buhl R, et al. Global Initiative for Asthma Strategy 2021: Executive Summary and Rationale for Key Changes. American journal of respiratory and critical care medicine. 2022;205(1):17-35.

s6. Ma X, Zhu L, Kurche J, Xiao H, Dai H, Wang C. Global and regional burden of interstitial lung disease and pulmonary sarcoidosis from 1990 to 2019: results from the Global Burden of Disease study 2019. Thorax. 2021.

s7. Shi P, Xing X, Xi S, Jing H, Yuan J, Fu Z, et al. Trends in global, regional and national incidence of pneumoconiosis caused by different aetiologies: an analysis from the Global Burden of Disease Study 2017. Occupational and environmental medicine. 2020;77(6):407-14.

s8. Collaborators GCRD. Prevalence and attributable health burden of chronic respiratory diseases, 1990-2017: a systematic analysis for the Global Burden of Disease Study 2017. The Lancet Respiratory medicine. 2020;8(6):585-96.

s9. Global age-sex-specific fertility, mortality, healthy life expectancy (HALE), and population estimates in 204 countries and territories, 1950-2019: a comprehensive demographic analysis for the Global Burden of Disease Study 2019. Lancet (London, England). 2020;396(10258):1160-203.

s10. Adeloye D, Song P, Zhu Y, Campbell H, Sheikh A, Rudan I. Global, regional, and national prevalence of, and risk factors for, chronic obstructive pulmonary disease (COPD) in 2019: a systematic review and modelling analysis. The Lancet Respiratory medicine. 2022.

s11. Gupta P. Standardization and decomposition of rates: a users's manual. US bureau of the census, current population reports. 1993:123-86.

s12. Das Gupta P. Standardization and decomposition of rates from cross-classified data. Genus. 1994;50(3-4):171-96.

s13. Chevan A, Sutherland M. Revisiting Das Gupta: refinement and extension of standardization and decomposition. Demography. 2009;46(3):429-49.

s14. Collaborators. GHAaQ. Healthcare Access and Quality Index based on mortality from causes amenable to personal health care in 195 countries and territories, 1990-2015: a novel analysis from the Global Burden of Disease Study 2015. Lancet (London, England). 2017;390(10091):231-66.

s15. Pearce N, Aït-Khaled N, Beasley R, Mallol J, Keil U, Mitchell E, et al. Worldwide trends in the prevalence of asthma symptoms: phase III of the International Study of Asthma and Allergies in Childhood (ISAAC). Thorax. 2007;62(9):758-66.

s16. Zou J, Sun T, Song X, Liu Y, Lei F, Chen M, et al. Distributions and trends of the global burden of COPD attributable to risk factors by SDI, age, and sex from 1990 to 2019: a systematic analysis of GBD 2019 data. Respiratory research. 2022;23(1):90.
