# Supplementary material for: Global, regional, and national burden of chronic respiratory diseases and associated risk factors, 1990–2019: Results from the Global Burden of Disease Study 2019
Source: Front Med (Lausanne). 2023 Mar 28;10:1066804. doi: 10.3389/fmed.2023.1066804 (PMC10088372; doi:10.3389/fmed.2023.1066804)
Supplement: Supplementary file 1 [file Data_Sheet_1.ZIP › Additional file 2.figures.docx]

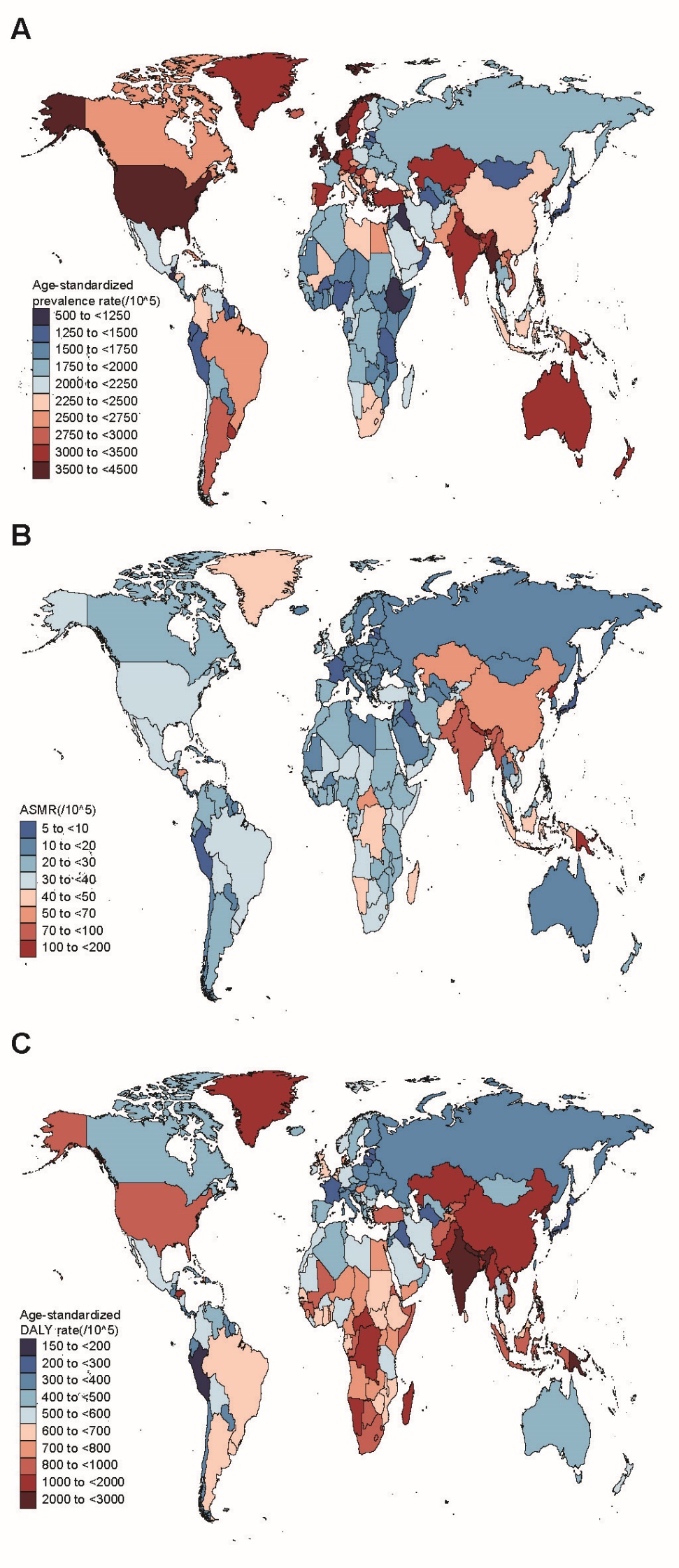


**Figure S1.** Global age-standardized prevalence (A), mortality (B) and DALY (C) rate of COPD in 204 countries and territories (2019).

COPD: chronic obstructive pulmonary disease; ASMR: age standardized mortality rate; DALY: disability-adjusted life year.


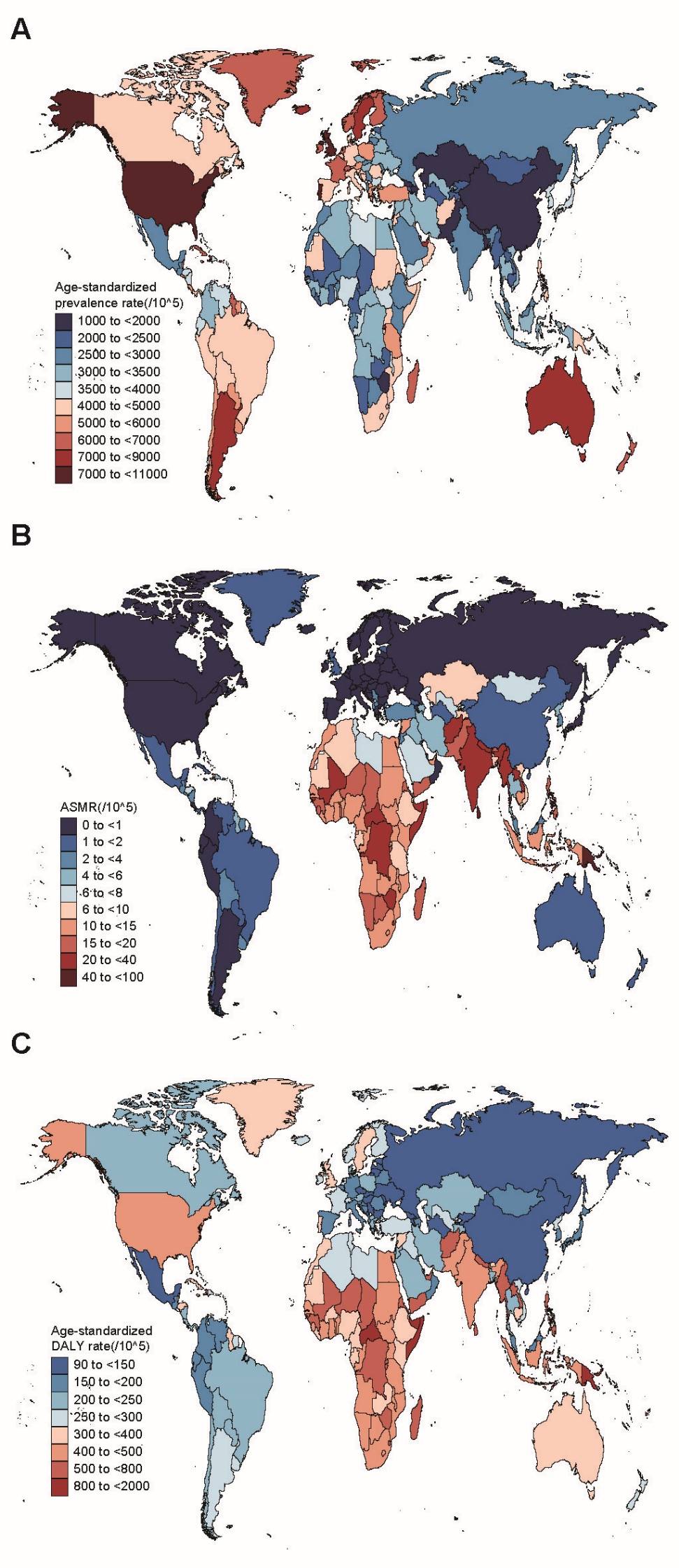


**Figure S2.** Global age-standardized prevalence (A), mortality (B) and DALY (C) rate of asthma in 204 countries and territories(2019).

ASMR: age standardized mortality rate; DALY: disability-adjusted life year.


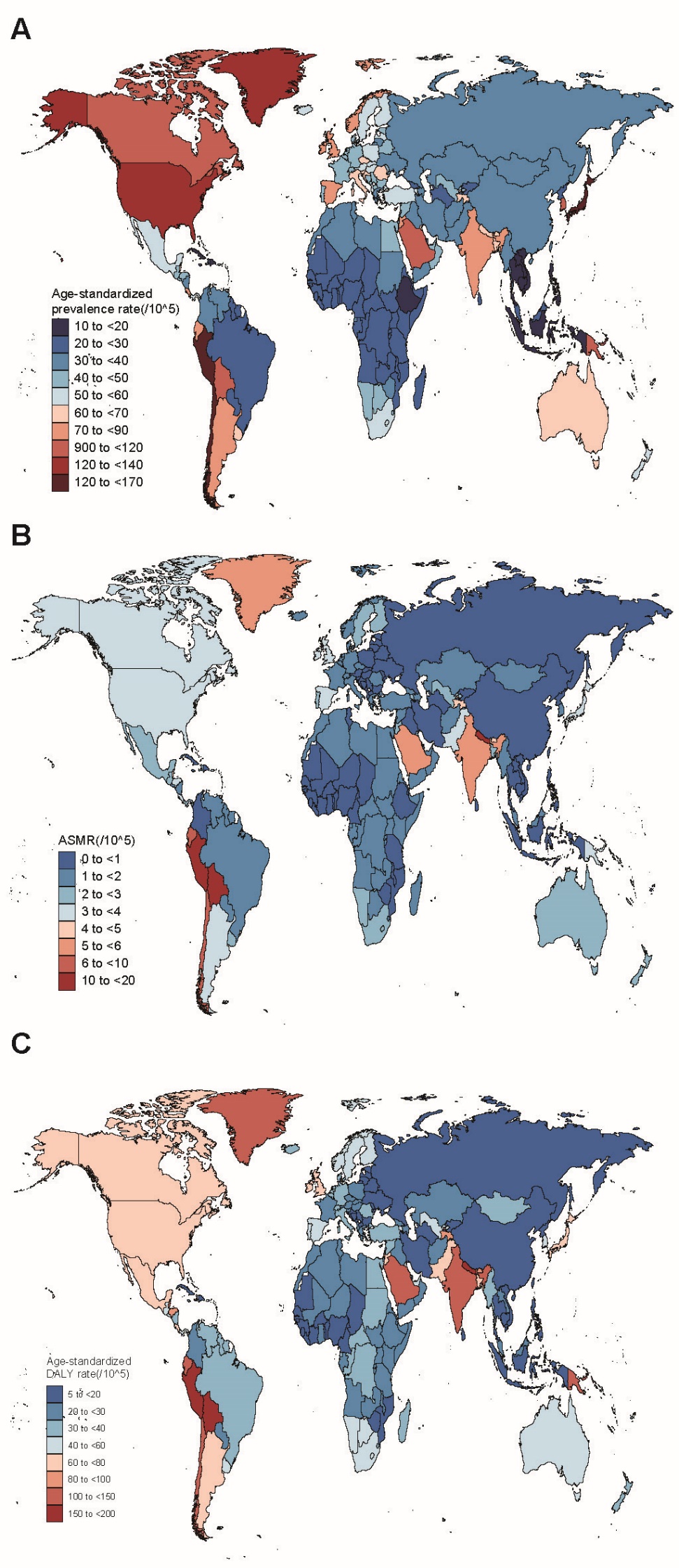


**Figure S3.** Global age-standardized prevalence (A), mortality (B) and DALY (C) rate of ILD&PS in 204 countries and territories(2019).

ILD&PS: interstitial lung disease and pulmonary sarcoidosis; ASMR: age standardized mortality rate; DALY: disability-adjusted life year.


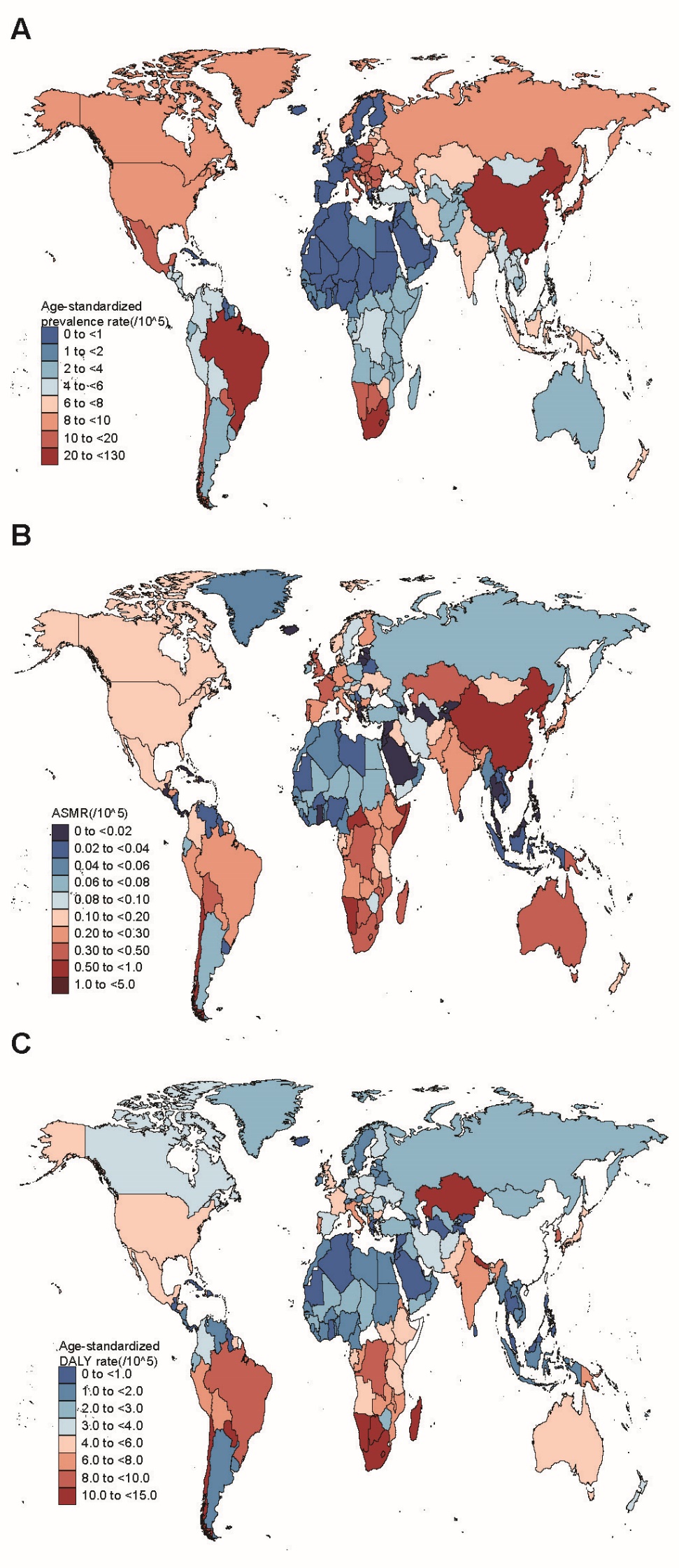


**Figure S4.** Global age-standardized prevalence (A), mortality (B) and DALY (C) rate of PNE in 204 countries and territories (2019).

PNE: pneumoconiosis; ASMR: age standardized mortality rate; DALY: disability-adjusted life year.


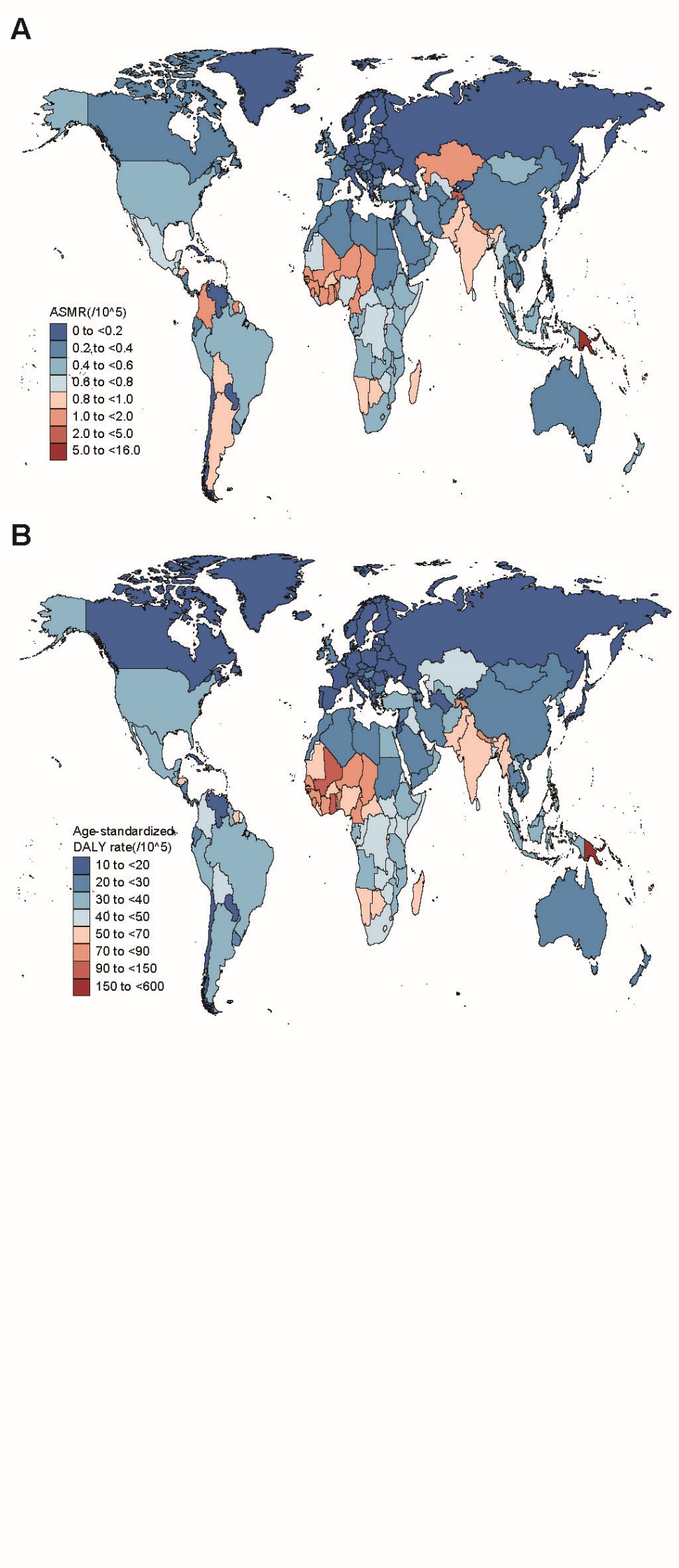


**Figure S5.** Global age-standardized mortality (A) and DALY (B) rate of other CRDs in 204 countries and territories (2019). CRDs: chronic respiratory diseases; ASMR: age standardized mortality rate; DALY: disability-adjusted life year.


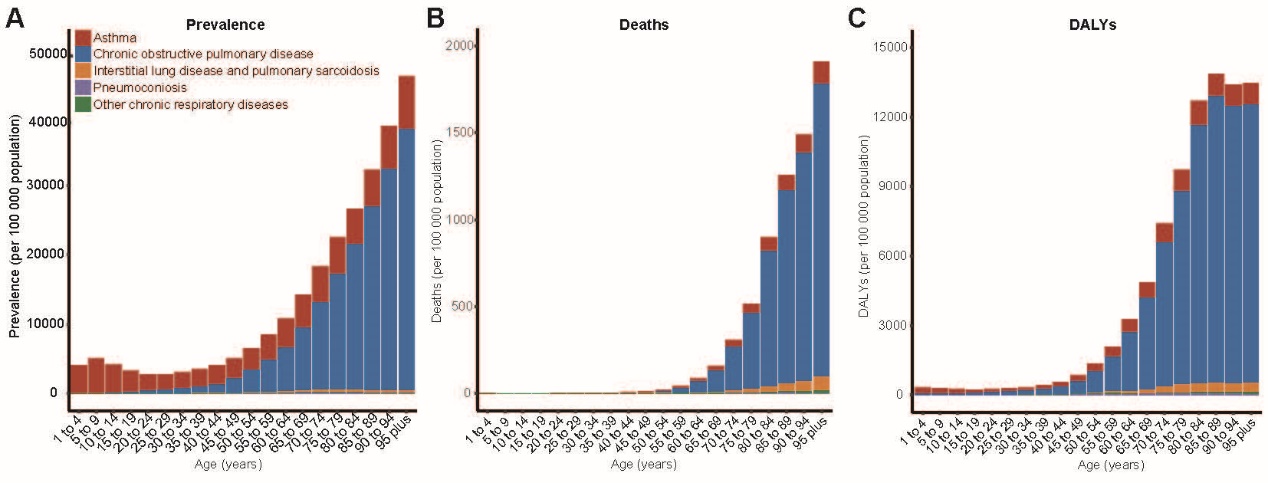


**Figure S6.** Global age-specific prevalence (A), mortality (B) and DALY (C) of CRDs by category for both sex in 2019. CRDs: chronic respiratory diseases; DALY: disability-adjusted life year.


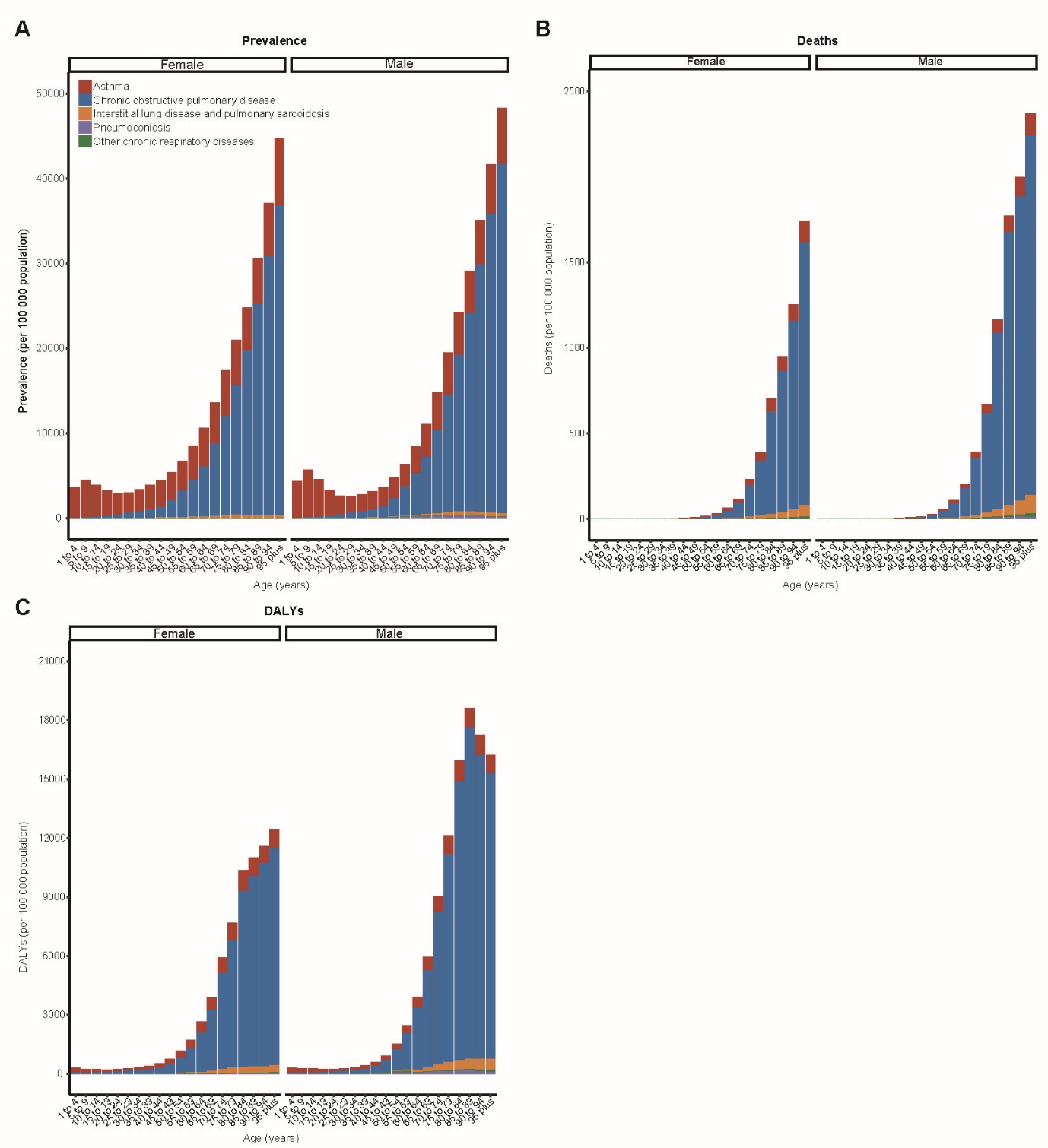


**A**

**Figure S7.** Global age-sex-specific prevalence (A), mortality (B) and DALY (C) of CRDs by disease category in 2019. CRDs: chronic respiratory diseases; DALY: disability-adjusted life year.


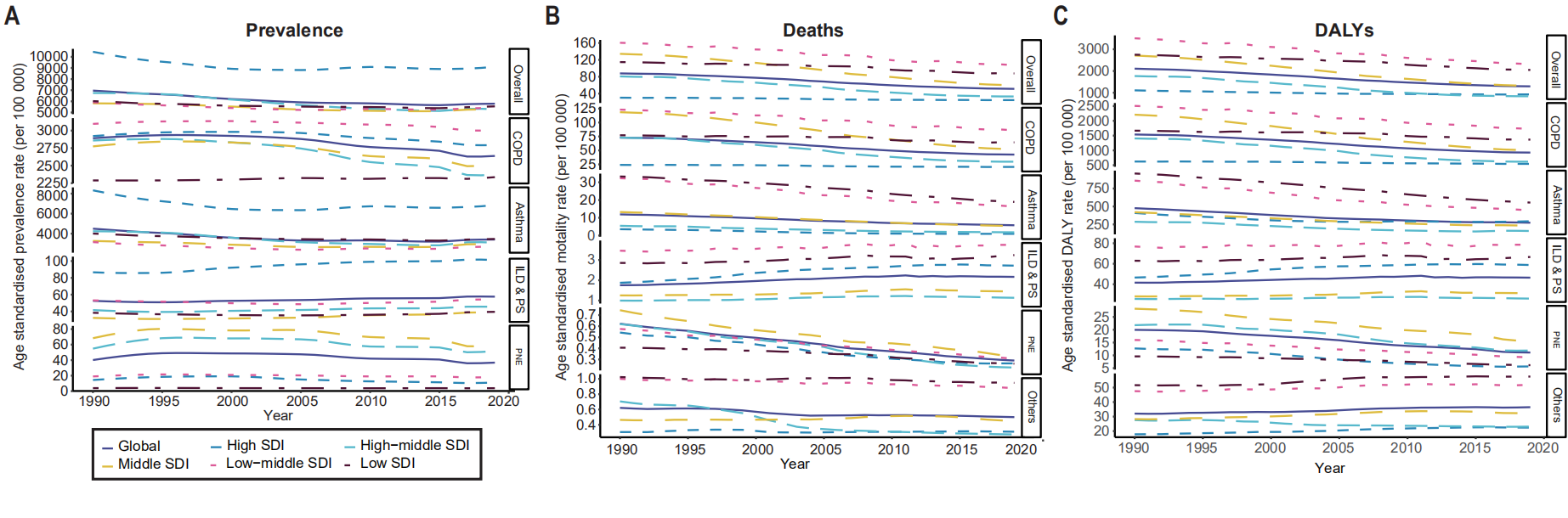


**Figure S8.** Age-standardized prevalence (A), mortality (B) and DALY (C) rates of specific CRDs grouped by SDI (1990-2019). CRDs include overall CRDs, COPD, asthma, ILD&PS, PNE, and others.

DALY: Disability-Adjusted Life Year; SDI: Socio-demographic index; CRDs: chronic respiratory diseases; COPD: chronic obstructive pulmonary disease; ILD&PS: Interstitial lung disease and pulmonary sarcoidosis; PNE: pneumoconiosis.


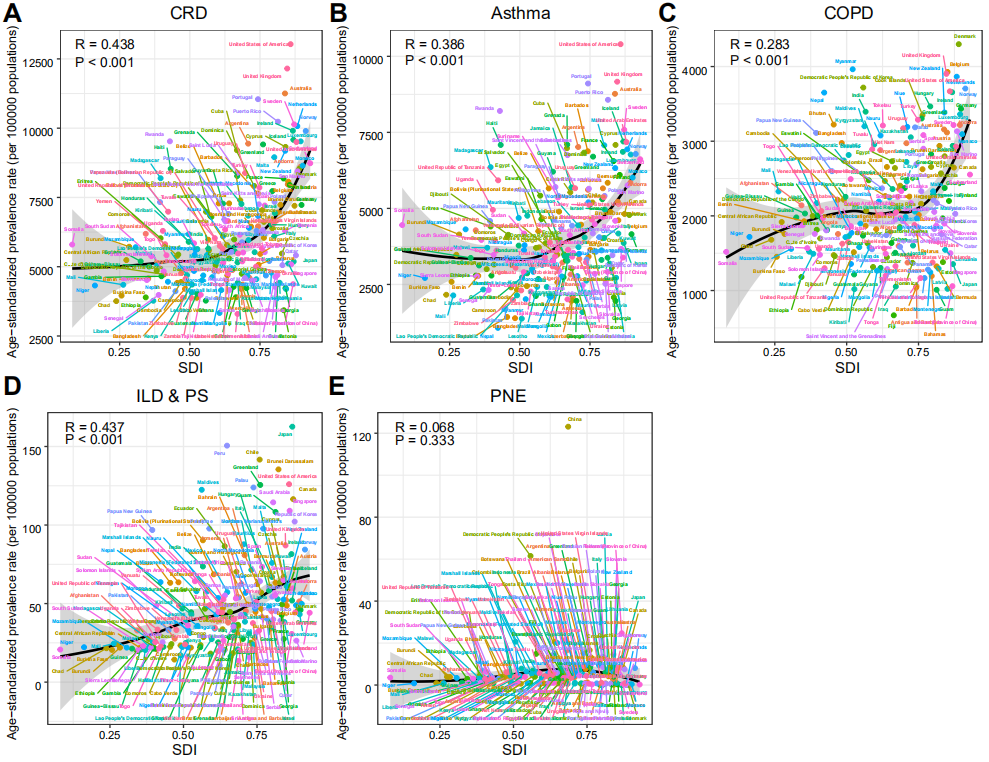


**Figure S9.** The correlation between the SDI and ASPR of overall and specific CRDs in 2019.

(A) CRDs, (B)asthma, (C) COPD, (D)ILD&PS and (E)PNE. The colored dots and lines represent countries in each SDI quintile.

SDI: Socio-demographic index; ASPR: age-standardized prevalence rate; CRDs: chronic respiratory diseases; COPD: chronic obstructive pulmonary disease; ILD&PS: Interstitial lung disease and pulmonary sarcoidosis; PNE: pneumoconiosis.


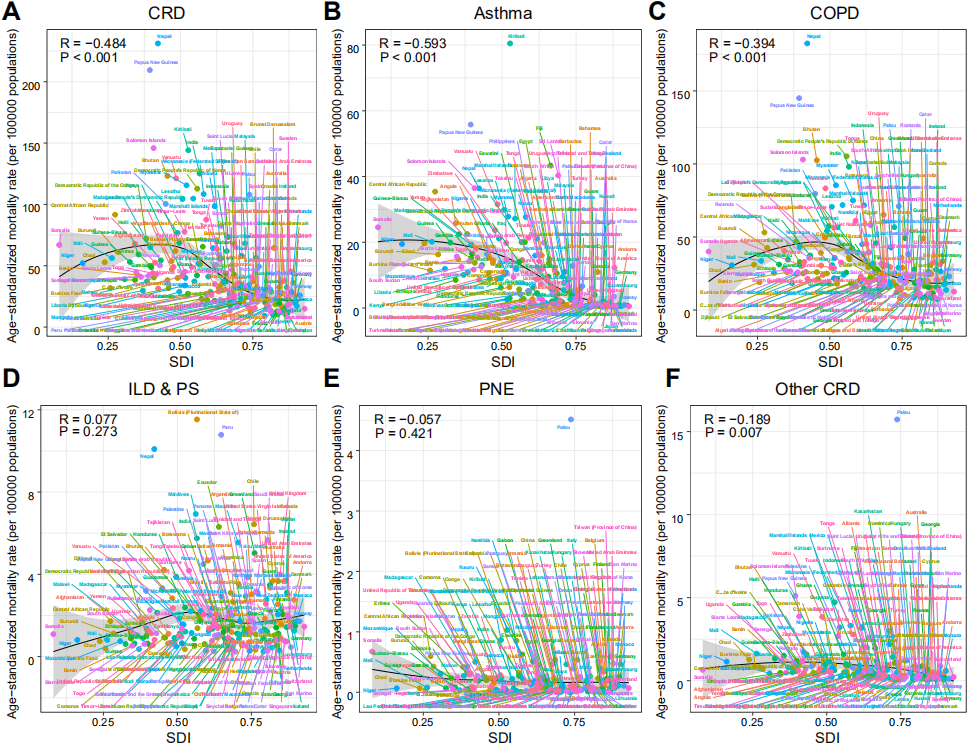


**Figure S10.** The correlation between the SDI and ASMR of overall and specific CRDs in 2019.

(A) CRDs, (B)asthma, (C) COPD, (D)ILD&PS, (E)PNE and (F) other CRDs. The colored dots and lines represent countries in each SDI quintile. SDI: Socio-demographic index; ASMR: age-standardized mortality rate; CRDs: chronic respiratory diseases; COPD: chronic obstructive pulmonary disease; ILD&PS: Interstitial lung disease and pulmonary sarcoidosis; PNE: pneumoconiosis.


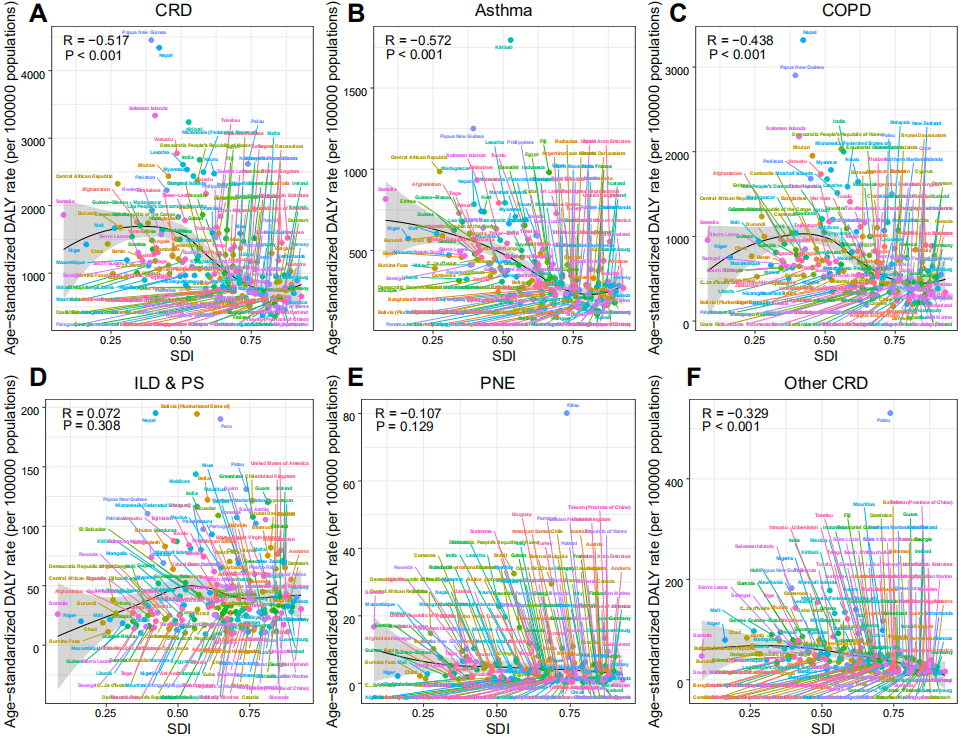


**Figure S11.** The correlation between the SDI and ASDR of overall and specific CRDs in 2019.

(A) CRDs, (B)asthma, (C) COPD, (D)ILD&PS, (E)PNE and (F) other CRDs. The colored dots and lines represent countries in each SDI quintile. SDI: Socio-demographic index; ASDR: age-standardized DALY rate; DALY: disability-adjusted life year; CRDs: chronic respiratory diseases; COPD: chronic obstructive pulmonary disease; ILD&PS: Interstitial lung disease and pulmonary sarcoidosis; PNE: pneumoconiosis.


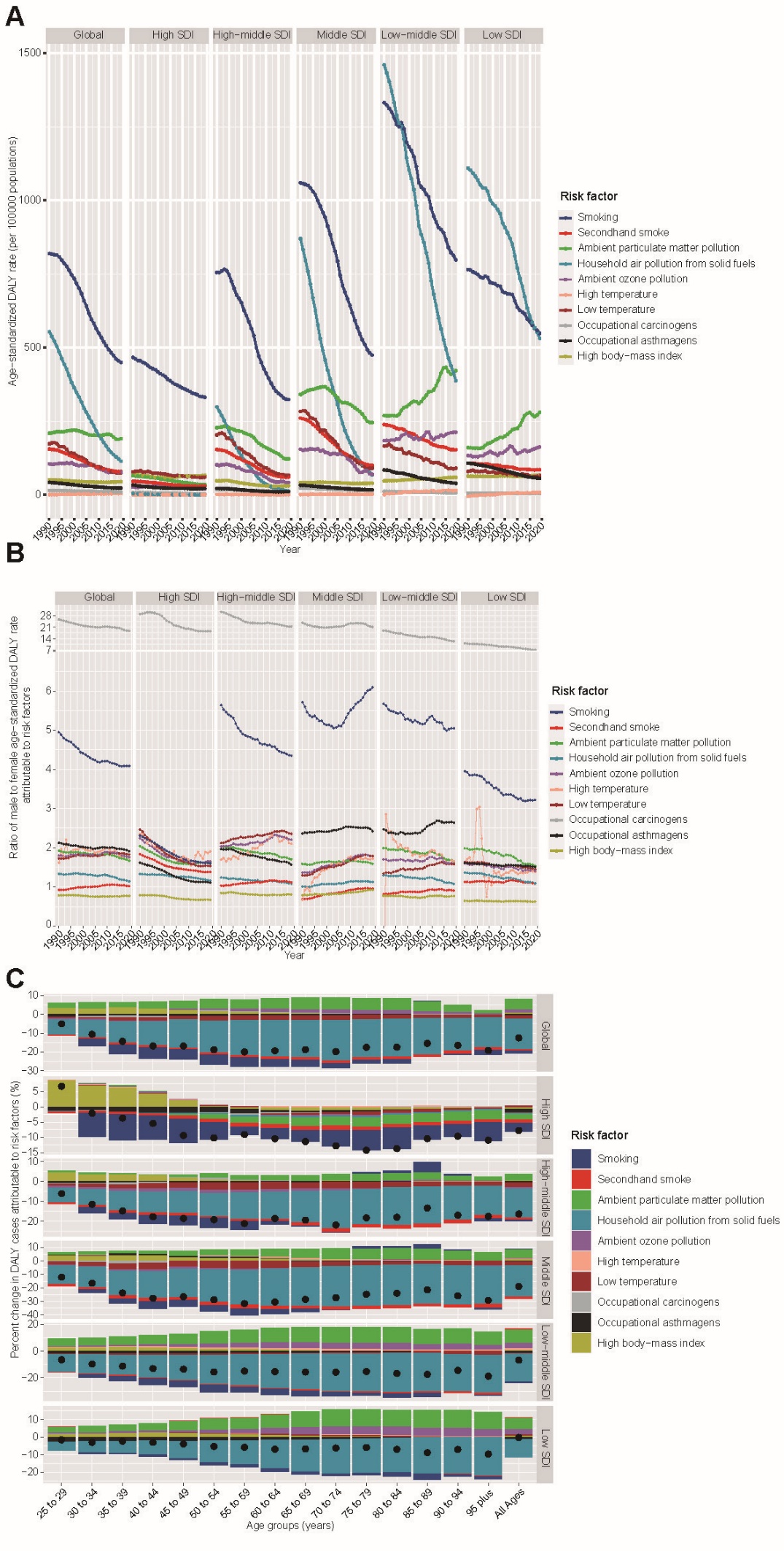


**Figure S12.** Prominent contribution of RFs to CRDs-related DALY by SDI, sex, and age groups

(A) The ASDR attributable to main RFs by SDI region(1990-2019).

(B) Male to female ratios of ASDR attributable to RFs (1990-2019).

(C) The percentage changes in DALY attributable to RFs by age group and SDI region (1990-2019).

The black dot represents the overall value of change

contributed by all RFs.

RF: risk factor; CRDs: chronic respiratory diseases; DALY: Disability adjusted life years; SDI: Socio-demographic index; ASDR: Age-standardized DALY rate.


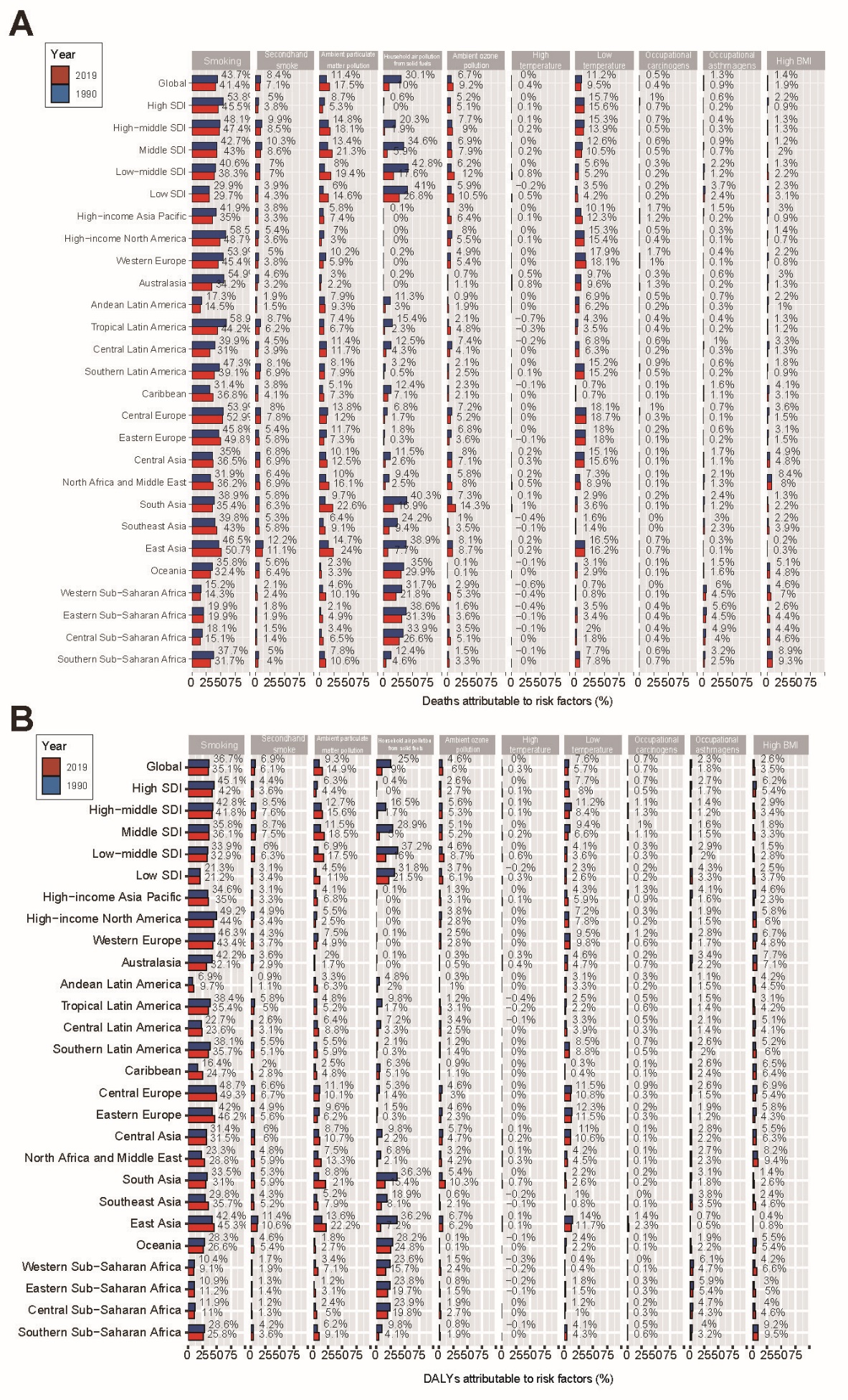


**Figure S13.** Percentage of CRDs mortality(A) and DALY(B) attributable to RFs for SDI and GBD regions (1990/2019); the red bar represents 2019 and the blue bar represents 1990. RF: risk factor; CRDs: chronic respiratory diseases; DALY: Disability adjusted life years; SDI: Socio-demographic index; GBD: Global Burden of Disease Study.
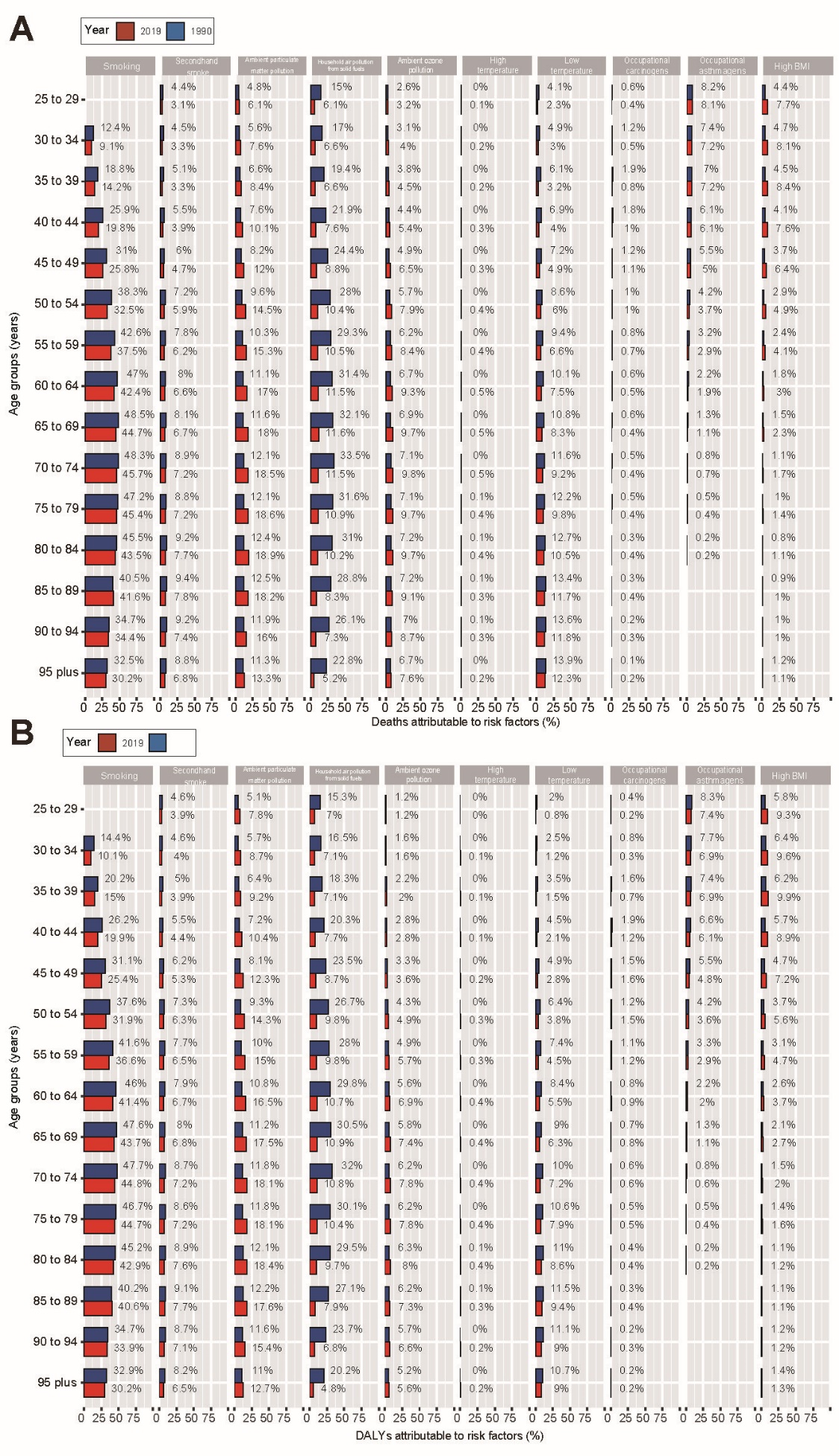


**Figure S14.** Percentage of mortality(A) and DALY(B) owing to CRDs attributable to RFs by age groups (1990/2019); the red bar represents 2019 and the blue bar represents 1990. RF: risk factor; CRDs: chronic respiratory diseases; DALY: Disability adjusted life years; SDI: Socio-demographic index; GBD: Global Burden of Disease Study.
