# Supplementary material for: Global, regional, and national burden of chronic respiratory diseases and associated risk factors, 1990–2019: Results from the Global Burden of Disease Study 2019
Source: Front Med (Lausanne). 2023 Mar 28;10:1066804. doi: 10.3389/fmed.2023.1066804 (PMC10088372; doi:10.3389/fmed.2023.1066804)
Supplement: Supplementary file 1 [file Data_Sheet_1.ZIP › Additional file 3.tables.docx]

Table S1. Prevalence of specific CRDs in 2019 for sexes, SDI and regions, with AAPC (1990-2019).

|  | CRDs | |  | COPD | |  | Asthma | |  | ILD&PS | |  | PNE | |
| --- | --- | --- | --- | --- | --- | --- | --- | --- | --- | --- | --- | --- | --- | --- |
| GBD data | Age-standardised prevalence per 100000 population (95% UI) | AAPC % (95% CI) 1990-2019 |  | Age-standardised prevalence per 100000 population (95% UI) | AAPC % (95% CI) 1990-2019 |  | Age-standardised prevalence per 100000 population (95% UI) | AAPC % (95% CI) 1990-2019 |  | Age-standardised prevalence per 100000 population (95% UI) | AAPC % (95% CI) 1990-2019 |  | Age-standardised prevalence per 100000 population (95% UI) | AAPC % (95% CI) 1990-2019 |
| Global | 5789.16 (5290.68 to 6418.14) | -0.64 (-0.7 to -0.58) |  | 2638.2 (2492.17 to 2796.14) | -0.34 (-0.4 to -0.28) |  | 3415.53 (2898.92 to 4066.2) | -0.91 (-1.04 to -0.79) |  | 57.62 (49.42 to 65.67) | 0.33 (0.25 to 0.4) |  | 36.78  (31.1 to 43.08) | -0.38 (-0.71 to -0.04) |
| **Sex** |  |  |  |  |  |  |  |  |  |  |  |  |  |  |
| Female | 5695.91 (5209.31 to 6282.15) | -0.67 (-0.73 to -0.62) |  | 2487.12 (2346.46 to 2640.57) | -0.26 (-0.33 to -0.19) |  | 3488.49 (2982.65 to 4112.59) | -0.98 (-1.09 to -0.88) |  | 56.45 (48.18 to 64.8) | 0.28 (0.19 to 0.37) |  | 5.28 (4.57 to 6.05) | -0.46 (-0.89 to -0.03) |
| Male | 5907.65 (5382.04 to 6581.16) | -0.63 (-0.71 to -0.55) |  | 2828.08 (2677.62 to 2990.53) | -0.46 (-0.52 to -0.41) |  | 3332.81 (2804.34 to 4028.84) | -0.86 (-1.02 to -0.69) |  | 59.67 (51.21 to 68.03) | 0.37 (0.29 to 0.45) |  | 71.36 (60.05 to 83.86) | -0.4 (-0.74 to -0.06) |
| **SDI** |  |  |  |  |  |  |  |  |  |  |  |  |  |  |
| High SDI | 9110.33 (8211.2 to 10240.43) | -0.49 (-0.59 to -0.4) |  | 2792.03 (2672.08 to 2914.75) | -0.17 (-0.2 to -0.14) |  | 6855.06 (5876.69 to 8058.48) | -0.7 (-0.84 to -0.56) |  | 101.21 (87.66 to 113.79) | 0.57 (0.49 to 0.65) |  | 11.02 (9.87 to 12.34) | -1.33 (-1.67 to -0.98) |
| High-middle SDI | 5296.89 (4736.23 to 6016.11) | -0.83 (-0.94 to -0.72) |  | 2370.37 (2218.01 to 2534.44) | -0.69 (-0.79 to -0.59) |  | 3103.02 (2541.3 to 3833.34) | -1.1 (-1.41 to -0.8) |  | 45.59 (38.85 to 52.37) | 0.32 (0.23 to 0.42) |  | 51.56 (43.47 to 60.99) | -0.33 (-0.68 to 0.02) |
| Middle SDI | 5264.17 (4747.44 to 5910.84) | -0.36 (-0.44 to -0.28) |  | 2512.83 (2336.82 to 2705.59) | -0.38 (-0.46 to -0.29) |  | 2927.13 (2429.34 to 3578.75) | -0.36 (-0.54 to -0.18) |  | 38.99 (32.77 to 45.41) | 0.66 (0.51 to 0.82) |  | 58.47 (48.81 to 69.34) | -0.56 (-0.96 to -0.16) |
| Low-middle SDI | 5417.1 (5005.46 to 5903.14) | -0.28 (-0.35 to -0.21) |  | 3005.2 (2849.42 to 3163.12) | -0.13 (-0.16 to -0.1) |  | 2713.25 (2317.27 to 3209.72) | -0.46 (-0.62 to -0.29) |  | 54.34 (45.83 to 63.34) | 0.1 (0.04 to 0.16) |  | 18.26 (15.59 to 21.45) | -0.24 (-0.39 to -0.09) |
| Low SDI | 5549.6 (5101.56 to 6120.66) | -0.27 (-0.31 to -0.23) |  | 2335.68 (2211.11 to 2474.39) | 0.05 (0.02 to 0.09) |  | 3468.63 (3015.27 to 4035.78) | -0.47 (-0.54 to -0.39) |  | 39.55 (33.5 to 46.06) | 0.16 (0.09 to 0.23) |  | 3.85 (3.32 to 4.49) | -0.1 (-0.23 to 0.02) |
| **Region** |  |  |  |  |  |  |  |  |  |  |  |  |  |  |
| High-income Asia Pacific | 5181.21 (4471.8 to 6125.97) | -1.81 (-1.96 to -1.67) |  | 1500.68 (1384.15 to 1621.26) | -0.78 (-0.89 to -0.67) |  | 3744.86 (3032.34 to 4726.2) | -2.46 (-2.69 to -2.23) |  | 147.91 (124.96 to 171.65) | 0.27 (0.19 to 0.36) |  | 11.63 (9.82 to 13.82) | -3.97 (-4.48 to -3.45) |
| High-income North America | 12449.57 (11351.77 to 13803.52) | 0.38 (0.09 to 0.67) |  | 3558.42 (3412.92 to 3694.43) | 0.02 (-0.02 to 0.07) |  | 9848.14 (8624.26 to 11312.08) | 0.59 (0.17 to 1.02) |  | 124.97 (109.31 to 140.35) | 0.22 (-0.3 to 0.74) |  | 8.34 (7.37 to 9.4) | -1.11 (-1.42 to -0.8) |
| Western Europe | 8449.61 (7477.19 to 9614.06) | -0.9 (-1.1 to -0.71) |  | 3017.46 (2874.68 to 3165.75) | 0 (-0.01 to 0.02) |  | 5893.41 (4900.26 to 7117.65) | -1.44 (-1.74 to -1.14) |  | 58.55 (50.53 to 66.9) | 0.92 (0.79 to 1.06) |  | 4.07 (3.56 to 4.69) | -3.79 (-4.17 to -3.41) |
| Australasia | 10936.44 (9497.47 to 12696.54) | -1.02 (-1.29 to -0.75) |  | 3192.77 (3027.74 to 3372.11) | -0.42 (-0.48 to -0.37) |  | 8393.25 (6908.82 to 10347.07) | -1.3 (-1.65 to -0.95) |  | 63.24 (55.07 to 71.19) | 1.33 (1.26 to 1.41) |  | 3.14 (2.59 to 4.06) | 0.21 (-0.23 to 0.65) |
| Andean Latin America | 5519.29 (4546.85 to 6761.35) | -0.59 (-0.72 to -0.46) |  | 1382.65 (1297.91 to 1464.47) | 0.55 (0.49 to 0.6) |  | 4215.37 (3152.42 to 5621.36) | -1 (-1.18 to -0.82) |  | 124.12 (111.75 to 135.46) | 1.92 (1.75 to 2.1) |  | 5.01 (4.04 to 6.16) | 1.18 (0.75 to 1.62) |
| Tropical Latin America | 7279.67 (6181.33 to 8633.32) | -0.69 (-0.77 to -0.6) |  | 2634.55 (2436.28 to 2860.76) | -0.63 (-0.71 to -0.56) |  | 4907.35 (3771.52 to 6383.32) | -0.74 (-0.91 to -0.57) |  | 20.66 (17.27 to 24.32) | -1.33 (-1.49 to -1.18) |  | 20.46 (17.22 to 24.76) | -0.35 (-0.82 to 0.11) |
| Central Latin America | 5309.65 (4606.64 to 6148.31) | -0.57 (-0.63 to -0.5) |  | 2186.52 (2052.61 to 2332.39) | 0.31 (0.24 to 0.38) |  | 3244.35 (2546.19 to 4160.4) | -1.1 (-1.22 to -0.98) |  | 47.66 (40.82 to 55.11) | 0.25 (0.23 to 0.26) |  | 12.31 (10.52 to 14.47) | -1.2 (-1.49 to -0.9) |
| Southern Latin America | 8716.12 (7771.03 to 10037.53) | 0.06 (-0.02 to 0.13) |  | 2741.66 (2619.33 to 2874.99) | 0.61 (0.57 to 0.66) |  | 6450.18 (5427.78 to 7800.39) | -0.15 (-0.25 to -0.04) |  | 97.12 (86.72 to 107.02) | 1.72 (1.62 to 1.82) |  | 7.31 (5.88 to 9.16) | 1.67 (1.3 to 2.04) |
| Caribbean | 7804.39 (6858.66 to 8982.39) | -0.23 (-0.27 to -0.18) |  | 2044.24 (1944.55 to 2141.04) | 0.88 (0.85 to 0.91) |  | 6072.63 (5048.46 to 7345.09) | -0.52 (-0.57 to -0.46) |  | 24.3 (20.98 to 28.2) | 0.84 (0.81 to 0.86) |  | 0.68 (0.55 to 0.86) | 0.29 (0.2 to 0.37) |
| Central Europe | 6405.17 (5664.26 to 7297.92) | -0.72 (-0.81 to -0.64) |  | 2427.44 (2316.89 to 2552.27) | -0.02 (-0.06 to 0.02) |  | 4203.57 (3452.76 to 5155.48) | -1.2 (-1.34 to -1.06) |  | 56 (48.28 to 65.05) | 0.2 (0.04 to 0.36) |  | 12.11 (10.35 to 14.12) | -0.85 (-1.01 to -0.69) |
| Eastern Europe | 4453.83 (3918.48 to 5167.66) | -1.59 (-1.66 to -1.51) |  | 1821.46 (1705.91 to 1949.5) | -1.25 (-1.38 to -1.12) |  | 2712.03 (2160.78 to 3412.64) | -1.94 (-2.1 to -1.78) |  | 39.9 (31.99 to 49.78) | -0.41 (-0.63 to -0.2) |  | 8.52 (7.08 to 10.32) | -0.22 (-0.41 to -0.04) |
| Central Asia | 4412.99 (4024.71 to 4927.71) | -0.53 (-0.58 to -0.47) |  | 2257.21 (2169.43 to 2349.15) | -0.5 (-0.67 to -0.34) |  | 2277.44 (1883.33 to 2787.8) | -0.57 (-0.73 to -0.41) |  | 37.62 (32.58 to 43.17) | -0.47 (-0.59 to -0.36) |  | 4.83 (3.92 to 5.99) | 1.31 (1.16 to 1.45) |
| North Africa and Middle East | 5891.24 (5320.98 to 6584.28) | 0.09 (0.05 to 0.12) |  | 2333.88 (2230.12 to 2443.59) | 0.91 (0.88 to 0.94) |  | 3819.33 (3262.52 to 4512.65) | -0.3 (-0.36 to -0.25) |  | 44.49 (38.2 to 51.28) | 1.28 (1.21 to 1.35) |  | 2.57 (2.12 to 3.08) | 1.49 (1.16 to 1.83) |
| South Asia | 5366.27 (4957.78 to 5815.77) | -0.24 (-0.42 to -0.07) |  | 3298.82 (3104.71 to 3490.79) | -0.19 (-0.2 to -0.18) |  | 2443.4 (2029.82 to 2909.76) | -0.3 (-0.7 to 0.1) |  | 69.31 (58.01 to 81.62) | -0.01 (-0.07 to 0.04) |  | 6.29 (5.33 to 7.55) | -0.39 (-0.5 to -0.29) |
| Southeast Asia | 5651.09 (5157.94 to 6262.32) | -0.13 (-0.21 to -0.06) |  | 2480.07 (2353.02 to 2624.53) | 0.09 (0.05 to 0.14) |  | 3431.82 (2926.72 to 4059.76) | -0.31 (-0.44 to -0.18) |  | 17.55 (14.54 to 20.64) | 0.95 (0.93 to 0.97) |  | 5.56 (4.35 to 6.99) | 1.05 (0.89 to 1.21) |
| East Asia | 4352.33 (3867.67 to 4978.69) | -0.72 (-0.89 to -0.55) |  | 2396.06 (2191.27 to 2620.06) | -1.14 (-1.28 to -1) |  | 2025.52 (1577.43 to 2631.41) | -0.31 (-0.9 to 0.28) |  | 35.35 (28.99 to 41.76) | 0.76 (0.42 to 1.1) |  | 120.41 (100.75 to 142.96) | -0.57 (-0.94 to -0.2) |
| Oceania | 6504.63 (6095.2 to 6939.03) | -0.52 (-0.55 to -0.48) |  | 2514.74 (2435.25 to 2581.71) | -0.03 (-0.04 to -0.02) |  | 4265.16 (3834.97 to 4731.89) | -0.83 (-0.89 to -0.77) |  | 85.25 (76.15 to 94.25) | 0.88 (0.84 to 0.92) |  | 6.68 (5.24 to 8.34) | 1.28 (1.17 to 1.38) |
| Western Sub-Saharan Africa | 4581.4 (4126.53 to 5168.81) | -0.57 (-0.7 to -0.43) |  | 1618.41 (1521.58 to 1721.28) | -0.2 (-0.25 to -0.15) |  | 3087.32 (2629.03 to 3669.71) | -0.78 (-0.96 to -0.59) |  | 22.07 (18.26 to 26.62) | -0.06 (-0.15 to 0.03) |  | 0.97 (0.82 to 1.14) | -0.85 (-0.91 to -0.78) |
| Eastern Sub-Saharan Africa | 5486.86 (4919.39 to 6242.77) | -0.57 (-0.62 to -0.52) |  | 1503.48 (1407.96 to 1608.75) | -0.18 (-0.28 to -0.07) |  | 4151.16 (3582.13 to 4898.7) | -0.71 (-0.77 to -0.66) |  | 22.65 (19.05 to 26.75) | 0.41 (0.34 to 0.48) |  | 3.21 (2.73 to 3.78) | -0.24 (-0.41 to -0.07) |
| Central Sub-Saharan Africa | 4876.92 (4434.13 to 5402.1) | -0.26 (-0.31 to -0.22) |  | 1943.19 (1816.65 to 2076.15) | 0.4 (0.27 to 0.53) |  | 3081.67 (2653.81 to 3633.35) | -0.62 (-0.67 to -0.57) |  | 27.58 (23.5 to 32.08) | 0.44 (0.37 to 0.51) |  | 4.01 (3.43 to 4.65) | 0.53 (0.24 to 0.83) |
| Southern Sub-Saharan Africa | 5523.82 (4620.04 to 6421.33) | -0.28 (-0.58 to 0.01) |  | 2243.92 (2090.05 to 2411.68) | -0.36 (-0.45 to -0.27) |  | 3476.21 (2532.37 to 4396.58) | -0.2 (-0.73 to 0.33) |  | 49.94 (42.07 to 58.86) | -0.36 (-0.41 to -0.31) |  | 18.99 (16.34 to 22.12) | 0.44 (0.18 to 0.7) |

CRDs: chronic respiratory diseases; COPD: chronic obstructive pulmonary disease; ILD&PS: Interstitial lung disease and pulmonary sarcoidosis; PNE: pneumoconiosis; AAPC: average annual percent change; UI: uncertainty interval; CI: confidence interval; SDI: Socio-demographic index.

Table S2. **Prevalence** of **CRDs** (1990/2019) for sexes, SDI and locations, with AAPC (1990-2019)

| GBD data | 1990 | |  | 2019 | | AAPC % (95% CI)  1990-2019 |
| --- | --- | --- | --- | --- | --- | --- |
|  | Cases (95% UI) | Age-standardised prevalence per  100 000 population (95% UI) |  | Cases (95% UI) | Age-standardised prevalence per  100 000 population (95% UI) |  |
| Global | 325227850 (294659801 to 363895648) | 6963.96 (6397.42 to 7654.04) |  | 454557390 (417354403 to 499144380) | 5789.16 (5290.68 to 6418.14) | -0.64 (-0.7 to -0.58) |
| **Sex** |  |  |  |  |  |  |
| Female | 165566199 (151088107 to 184322405) | 6899.4 (6348.35 to 7584.81) |  | 231152957 (212559495 to 252660333) | 5695.91 (5209.31 to 6282.15) | -0.67 (-0.73 to -0.62) |
| Male | 159661651 (143862173 to 180698632) | 7096.39 (6523.78 to 7817.54) |  | 223404433 (203679747 to 248407883) | 5907.65 (5382.04 to 6581.16) | -0.63 (-0.71 to -0.55) |
| **SDI** |  |  |  |  |  |  |
| High SDI | 93227759 (85561117 to 103117097) | 10448.79 (9478.81 to 11715.9) |  | 111890796 (103862564 to 120447230) | 9110.33 (8211.2 to 10240.43) | -0.49 (-0.59 to -0.4) |
| High-middle SDI | 74014630 (67188410 to 82435049) | 6746.54 (6145.74 to 7468.94) |  | 86144403 (78876826 to 94478706) | 5296.89 (4736.23 to 6016.11) | -0.83 (-0.94 to -0.72) |
| Middle SDI | 82061538 (72700687 to 94295218) | 5821.16 (5307.83 to 6448.51) |  | 123815506 (112658899 to 138000392) | 5264.17 (4747.44 to 5910.84) | -0.36 (-0.44 to -0.28) |
| Low-middle SDI | 49935035 (45144024 to 56347784) | 5914.94 (5489.04 to 6395.52) |  | 82481524 (75628261 to 91033633) | 5417.1 (5005.46 to 5903.14) | -0.28 (-0.35 to -0.21) |
| Low SDI | 25779187 (22704696 to 30113837) | 6020 (5549.69 to 6583.57) |  | 49928256 (43435735 to 58192038) | 5549.6 (5101.56 to 6120.66) | -0.27 (-0.31 to -0.23) |
| **Region** |  |  |  |  |  |  |
| High-income Asia Pacific | 15595177 (13981664 to 17496395) | 8553.03 (7678.66 to 9643.7) |  | 13524396 (12384865 to 14875991) | 5181.21 (4471.8 to 6125.97) | -1.81 (-1.96 to -1.67) |
| High-income North America | 34339356 (30978426 to 38645830) | 11647.38 (10322.05 to 13333.07) |  | 52638477 (49184741 to 56555381) | 12449.57 (11351.77 to 13803.52) | 0.38 (0.09 to 0.67) |
| Western Europe | 48279119 (44437628 to 52828895) | 10475.29 (9517.15 to 11734.07) |  | 49616742 (45577274 to 54278252) | 8449.61 (7477.19 to 9614.06) | -0.9 (-1.1 to -0.71) |
| Australasia | 2990208 (2826368 to 3167991) | 14487.09 (13684.54 to 15423.02) |  | 3596396 (3240398 to 4002542) | 10936.44 (9497.47 to 12696.54) | -1.02 (-1.29 to -0.75) |
| Andean Latin America | 2794666 (2264516 to 3442478) | 6388.86 (5364.58 to 7647.34) |  | 3434185 (2812614 to 4232116) | 5519.29 (4546.85 to 6761.35) | -0.59 (-0.72 to -0.46) |
| Tropical Latin America | 12533364 (10309643 to 15629192) | 8710.37 (7520.64 to 10279.63) |  | 15686977 (13554279 to 18289360) | 7279.67 (6181.33 to 8633.32) | -0.69 (-0.77 to -0.6) |
| Central Latin America | 9414720 (7887413 to 11339287) | 6181.41 (5467.2 to 7057.65) |  | 12802204 (11151611 to 14835688) | 5309.65 (4606.64 to 6148.31) | -0.57 (-0.63 to -0.5) |
| Southern Latin America | 4106470 (3681444 to 4634942) | 8516.14 (7663.85 to 9556.88) |  | 6289607 (5692879 to 7053892) | 8716.12 (7771.03 to 10037.53) | 0.06 (-0.02 to 0.13) |
| Caribbean | 2937317 (2546756 to 3416900) | 8331.63 (7374.79 to 9528.16) |  | 3643997 (3236341 to 4150963) | 7804.39 (6858.66 to 8982.39) | -0.23 (-0.27 to -0.18) |
| Central Europe | 10365365 (9352293 to 11670307) | 7765.73 (6952.84 to 8821.52) |  | 9408225 (8600853 to 10356267) | 6405.17 (5664.26 to 7297.92) | -0.72 (-0.81 to -0.64) |
| Eastern Europe | 17335641 (15622780 to 19194958) | 6990.22 (6251.95 to 7831.43) |  | 10862111 (9826188 to 11974988) | 4453.83 (3918.48 to 5167.66) | -1.59 (-1.66 to -1.51) |
| Central Asia | 2915248 (2651635 to 3266120) | 5084.32 (4710.99 to 5519.7) |  | 3671866 (3309535 to 4163424) | 4412.99 (4024.71 to 4927.71) | -0.53 (-0.58 to -0.47) |
| North Africa and Middle East | 16588154 (14452975 to 19230272) | 5723.13 (5213.35 to 6346.56) |  | 31776197 (28349827 to 36022813) | 5891.24 (5320.98 to 6584.28) | 0.09 (0.05 to 0.12) |
| South Asia | 42192173 (38510716 to 46845917) | 5809.11 (5407.55 to 6236.6) |  | 81003958 (74252202 to 88513792) | 5366.27 (4957.78 to 5815.77) | -0.24 (-0.42 to -0.07) |
| Southeast Asia | 22527769 (20043832 to 25889916) | 5847.77 (5367.71 to 6464.54) |  | 35926312 (32695055 to 39797804) | 5651.09 (5157.94 to 6262.32) | -0.13 (-0.21 to -0.06) |
| East Asia | 54752610 (49024630 to 61938128) | 5478.17 (4977.4 to 6076.07) |  | 72603642 (66122037 to 79486904) | 4352.33 (3867.67 to 4978.69) | -0.72 (-0.89 to -0.55) |
| Oceania | 381292 (347259 to 424961) | 7570.13 (7089.9 to 8107.33) |  | 699604 (640666 to 766013) | 6504.63 (6095.2 to 6939.03) | -0.52 (-0.55 to -0.48) |
| Western Sub-Saharan Africa | 8546433 (7383436 to 10131937) | 5295.28 (4787.76 to 5914.77) |  | 17677345 (15043894 to 21372019) | 4581.4 (4126.53 to 5168.81) | -0.57 (-0.7 to -0.43) |
| Eastern Sub-Saharan Africa | 11347410 (9764822 to 13579019) | 6471.92 (5866.94 to 7242.64) |  | 20536229 (17359042 to 24732550) | 5486.86 (4919.39 to 6242.77) | -0.57 (-0.62 to -0.52) |
| Central Sub-Saharan Africa | 2533341 (2165396 to 3002527) | 5238.02 (4770.82 to 5786.12) |  | 5276142 (4515014 to 6211222) | 4876.92 (4434.13 to 5402.1) | -0.26 (-0.31 to -0.22) |
| Southern Sub-Saharan Africa | 2752014 (2170059 to 3293643) | 6354.55 (5251.43 to 7283.57) |  | 3882778 (3173435 to 4588692) | 5523.82 (4620.04 to 6421.33) | -0.28 (-0.58 to 0.01) |
| **Countries** |  |  |  |  |  |  |
| Afghanistan | 613638 (543637 to 696040) | 6271.07 (5753.69 to 6836.34) |  | 1838723 (1573141 to 2197794) | 6187.2 (5648.37 to 6825.71) | -0.05 (-0.09 to -0.01) |
| Albania | 114846 (101318 to 134318) | 4217.39 (3837.03 to 4739.89) |  | 138322 (124894 to 153828) | 4318.57 (3802.98 to 4971.02) | 0.1 (-0.15 to 0.36) |
| Algeria | 970350 (833644 to 1159953) | 4503.65 (4056.66 to 5081.06) |  | 1900259 (1665739 to 2205755) | 4879.6 (4306.8 to 5592.56) | 0.31 (0.22 to 0.39) |
| American Samoa | 2029 (1702 to 2472) | 4996.21 (4325.88 to 5761.92) |  | 2423 (1877 to 2918) | 4604.76 (3588.7 to 5445.74) | -0.18 (-0.54 to 0.19) |
| Andorra | 5164 (4526 to 5892) | 9320.19 (8206.71 to 10555.81) |  | 8956 (8093 to 9956) | 8606.42 (7563.64 to 9814.83) | -0.28 (-0.31 to -0.24) |
| Angola | 546752 (475598 to 644008) | 6000.11 (5491.48 to 6613.94) |  | 1216879 (1019606 to 1451787) | 4768.85 (4299.47 to 5286.53) | -0.83 (-0.89 to -0.77) |
| Antigua and Barbuda | 3721 (3046 to 4613) | 5997.43 (4963.75 to 7355.74) |  | 5189 (4422 to 6155) | 6458.77 (5288.66 to 7846.12) | 0.3 (0.15 to 0.46) |
| Argentina | 2993724 (2667601 to 3377182) | 9122.76 (8138.36 to 10268) |  | 4519829 (4071061 to 5092282) | 9441.34 (8386.87 to 10805.9) | 0.08 (0.02 to 0.13) |
| Armenia | 136393 (123589 to 153116) | 4569.39 (4198.23 to 5035.71) |  | 151184 (135978 to 165956) | 4356.16 (3878.26 to 4904.05) | -0.11 (-0.23 to 0.01) |
| Australia | 2415482 (2301266 to 2541254) | 14152.84 (13404.71 to 14933.81) |  | 3105185 (2787897 to 3479005) | 11253.26 (9736.76 to 13143.42) | -0.83 (-1.11 to -0.55) |
| Austria | 861888 (777399 to 953271) | 9196.34 (8202.18 to 10391.56) |  | 956288 (876147 to 1054459) | 7971.71 (7046.84 to 9117.54) | -0.49 (-0.64 to -0.33) |
| Azerbaijan | 275538 (248543 to 311443) | 4461.04 (4113.1 to 4877.23) |  | 398582 (361487 to 441392) | 4199.04 (3814.09 to 4681.1) | -0.19 (-0.26 to -0.13) |
| Bahamas | 16528 (13741 to 19840) | 6473.89 (5510.19 to 7631.1) |  | 23202 (19866 to 27404) | 6648.75 (5578.25 to 8028.24) | 0.17 (0.06 to 0.28) |
| Bahrain | 24249 (21254 to 28095) | 6694.68 (6138.23 to 7383.39) |  | 65325 (57398 to 75096) | 5462.05 (4803.12 to 6235.58) | -0.66 (-0.79 to -0.53) |
| Bangladesh | 3028511 (2835030 to 3263392) | 4710.89 (4478.99 to 4952.63) |  | 5812846 (5477418 to 6153203) | 4202.52 (3974.73 to 4431.01) | -0.41 (-0.45 to -0.37) |
| Barbados | 18690 (16253 to 21744) | 7375.35 (6376.77 to 8668.52) |  | 21636 (18873 to 24807) | 7643.94 (6368.05 to 9144.7) | 0.09 (-0.01 to 0.2) |
| Belarus | 913843 (821882 to 1011266) | 7898.55 (7036.71 to 8855.18) |  | 568925 (513274 to 637054) | 5071.11 (4443.03 to 5911.34) | -1.59 (-1.67 to -1.5) |
| Belgium | 1218111 (1118685 to 1338370) | 9845.08 (8916.07 to 11076.36) |  | 1292283 (1207267 to 1391697) | 8071.58 (7258.35 to 9018.35) | -0.92 (-1.16 to -0.69) |
| Belize | 14569 (12097 to 17932) | 7224.4 (6287.5 to 8429.14) |  | 29175 (24539 to 34675) | 7283.49 (6242.69 to 8610.32) | -0.03 (-0.23 to 0.16) |
| Benin | 191632 (166339 to 224790) | 4992.03 (4602.98 to 5470.18) |  | 458166 (390826 to 547580) | 4577.02 (4179.89 to 5085.51) | -0.34 (-0.44 to -0.25) |
| Bermuda | 3618 (3101 to 4243) | 6545.41 (5477.26 to 7844.71) |  | 4140 (3662 to 4716) | 6956.68 (5711.17 to 8430.68) | 0.28 (0.19 to 0.37) |
| Bhutan | 18155 (16843 to 19647) | 5082 (4825.89 to 5335.27) |  | 27006 (25500 to 28611) | 4417.22 (4179.07 to 4658.13) | -0.54 (-0.62 to -0.47) |
| Bolivia (Plurinational State of) | 498145 (403680 to 611574) | 6805.38 (5787.67 to 8011.62) |  | 708048 (579436 to 865706) | 5973.71 (5033.38 to 7169.16) | -0.45 (-0.7 to -0.19) |
| Bosnia and Herzegovina | 285746 (252266 to 326881) | 6619.12 (5908.28 to 7522.33) |  | 269265 (243210 to 300772) | 6449.23 (5670.66 to 7466.51) | -0.11 (-0.19 to -0.04) |
| Botswana | 42670 (37942 to 48530) | 4603.55 (4278.05 to 4981.16) |  | 88589 (78641 to 100624) | 4688.98 (4270.64 to 5173.54) | -0.02 (-0.12 to 0.07) |
| Brazil | 12282905 (10101934 to 15312492) | 8788.41 (7585.79 to 10371.87) |  | 15225302 (13164259 to 17721696) | 7285.62 (6172.03 to 8643.95) | -0.72 (-0.81 to -0.63) |
| Brunei Darussalam | 13961 (12024 to 16670) | 8305.65 (7534.97 to 9355.01) |  | 24832 (22016 to 28895) | 7234.56 (6489.23 to 8161.19) | -0.52 (-0.57 to -0.47) |
| Bulgaria | 648021 (588008 to 721506) | 6421.45 (5748.59 to 7224.62) |  | 571762 (524260 to 622523) | 6042.61 (5362.42 to 6817.85) | -0.24 (-0.32 to -0.16) |
| Burkina Faso | 332087 (279790 to 401074) | 3861.68 (3486.91 to 4307.84) |  | 782799 (659328 to 959128) | 3957 (3566.98 to 4468.64) | 0.11 (-0.01 to 0.22) |
| Burundi | 386999 (334182 to 450346) | 7623.88 (6927.99 to 8370.72) |  | 607918 (513647 to 726812) | 5721.97 (5160.47 to 6357.97) | -1.02 (-1.14 to -0.91) |
| Cabo Verde | 14462 (12470 to 17066) | 4638.45 (4192.13 to 5172.87) |  | 20452 (17970 to 23816) | 3876.62 (3451.42 to 4446.2) | -0.61 (-0.64 to -0.59) |
| Cambodia | 315476 (273303 to 372245) | 3827.98 (3523.28 to 4188.51) |  | 639096 (574223 to 725459) | 4449.51 (4074.76 to 4933.31) | 0.54 (0.42 to 0.66) |
| Cameroon | 375100 (326731 to 440390) | 4796.36 (4412.95 to 5284.85) |  | 993034 (850267 to 1186086) | 4410.53 (4010.16 to 4914.37) | -0.31 (-0.47 to -0.15) |
| Canada | 2148690 (1921460 to 2445514) | 7589.5 (6678.79 to 8735.51) |  | 3310838 (3022305 to 3634133) | 7199.42 (6329.21 to 8328.5) | -0.04 (-0.31 to 0.24) |
| Central African Republic | 142288 (124764 to 163675) | 6121.86 (5624.54 to 6687.48) |  | 230785 (201815 to 266824) | 5239.59 (4797.5 to 5738.22) | -0.53 (-0.55 to -0.51) |
| Chad | 189322 (164136 to 223208) | 3874.66 (3558.57 to 4268.88) |  | 491906 (412501 to 598915) | 3758.21 (3418.39 to 4171) | -0.15 (-0.23 to -0.07) |
| Chile | 819004 (728115 to 945142) | 6923.11 (6260.8 to 7839.01) |  | 1424931 (1288202 to 1586154) | 7048.61 (6233.24 to 8057.35) | 0.04 (-0.11 to 0.19) |
| China | 52390890 (46868679 to 59414716) | 5436.5 (4929.73 to 6037.71) |  | 69467916 (63164092 to 76091527) | 4314.55 (3833.19 to 4932.81) | -0.71 (-0.88 to -0.54) |
| Colombia | 1980045 (1651634 to 2372698) | 6805.58 (5971.74 to 7796.95) |  | 2788325 (2469400 to 3184962) | 5781.09 (5041.8 to 6690.35) | -0.5 (-0.71 to -0.29) |
| Comoros | 26823 (23100 to 31674) | 6143.64 (5545.63 to 6895.48) |  | 36392 (31752 to 42313) | 5391.81 (4817.37 to 6129.84) | -0.44 (-0.48 to -0.41) |
| Congo | 129688 (112531 to 152112) | 6312.5 (5758.68 to 7027.5) |  | 248793 (214402 to 292188) | 5591.77 (5041.51 to 6257.58) | -0.41 (-0.57 to -0.25) |
| Cook Islands | 1145 (968 to 1349) | 7028.24 (6010.03 to 7983.29) |  | 1357 (1155 to 1502) | 6617.45 (5620.65 to 7518.75) | -0.12 (-0.23 to -0.01) |
| Costa Rica | 249287 (209060 to 297155) | 8651.02 (7571.34 to 10039.5) |  | 343246 (299575 to 397840) | 7394.18 (6373.18 to 8684.55) | -0.57 (-0.67 to -0.47) |
| Croatia | 397781 (350706 to 452670) | 7169.67 (6289.79 to 8204.71) |  | 382594 (353007 to 420099) | 6387.84 (5690.92 to 7279.19) | -0.47 (-0.58 to -0.35) |
| Cuba | 959573 (821440 to 1120226) | 9041.96 (7776.73 to 10676.45) |  | 1081427 (974990 to 1209397) | 9203.59 (7831.66 to 10865.2) | 0.08 (0.05 to 0.11) |
| Cyprus | 76882 (66256 to 89012) | 9735.75 (8382.93 to 11283.16) |  | 141606 (124393 to 161102) | 9323.95 (8061.54 to 10757.96) | -0.14 (-0.18 to -0.1) |
| Czechia | 582813 (531112 to 651885) | 5128 (4602.22 to 5844.2) |  | 789379 (729487 to 858555) | 5475.75 (4896.06 to 6208.45) | 0.21 (0.1 to 0.33) |
| Côte d’Ivoire | 487402 (419351 to 578530) | 5078.05 (4635.01 to 5636.55) |  | 1033030 (890225 to 1221181) | 4722.94 (4266.8 to 5284.65) | -0.2 (-0.3 to -0.11) |
| Democratic People's Republic of Korea | 1317956 (1190579 to 1501193) | 7401.6 (6787.52 to 8331.4) |  | 2029244 (1912115 to 2164843) | 6981.96 (6486.48 to 7650.27) | -0.22 (-0.34 to -0.09) |
| Democratic Republic of the Congo | 1650765 (1397967 to 1976318) | 4904.74 (4434.07 to 5429.82) |  | 3459256 (2975995 to 4088962) | 4851.43 (4419.85 to 5397.81) | -0.03 (-0.09 to 0.03) |
| Denmark | 607921 (563502 to 658344) | 9486.76 (8683.67 to 10505.66) |  | 697396 (652171 to 749471) | 8530.19 (7678.93 to 9543.84) | -0.4 (-0.58 to -0.22) |
| Djibouti | 28825 (24468 to 34339) | 6438 (5791.07 to 7211.46) |  | 58913 (50655 to 69482) | 5441.1 (4821.6 to 6193.78) | -0.54 (-0.62 to -0.47) |
| Dominica | 5703 (4879 to 6746) | 7499.1 (6522.67 to 8745.83) |  | 5309 (4751 to 6001) | 7917.71 (6922.93 to 9188.96) | 0.22 (0.16 to 0.28) |
| Dominican Republic | 364518 (305939 to 435843) | 4758.01 (4121.47 to 5507.77) |  | 536784 (457799 to 636207) | 5054.06 (4334.62 to 5983.85) | 0.25 (0.03 to 0.48) |
| Ecuador | 687727 (543258 to 858482) | 6066.46 (5015.99 to 7333.08) |  | 901984 (734423 to 1126682) | 5298.4 (4352.9 to 6561.04) | -0.46 (-0.6 to -0.32) |
| Egypt | 2553451 (2206835 to 2984221) | 5237.32 (4695.1 to 5866.69) |  | 5077154 (4475511 to 5799595) | 5983.21 (5399.43 to 6671.01) | 0.47 (0.42 to 0.52) |
| El Salvador | 533161 (423750 to 656902) | 9410.29 (7747.62 to 11174.07) |  | 425362 (363354 to 505386) | 6835.11 (5857.01 to 8051.9) | -1.15 (-1.29 to -1) |
| Equatorial Guinea | 20875 (18071 to 24404) | 5529.42 (5043.45 to 6064.45) |  | 54221 (44755 to 66349) | 4743.91 (4244.92 to 5341.47) | -0.49 (-0.6 to -0.39) |
| Eritrea | 182239 (157614 to 213704) | 6898.78 (6280.24 to 7575.28) |  | 352251 (305422 to 411608) | 6027.6 (5482.74 to 6713.81) | -0.49 (-0.55 to -0.43) |
| Estonia | 71402 (65938 to 77316) | 4152.91 (3781.83 to 4561.46) |  | 57299 (52707 to 62418) | 3429.92 (3017.72 to 3934.08) | -0.67 (-0.79 to -0.56) |
| Eswatini | 42680 (37350 to 49312) | 7370.95 (6766.51 to 7975.77) |  | 61021 (54848 to 68590) | 6798.14 (6266.11 to 7353.29) | -0.35 (-0.43 to -0.27) |
| Ethiopia | 2354810 (1974987 to 2849809) | 5258.13 (4705.82 to 5953.28) |  | 3863517 (3168317 to 4785790) | 3894.17 (3407.77 to 4508.19) | -1.03 (-1.09 to -0.96) |
| Fiji | 34516 (30271 to 39439) | 5548.43 (4989.05 to 6176.75) |  | 33500 (29503 to 38169) | 3897.58 (3462.6 to 4415.04) | -1.22 (-1.3 to -1.14) |
| Finland | 484987 (436143 to 540117) | 8499.97 (7520.64 to 9603.39) |  | 590317 (535092 to 649840) | 7949.58 (6949.99 to 9155.97) | -0.26 (-0.4 to -0.11) |
| France | 7536435 (6913321 to 8325862) | 11229.59 (10193.96 to 12669.65) |  | 6773522 (6096360 to 7551936) | 8183.61 (7040.54 to 9500.91) | -1.37 (-1.6 to -1.15) |
| Gabon | 42972 (37120 to 50274) | 4957.58 (4473.75 to 5543.95) |  | 66209 (56852 to 78666) | 4275.82 (3804.05 to 4899.47) | -0.51 (-0.58 to -0.45) |
| Gambia | 37561 (32186 to 44399) | 4963.85 (4560.92 to 5481.95) |  | 83343 (71583 to 98465) | 4655.25 (4250.93 to 5123.95) | -0.26 (-0.32 to -0.2) |
| Georgia | 202806 (186862 to 224930) | 3565.84 (3272.6 to 3964.84) |  | 196346 (182141 to 212668) | 4226.92 (3815.1 to 4743.8) | 0.64 (0.55 to 0.73) |
| Germany | 9364773 (8571940 to 10248024) | 9266.98 (8429.54 to 10325.35) |  | 9104744 (8495336 to 9763704) | 7229.33 (6490.57 to 8130.55) | -0.91 (-1.24 to -0.57) |
| Ghana | 428365 (364658 to 507473) | 3388.52 (3077.3 to 3746.13) |  | 1004340 (878481 to 1185778) | 3775.01 (3437.01 to 4231.9) | 0.37 (0.33 to 0.4) |
| Greece | 974695 (867878 to 1102645) | 7876.96 (6912.69 to 9112.21) |  | 1158107 (1056172 to 1280049) | 7728.99 (6762.59 to 8927.41) | -0.06 (-0.11 to -0.01) |
| Greenland | 5754 (4857 to 6597) | 12507.71 (10706.96 to 14166.06) |  | 5522 (5091 to 6053) | 9412.76 (8507.94 to 10584.01) | -1.04 (-1.13 to -0.96) |
| Grenada | 7250 (5985 to 8833) | 8085.25 (6830.88 to 9596.74) |  | 8142 (6929 to 9677) | 8272.73 (6945.4 to 10011.04) | 0.06 (0.03 to 0.09) |
| Guam | 6200 (4901 to 7611) | 5222.6 (4147.57 to 6174.57) |  | 7495 (5761 to 8993) | 4377.81 (3407.7 to 5285.89) | -0.38 (-0.65 to -0.12) |
| Guatemala | 557282 (463987 to 672175) | 6022.78 (5241.79 to 6943.03) |  | 692031 (565195 to 846221) | 4029.72 (3432.22 to 4792.99) | -1.46 (-1.57 to -1.35) |
| Guinea | 282926 (243825 to 333615) | 5267.04 (4740.98 to 5889.88) |  | 544268 (464319 to 653522) | 5193.14 (4689.97 to 5816.05) | -0.13 (-0.25 to 0) |
| Guinea-Bissau | 45030 (39600 to 51835) | 5760.29 (5337.08 to 6280.19) |  | 74463 (64976 to 87111) | 5004.64 (4614.55 to 5506.68) | -0.55 (-0.65 to -0.46) |
| Guyana | 58068 (49067 to 69462) | 7466.52 (6569.09 to 8638.51) |  | 55223 (47231 to 64772) | 7276.77 (6267.4 to 8488.7) | -0.13 (-0.21 to -0.06) |
| Haiti | 637290 (545714 to 748530) | 9209.4 (8198.54 to 10425.36) |  | 1044343 (907757 to 1213420) | 8533.3 (7574.18 to 9668.54) | -0.28 (-0.32 to -0.24) |
| Honduras | 368373 (306659 to 444756) | 7005.79 (6153.59 to 8046.83) |  | 540076 (454314 to 649833) | 5898.01 (5156.37 to 6840.12) | -0.62 (-0.71 to -0.53) |
| Hungary | 810025 (747650 to 879708) | 6711.91 (6092.57 to 7433.14) |  | 887652 (833867 to 949239) | 6517.54 (5916.87 to 7302.37) | -0.12 (-0.22 to -0.03) |
| Iceland | 30477 (26935 to 34631) | 11653.66 (10263.36 to 13190.25) |  | 38801 (34722 to 43767) | 9581.09 (8339.82 to 11116.77) | -0.79 (-1.08 to -0.49) |
| India | 35162150 (31915595 to 39112467) | 6174.23 (5734.03 to 6658.12) |  | 68032773 (61964258 to 74601617) | 5654.87 (5180.05 to 6155.12) | -0.26 (-0.49 to -0.03) |
| Indonesia | 8938238 (7876436 to 10342889) | 5907.17 (5357.82 to 6577.18) |  | 13362704 (12086913 to 14894510) | 5558.37 (5043.43 to 6182.65) | -0.31 (-0.44 to -0.19) |
| Iran (Islamic Republic of) | 2737620 (2317641 to 3270976) | 5670.25 (5033.62 to 6394.6) |  | 4076810 (3634959 to 4620503) | 5155.4 (4567.16 to 5859.61) | -0.38 (-0.47 to -0.3) |
| Iraq | 937415 (808783 to 1123407) | 6283.04 (5687.27 to 7012.08) |  | 1733352 (1468258 to 2097993) | 4426.29 (3873.24 to 5168.34) | -1.22 (-1.26 to -1.18) |
| Ireland | 495448 (448642 to 548406) | 13167.04 (11891.48 to 14626.05) |  | 569208 (517054 to 629831) | 9905.8 (8804.17 to 11317.11) | -1.04 (-1.41 to -0.67) |
| Israel | 415288 (367668 to 470621) | 8575.41 (7594.6 to 9653.58) |  | 687888 (613391 to 778410) | 6935.48 (6117.88 to 7941.77) | -0.8 (-0.9 to -0.69) |
| Italy | 5703200 (5146015 to 6322793) | 7725.13 (6897.99 to 8723.69) |  | 5497358 (4973307 to 6064704) | 6096.56 (5266.61 to 7094.58) | -0.76 (-1.11 to -0.41) |
| Jamaica | 196449 (166795 to 235381) | 7991.96 (6975.63 to 9370.85) |  | 195364 (167757 to 229607) | 7263.4 (6117.93 to 8628.52) | -0.41 (-0.6 to -0.23) |
| Japan | 13219128 (11866600 to 14778370) | 9392.1 (8320.74 to 10712.68) |  | 9764702 (8928522 to 10761853) | 5186.63 (4440.17 to 6205.62) | -2.16 (-2.31 to -2.02) |
| Jordan | 183230 (150300 to 225092) | 5861.24 (5170.87 to 6691.65) |  | 579726 (489172 to 702294) | 5523.98 (4789.04 to 6462.37) | -0.18 (-0.24 to -0.12) |
| Kazakhstan | 629661 (584062 to 683060) | 4495.7 (4223.91 to 4808.41) |  | 788932 (732370 to 857950) | 4493.26 (4180.49 to 4862.99) | 0 (-0.06 to 0.06) |
| Kenya | 939159 (779311 to 1151922) | 4807.71 (4278.89 to 5430.78) |  | 1871391 (1587144 to 2234348) | 4355.17 (3873.16 to 4952.45) | -0.32 (-0.35 to -0.28) |
| Kiribati | 4892 (4501 to 5334) | 7482.35 (6987.01 to 7990.18) |  | 5950 (5487 to 6521) | 5637.68 (5267.53 to 6061.78) | -0.96 (-0.99 to -0.93) |
| Kuwait | 83505 (69384 to 100409) | 5355.53 (4646.93 to 6201.05) |  | 209896 (174191 to 254890) | 5116.28 (4278.99 to 6137.9) | -0.2 (-0.32 to -0.09) |
| Kyrgyzstan | 243863 (221447 to 273789) | 6761.99 (6264.71 to 7360.49) |  | 286383 (253882 to 327952) | 5177.02 (4689.48 to 5748.15) | -0.96 (-1.05 to -0.86) |
| Lao People's Democratic Republic | 153339 (136738 to 175615) | 4561.91 (4219.69 to 4964.12) |  | 269773 (246910 to 297242) | 4698.27 (4377.12 to 5068.02) | 0.04 (-0.04 to 0.12) |
| Latvia | 188847 (170722 to 209221) | 6330.54 (5704.17 to 7120.48) |  | 102393 (92940 to 114293) | 4421.24 (3817.55 to 5235.77) | -1.22 (-1.29 to -1.16) |
| Lebanon | 183462 (159818 to 212853) | 6195.7 (5550.06 to 6968.89) |  | 352118 (313588 to 399702) | 6840.3 (6054.29 to 7766.49) | 0.33 (0.3 to 0.37) |
| Lesotho | 49112 (44961 to 53796) | 3764.02 (3547.3 to 4003.09) |  | 64558 (60129 to 69369) | 4066.12 (3829.5 to 4312.77) | 0.26 (0.23 to 0.29) |
| Liberia | 74306 (63899 to 89007) | 3916.73 (3565.49 to 4394.02) |  | 155554 (132050 to 187738) | 3772.36 (3391.64 to 4271.51) | -0.13 (-0.27 to 0.01) |
| Libya | 202103 (171610 to 243517) | 5780.86 (5226.49 to 6492.39) |  | 364029 (326892 to 410352) | 5961.85 (5335.92 to 6756.29) | 0.02 (-0.12 to 0.16) |
| Lithuania | 227356 (208736 to 246239) | 5592.18 (5090.04 to 6161.1) |  | 147594 (134846 to 162120) | 4194.64 (3675.56 to 4834.73) | -1 (-1.13 to -0.86) |
| Luxembourg | 51154 (45723 to 60039) | 11628.06 (10215.36 to 13555.73) |  | 71771 (64769 to 79269) | 9523.99 (8374.44 to 10840.66) | -0.7 (-0.81 to -0.58) |
| Madagascar | 1369912 (1208408 to 1574648) | 11301.55 (10259.5 to 12491.22) |  | 1973064 (1745919 to 2247304) | 8192.17 (7494.85 to 8985.72) | -1.28 (-1.45 to -1.1) |
| Malawi | 471682 (395024 to 569309) | 5287.81 (4729.05 to 5983.26) |  | 847687 (699494 to 1028566) | 5069.26 (4480.38 to 5820.16) | -0.07 (-0.2 to 0.05) |
| Malaysia | 715448 (645834 to 808815) | 5270.44 (4897.12 to 5721) |  | 1308482 (1172107 to 1490477) | 4547.87 (4071.48 to 5193.16) | -0.62 (-0.72 to -0.52) |
| Maldives | 11988 (10622 to 13721) | 6825.5 (6380.26 to 7348.61) |  | 20938 (18752 to 23797) | 5428.13 (4905.9 to 6056.38) | -0.89 (-0.97 to -0.81) |
| Mali | 278824 (244317 to 324390) | 4144.81 (3824.26 to 4530.19) |  | 714227 (621998 to 851797) | 4347.95 (3999.07 to 4789.65) | 0.19 (0.05 to 0.33) |
| Malta | 42693 (38191 to 48120) | 10742.18 (9593.04 to 12150.45) |  | 51640 (46484 to 57969) | 8951.79 (7779.49 to 10400.44) | -0.69 (-0.75 to -0.62) |
| Marshall Islands | 1997 (1748 to 2340) | 5663.71 (5273.92 to 6155.61) |  | 2247 (1946 to 2621) | 4512.52 (3988.02 to 5163.64) | -0.68 (-0.84 to -0.51) |
| Mauritania | 123361 (104268 to 145591) | 7191.25 (6363.41 to 8083.79) |  | 216413 (184367 to 259383) | 6174.23 (5538.62 to 6990.43) | -0.56 (-0.61 to -0.51) |
| Mauritius | 57811 (52399 to 64574) | 6194.37 (5712.7 to 6808.53) |  | 64907 (60489 to 70625) | 4669.31 (4232.41 to 5270.19) | -1.06 (-1.14 to -0.98) |
| Mexico | 4128102 (3448805 to 5053896) | 5566.8 (4931.66 to 6370.39) |  | 5855499 (5074779 to 6877642) | 4918.75 (4250.97 to 5793.91) | -0.47 (-0.6 to -0.35) |
| Micronesia (Federated States of) | 4648 (4133 to 5320) | 5596 (5204.87 to 6062.06) |  | 4177 (3621 to 4863) | 4623.91 (4096.88 to 5296.36) | -0.59 (-0.78 to -0.41) |
| Monaco | 3813 (3478 to 4190) | 8839.61 (7799.56 to 10098.75) |  | 4806 (4427 to 5246) | 8646.11 (7618.93 to 9826.25) | -0.05 (-0.09 to -0.01) |
| Mongolia | 66777 (58270 to 78148) | 4129.11 (3775.63 to 4531.3) |  | 99094 (87057 to 115004) | 3331.34 (2978.47 to 3764.9) | -0.7 (-0.84 to -0.55) |
| Montenegro | 27301 (23566 to 32364) | 4385.52 (3794.69 to 5208.37) |  | 33061 (28692 to 38185) | 4783.34 (4073.44 to 5659) | 0.36 (0.25 to 0.48) |
| Morocco | 874867 (759825 to 1011896) | 3960.57 (3582.28 to 4402.18) |  | 1595027 (1428951 to 1804287) | 4685.51 (4213.64 to 5282.06) | 0.58 (0.55 to 0.6) |
| Mozambique | 700141 (597823 to 836112) | 5401.7 (4875.46 to 6075.21) |  | 1526587 (1277781 to 1853253) | 5703.67 (5127.72 to 6431.41) | 0.18 (0.17 to 0.2) |
| Myanmar | 1605918 (1484324 to 1756972) | 5384.46 (5075.53 to 5701.6) |  | 2708393 (2569011 to 2862584) | 5601.8 (5329.8 to 5892.29) | 0.1 (0.03 to 0.17) |
| Namibia | 49036 (44186 to 55107) | 4696.36 (4389.37 to 5081.13) |  | 79002 (70760 to 89374) | 4157.44 (3849.53 to 4528.28) | -0.46 (-0.51 to -0.42) |
| Nauru | 468 (410 to 555) | 6386.02 (5917.26 to 7035.15) |  | 472 (393 to 578) | 6099.59 (5346.41 to 6990.21) | -0.07 (-0.22 to 0.08) |
| Nepal | 509513 (480773 to 541488) | 4453.05 (4234.21 to 4671.79) |  | 1045656 (994253 to 1100068) | 4457.14 (4255.21 to 4666.82) | 0.01 (-0.04 to 0.05) |
| Netherlands | 1837423 (1599174 to 2098844) | 10803.99 (9244.47 to 12546.57) |  | 2372614 (2171038 to 2610071) | 10073.88 (8887.96 to 11406.31) | -0.2 (-0.35 to -0.05) |
| New Zealand | 574727 (502101 to 660781) | 16228.77 (14077.75 to 18785.66) |  | 491210 (445804 to 546808) | 9185.56 (8054.85 to 10628.6) | -2 (-2.13 to -1.88) |
| Nicaragua | 298311 (237726 to 368080) | 6700.42 (5734.63 to 7834.84) |  | 329496 (275324 to 397510) | 5430.13 (4657.83 to 6371.29) | -0.74 (-0.85 to -0.63) |
| Niger | 330020 (283204 to 393396) | 4911.43 (4497.22 to 5438.12) |  | 818783 (683751 to 999451) | 4321.81 (3934.72 to 4828.08) | -0.5 (-0.59 to -0.4) |
| Nigeria | 4773788 (4094409 to 5711078) | 6152.48 (5461.3 to 6962.67) |  | 9107672 (7604862 to 11241524) | 4847.84 (4301.2 to 5570.91) | -0.94 (-1.15 to -0.73) |
| Niue | 149 (130 to 172) | 6418.04 (5676.45 to 7311.68) |  | 119 (100 to 133) | 6401.57 (5367.87 to 7303.41) | 0.08 (-0.08 to 0.24) |
| North Macedonia | 160712 (134860 to 188194) | 8326.45 (6982.43 to 9737.25) |  | 171489 (154537 to 191332) | 6820.77 (6022.41 to 7763.58) | -0.74 (-0.86 to -0.63) |
| Northern Mariana Islands | 1979 (1629 to 2405) | 5530.37 (4672.47 to 6464.29) |  | 1882 (1418 to 2220) | 4684 (3763.37 to 5628.23) | -0.49 (-0.79 to -0.18) |
| Norway | 669791 (602916 to 745952) | 12861.1 (11400.84 to 14541.33) |  | 666686 (604066 to 740039) | 9898.33 (8700.66 to 11300.44) | -0.91 (-1.06 to -0.75) |
| Oman | 68394 (56039 to 85600) | 3898.12 (3330.42 to 4632.6) |  | 201603 (167505 to 245822) | 5206.54 (4447.54 to 6159.62) | 1.09 (0.94 to 1.25) |
| Pakistan | 3473843 (3149403 to 3873952) | 4562.13 (4221.99 to 4933.01) |  | 6085677 (5506999 to 6726139) | 4115.27 (3814.08 to 4461.85) | -0.36 (-0.47 to -0.25) |
| Palau | 909 (809 to 1025) | 7109.83 (6431.88 to 7827.69) |  | 1178 (1065 to 1278) | 6294.4 (5709.82 to 6976.68) | -0.35 (-0.44 to -0.25) |
| Palestine | 88567 (73870 to 108847) | 5253.19 (4697.38 to 5939.36) |  | 209626 (173655 to 256627) | 4826.47 (4214.91 to 5597.55) | -0.31 (-0.38 to -0.25) |
| Panama | 161434 (137918 to 190843) | 6686.52 (5859.77 to 7780.95) |  | 243996 (207804 to 289519) | 5882.81 (4991.77 to 7007.69) | -0.46 (-0.53 to -0.39) |
| Papua New Guinea | 268251 (244542 to 296387) | 8627.91 (8107.24 to 9189.16) |  | 553985 (509090 to 603791) | 7330.19 (6897.06 to 7771.52) | -0.58 (-0.61 to -0.55) |
| Paraguay | 250460 (204774 to 311861) | 5941.88 (5142.62 to 7003.71) |  | 461675 (385047 to 565129) | 6904.84 (5827.6 to 8350.85) | 0.5 (0.46 to 0.54) |
| Peru | 1608794 (1296288 to 1983266) | 6440.25 (5377.47 to 7728.89) |  | 1824153 (1485934 to 2262887) | 5534.51 (4478.89 to 6891.01) | -0.58 (-0.65 to -0.52) |
| Philippines | 4481879 (3807242 to 5433310) | 7577.95 (6744.79 to 8734.55) |  | 6895160 (6080864 to 7970472) | 6704.55 (6024.76 to 7613.29) | -0.43 (-0.49 to -0.38) |
| Poland | 4603335 (4042281 to 5274156) | 11249.95 (9851.92 to 12970) |  | 3477351 (3114293 to 3901427) | 7339.44 (6362.42 to 8525.05) | -1.57 (-1.66 to -1.47) |
| Portugal | 1494331 (1315900 to 1711181) | 12953.41 (11244.98 to 15071.15) |  | 1579044 (1416698 to 1761149) | 11045.47 (9576.66 to 12854.9) | -0.92 (-1.33 to -0.52) |
| Puerto Rico | 434476 (376693 to 498268) | 12090.82 (10481.16 to 13894.43) |  | 367886 (331042 to 414390) | 10234.12 (8572.54 to 12124.38) | -0.62 (-0.77 to -0.47) |
| Qatar | 17809 (15250 to 21098) | 5214.05 (4638.83 to 5905.42) |  | 109487 (92111 to 132846) | 5056.7 (4392.22 to 5876.59) | -0.09 (-0.14 to -0.04) |
| Republic of Korea | 2125934 (1881638 to 2458568) | 5921.03 (5325.63 to 6617.75) |  | 3426022 (3100206 to 3805666) | 5408.67 (4763.02 to 6283.3) | -0.33 (-0.53 to -0.13) |
| Republic of Moldova | 280290 (257106 to 310084) | 6343.38 (5825.74 to 7023.72) |  | 197839 (177840 to 219188) | 4597.23 (4046.4 to 5324.78) | -1.14 (-1.2 to -1.08) |
| Romania | 1781029 (1586494 to 1996966) | 7082.78 (6259.96 to 8058.4) |  | 1558437 (1413589 to 1721835) | 6322.43 (5548.58 to 7257.77) | -0.39 (-0.46 to -0.32) |
| Russian Federation | 11030338 (9871557 to 12323904) | 6729.41 (5985.5 to 7567.17) |  | 7290000 (6611072 to 8062459) | 4271.77 (3765.79 to 4919.08) | -1.6 (-1.68 to -1.52) |
| Rwanda | 887030 (754508 to 1038173) | 13116.47 (11595.95 to 14671.91) |  | 1100689 (943557 to 1309986) | 9525.28 (8484.34 to 10852.92) | -1.11 (-1.34 to -0.89) |
| Saint Kitts and Nevis | 3074 (2565 to 3711) | 7313.75 (6250.78 to 8639.61) |  | 4004 (3459 to 4653) | 7251.74 (6075.17 to 8637.87) | -0.03 (-0.09 to 0.03) |
| Saint Lucia | 11710 (9781 to 14101) | 8497.95 (7372.93 to 9856.74) |  | 14162 (12696 to 15868) | 8391.17 (7304.02 to 9732.62) | -0.06 (-0.13 to 0) |
| Saint Vincent and the Grenadines | 7603 (6223 to 9316) | 6552.93 (5574.65 to 7788.14) |  | 7154 (6171 to 8439) | 6635.32 (5600.1 to 8021.45) | 0.08 (-0.02 to 0.18) |
| Samoa | 6969 (6035 to 8168) | 5116.07 (4611.54 to 5667.58) |  | 8015 (6797 to 9504) | 4158.59 (3629.73 to 4777.64) | -0.64 (-0.73 to -0.54) |
| San Marino | 2316 (2079 to 2575) | 8630.93 (7667.1 to 9822.01) |  | 3648 (3313 to 4037) | 8443.35 (7456.68 to 9734.85) | -0.05 (-0.08 to -0.03) |
| Sao Tome and Principe | 7483 (6516 to 8545) | 7186.34 (6619.63 to 7838.02) |  | 11497 (10379 to 13037) | 7207.57 (6724.93 to 7792.84) | -0.07 (-0.14 to 0) |
| Saudi Arabia | 441145 (370587 to 538305) | 3444 (3102.28 to 3897.38) |  | 1281110 (1133545 to 1454706) | 4632.67 (4148.42 to 5247.1) | 0.93 (0.77 to 1.09) |
| Senegal | 251275 (218490 to 297236) | 4148.91 (3808.86 to 4558.38) |  | 499548 (431723 to 588052) | 3981.45 (3617.56 to 4441.93) | -0.16 (-0.21 to -0.1) |
| Serbia | 537533 (495705 to 588546) | 5282.88 (4830.84 to 5876.4) |  | 646150 (604128 to 695451) | 5524.42 (5034.3 to 6135.18) | 0.17 (0.09 to 0.25) |
| Seychelles | 2814 (2516 to 3211) | 4224.01 (3844.3 to 4705.01) |  | 4626 (4257 to 5106) | 4499.28 (4070.74 to 5065.49) | 0.24 (0.19 to 0.29) |
| Sierra Leone | 151671 (133222 to 176130) | 4736.98 (4358.16 to 5209.49) |  | 309017 (268123 to 364004) | 4518.64 (4141.3 to 4998.09) | -0.2 (-0.34 to -0.07) |
| Singapore | 236154 (206555 to 265917) | 8875.8 (7963.64 to 9817.24) |  | 308841 (275045 to 349140) | 5084.21 (4423.13 to 5968.4) | -1.92 (-2.04 to -1.8) |
| Slovakia | 245722 (219479 to 279450) | 4493.82 (3982.07 to 5123.12) |  | 309999 (281127 to 342849) | 4698.9 (4154.78 to 5354.96) | 0.14 (0.06 to 0.23) |
| Slovenia | 170501 (152203 to 193674) | 7848.89 (6970.48 to 8940.86) |  | 172765 (155054 to 192373) | 6286.3 (5453.86 to 7318.99) | -0.76 (-0.85 to -0.68) |
| Solomon Islands | 14319 (12372 to 16819) | 5394.89 (4952.91 to 5957) |  | 26580 (22564 to 32258) | 4833.75 (4251.93 to 5611.99) | -0.27 (-0.46 to -0.07) |
| Somalia | 470451 (405990 to 556016) | 7159.61 (6527.9 to 7914.69) |  | 1039772 (885161 to 1244289) | 5799.23 (5263.41 to 6503.5) | -0.71 (-0.74 to -0.67) |
| South Africa | 2302291 (1732164 to 2782333) | 7238.55 (5714.04 to 8450.78) |  | 3163955 (2469768 to 3804610) | 6154.01 (4869.23 to 7367.72) | -0.29 (-0.67 to 0.09) |
| South Sudan | 335658 (287493 to 398283) | 6258.93 (5650.54 to 6988.91) |  | 457823 (384592 to 551917) | 5379.52 (4815.08 to 6065) | -0.47 (-0.53 to -0.41) |
| Spain | 3445965 (3163330 to 3820702) | 7487.63 (6771.16 to 8507.93) |  | 4791673 (4446201 to 5219359) | 7208.95 (6417.54 to 8184.05) | -0.19 (-0.26 to -0.12) |
| Sri Lanka | 741571 (676070 to 823963) | 5281.22 (4879.63 to 5726.29) |  | 1281126 (1186836 to 1392201) | 5513.1 (5056.57 to 6072.27) | 0.08 (0.01 to 0.14) |
| Sudan | 995290 (835481 to 1183724) | 5721.78 (5089.69 to 6479.41) |  | 2005577 (1689368 to 2429859) | 5845.47 (5174.41 to 6673.39) | 0.03 (-0.02 to 0.08) |
| Suriname | 26142 (22128 to 31220) | 6658.45 (5788.38 to 7738.28) |  | 37298 (32148 to 44184) | 6648.7 (5661.12 to 7921.79) | -0.01 (-0.09 to 0.08) |
| Sweden | 1417462 (1257254 to 1600771) | 13019.95 (11395.81 to 14976.86) |  | 1356262 (1211951 to 1527875) | 10136.19 (8677.23 to 11885.75) | -0.87 (-1.05 to -0.7) |
| Switzerland | 851583 (769106 to 952412) | 10504.17 (9289.8 to 11995.46) |  | 970855 (876306 to 1086663) | 8734.69 (7566.63 to 10075.04) | -0.63 (-0.68 to -0.58) |
| Syrian Arab Republic | 528958 (456032 to 622779) | 4875.18 (4446.68 to 5397.69) |  | 702544 (626729 to 790442) | 5136.93 (4638.7 to 5744.44) | 0.14 (0.01 to 0.26) |
| Taiwan (Province of China) | 1043764 (922241 to 1221808) | 5797.77 (5166.73 to 6705.17) |  | 1106482 (992378 to 1242213) | 4368.24 (3727.14 to 5248.2) | -1.31 (-1.65 to -0.96) |
| Tajikistan | 192262 (169172 to 224664) | 4789.2 (4404.19 to 5246.66) |  | 304980 (264389 to 359089) | 3985.15 (3576.61 to 4494.75) | -0.65 (-0.7 to -0.6) |
| Thailand | 2920668 (2651739 to 3247712) | 6258.99 (5785.44 to 6799.95) |  | 4051736 (3731730 to 4426815) | 5192.58 (4631 to 5915.24) | -0.83 (-1.03 to -0.62) |
| Timor-Leste | 32652 (28330 to 37737) | 4900.01 (4486.89 to 5366.95) |  | 58720 (52645 to 66054) | 5185.22 (4803.84 to 5621.46) | 0.2 (0.17 to 0.24) |
| Togo | 171530 (144504 to 206015) | 5954.49 (5363.69 to 6630.53) |  | 358586 (307990 to 426197) | 5411.64 (4865.53 to 6079.03) | -0.3 (-0.41 to -0.2) |
| Tokelau | 97 (83 to 118) | 6235.23 (5454.38 to 7338.77) |  | 87 (74 to 101) | 6338.95 (5340.69 to 7179.46) | 0.16 (0 to 0.32) |
| Tonga | 4898 (4141 to 5971) | 5765.59 (5129.96 to 6602.77) |  | 4669 (3926 to 5613) | 4741.74 (4089.32 to 5593.26) | -0.56 (-0.76 to -0.35) |
| Trinidad and Tobago | 64314 (54316 to 77015) | 5301.82 (4591.64 to 6205.17) |  | 73349 (62250 to 86325) | 5657.91 (4656.27 to 6895.44) | 0.37 (0.15 to 0.6) |
| Tunisia | 363835 (316542 to 430592) | 4980.95 (4488.32 to 5634.87) |  | 639006 (573348 to 717371) | 5477.15 (4869.78 to 6227.66) | 0.34 (0.3 to 0.39) |
| Turkey | 3875810 (3446651 to 4489886) | 7652.85 (7011.34 to 8498.45) |  | 6532177 (5975498 to 7185918) | 7856.17 (7167.31 to 8742.04) | 0.14 (0.03 to 0.26) |
| Turkmenistan | 151612 (134854 to 173522) | 5311.38 (4920.52 to 5773.33) |  | 156804 (133970 to 184852) | 3297.51 (2839.46 to 3828.59) | -1.72 (-1.82 to -1.61) |
| Tuvalu | 473 (431 to 528) | 5869.03 (5446.27 to 6424.65) |  | 661 (544 to 764) | 6031.27 (5006.11 to 6877.85) | 0.12 (-0.03 to 0.27) |
| Uganda | 1152968 (967992 to 1402201) | 7108.74 (6388.6 to 8058.98) |  | 2371576 (1947711 to 2942364) | 6294.06 (5580.96 to 7182.52) | -0.45 (-0.5 to -0.4) |
| Ukraine | 4623565 (4195012 to 5108837) | 7802.77 (6963.97 to 8795.73) |  | 2498061 (2237945 to 2803899) | 5003.02 (4305.17 to 5828.69) | -1.56 (-1.65 to -1.46) |
| United Arab Emirates | 141443 (121882 to 164194) | 9769.22 (8659.84 to 10878.08) |  | 774386 (680957 to 872724) | 9499.63 (8528.51 to 10587.98) | -0.33 (-0.76 to 0.11) |
| United Kingdom | 10647235 (9611573 to 11795879) | 16103.28 (14316.48 to 18190.08) |  | 10188015 (9254912 to 11185536) | 12151.98 (10750.52 to 13846.11) | -1.11 (-1.38 to -0.84) |
| United Republic of Tanzania | 1777737 (1469511 to 2167870) | 7166.71 (6294.81 to 8208.29) |  | 3810840 (3142421 to 4646752) | 6985.09 (6124.28 to 8110.18) | -0.05 (-0.28 to 0.18) |
| United States of America | 32184128 (29006940 to 36375344) | 12083.96 (10706.68 to 13846.29) |  | 49321281 (46068479 to 52947606) | 13030.37 (11908.32 to 14412.04) | 0.46 (0.15 to 0.77) |
| United States Virgin Islands | 6173 (5174 to 7430) | 5917.19 (5003.86 to 7066.52) |  | 6769 (6011 to 7727) | 6553.69 (5532.55 to 7824.1) | 0.43 (0.34 to 0.51) |
| Uruguay | 293576 (269994 to 321114) | 8647.15 (7886.21 to 9578.36) |  | 344532 (318402 to 376311) | 8381.55 (7542.44 to 9439.3) | -0.13 (-0.2 to -0.06) |
| Uzbekistan | 1016336 (902586 to 1153537) | 6457.16 (5892.52 to 7070.18) |  | 1289560 (1125948 to 1502375) | 4512.6 (4042.86 to 5101.01) | -1.32 (-1.42 to -1.21) |
| Vanuatu | 6254 (5511 to 7203) | 5241.3 (4849.26 to 5730.16) |  | 11763 (9419 to 14647) | 4568.97 (3750.59 to 5482.51) | -0.18 (-0.54 to 0.19) |
| Venezuela (Bolivarian Republic of) | 1138725 (942702 to 1390154) | 5999.74 (5177.08 to 7076.39) |  | 1584173 (1378475 to 1834547) | 5748.71 (4940.63 to 6727.53) | -0.21 (-0.31 to -0.11) |
| Viet Nam | 2520020 (2210994 to 2939130) | 4481.21 (4091.83 to 4962.64) |  | 5213582 (4785689 to 5726561) | 5648.23 (5165.76 to 6256) | 0.85 (0.66 to 1.03) |
| Yemen | 691857 (596286 to 822295) | 6307.68 (5779.99 to 6936.59) |  | 1141827 (981907 to 1311933) | 4760.94 (4202.96 to 5400.36) | -0.27 (-0.29 to -0.25) |
| Zambia | 254650 (217259 to 305304) | 3788.98 (3447.3 to 4202.51) |  | 561305 (483920 to 649563) | 4385.96 (3850.75 to 5006.52) | 0.1 (0.02 to 0.18) |
| Zimbabwe | 266225 (232233 to 310140) | 3762.11 (3485.16 to 4061.43) |  | 498291 (432239 to 574055) | 4396.02 (3861.56 to 5017.81) | 0.09 (0 to 0.17) |

CRDs: chronic respiratory diseases; AAPC: average annual percent change; UI: uncertainty interval; CI: confidence interval; SDI: Socio-demographic index.

Table S3. Prevalence of COPD (1990/2019) for sexes, SDI and locations, with AAPC (1990-2019)

| GBD data | 1990 | | |  | 2019 | | AAPC % (95% CI)  1990-2019 |
| --- | --- | --- | --- | --- | --- | --- | --- |
|  | Cases (95% UI) | | Age-standardised prevalence per  100 000 population (95% UI) |  | Cases (95% UI) | Age-standardised prevalence per  100 000 population (95% UI) |  |
| Global | 114923955 (109391037 to 120683217) | | 2890.89 (2743.25 to 3038.09) |  | 212335951 (200422146 to 225097834) | 2638.2 (2492.17 to 2796.14) | -0.34 (-0.4 to -0.28) |
| **Sex** |  |  | |  |  |  |  |
| Female | 57065974 (54161776 to 60097238) | 2657.03 (2520.95 to 2799.22) | |  | 107368252 (101311402 to 114029088) | 2487.12 (2346.46 to 2640.57) | -0.26 (-0.33 to -0.19) |
| Male | 57857981 (55086510 to 60743544) | 3211.1 (3052.53 to 3367.17) | |  | 104967700 (99208838 to 111045776) | 2828.08 (2677.62 to 2990.53) | -0.46 (-0.52 to -0.41) |
| **SDI** |  |  | |  |  |  |  |
| High SDI | 29836140 (28175995 to 31400705) | 2921.88 (2761.72 to 3071.97) | |  | 51288313 (49059906 to 53482844) | 2792.03 (2672.08 to 2914.75) | -0.17 (-0.2 to -0.14) |
| High-middle SDI | 29873546 (28324543 to 31532285) | 2858.35 (2710.08 to 3012) | |  | 46333398 (43250474 to 49744661) | 2370.37 (2218.01 to 2534.44) | -0.69 (-0.79 to -0.59) |
| Middle SDI | 29688384 (28089875 to 31416821) | 2774.4 (2613.46 to 2942.53) | |  | 60052410 (55803448 to 64515117) | 2512.83 (2336.82 to 2705.59) | -0.38 (-0.46 to -0.29) |
| Low-middle SDI | 19377033 (18599556 to 20109101) | 3095.75 (2954.85 to 3226.64) | |  | 40979413 (38960697 to 43097266) | 3005.2 (2849.42 to 3163.12) | -0.13 (-0.16 to -0.1) |
| Low SDI | 6103387 (5836002 to 6395817) | 2288.88 (2174.17 to 2409.19) | |  | 13581297 (12914964 to 14336674) | 2335.68 (2211.11 to 2474.39) | 0.05 (0.02 to 0.09) |
| **Region** |  |  | |  |  |  |  |
| High-income Asia Pacific | 3599283 (3292235 to 3929485) | 1887.83 (1734.36 to 2059.42) | |  | 6581036 (6084156 to 7107505) | 1500.68 (1384.15 to 1621.26) | -0.78 (-0.89 to -0.67) |
| High-income North America | 12245378 (11349362 to 12977981) | 3520.96 (3283.32 to 3725.25) | |  | 21968671 (20985083 to 22806701) | 3558.42 (3412.92 to 3694.43) | 0.02 (-0.02 to 0.07) |
| Western Europe | 16577913 (15847798 to 17358907) | 3002.66 (2872.86 to 3137.45) | |  | 26320873 (25114808 to 27583392) | 3017.46 (2874.68 to 3165.75) | 0 (-0.01 to 0.02) |
| Australasia | 818140 (783973 to 850593) | 3552.06 (3403.38 to 3696.85) | |  | 1540426 (1462887 to 1624938) | 3192.77 (3027.74 to 3372.11) | -0.42 (-0.48 to -0.37) |
| Andean Latin America | 300258 (283720 to 314305) | 1174.21 (1113.88 to 1232.68) | |  | 801856 (750171 to 851835) | 1382.65 (1297.91 to 1464.47) | 0.55 (0.49 to 0.6) |
| Tropical Latin America | 2969035 (2774856 to 3168873) | 3116.01 (2895.13 to 3344.67) | |  | 6330068 (5844669 to 6860751) | 2634.55 (2436.28 to 2860.76) | -0.63 (-0.71 to -0.56) |
| Central Latin America | 1857119 (1739756 to 1969452) | 1983.24 (1863.85 to 2118.53) | |  | 5157823 (4847688 to 5502692) | 2186.52 (2052.61 to 2332.39) | 0.31 (0.24 to 0.38) |
| Southern Latin America | 1047057 (1005814 to 1090801) | 2288.8 (2199.45 to 2384.48) | |  | 2234237 (2134786 to 2344295) | 2741.66 (2619.33 to 2874.99) | 0.61 (0.57 to 0.66) |
| Caribbean | 437591 (415043 to 459808) | 1582.46 (1508.24 to 1660.94) | |  | 1050382 (999066 to 1099680) | 2044.24 (1944.55 to 2141.04) | 0.88 (0.85 to 0.91) |
| Central Europe | 3429031 (3277526 to 3587713) | 2436.3 (2332.66 to 2548.08) | |  | 4764340 (4537311 to 5018946) | 2427.44 (2316.89 to 2552.27) | -0.02 (-0.06 to 0.02) |
| Eastern Europe | 6832230 (6412131 to 7283476) | 2585.16 (2432.25 to 2750.29) | |  | 5716966 (5330259 to 6145576) | 1821.46 (1705.91 to 1949.5) | -1.25 (-1.38 to -1.12) |
| Central Asia | 1275115 (1240112 to 1313192) | 2579.57 (2503.81 to 2661.45) | |  | 1677622 (1613858 to 1745238) | 2257.21 (2169.43 to 2349.15) | -0.5 (-0.67 to -0.34) |
| North Africa and Middle East | 3584089 (3404769 to 3768097) | 1787.6 (1690.86 to 1887.45) | |  | 10720230 (10262918 to 11204177) | 2333.88 (2230.12 to 2443.59) | 0.91 (0.88 to 0.94) |
| South Asia | 20038950 (19121289 to 20871763) | 3472.33 (3298.73 to 3631.4) | |  | 45677426 (43105372 to 48374684) | 3298.82 (3104.71 to 3490.79) | -0.19 (-0.2 to -0.18) |
| Southeast Asia | 6597328 (6264624 to 6996183) | 2387.19 (2243.08 to 2552.14) | |  | 14999748 (14232397 to 15835709) | 2480.07 (2353.02 to 2624.53) | 0.09 (0.05 to 0.14) |
| East Asia | 28661117 (27060997 to 30313430) | 3288.79 (3098.92 to 3477.4) | |  | 46713574 (42642934 to 51188724) | 2396.06 (2191.27 to 2620.06) | -1.14 (-1.28 to -1) |
| Oceania | 80256 (78523 to 81849) | 2528.72 (2463.57 to 2589.26) | |  | 188439 (183116 to 192867) | 2514.74 (2435.25 to 2581.71) | -0.03 (-0.04 to -0.02) |
| Western Sub-Saharan Africa | 1808146 (1713379 to 1917468) | 1695.3 (1588.35 to 1813.52) | |  | 3991683 (3783437 to 4209533) | 1618.41 (1521.58 to 1721.28) | -0.2 (-0.25 to -0.15) |
| Eastern Sub-Saharan Africa | 1524659 (1435212 to 1621299) | 1576.83 (1473.43 to 1690.6) | |  | 3292819 (3078465 to 3515414) | 1503.48 (1407.96 to 1608.75) | -0.18 (-0.28 to -0.07) |
| Central Sub-Saharan Africa | 482472 (451454 to 514449) | 1730.86 (1619.29 to 1860.31) | |  | 1270256 (1186242 to 1352608) | 1943.19 (1816.65 to 2076.15) | 0.4 (0.27 to 0.53) |
| Southern Sub-Saharan Africa | 758790 (713993 to 806720) | 2483.95 (2321.08 to 2658.27) | |  | 1337479 (1250978 to 1433369) | 2243.92 (2090.05 to 2411.68) | -0.36 (-0.45 to -0.27) |
| **Countries** |  |  | |  |  |  |  |
| Afghanistan | 134721 (124810 to 145783) | 1764 (1635.32 to 1913.33) | |  | 371175 (349917 to 393665) | 2141.35 (1998 to 2291.37) | 0.65 (0.62 to 0.68) |
| Albania | 37993 (35414 to 40630) | 1749.24 (1647.08 to 1855.52) | |  | 62519 (58363 to 67006) | 1597.03 (1487.95 to 1713.74) | -0.37 (-0.47 to -0.28) |
| Algeria | 210235 (196977 to 222964) | 1442.2 (1355.94 to 1534.24) | |  | 628753 (590277 to 667208) | 1787.76 (1681.96 to 1899.76) | 0.76 (0.71 to 0.81) |
| American Samoa | 414 (392 to 435) | 1667.25 (1581.23 to 1770.03) | |  | 617 (583 to 651) | 1374.16 (1299.67 to 1454.45) | -0.64 (-0.71 to -0.57) |
| Andorra | 1632 (1525 to 1738) | 3043.14 (2856.75 to 3225.53) | |  | 4441 (4180 to 4721) | 3217.02 (3020.71 to 3423.47) | 0.18 (0.15 to 0.21) |
| Angola | 87871 (82624 to 93253) | 1725.61 (1615.56 to 1847.57) | |  | 254081 (236617 to 271195) | 1757.57 (1644.56 to 1870.57) | 0.04 (-0.06 to 0.13) |
| Antigua and Barbuda | 466 (424 to 507) | 841.21 (769.64 to 912.4) | |  | 1052 (965 to 1146) | 1057.16 (969.95 to 1148.13) | 0.76 (0.7 to 0.82) |
| Argentina | 760655 (729290 to 792614) | 2400.53 (2301.2 to 2497.96) | |  | 1569490 (1498398 to 1649152) | 2973.1 (2836.92 to 3123.52) | 0.73 (0.7 to 0.76) |
| Armenia | 74339 (71481 to 77080) | 2753.83 (2642.45 to 2865.33) | |  | 93518 (89021 to 98603) | 2388 (2273.67 to 2515.37) | -0.51 (-0.61 to -0.41) |
| Australia | 676694 (651315 to 703043) | 3527.11 (3387.36 to 3667.57) | |  | 1284616 (1218823 to 1357765) | 3157.32 (2989.94 to 3336.45) | -0.44 (-0.51 to -0.38) |
| Austria | 308266 (294613 to 323825) | 2773.29 (2656.49 to 2901.42) | |  | 524635 (499937 to 553157) | 3116.44 (2966.1 to 3288.31) | 0.31 (0.17 to 0.44) |
| Azerbaijan | 134730 (129181 to 139871) | 2477.8 (2367.11 to 2579.9) | |  | 216758 (203899 to 230539) | 2357.9 (2223.99 to 2504.48) | -0.16 (-0.23 to -0.08) |
| Bahamas | 2031 (1846 to 2210) | 1076.06 (996.71 to 1155.74) | |  | 4772 (4432 to 5132) | 1194.72 (1113.2 to 1281.71) | 0.38 (0.33 to 0.42) |
| Bahrain | 5335 (4908 to 5750) | 2584.52 (2430.19 to 2743.47) | |  | 20177 (18350 to 22134) | 2013.41 (1880.36 to 2151.11) | -0.95 (-1.18 to -0.71) |
| Bangladesh | 1598536 (1527672 to 1665861) | 3065.42 (2906.58 to 3217.2) | |  | 3924777 (3722100 to 4140221) | 2975.43 (2820.24 to 3138.57) | -0.07 (-0.17 to 0.03) |
| Barbados | 2394 (2205 to 2594) | 863.86 (793.79 to 938.33) | |  | 4459 (4129 to 4810) | 1039.63 (957.36 to 1124.89) | 0.64 (0.61 to 0.67) |
| Belarus | 367983 (355902 to 380083) | 2963.66 (2871.18 to 3058.08) | |  | 255515 (241451 to 269505) | 1765.18 (1660.59 to 1865.85) | -1.82 (-2.16 to -1.49) |
| Belgium | 493983 (478812 to 509352) | 3373.76 (3263.86 to 3480.3) | |  | 853012 (826328 to 879944) | 3927.75 (3803.69 to 4052.93) | 0.52 (0.5 to 0.54) |
| Belize | 1606 (1500 to 1699) | 1440.62 (1354.73 to 1520.89) | |  | 5738 (5463 to 6028) | 1821.68 (1729.73 to 1917.57) | 0.7 (0.6 to 0.81) |
| Benin | 46526 (44040 to 48894) | 1903.98 (1784.85 to 2020.42) | |  | 122253 (116116 to 128660) | 1928.04 (1809.2 to 2048.92) | 0.02 (-0.08 to 0.11) |
| Bermuda | 752 (695 to 813) | 1194.77 (1110.25 to 1285.06) | |  | 1499 (1401 to 1603) | 1324.04 (1224.46 to 1423.8) | 0.33 (0.29 to 0.37) |
| Bhutan | 8940 (8526 to 9358) | 3357.37 (3170.8 to 3546.26) | |  | 18347 (17318 to 19442) | 3222.65 (3030.15 to 3418.88) | -0.15 (-0.23 to -0.08) |
| Bolivia (Plurinational State of) | 64445 (61397 to 67352) | 1714.92 (1624.88 to 1810.12) | |  | 167240 (157051 to 178097) | 1864.83 (1753.75 to 1985.68) | 0.25 (0.19 to 0.32) |
| Bosnia and Herzegovina | 94640 (89340 to 100335) | 2333.59 (2208.14 to 2478.44) | |  | 122239 (115433 to 130034) | 2257.85 (2134.07 to 2390.52) | -0.17 (-0.3 to -0.03) |
| Botswana | 14865 (13972 to 15774) | 2266.13 (2136.31 to 2406.43) | |  | 33850 (31750 to 36001) | 2265.91 (2126.73 to 2409.33) | -0.03 (-0.14 to 0.09) |
| Brazil | 2929615 (2737000 to 3127815) | 3158.47 (2931.72 to 3392.14) | |  | 6228274 (5745150 to 6755662) | 2655.46 (2453.71 to 2885.75) | -0.65 (-0.72 to -0.57) |
| Brunei Darussalam | 3531 (3295 to 3750) | 3467.82 (3286.4 to 3643.99) | |  | 8122 (7549 to 8690) | 2937.75 (2771.01 to 3106.41) | -0.66 (-0.74 to -0.57) |
| Bulgaria | 280060 (269169 to 290775) | 2477.89 (2392.23 to 2568.81) | |  | 335055 (317939 to 353729) | 2624.62 (2495.63 to 2751.18) | 0.18 (0.13 to 0.24) |
| Burkina Faso | 72450 (68515 to 76226) | 1344.52 (1269.6 to 1422.37) | |  | 180238 (170496 to 189858) | 1451.97 (1372.06 to 1534.4) | 0.2 (0.06 to 0.34) |
| Burundi | 55035 (52126 to 58029) | 1913.62 (1791.76 to 2036.66) | |  | 106070 (99945 to 112433) | 1758.17 (1652.69 to 1871.2) | -0.34 (-0.41 to -0.27) |
| Cabo Verde | 4110 (3906 to 4328) | 1713.41 (1621.45 to 1808.76) | |  | 7318 (6951 to 7690) | 1514.18 (1436.95 to 1597.79) | -0.44 (-0.5 to -0.37) |
| Cambodia | 94154 (88429 to 100341) | 1764.59 (1647.1 to 1886.47) | |  | 271619 (254784 to 288883) | 2214.56 (2082.68 to 2347.71) | 0.77 (0.64 to 0.9) |
| Cameroon | 120996 (114480 to 127614) | 2254.81 (2117.3 to 2394.57) | |  | 333003 (315180 to 351183) | 2216.02 (2082.32 to 2359.98) | -0.05 (-0.41 to 0.31) |
| Canada | 833885 (793343 to 875356) | 2581.95 (2456.95 to 2710.59) | |  | 1818278 (1721234 to 1934105) | 2646.54 (2494.64 to 2810.71) | 0.02 (-0.06 to 0.11) |
| Central African Republic | 27424 (25723 to 29229) | 1920.3 (1789.2 to 2055.71) | |  | 52811 (49579 to 56338) | 1917.83 (1786.5 to 2053.6) | -0.02 (-0.06 to 0.01) |
| Chad | 54923 (52013 to 57962) | 1641.36 (1542.82 to 1748.65) | |  | 128055 (121772 to 134579) | 1680.43 (1579.6 to 1784.17) | 0.01 (-0.03 to 0.06) |
| Chile | 195431 (183368 to 207594) | 1902.42 (1801.74 to 2008.79) | |  | 502110 (472286 to 532266) | 2162.63 (2033.78 to 2291.39) | 0.38 (0.29 to 0.47) |
| China | 27750213 (26163289 to 29381254) | 3300.94 (3104.84 to 3496.06) | |  | 45164960 (41126104 to 49616063) | 2404.42 (2195.92 to 2636.26) | -1.14 (-1.29 to -1) |
| Colombia | 436335 (415771 to 458922) | 2227.03 (2128.67 to 2343.21) | |  | 1319481 (1252085 to 1391749) | 2467.49 (2338.49 to 2605.14) | 0.24 (0.13 to 0.35) |
| Comoros | 4080 (3853 to 4312) | 1520.06 (1432.78 to 1617.81) | |  | 8091 (7559 to 8638) | 1475 (1378.79 to 1568.99) | -0.09 (-0.12 to -0.07) |
| Congo | 25730 (24214 to 27328) | 2047.02 (1917.11 to 2190.49) | |  | 63447 (59272 to 67413) | 2069.35 (1935.61 to 2208.91) | -0.03 (-0.16 to 0.11) |
| Cook Islands | 438 (420 to 456) | 3415.53 (3251.92 to 3574.27) | |  | 791 (750 to 830) | 3349.02 (3187.98 to 3501.3) | -0.08 (-0.09 to -0.06) |
| Costa Rica | 37975 (35402 to 40545) | 2017.16 (1902.39 to 2139.16) | |  | 106907 (101248 to 113958) | 2092.86 (1981.29 to 2230.49) | 0.09 (0.01 to 0.16) |
| Croatia | 137325 (130203 to 144385) | 2266.1 (2154.78 to 2377.48) | |  | 219344 (210000 to 230241) | 2655.73 (2534 to 2789.46) | 0.54 (0.47 to 0.62) |
| Cuba | 189823 (180545 to 200065) | 1825.16 (1739.96 to 1924.35) | |  | 475795 (453102 to 499433) | 2641.23 (2507.38 to 2766.95) | 1.29 (1.26 to 1.33) |
| Cyprus | 21106 (19734 to 22560) | 2759.5 (2596.54 to 2928.65) | |  | 51510 (48462 to 54815) | 2798.82 (2637.83 to 2970.18) | -0.02 (-0.13 to 0.09) |
| Czechia | 269761 (258686 to 281758) | 2107.86 (2023.51 to 2201.15) | |  | 489950 (466462 to 514836) | 2569.85 (2446.66 to 2693.91) | 0.68 (0.61 to 0.76) |
| Côte d’Ivoire | 103099 (97999 to 107932) | 1777.68 (1670.54 to 1887.5) | |  | 248534 (236502 to 261568) | 1697.73 (1601.14 to 1805.05) | -0.16 (-0.2 to -0.12) |
| Democratic People's Republic of Korea | 625083 (616513 to 632480) | 4074.95 (4021.52 to 4123.64) | |  | 1161210 (1120025 to 1196715) | 3718.48 (3588.38 to 3834.48) | -0.34 (-0.4 to -0.29) |
| Democratic Republic of the Congo | 327331 (304148 to 351116) | 1701.07 (1584.44 to 1833) | |  | 868445 (808523 to 929199) | 1998.78 (1865.52 to 2145.67) | 0.55 (0.43 to 0.67) |
| Denmark | 305492 (296696 to 313693) | 3936.02 (3823.89 to 4032.96) | |  | 475839 (464045 to 486314) | 4299.45 (4174.27 to 4411.79) | 0.31 (0.29 to 0.34) |
| Djibouti | 3221 (2981 to 3444) | 1399.97 (1303.68 to 1499.74) | |  | 11476 (10514 to 12402) | 1460.35 (1357.5 to 1572.96) | 0.14 (0.09 to 0.19) |
| Dominica | 721 (668 to 775) | 1044.02 (968.05 to 1127.36) | |  | 1163 (1095 to 1239) | 1371.24 (1286.96 to 1461.33) | 0.96 (0.91 to 1.01) |
| Dominican Republic | 45803 (41800 to 49395) | 945.19 (877.59 to 1010.6) | |  | 129279 (120868 to 137518) | 1316.3 (1235.09 to 1396.79) | 1.1 (0.99 to 1.21) |
| Ecuador | 81756 (77261 to 85742) | 1260.51 (1197.46 to 1316.74) | |  | 217229 (202962 to 232141) | 1445.1 (1358 to 1540.9) | 0.42 (0.31 to 0.54) |
| Egypt | 588953 (556275 to 622076) | 1685.43 (1581.62 to 1790.48) | |  | 1881259 (1787452 to 1965262) | 2731.04 (2588.06 to 2872.04) | 1.69 (1.6 to 1.78) |
| El Salvador | 41545 (38497 to 44518) | 1192.18 (1113.08 to 1270.77) | |  | 101516 (95246 to 108231) | 1630.95 (1529.03 to 1741.2) | 1.1 (1.07 to 1.13) |
| Equatorial Guinea | 3942 (3691 to 4210) | 1701.77 (1589.48 to 1831.95) | |  | 11740 (10799 to 12645) | 1850.12 (1710.53 to 1992.41) | 0.2 (0.07 to 0.33) |
| Eritrea | 25679 (24242 to 27128) | 1778.45 (1657.57 to 1897.62) | |  | 67089 (63358 to 71102) | 1814.41 (1697.16 to 1939.95) | 0.03 (-0.04 to 0.09) |
| Estonia | 27092 (25675 to 28728) | 1417.77 (1343.07 to 1504.9) | |  | 31791 (30055 to 33696) | 1380.85 (1291.2 to 1472.88) | -0.09 (-0.21 to 0.04) |
| Eswatini | 9148 (8719 to 9553) | 2602.9 (2463.78 to 2735.5) | |  | 16362 (15605 to 17119) | 2476.74 (2347.7 to 2605.9) | -0.18 (-0.29 to -0.06) |
| Ethiopia | 420126 (386414 to 455091) | 1631.07 (1492.81 to 1792.47) | |  | 683970 (612886 to 751405) | 1182.15 (1068.06 to 1302.67) | -1.18 (-1.28 to -1.09) |
| Fiji | 3840 (3548 to 4123) | 933.9 (870.26 to 1001.64) | |  | 4710 (4355 to 5056) | 668.54 (622.11 to 713.73) | -1.14 (-1.19 to -1.1) |
| Finland | 149351 (142437 to 156800) | 2220.74 (2115.35 to 2337.68) | |  | 243426 (229106 to 257920) | 2199.6 (2060.4 to 2341.84) | -0.03 (-0.11 to 0.04) |
| France | 1932582 (1844226 to 2034665) | 2406.71 (2297.1 to 2531.7) | |  | 2543487 (2406078 to 2699316) | 1966.72 (1850.71 to 2089.27) | -0.75 (-0.87 to -0.63) |
| Gabon | 10174 (9415 to 10917) | 1646.89 (1535.8 to 1767.66) | |  | 19732 (18295 to 21159) | 1636.89 (1529.56 to 1752.21) | -0.06 (-0.14 to 0.02) |
| Gambia | 8944 (8499 to 9411) | 1899.47 (1785.43 to 2019.79) | |  | 24182 (22965 to 25460) | 2004.45 (1882.6 to 2132.91) | 0.14 (0.09 to 0.18) |
| Georgia | 90103 (85628 to 94865) | 1531.69 (1458.99 to 1608.2) | |  | 132801 (126067 to 140137) | 2373.07 (2260.86 to 2495.19) | 1.48 (1.25 to 1.7) |
| Germany | 3596678 (3446048 to 3758539) | 2990.05 (2857.3 to 3120.38) | |  | 6130746 (5844770 to 6420710) | 3433.36 (3263.75 to 3592.14) | 0.48 (0.4 to 0.56) |
| Ghana | 120121 (114921 to 125622) | 1409.26 (1341.44 to 1485.31) | |  | 353063 (335031 to 371410) | 1734.29 (1637.77 to 1833.28) | 0.71 (0.69 to 0.74) |
| Greece | 353147 (334886 to 373895) | 2490.32 (2362.61 to 2631.73) | |  | 657517 (626678 to 693971) | 2824.21 (2694.83 to 2976.45) | 0.41 (0.35 to 0.47) |
| Greenland | 1143 (1087 to 1195) | 3505.38 (3384.37 to 3625.87) | |  | 2126 (2041 to 2215) | 3304.59 (3167.65 to 3442.66) | -0.21 (-0.25 to -0.17) |
| Grenada | 935 (878 to 991) | 1292.44 (1213.03 to 1369.82) | |  | 1673 (1570 to 1778) | 1506.35 (1412.46 to 1596.66) | 0.49 (0.45 to 0.53) |
| Guam | 985 (920 to 1049) | 1311.35 (1234.95 to 1389.36) | |  | 1828 (1724 to 1945) | 1010.01 (951.01 to 1073.97) | -0.9 (-0.94 to -0.86) |
| Guatemala | 50218 (47224 to 52993) | 1071.15 (1014.24 to 1127.42) | |  | 154061 (143133 to 165670) | 1215.25 (1139.75 to 1296.4) | 0.41 (0.36 to 0.46) |
| Guinea | 65397 (61747 to 69051) | 1731.46 (1624.24 to 1834.93) | |  | 134216 (126884 to 141604) | 1979.37 (1858.57 to 2108.38) | 0.45 (0.35 to 0.56) |
| Guinea-Bissau | 10332 (9742 to 10929) | 2041.19 (1901.9 to 2182.61) | |  | 19764 (18753 to 20857) | 2031.75 (1908.06 to 2164.6) | -0.06 (-0.13 to 0) |
| Guyana | 5026 (4572 to 5507) | 1009.63 (938.56 to 1083.93) | |  | 8896 (8278 to 9525) | 1302.95 (1219.92 to 1389.46) | 0.86 (0.84 to 0.88) |
| Haiti | 55627 (51579 to 59542) | 1439.88 (1342.41 to 1543.22) | |  | 145127 (134824 to 154924) | 1810.24 (1689.87 to 1935.85) | 0.81 (0.77 to 0.85) |
| Honduras | 45454 (43362 to 47707) | 1807.48 (1713.09 to 1914.17) | |  | 147922 (139987 to 157212) | 2254.91 (2123.07 to 2410.31) | 0.73 (0.7 to 0.77) |
| Hungary | 400635 (385737 to 418595) | 2931.8 (2835.25 to 3047.32) | |  | 612233 (592009 to 634589) | 3465.61 (3369.09 to 3568.66) | 0.58 (0.55 to 0.61) |
| Iceland | 8611 (8164 to 9066) | 3040.59 (2878.16 to 3200.5) | |  | 16033 (15245 to 16915) | 2938.18 (2786.14 to 3103.19) | -0.16 (-0.21 to -0.11) |
| India | 16493706 (15757382 to 17147028) | 3680.59 (3500.35 to 3835.37) | |  | 37874659 (35668470 to 40143766) | 3395.14 (3192.96 to 3594.53) | -0.29 (-0.31 to -0.27) |
| Indonesia | 2524627 (2342195 to 2716669) | 2373.13 (2180.49 to 2582.4) | |  | 5147353 (4754573 to 5557902) | 2359.15 (2179.16 to 2567.13) | -0.06 (-0.1 to -0.01) |
| Iran (Islamic Republic of) | 509008 (465525 to 555495) | 1640 (1508.23 to 1794.92) | |  | 1562415 (1446402 to 1688536) | 2055.51 (1891.37 to 2232.56) | 0.76 (0.72 to 0.8) |
| Iraq | 144140 (135387 to 152870) | 1481.17 (1390.15 to 1578) | |  | 308960 (281559 to 338889) | 1053.46 (976.69 to 1139.4) | -1.26 (-1.37 to -1.14) |
| Ireland | 166701 (161683 to 171505) | 4169.03 (4040.61 to 4293.89) | |  | 267359 (254770 to 280102) | 3656.14 (3483.57 to 3826.95) | -0.48 (-0.53 to -0.43) |
| Israel | 114299 (108540 to 121163) | 2460.34 (2342.78 to 2604.45) | |  | 253531 (240808 to 267767) | 2256.05 (2138.43 to 2389.57) | -0.34 (-0.45 to -0.23) |
| Italy | 2266653 (2084678 to 2452655) | 2684.64 (2474.15 to 2898.76) | |  | 3253110 (2970227 to 3542194) | 2287.08 (2094.5 to 2491.19) | -0.57 (-0.62 to -0.51) |
| Jamaica | 29519 (27777 to 31240) | 1595.46 (1503.09 to 1690.81) | |  | 55521 (52686 to 58296) | 1833.32 (1738.82 to 1927.2) | 0.42 (0.26 to 0.58) |
| Japan | 2996186 (2715338 to 3305788) | 1876.99 (1709.5 to 2062.45) | |  | 4789562 (4350538 to 5272870) | 1354.19 (1226.48 to 1487.23) | -1.14 (-1.24 to -1.05) |
| Jordan | 31615 (29675 to 33533) | 1744.28 (1641.58 to 1850.1) | |  | 119919 (110359 to 129523) | 1602.29 (1504.3 to 1706.57) | -0.37 (-0.5 to -0.25) |
| Kazakhstan | 397675 (387352 to 409104) | 3057.07 (2968.09 to 3154.09) | |  | 524697 (508006 to 542490) | 3052.58 (2948.61 to 3158.75) | 0.03 (-0.03 to 0.09) |
| Kenya | 183853 (170126 to 199017) | 1718.09 (1575.26 to 1882.2) | |  | 488588 (452563 to 528500) | 1730.38 (1589.64 to 1892.54) | -0.02 (-0.05 to 0.01) |
| Kiribati | 463 (437 to 488) | 1021.74 (963.61 to 1078.79) | |  | 796 (750 to 843) | 1019.36 (959.19 to 1084.76) | 0.01 (-0.09 to 0.11) |
| Kuwait | 11102 (9849 to 12363) | 995.02 (920.69 to 1075.8) | |  | 39087 (34844 to 43652) | 1023.09 (939.34 to 1115.6) | -0.01 (-0.26 to 0.25) |
| Kyrgyzstan | 115349 (112489 to 118304) | 3725.91 (3620.02 to 3832.96) | |  | 138722 (133002 to 144148) | 2955.62 (2821.78 to 3080.09) | -0.82 (-0.97 to -0.68) |
| Lao People's Democratic Republic | 53007 (49361 to 56745) | 2310.01 (2143.58 to 2485.28) | |  | 130239 (122407 to 137856) | 2741.77 (2568.28 to 2911.16) | 0.54 (0.51 to 0.58) |
| Latvia | 52673 (49903 to 55610) | 1589.69 (1509.32 to 1672.81) | |  | 43304 (40889 to 45941) | 1293.34 (1210.19 to 1380.33) | -0.59 (-0.88 to -0.29) |
| Lebanon | 45238 (42396 to 48187) | 1904.53 (1788.11 to 2030.26) | |  | 141237 (131990 to 150458) | 2723.12 (2548.32 to 2901.71) | 1.25 (1.2 to 1.29) |
| Lesotho | 27307 (25940 to 28622) | 2532.44 (2397.92 to 2664.87) | |  | 38827 (37057 to 40680) | 2815.34 (2670.69 to 2960.59) | 0.36 (0.3 to 0.42) |
| Liberia | 16399 (15484 to 17293) | 1283.07 (1210.15 to 1355.5) | |  | 42788 (40388 to 45276) | 1520.44 (1430.59 to 1609.41) | 0.53 (0.44 to 0.62) |
| Libya | 39402 (37040 to 41923) | 1714.15 (1606.27 to 1832.55) | |  | 139857 (131289 to 147932) | 2460.98 (2306.8 to 2612.15) | 1.24 (1.19 to 1.28) |
| Lithuania | 109415 (105712 to 113475) | 2505.79 (2418.82 to 2595.75) | |  | 76722 (72967 to 80785) | 1544.91 (1463.6 to 1634.47) | -1.67 (-1.83 to -1.51) |
| Luxembourg | 17174 (16525 to 17875) | 3283.28 (3156.1 to 3415.51) | |  | 33204 (31539 to 35080) | 3361.99 (3190.15 to 3558.42) | 0.09 (0.03 to 0.14) |
| Madagascar | 122717 (116293 to 129152) | 1951.59 (1833.67 to 2068.99) | |  | 304017 (286418 to 321707) | 2171.05 (2027.2 to 2318.99) | 0.28 (0.16 to 0.4) |
| Malawi | 71428 (66472 to 76365) | 1411.62 (1319.4 to 1512.45) | |  | 147080 (137355 to 156496) | 1492.5 (1404.83 to 1586.97) | 0.14 (0.06 to 0.22) |
| Malaysia | 207562 (194105 to 221837) | 2171 (2054.69 to 2305.51) | |  | 548253 (515596 to 585959) | 2057.23 (1944.5 to 2190.39) | -0.26 (-0.37 to -0.14) |
| Maldives | 3371 (3270 to 3465) | 3539.75 (3422 to 3661.59) | |  | 10414 (9859 to 10943) | 3199.88 (3030.77 to 3371.74) | -0.44 (-0.58 to -0.3) |
| Mali | 103367 (96538 to 110605) | 2121.2 (1974.77 to 2275.48) | |  | 247544 (232568 to 261553) | 2326.9 (2175.58 to 2479.62) | 0.3 (0.17 to 0.43) |
| Malta | 10624 (10071 to 11231) | 2583.02 (2449.33 to 2727.98) | |  | 20055 (18967 to 21195) | 2329.09 (2193.27 to 2459.96) | -0.41 (-0.5 to -0.32) |
| Marshall Islands | 391 (373 to 408) | 1951.2 (1837.53 to 2070.02) | |  | 591 (562 to 619) | 1581.61 (1494.24 to 1673.66) | -0.74 (-0.84 to -0.65) |
| Mauritania | 21593 (20455 to 22770) | 1825.83 (1716.67 to 1938.39) | |  | 42163 (39788 to 44643) | 1715.34 (1615.97 to 1823.62) | -0.25 (-0.29 to -0.21) |
| Mauritius | 17701 (16878 to 18543) | 2258.92 (2155.71 to 2372.41) | |  | 32393 (30706 to 34221) | 1984.59 (1882.47 to 2085.96) | -0.53 (-0.61 to -0.46) |
| Mexico | 1024529 (940755 to 1109586) | 2181.38 (1995.81 to 2384.67) | |  | 2600960 (2385776 to 2844272) | 2247.21 (2053.18 to 2462.97) | 0.06 (-0.01 to 0.13) |
| Micronesia (Federated States of) | 979 (930 to 1035) | 1887.91 (1776.34 to 2023.51) | |  | 1092 (1030 to 1156) | 1575.61 (1485.85 to 1669.59) | -0.6 (-0.9 to -0.3) |
| Monaco | 2089 (1888 to 2201) | 3171.41 (2940.97 to 3338.28) | |  | 2785 (2627 to 2928) | 3094.6 (2928.02 to 3256.11) | -0.09 (-0.11 to -0.06) |
| Mongolia | 20577 (19548 to 21624) | 1641.26 (1549.73 to 1737.26) | |  | 33038 (30410 to 35625) | 1266.2 (1179.48 to 1354.73) | -0.95 (-1.07 to -0.83) |
| Montenegro | 7327 (6783 to 7959) | 1200.01 (1112.52 to 1296.55) | |  | 10750 (9940 to 11582) | 1245.21 (1149.21 to 1344.76) | 0.15 (0.09 to 0.21) |
| Morocco | 223596 (208137 to 238935) | 1365.59 (1274.86 to 1460.35) | |  | 611177 (573610 to 650326) | 1896.68 (1782.42 to 2018.8) | 1.14 (1.12 to 1.17) |
| Mozambique | 95739 (88644 to 102826) | 1246.47 (1161.83 to 1339.86) | |  | 234441 (220557 to 247596) | 1557.68 (1459.98 to 1650.99) | 0.74 (0.67 to 0.81) |
| Myanmar | 827852 (787278 to 867068) | 3526.26 (3334.5 to 3718.35) | |  | 1791217 (1732159 to 1848603) | 3963.73 (3825.41 to 4096.67) | 0.35 (0.29 to 0.4) |
| Namibia | 18823 (17786 to 19847) | 2369.13 (2241.37 to 2510.04) | |  | 33664 (31536 to 35779) | 2191.5 (2063.38 to 2329.54) | -0.29 (-0.41 to -0.17) |
| Nauru | 134 (130 to 139) | 3206.19 (3054.94 to 3357.12) | |  | 142 (136 to 148) | 3076.73 (2924.96 to 3237.96) | -0.12 (-0.19 to -0.06) |
| Nepal | 334833 (319053 to 350059) | 3439.39 (3250.43 to 3622.3) | |  | 803246 (764028 to 839952) | 3651.03 (3476.38 to 3824.18) | 0.2 (0.19 to 0.21) |
| Netherlands | 678338 (653219 to 703348) | 3505.64 (3364.75 to 3638.48) | |  | 1271027 (1220730 to 1321914) | 3863.8 (3695.17 to 4023.14) | 0.33 (0.26 to 0.39) |
| New Zealand | 141447 (129989 to 151923) | 3672.77 (3382.92 to 3945.18) | |  | 255810 (234240 to 278107) | 3384.32 (3116.83 to 3667.15) | -0.31 (-0.37 to -0.25) |
| Nicaragua | 23369 (21263 to 25417) | 1170.06 (1089.7 to 1250.26) | |  | 81712 (76164 to 87520) | 1773.39 (1668.12 to 1888.41) | 1.42 (1.39 to 1.46) |
| Niger | 62840 (59577 to 66161) | 1633.79 (1529.03 to 1749.47) | |  | 179661 (169575 to 189387) | 1718.54 (1606.74 to 1832.13) | 0.15 (0.11 to 0.2) |
| Nigeria | 855433 (795129 to 923742) | 1646.14 (1514.67 to 1797.3) | |  | 1587743 (1449908 to 1718544) | 1355.99 (1245.04 to 1478.43) | -0.72 (-0.79 to -0.64) |
| Niue | 70 (66 to 74) | 3160.96 (3001.27 to 3330.63) | |  | 67 (63 to 70) | 3191.08 (3032.23 to 3371.92) | 0.03 (0 to 0.05) |
| North Macedonia | 36119 (34120 to 38221) | 1964.98 (1859.67 to 2073) | |  | 57366 (53942 to 61033) | 1966.8 (1857.01 to 2083.62) | -0.03 (-0.07 to 0.02) |
| Northern Mariana Islands | 333 (309 to 358) | 1608.78 (1514.79 to 1709.35) | |  | 470 (440 to 501) | 1167.64 (1096.76 to 1238.3) | -1.14 (-1.21 to -1.07) |
| Norway | 191196 (174596 to 208571) | 2961.46 (2728.11 to 3219.33) | |  | 346210 (320635 to 371915) | 3705.18 (3434.47 to 3982.66) | 0.77 (0.72 to 0.82) |
| Oman | 8852 (7578 to 10134) | 876.48 (784.37 to 968.72) | |  | 33346 (28850 to 38642) | 1281.21 (1172.55 to 1395.83) | 1.38 (1.3 to 1.45) |
| Pakistan | 1602936 (1485122 to 1734122) | 2639.12 (2428.51 to 2879.3) | |  | 3056397 (2839592 to 3292395) | 2598.59 (2378.47 to 2830.99) | -0.05 (-0.11 to 0.01) |
| Palau | 336 (321 to 349) | 3339 (3172.48 to 3489.54) | |  | 632 (601 to 664) | 3186.06 (3040.65 to 3344.03) | -0.18 (-0.2 to -0.15) |
| Palestine | 17881 (16699 to 18965) | 1707.26 (1607.6 to 1810.56) | |  | 44568 (40888 to 48163) | 1552.66 (1454.08 to 1659.82) | -0.37 (-0.48 to -0.26) |
| Panama | 24821 (23169 to 26629) | 1503.76 (1413.64 to 1598.14) | |  | 66877 (62966 to 71327) | 1584.03 (1490.81 to 1690.84) | 0.1 (0.03 to 0.17) |
| Papua New Guinea | 60711 (59419 to 61815) | 3120.03 (3045.49 to 3191.19) | |  | 156629 (152109 to 160383) | 3110.77 (3012.9 to 3197.4) | -0.01 (-0.02 to -0.01) |
| Paraguay | 39420 (37322 to 41409) | 1540.5 (1463.79 to 1619.69) | |  | 101794 (95740 to 107746) | 1741.95 (1641.47 to 1846.28) | 0.41 (0.37 to 0.45) |
| Peru | 154056 (143144 to 163783) | 1003.53 (939.11 to 1063.66) | |  | 417387 (388012 to 446187) | 1250.73 (1164.05 to 1336.16) | 0.83 (0.66 to 1.01) |
| Philippines | 703251 (657004 to 751419) | 2050.24 (1892.74 to 2215.15) | |  | 1929067 (1804907 to 2063892) | 2369.23 (2198.59 to 2555.03) | 0.48 (0.45 to 0.51) |
| Poland | 1018655 (939695 to 1104555) | 2402.8 (2220.69 to 2600.96) | |  | 1430280 (1307014 to 1572224) | 2210.3 (2026.35 to 2414.97) | -0.31 (-0.37 to -0.24) |
| Portugal | 387425 (372544 to 403279) | 2994.6 (2885.32 to 3109.89) | |  | 630134 (601544 to 663586) | 2692.98 (2558.47 to 2839.44) | -0.37 (-0.46 to -0.28) |
| Puerto Rico | 69216 (65785 to 72895) | 1944.23 (1849.74 to 2045.31) | |  | 141664 (134603 to 149233) | 2170.59 (2061.19 to 2290.97) | 0.38 (0.28 to 0.47) |
| Qatar | 4075 (3757 to 4378) | 1981.81 (1865.76 to 2100.98) | |  | 26415 (23313 to 29791) | 1754.91 (1633.38 to 1886.32) | -0.37 (-0.61 to -0.14) |
| Republic of Korea | 538428 (498278 to 577133) | 1828.38 (1720.91 to 1945.98) | |  | 1669944 (1582268 to 1761412) | 2027.25 (1921.51 to 2140.83) | 0.22 (0.04 to 0.39) |
| Republic of Moldova | 129046 (124839 to 133416) | 2986.73 (2889.31 to 3093.27) | |  | 103794 (98916 to 109202) | 1930.89 (1836.53 to 2028.22) | -1.55 (-1.69 to -1.42) |
| Romania | 748063 (720936 to 776844) | 2879.82 (2775.93 to 2985.83) | |  | 772272 (740259 to 807347) | 2329.31 (2237.28 to 2430.04) | -0.77 (-0.82 to -0.72) |
| Russian Federation | 3819371 (3549316 to 4102811) | 2229.03 (2073.98 to 2393.17) | |  | 3921088 (3641585 to 4227149) | 1823.84 (1699.82 to 1960.33) | -0.71 (-0.84 to -0.58) |
| Rwanda | 69303 (65700 to 72836) | 1907.35 (1794.16 to 2023.63) | |  | 134377 (126021 to 143261) | 1806.06 (1693.06 to 1937.85) | -0.26 (-0.38 to -0.14) |
| Saint Kitts and Nevis | 517 (489 to 549) | 1449.89 (1378.28 to 1531.87) | |  | 1228 (1157 to 1299) | 1854.17 (1753.86 to 1956.14) | 0.81 (0.78 to 0.85) |
| Saint Lucia | 1515 (1430 to 1598) | 1594.89 (1513.12 to 1682.87) | |  | 4124 (3914 to 4335) | 1950.32 (1855.4 to 2049.48) | 0.67 (0.63 to 0.7) |
| Saint Vincent and the Grenadines | 703 (637 to 770) | 871.82 (800.27 to 947.5) | |  | 1483 (1386 to 1589) | 1144.76 (1067.83 to 1225.64) | 0.91 (0.83 to 0.99) |
| Samoa | 1638 (1558 to 1719) | 1734.87 (1636.61 to 1842.91) | |  | 1894 (1774 to 2013) | 1287.61 (1203.19 to 1375.8) | -1.01 (-1.07 to -0.96) |
| San Marino | 1021 (961 to 1066) | 3226.41 (3053.35 to 3367.9) | |  | 1954 (1854 to 2036) | 3132.77 (2979.24 to 3275.02) | -0.12 (-0.14 to -0.1) |
| Sao Tome and Principe | 1750 (1651 to 1851) | 2535.49 (2382.82 to 2684.48) | |  | 3934 (3787 to 4087) | 3391.96 (3221.63 to 3560.04) | 1 (0.92 to 1.07) |
| Saudi Arabia | 101104 (91334 to 111224) | 1381.26 (1285.35 to 1484.96) | |  | 434561 (396012 to 473597) | 2053.04 (1918.06 to 2194.29) | 1.41 (1.3 to 1.53) |
| Senegal | 69441 (65831 to 72810) | 1732.22 (1630.1 to 1833.61) | |  | 159836 (151194 to 168298) | 1758.62 (1655.07 to 1860.78) | 0.02 (-0.01 to 0.06) |
| Serbia | 251479 (238446 to 265655) | 2294.9 (2176.11 to 2418.81) | |  | 417284 (394857 to 440846) | 2899.77 (2758.79 to 3055.05) | 0.77 (0.7 to 0.84) |
| Seychelles | 1106 (1047 to 1165) | 1873.55 (1771.48 to 1976.46) | |  | 2377 (2249 to 2512) | 2171.46 (2056.57 to 2290.81) | 0.5 (0.43 to 0.57) |
| Sierra Leone | 37561 (35682 to 39399) | 1672.35 (1576.55 to 1767.76) | |  | 86262 (81988 to 90499) | 1874.5 (1767.03 to 1986.51) | 0.39 (0.27 to 0.5) |
| Singapore | 61138 (58372 to 64211) | 2845.02 (2748.13 to 2948.6) | |  | 113408 (106305 to 121242) | 1499.9 (1407.53 to 1598.61) | -2.25 (-2.45 to -2.05) |
| Slovakia | 90286 (85060 to 95903) | 1581.41 (1491.51 to 1679.5) | |  | 156589 (147908 to 166436) | 1870.03 (1764.26 to 1983.12) | 0.62 (0.5 to 0.73) |
| Slovenia | 56688 (53864 to 59428) | 2405.22 (2286.7 to 2521.9) | |  | 78459 (74282 to 82875) | 1897.02 (1784.22 to 2019.69) | -0.85 (-0.92 to -0.78) |
| Solomon Islands | 2541 (2391 to 2690) | 1649.81 (1546.99 to 1757.06) | |  | 4941 (4617 to 5265) | 1478.48 (1386.41 to 1575.48) | -0.35 (-0.39 to -0.31) |
| Somalia | 56230 (52735 to 59955) | 1586.17 (1482.83 to 1704.87) | |  | 147261 (136640 to 157532) | 1518.23 (1409.24 to 1627.78) | -0.14 (-0.24 to -0.05) |
| South Africa | 591669 (553135 to 632435) | 2583.24 (2398.03 to 2785.9) | |  | 1046594 (970945 to 1127782) | 2256 (2087.84 to 2439.82) | -0.52 (-0.61 to -0.43) |
| South Sudan | 50044 (46875 to 53197) | 1631.46 (1522.95 to 1752.87) | |  | 79130 (73518 to 84549) | 1568.18 (1457.98 to 1683.72) | -0.21 (-0.33 to -0.1) |
| Spain | 1656213 (1603124 to 1705537) | 3190.96 (3088.18 to 3282.26) | |  | 2905818 (2788360 to 3034266) | 3081.85 (2935.31 to 3235.71) | -0.15 (-0.2 to -0.1) |
| Sri Lanka | 231017 (216827 to 245539) | 1916.16 (1797.12 to 2040.6) | |  | 561222 (526822 to 596238) | 2330.45 (2193.44 to 2463.67) | 0.68 (0.57 to 0.79) |
| Sudan | 177280 (165885 to 189352) | 1574.03 (1462.64 to 1696.99) | |  | 428128 (401529 to 455381) | 1862.62 (1748.03 to 1989.12) | 0.54 (0.5 to 0.59) |
| Suriname | 3807 (3564 to 4051) | 1298.52 (1221.88 to 1374.45) | |  | 8972 (8466 to 9493) | 1492.66 (1413.57 to 1574.86) | 0.5 (0.38 to 0.62) |
| Sweden | 409237 (371381 to 450841) | 2916.14 (2663.84 to 3175.32) | |  | 657306 (598730 to 721926) | 3216.73 (2956.64 to 3508.36) | 0.4 (0.36 to 0.44) |
| Switzerland | 286322 (272611 to 300657) | 2872.83 (2727.81 to 3017.35) | |  | 427179 (405500 to 450249) | 2553.09 (2423.99 to 2699.14) | -0.47 (-0.54 to -0.4) |
| Syrian Arab Republic | 108889 (102798 to 114953) | 1651.08 (1557.43 to 1748.47) | |  | 249284 (233862 to 265450) | 2017.27 (1902.96 to 2137.82) | 0.68 (0.54 to 0.82) |
| Taiwan (Province of China) | 285820 (267523 to 305150) | 1833.8 (1727.99 to 1936.18) | |  | 387404 (364073 to 413465) | 1058.63 (987.66 to 1135.6) | -1.93 (-2.02 to -1.84) |
| Tajikistan | 73630 (70600 to 76891) | 2294.52 (2182.99 to 2411.53) | |  | 113590 (108025 to 118875) | 1777.96 (1683.2 to 1874.92) | -0.91 (-0.99 to -0.83) |
| Thailand | 1033078 (991954 to 1075733) | 2687.33 (2566.16 to 2820.69) | |  | 1906660 (1799459 to 2015166) | 1968.61 (1860.19 to 2079.1) | -1.18 (-1.36 to -1.01) |
| Timor-Leste | 7099 (6706 to 7509) | 1900.82 (1771.33 to 2036.61) | |  | 20767 (19536 to 22102) | 2492.41 (2344.77 to 2649.63) | 0.91 (0.88 to 0.95) |
| Togo | 32803 (31145 to 34368) | 1953.09 (1836.79 to 2073.35) | |  | 91070 (86074 to 96044) | 1969.17 (1849.72 to 2094.94) | 0.01 (-0.11 to 0.12) |
| Tokelau | 42 (40 to 44) | 3162.19 (3013.22 to 3304.78) | |  | 42 (39 to 44) | 3210.07 (3052.13 to 3364.83) | 0.04 (0.02 to 0.06) |
| Tonga | 817 (766 to 866) | 1482.87 (1394.22 to 1575.71) | |  | 978 (918 to 1034) | 1200.72 (1126.43 to 1273.35) | -0.74 (-0.78 to -0.7) |
| Trinidad and Tobago | 11601 (10918 to 12263) | 1231.13 (1161.07 to 1297.06) | |  | 20158 (18971 to 21443) | 1166.94 (1096.27 to 1240.27) | -0.25 (-0.34 to -0.16) |
| Tunisia | 82010 (75916 to 87875) | 1543.48 (1445.83 to 1643.85) | |  | 249513 (233445 to 266354) | 2048.55 (1916.78 to 2186.19) | 1.03 (0.94 to 1.11) |
| Turkey | 1007579 (965697 to 1047402) | 2552.71 (2439.74 to 2667.99) | |  | 2885498 (2798099 to 2967100) | 3287.12 (3187.36 to 3380.3) | 0.88 (0.81 to 0.95) |
| Turkmenistan | 54792 (52799 to 56772) | 2548.33 (2440.35 to 2649.03) | |  | 56871 (53880 to 60045) | 1326.9 (1260.85 to 1398.18) | -2.27 (-2.45 to -2.09) |
| Tuvalu | 203 (192 to 213) | 3025.21 (2862.76 to 3187.12) | |  | 289 (274 to 306) | 2891.4 (2732.05 to 3064.48) | -0.19 (-0.21 to -0.16) |
| Uganda | 131626 (123059 to 140048) | 1561.92 (1455.32 to 1674.52) | |  | 303362 (278483 to 326245) | 1556.28 (1450.33 to 1661.88) | -0.1 (-0.23 to 0.03) |
| Ukraine | 2326649 (2161765 to 2500178) | 3451.34 (3219.34 to 3691.45) | |  | 1284752 (1180459 to 1402555) | 1878.68 (1736.26 to 2032.23) | -2.11 (-2.34 to -1.89) |
| United Arab Emirates | 20338 (19552 to 21023) | 2253.02 (2126.03 to 2379.72) | |  | 191068 (187010 to 194836) | 2926.88 (2811.15 to 3046.44) | 0.88 (0.84 to 0.91) |
| United Kingdom | 3206010 (2992311 to 3407355) | 3701.59 (3476.97 to 3923.31) | |  | 4727607 (4443749 to 5006429) | 3902.15 (3678.51 to 4125.74) | 0.17 (0.15 to 0.19) |
| United Republic of Tanzania | 175315 (161450 to 187732) | 1233.06 (1145.86 to 1316.74) | |  | 423197 (389806 to 453143) | 1299.49 (1208.35 to 1385.44) | 0.24 (0.11 to 0.38) |
| United States of America | 11410071 (10538686 to 12133375) | 3622.08 (3363.69 to 3846.2) | |  | 20147917 (19242749 to 20933265) | 3668.54 (3514.64 to 3805.41) | 0.04 (0 to 0.08) |
| United States Virgin Islands | 950 (866 to 1037) | 1051.93 (969.09 to 1137.36) | |  | 2196 (2046 to 2362) | 1376.06 (1279.59 to 1484.11) | 0.95 (0.88 to 1.02) |
| Uruguay | 90928 (87468 to 94303) | 2461.98 (2369.29 to 2550.05) | |  | 162524 (156155 to 168419) | 3173.29 (3057.29 to 3286.71) | 0.9 (0.84 to 0.95) |
| Uzbekistan | 313920 (300988 to 326459) | 2471.75 (2360.18 to 2581.36) | |  | 367628 (344409 to 389220) | 1562.64 (1480.89 to 1643.78) | -1.66 (-2.07 to -1.25) |
| Vanuatu | 1478 (1400 to 1560) | 1980.45 (1857.98 to 2121) | |  | 3031 (2844 to 3231) | 1627.39 (1520.08 to 1741.72) | -0.65 (-0.7 to -0.6) |
| Venezuela (Bolivarian Republic of) | 172873 (160288 to 184266) | 1488.21 (1407.94 to 1575.94) | |  | 578386 (546082 to 614104) | 2003 (1890.04 to 2126.71) | 1.02 (0.99 to 1.05) |
| Viet Nam | 884734 (831478 to 940456) | 2048.11 (1928.09 to 2181.97) | |  | 2628515 (2501573 to 2756167) | 2880.7 (2743.59 to 3024.59) | 1.16 (1.13 to 1.2) |
| Yemen | 110325 (103679 to 117954) | 1732.82 (1612.14 to 1879.26) | |  | 342941 (323483 to 363074) | 2064.96 (1932.8 to 2207.03) | 0.63 (0.6 to 0.66) |
| Zambia | 59144 (55677 to 62693) | 1521.91 (1427.68 to 1627.89) | |  | 152038 (141443 to 161813) | 1650.04 (1551.09 to 1755.41) | 0.29 (0.14 to 0.43) |
| Zimbabwe | 96979 (91167 to 103222) | 1944.51 (1832.02 to 2065.36) | |  | 168183 (157789 to 179079) | 1991.8 (1861.45 to 2134.23) | 0.07 (-0.03 to 0.17) |

COPD: chronic obstructive pulmonary disease; AAPC: average annual percent change; UI: uncertainty interval; CI: confidence interval; SDI: Socio-demographic index.

Table S4. Prevalence of asthma (1990/2019) for sexes, SDI and locations, with AAPC (1990-2019)

| GBD data | 1990 | |  | 2019 | | | AAPC % (95% CI)  1990-2019 |
| --- | --- | --- | --- | --- | --- | --- | --- |
|  | Cases (95% UI) | Age-standardised prevalence per  100 000 population (95% UI) |  | Cases (95% UI) | Age-standardised prevalence per  100 000 population (95% UI) | |  |
| Global | 226904790 (195205509 to 269078167) | 4496.93 (3913.55 to 5224.32) |  | 262405182 (224047914 to 309452681) | | 3415.53 (2898.92 to 4066.2) | -0.91 (-1.04 to -0.79) |
| **Sex** |  |  |  |  |  | |  |
| Female | 117519064 (101697699 to 137326170) | 4657.93 (4078.24 to 5381.05) |  | 135641710 (116701201 to 157898921) | | 3488.49 (2982.65 to 4112.59) | -0.98 (-1.09 to -0.88) |
| Male | 109385726 (93205287 to 131711665) | 4342.34 (3761.89 to 5126.4) |  | 126763472 (106760386 to 152696253) | | 3332.81 (2804.34 to 4028.84) | -0.86 (-1.02 to -0.69) |
| **SDI** |  |  |  |  |  | |  |
| High SDI | 71267451 (62525167 to 82436645) | 8329.46 (7264.7 to 9693.09) |  | 68952653 (60703694 to 78756181) | 6855.06 (5876.69 to 8058.48) | | -0.7 (-0.84 to -0.56) |
| High-middle SDI | 47644397 (40601957 to 56426837) | 4240.89 (3624.32 to 5025.44) |  | 42846686 (35939860 to 51183980) | 3103.02 (2541.3 to 3833.34) | | -1.1 (-1.41 to -0.8) |
| Middle SDI | 54683036 (45361677 to 67653910) | 3268.82 (2764.99 to 3919.42) |  | 67251100 (56236271 to 81268923) | 2927.13 (2429.34 to 3578.75) | | -0.36 (-0.54 to -0.18) |
| Low-middle SDI | 32578711 (27508062 to 39391204) | 3154.12 (2737.24 to 3679.79) |  | 45235060 (38351484 to 54097332) | 2713.25 (2317.27 to 3209.72) | | -0.46 (-0.62 to -0.29) |
| Low SDI | 20556433 (17394826 to 25114690) | 4007.06 (3512.75 to 4592.39) |  | 37911430 (31439353 to 46543194) | 3468.63 (3015.27 to 4035.78) | | -0.47 (-0.54 to -0.39) |
| **Region** |  |  |  |  |  | |  |
| High-income Asia Pacific | 13119777 (11259128 to 15128897) | 7293.59 (6258.92 to 8484.18) |  | 7250096 (6115953 to 8632440) | 3744.86 (3032.34 to 4726.2) | | -2.46 (-2.69 to -2.23) |
| High-income North America | 24907789 (21234419 to 29735299) | 8986.5 (7533.24 to 10805) |  | 35609106 (31843996 to 39983534) | 9848.14 (8624.26 to 11312.08) | | 0.59 (0.17 to 1.02) |
| Western Europe | 36305394 (31890731 to 41260353) | 8332.15 (7287.97 to 9673.84) |  | 27043663 (22873450 to 31920142) | 5893.41 (4900.26 to 7117.65) | | -1.44 (-1.74 to -1.14) |
| Australasia | 2416862 (2231065 to 2617852) | 12086.37 (11123.78 to 13090.95) |  | 2317311 (1958150 to 2773621) | 8393.25 (6908.82 to 10347.07) | | -1.3 (-1.65 to -0.95) |
| Andean Latin America | 2632200 (2037393 to 3368299) | 5459.03 (4308.73 to 6885.3) |  | 2688039 (2008863 to 3586171) | 4215.37 (3152.42 to 5621.36) | | -1 (-1.18 to -0.82) |
| Tropical Latin America | 10113866 (7724946 to 13447079) | 6003.22 (4699.42 to 7741.65) |  | 9896164 (7738019 to 12694179) | 4907.35 (3771.52 to 6383.32) | | -0.74 (-0.91 to -0.57) |
| Central Latin America | 7842460 (6237000 to 9867953) | 4394.09 (3669.97 to 5344.06) |  | 7921211 (6215577 to 10048175) | 3244.35 (2546.19 to 4160.4) | | -1.1 (-1.22 to -0.98) |
| Southern Latin America | 3277350 (2819037 to 3831780) | 6696.09 (5768.59 to 7777.84) |  | 4431281 (3786071 to 5243875) | 6450.18 (5427.78 to 7800.39) | | -0.15 (-0.25 to -0.04) |
| Caribbean | 2627837 (2201600 to 3160970) | 7109.69 (6078.72 to 8417.15) |  | 2736600 (2297598 to 3292308) | 6072.63 (5048.46 to 7345.09) | | -0.52 (-0.57 to -0.46) |
| Central Europe | 7584607 (6440124 to 8966701) | 5793.98 (4905.79 to 6911.38) |  | 5066122 (4218455 to 6051251) | 4203.57 (3452.76 to 5155.48) | | -1.2 (-1.34 to -1.06) |
| Eastern Europe | 11342552 (9588423 to 13401133) | 4728 (3978.76 to 5645.86) |  | 5363011 (4377809 to 6458925) | 2712.03 (2160.78 to 3412.64) | | -1.94 (-2.1 to -1.78) |
| Central Asia | 1729168 (1458761 to 2094774) | 2691.38 (2311.02 to 3149.1) |  | 2072070 (1692605 to 2559339) | 2277.44 (1883.33 to 2787.8) | | -0.57 (-0.73 to -0.41) |
| North Africa and Middle East | 13463264 (11334327 to 16249057) | 4145.9 (3633.93 to 4785.99) |  | 22132734 (18619787 to 26444040) | 3819.33 (3262.52 to 4512.65) | | -0.3 (-0.36 to -0.25) |
| South Asia | 24040433 (20356738 to 28785094) | 2741.23 (2334.07 to 3211.04) |  | 39871689 (33201510 to 47765389) | 2443.4 (2029.82 to 2909.76) | | -0.3 (-0.7 to 0.1) |
| Southeast Asia | 16716040 (14178709 to 20256894) | 3737.17 (3253.92 to 4370.19) |  | 22359243 (19257485 to 26371311) | 3431.82 (2926.72 to 4059.76) | | -0.31 (-0.44 to -0.18) |
| East Asia | 27119267 (21801409 to 34527343) | 2359.25 (1912.28 to 2950.44) |  | 26502559 (21606395 to 32719373) | 2025.52 (1577.43 to 2631.41) | | -0.31 (-0.9 to 0.28) |
| Oceania | 314116 (278533 to 358461) | 5449.75 (4929.29 to 6014.58) |  | 529213 (467646 to 599363) | 4265.16 (3834.97 to 4731.89) | | -0.83 (-0.89 to -0.77) |
| Western Sub-Saharan Africa | 6952062 (5732499 to 8617231) | 3770.29 (3254.1 to 4414.65) |  | 14052637 (11321219 to 17960481) | 3087.32 (2629.03 to 3669.71) | | -0.78 (-0.96 to -0.59) |
| Eastern Sub-Saharan Africa | 10200333 (8503358 to 12613584) | 5133.93 (4490.9 to 5952.11) |  | 17781817 (14422770 to 22250549) | 4151.16 (3582.13 to 4898.7) | | -0.71 (-0.77 to -0.66) |
| Central Sub-Saharan Africa | 2111203 (1737056 to 2594521) | 3664.87 (3189.07 to 4228.16) |  | 4117823 (3355827 to 5095414) | 3081.67 (2653.81 to 3633.35) | | -0.62 (-0.67 to -0.57) |
| Southern Sub-Saharan Africa | 2088211 (1464725 to 2665580) | 4135.84 (2966.16 to 5083.65) |  | 2662794 (1922876 to 3414012) | 3476.21 (2532.37 to 4396.58) | | -0.2 (-0.73 to 0.33) |
| **Countries** |  |  |  |  |  | |  |
| Afghanistan | 498371 (428705 to 582821) | 4740.1 (4210.69 to 5299.51) |  | 1515532 (1250584 to 1877887) | 4281.02 (3758.84 to 4909.04) | | -0.36 (-0.41 to -0.31) |
| Albania | 79535 (66440 to 98952) | 2613.12 (2252.14 to 3122.14) |  | 79785 (66537 to 96602) | 2828.38 (2319.68 to 3483.86) | | 0.12 (-0.12 to 0.36) |
| Algeria | 777903 (640644 to 976466) | 3171.04 (2723.35 to 3752.87) |  | 1311915 (1073558 to 1613359) | 3219.45 (2632.92 to 3923.89) | | 0.11 (0 to 0.22) |
| American Samoa | 1655 (1320 to 2109) | 3486.46 (2789.18 to 4263.99) |  | 1853 (1277 to 2351) | 3340.52 (2255.05 to 4204.59) | | 0.11 (-0.46 to 0.69) |
| Andorra | 3822 (3175 to 4579) | 6830.61 (5712.59 to 8190.86) |  | 5104 (4196 to 6161) | 5846.73 (4810.24 to 7128.01) | | -0.55 (-0.63 to -0.48) |
| Angola | 473612 (399474 to 576302) | 4472.68 (3958.66 to 5120.28) |  | 987366 (784259 to 1232058) | 3140.14 (2694.02 to 3682.62) | | -1.25 (-1.31 to -1.18) |
| Antigua and Barbuda | 3370 (2642 to 4341) | 5338.2 (4266.69 to 6817.64) |  | 4281 (3485 to 5300) | 5611.62 (4390.4 to 7155.64) | | 0.23 (0.08 to 0.39) |
| Argentina | 2401217 (2056469 to 2793030) | 7241.79 (6184.77 to 8404.3) |  | 3234945 (2748264 to 3848411) | 7019.77 (5893.61 to 8448.07) | | -0.16 (-0.23 to -0.1) |
| Armenia | 65275 (52617 to 81444) | 1951.76 (1590.37 to 2395.32) |  | 62216 (46975 to 76442) | 2093.13 (1628.91 to 2624.83) | | 0.28 (0.18 to 0.39) |
| Australia | 1926397 (1794473 to 2066600) | 11699.48 (10879.25 to 12543.42) |  | 2047081 (1720113 to 2448384) | 8768.43 (7162.3 to 10821.74) | | -1.02 (-1.39 to -0.65) |
| Austria | 618927 (525088 to 718198) | 7014.85 (5951.85 to 8272.91) |  | 489835 (405031 to 590357) | 5220.26 (4280.7 to 6380.52) | | -1.08 (-1.29 to -0.88) |
| Azerbaijan | 147618 (121631 to 182986) | 2114.05 (1788.84 to 2546.52) |  | 189910 (156275 to 234271) | 1961.21 (1608.59 to 2435.73) | | -0.19 (-0.4 to 0.01) |
| Bahamas | 14987 (12010 to 18587) | 5582.22 (4560.77 to 6869.15) |  | 19052 (15601 to 23533) | 5660.23 (4491.02 to 7161.64) | | 0.15 (0.01 to 0.29) |
| Bahrain | 19611 (16473 to 23653) | 4485.51 (3915.71 to 5195.32) |  | 46333 (38382 to 56216) | 3639.21 (2998.99 to 4452.81) | | -0.67 (-0.76 to -0.58) |
| Bangladesh | 1522763 (1338902 to 1747547) | 1855.79 (1667.22 to 2057.79) |  | 2073478 (1801991 to 2362800) | 1390.91 (1217.24 to 1574.2) | | -1.11 (-1.27 to -0.95) |
| Barbados | 16886 (14246 to 20256) | 6747.64 (5681.4 to 8152.07) |  | 17847 (14892 to 21186) | 6878.85 (5508.21 to 8515.22) | | -0.02 (-0.16 to 0.11) |
| Belarus | 601869 (498203 to 710929) | 5389.71 (4451.56 to 6393.18) |  | 328033 (271030 to 396927) | 3417.66 (2760.82 to 4292.83) | | -1.63 (-1.85 to -1.41) |
| Belgium | 838526 (729289 to 974390) | 7261.55 (6235.65 to 8543.23) |  | 542551 (456000 to 645456) | 4609.89 (3791.88 to 5606.24) | | -2.01 (-2.46 to -1.56) |
| Belize | 13506 (10851 to 17142) | 6011.39 (5038.07 to 7353.51) |  | 24260 (19296 to 30534) | 5657.5 (4549 to 7101.45) | | -0.19 (-0.42 to 0.04) |
| Benin | 149531 (123695 to 182224) | 3241.92 (2874.74 to 3694.52) |  | 345397 (275121 to 436254) | 2783.41 (2387.15 to 3274.67) | | -0.57 (-0.69 to -0.44) |
| Bermuda | 2956 (2410 to 3630) | 5534.53 (4390.98 to 6936.55) |  | 2713 (2226 to 3335) | 5842.79 (4499.49 to 7495.36) | | 0.3 (0.13 to 0.47) |
| Bhutan | 9816 (8567 to 11266) | 2022.18 (1811.26 to 2239.02) |  | 9763 (8608 to 11051) | 1429.78 (1272.34 to 1603.04) | | -1.25 (-1.39 to -1.11) |
| Bolivia (Plurinational State of) | 459655 (351462 to 590009) | 5363.51 (4229.61 to 6709.93) |  | 559829 (420623 to 732214) | 4250.69 (3250.05 to 5536.08) | | -0.96 (-1.24 to -0.69) |
| Bosnia and Herzegovina | 202586 (169114 to 245602) | 4597.21 (3865.68 to 5553.5) |  | 161181 (134157 to 192943) | 4466.42 (3656.72 to 5529.45) | | -0.1 (-0.18 to -0.02) |
| Botswana | 28782 (24204 to 34738) | 2497.09 (2199.84 to 2832.94) |  | 56626 (46319 to 68640) | 2572.75 (2165.53 to 3052.39) | | 0 (-0.16 to 0.15) |
| Brazil | 9894735 (7559903 to 13161901) | 6045.9 (4725.06 to 7807.75) |  | 9520211 (7444049 to 12180750) | 4892.43 (3757.28 to 6352.04) | | -0.78 (-0.94 to -0.61) |
| Brunei Darussalam | 11045 (9069 to 13801) | 5638.87 (4734.3 to 6942.58) |  | 17726 (14631 to 21814) | 4887.06 (4088.09 to 5908.93) | | -0.52 (-0.58 to -0.46) |
| Bulgaria | 395019 (328696 to 474831) | 4196.23 (3509.42 to 5086.58) |  | 260803 (213949 to 311776) | 3612.72 (2919.2 to 4427.34) | | -0.57 (-0.74 to -0.4) |
| Burkina Faso | 265313 (212800 to 336830) | 2595.47 (2228.41 to 3056.79) |  | 615592 (487310 to 799997) | 2586.12 (2178.5 to 3125.76) | | -0.03 (-0.14 to 0.08) |
| Burundi | 344827 (289564 to 413622) | 6006.62 (5296 to 6776.02) |  | 517185 (419705 to 636973) | 4142.85 (3585.48 to 4806.9) | | -1.28 (-1.41 to -1.15) |
| Cabo Verde | 10691 (8686 to 13395) | 3046.64 (2631.55 to 3583.54) |  | 13527 (11061 to 16913) | 2443.63 (2020.5 to 3024.72) | | -0.73 (-0.78 to -0.68) |
| Cambodia | 227575 (184185 to 283855) | 2172.79 (1884.4 to 2526.42) |  | 386095 (322587 to 474535) | 2403.29 (2034.03 to 2896.1) | | 0.39 (0.26 to 0.52) |
| Cameroon | 262619 (213036 to 328473) | 2694.41 (2310.95 to 3168.33) |  | 679955 (536381 to 880881) | 2325.79 (1937.92 to 2835.75) | | -0.51 (-0.62 to -0.39) |
| Canada | 1424421 (1181401 to 1715394) | 5369.62 (4412.68 to 6548.46) |  | 1650337 (1381363 to 1967955) | 4817.27 (3892.85 to 5970.94) | | -0.51 (-0.88 to -0.14) |
| Central African Republic | 118621 (100637 to 141253) | 4409.86 (3923.28 to 4972.66) |  | 183039 (152909 to 221751) | 3476.77 (3045.21 to 3965.72) | | -0.81 (-0.82 to -0.8) |
| Chad | 137722 (112144 to 172438) | 2320.1 (2009.53 to 2722.77) |  | 371908 (292030 to 479772) | 2161.69 (1838.47 to 2574.23) | | -0.28 (-0.37 to -0.19) |
| Chile | 654433 (558825 to 785780) | 5329.9 (4616.15 to 6309.12) |  | 990775 (847405 to 1160035) | 5196.37 (4391.45 to 6249.28) | | -0.07 (-0.31 to 0.17) |
| China | 25559015 (20406450 to 32633238) | 2296.63 (1852.1 to 2880.73) |  | 24766952 (20076066 to 30666151) | 1974.16 (1530.16 to 2565.37) | | -0.27 (-0.9 to 0.36) |
| Colombia | 1605880 (1267081 to 2032068) | 4826.13 (3977.63 to 5909.6) |  | 1551388 (1228472 to 1944968) | 3478.04 (2729.24 to 4428.09) | | -1.03 (-1.29 to -0.76) |
| Comoros | 23464 (19701 to 28536) | 4807.4 (4195.46 to 5604.7) |  | 29194 (24318 to 35464) | 4061.88 (3450.4 to 4841.62) | | -0.58 (-0.61 to -0.54) |
| Congo | 107611 (89540 to 130973) | 4496.14 (3934.29 to 5172.43) |  | 191764 (156525 to 236966) | 3717.56 (3167.36 to 4387.34) | | -0.67 (-0.8 to -0.54) |
| Cook Islands | 754 (563 to 970) | 3996.52 (2926.74 to 5014.24) |  | 638 (422 to 786) | 3582.17 (2502.86 to 4533.59) | | -0.24 (-0.51 to 0.04) |
| Costa Rica | 220713 (177445 to 271958) | 6971.38 (5804.34 to 8480.04) |  | 246652 (200613 to 304694) | 5529.27 (4443.27 to 6905.54) | | -0.84 (-0.97 to -0.7) |
| Croatia | 285241 (232914 to 342261) | 5327.11 (4355.52 to 6448.66) |  | 188078 (158810 to 223334) | 4018.57 (3316.2 to 4929.38) | | -1.1 (-1.31 to -0.89) |
| Cuba | 808383 (661439 to 988727) | 7590.62 (6224.79 to 9387.2) |  | 653775 (535739 to 791660) | 6979.37 (5504.92 to 8790.42) | | -0.28 (-0.32 to -0.23) |
| Cyprus | 60638 (48931 to 73868) | 7624.9 (6154.05 to 9289.55) |  | 99525 (80592 to 120953) | 7088.53 (5756.88 to 8584.47) | | -0.26 (-0.33 to -0.18) |
| Czechia | 328324 (275297 to 398492) | 3146.19 (2596.81 to 3844.58) |  | 327052 (269708 to 392256) | 3053.07 (2479.86 to 3781.11) | | -0.19 (-0.42 to 0.05) |
| Côte d’Ivoire | 394613 (324191 to 487962) | 3450.94 (2993.9 to 4035.39) |  | 805617 (658270 to 1010962) | 3154.29 (2690.66 to 3760.81) | | -0.22 (-0.36 to -0.09) |
| Democratic People's Republic of Korea | 763486 (628964 to 963121) | 3852.07 (3195.86 to 4916.22) |  | 998398 (880328 to 1140797) | 3704.71 (3216.47 to 4372.58) | | -0.2 (-0.38 to -0.02) |
| Democratic Republic of the Congo | 1360068 (1097868 to 1692027) | 3342.79 (2865.73 to 3903.78) |  | 2664393 (2183112 to 3293446) | 3003.45 (2582.79 to 3545.38) | | -0.38 (-0.44 to -0.32) |
| Denmark | 359686 (309203 to 417605) | 6288.16 (5436.02 to 7394.62) |  | 282375 (236946 to 337040) | 4782.68 (3910.04 to 5846.14) | | -1 (-1.37 to -0.63) |
| Djibouti | 26428 (21824 to 32455) | 5245.52 (4563.13 to 6057) |  | 48835 (40330 to 59712) | 4132.74 (3519.31 to 4912.78) | | -0.8 (-0.89 to -0.72) |
| Dominica | 5180 (4298 to 6332) | 6708.59 (5684.05 to 8026.61) |  | 4327 (3729 to 5082) | 6834.37 (5762.73 to 8245.25) | | 0.08 (0.02 to 0.14) |
| Dominican Republic | 328856 (267105 to 407417) | 3933.48 (3275.5 to 4743.56) |  | 420153 (335178 to 527757) | 3858.57 (3093.28 to 4851.15) | | 0 (-0.29 to 0.3) |
| Ecuador | 640099 (479397 to 830020) | 5063.61 (3886 to 6475.25) |  | 703958 (523152 to 942678) | 3966.97 (2945.08 to 5332.17) | | -0.81 (-1.02 to -0.6) |
| Egypt | 2023077 (1676916 to 2471493) | 3697.98 (3170.86 to 4332.3) |  | 3347076 (2743236 to 4121162) | 3486.8 (2903.16 to 4200.21) | | -0.19 (-0.24 to -0.14) |
| El Salvador | 524413 (400755 to 667852) | 8741.65 (6849.07 to 10792.19) |  | 337603 (272860 to 419278) | 5419.43 (4395.27 to 6674.38) | | -1.72 (-1.9 to -1.53) |
| Equatorial Guinea | 17470 (14647 to 21190) | 4004.75 (3518.47 to 4559.11) |  | 43537 (33747 to 55835) | 3027.96 (2529.24 to 3664.17) | | -0.94 (-1.02 to -0.86) |
| Eritrea | 161786 (135704 to 194610) | 5360.57 (4737.84 to 6074.41) |  | 294185 (245719 to 357430) | 4403.82 (3837.02 to 5096.06) | | -0.71 (-0.76 to -0.66) |
| Estonia | 45504 (39891 to 51539) | 2800.52 (2426.71 to 3210.7) |  | 26128 (21891 to 30890) | 2075.2 (1663.82 to 2580.6) | | -1.04 (-1.19 to -0.9) |
| Eswatini | 34986 (29498 to 42264) | 5161.17 (4529.13 to 5816.13) |  | 46774 (40350 to 54756) | 4646.92 (4078.1 to 5220.68) | | -0.42 (-0.5 to -0.33) |
| Ethiopia | 1989811 (1603190 to 2509503) | 3780.55 (3205.88 to 4489.35) |  | 3249237 (2524664 to 4200802) | 2791.61 (2299.35 to 3414.23) | | -0.98 (-1.08 to -0.88) |
| Fiji | 31450 (27142 to 36539) | 4795.31 (4209.92 to 5431.4) |  | 29196 (25128 to 34144) | 3289.09 (2847.38 to 3816.31) | | -1.3 (-1.4 to -1.2) |
| Finland | 363900 (310887 to 422891) | 6716.65 (5669.76 to 7866.09) |  | 382167 (324212 to 446291) | 6102.5 (5043.17 to 7355.66) | | -0.37 (-0.56 to -0.18) |
| France | 6252763 (5566864 to 7109420) | 9667.56 (8521.8 to 11260) |  | 4605053 (3858024 to 5438405) | 6555.19 (5357.38 to 7932.96) | | -1.68 (-1.97 to -1.39) |
| Gabon | 33822 (28053 to 41413) | 3454.6 (2986.13 to 4055.44) |  | 47724 (38513 to 60950) | 2736.53 (2258.42 to 3395.03) | | -0.79 (-0.89 to -0.7) |
| Gambia | 29420 (23910 to 36444) | 3217.6 (2822.5 to 3745.44) |  | 60978 (49112 to 76401) | 2791.54 (2399.96 to 3300.47) | | -0.51 (-0.6 to -0.43) |
| Georgia | 115958 (99783 to 136629) | 2093.3 (1799.08 to 2487.86) |  | 69582 (56805 to 85459) | 1958.92 (1572.51 to 2475.31) | | -0.23 (-0.51 to 0.05) |
| Germany | 6650125 (5702966 to 7623160) | 7013.11 (6073.46 to 8135.45) |  | 3609452 (3014106 to 4299203) | 4153.11 (3405.37 to 5081.31) | | -1.88 (-2.39 to -1.36) |
| Ghana | 314077 (250560 to 395456) | 2037.4 (1731.91 to 2412.07) |  | 667512 (541608 to 840813) | 2118.82 (1785.69 to 2580.9) | | 0.14 (0.03 to 0.26) |
| Greece | 690826 (577397 to 825221) | 5885.14 (4858.42 to 7170.64) |  | 591070 (487644 to 712681) | 5301.47 (4326.18 to 6546.28) | | -0.37 (-0.42 to -0.31) |
| Greenland | 5034 (4016 to 5984) | 10231.08 (8081.11 to 12146.18) |  | 3736 (3272 to 4293) | 6702.35 (5767.29 to 7927.96) | | -1.63 (-1.75 to -1.5) |
| Grenada | 6601 (5224 to 8351) | 7094.83 (5720.49 to 8789.49) |  | 6768 (5434 to 8402) | 7097.17 (5663.9 to 8994.22) | | -0.01 (-0.04 to 0.02) |
| Guam | 5265 (3872 to 6709) | 3991.59 (2848.07 to 5007.12) |  | 5680 (3905 to 7189) | 3380.03 (2381.97 to 4343.26) | | -0.46 (-0.79 to -0.13) |
| Guatemala | 529027 (427158 to 658830) | 5135.94 (4304.93 to 6155.73) |  | 547912 (420001 to 720467) | 2865.85 (2258.38 to 3708.11) | | -2.06 (-2.19 to -1.92) |
| Guinea | 224458 (183775 to 275830) | 3690.27 (3154.77 to 4346.61) |  | 422769 (336844 to 536671) | 3372.99 (2835.33 to 3999.13) | | -0.39 (-0.51 to -0.28) |
| Guinea-Bissau | 35844 (30228 to 43000) | 3917.06 (3485.23 to 4395.86) |  | 56299 (46912 to 68780) | 3123.22 (2760.66 to 3618.12) | | -0.86 (-0.98 to -0.75) |
| Guyana | 55001 (45327 to 67075) | 6701.1 (5741.22 to 7946.06) |  | 48079 (39843 to 58263) | 6206.78 (5127.47 to 7539.04) | | -0.28 (-0.38 to -0.19) |
| Haiti | 616839 (513398 to 746778) | 8218.27 (7092.08 to 9611.38) |  | 944674 (796000 to 1137376) | 7086.45 (6059.95 to 8372.99) | | -0.54 (-0.59 to -0.49) |
| Honduras | 338107 (271543 to 422419) | 5433.45 (4551.69 to 6564.12) |  | 404374 (317884 to 517982) | 3778.05 (3053.69 to 4714.63) | | -1.27 (-1.39 to -1.15) |
| Hungary | 447614 (382530 to 522251) | 4067.31 (3435.01 to 4817.84) |  | 321229 (265614 to 385390) | 3307.09 (2677.6 to 4083.25) | | -0.75 (-0.93 to -0.56) |
| Iceland | 24412 (20629 to 28822) | 9533.07 (8020.66 to 11268.75) |  | 25584 (21169 to 30771) | 7200.09 (5844.36 to 8849.12) | | -1.14 (-1.55 to -0.72) |
| India | 20333262 (17097406 to 24464734) | 2970.59 (2519.45 to 3502.25) |  | 34305973 (27950714 to 41323723) | 2680.88 (2189.55 to 3221.55) | | -0.21 (-0.76 to 0.34) |
| Indonesia | 6702563 (5644399 to 8162426) | 3811.87 (3282.97 to 4515.18) |  | 8673884 (7367856 to 10270736) | 3431.25 (2918.28 to 4055.37) | | -0.51 (-0.71 to -0.31) |
| Iran (Islamic Republic of) | 2298639 (1871634 to 2861152) | 4235.23 (3602.37 to 5011.99) |  | 2635573 (2182430 to 3205594) | 3280.14 (2717.29 to 3978.44) | | -0.96 (-1.09 to -0.83) |
| Iraq | 818213 (685131 to 1010795) | 5006.8 (4413.91 to 5753.84) |  | 1451876 (1169492 to 1834319) | 3446.54 (2875.1 to 4218.2) | | -1.29 (-1.35 to -1.24) |
| Ireland | 376823 (323905 to 434076) | 10213.71 (8764.93 to 11834.99) |  | 343380 (288588 to 410323) | 6860.25 (5695.04 to 8348.21) | | -1.47 (-2 to -0.95) |
| Israel | 324289 (271489 to 382244) | 6611.94 (5557.39 to 7744.74) |  | 465362 (392157 to 560172) | 4958.02 (4134.53 to 6019.91) | | -1.09 (-1.28 to -0.9) |
| Italy | 3953862 (3330334 to 4689031) | 5629.27 (4734.78 to 6690.8) |  | 2561074 (2052642 to 3143467) | 4027.89 (3173.84 to 5106.71) | | -1.05 (-1.63 to -0.47) |
| Jamaica | 175081 (142442 to 218619) | 6714.03 (5616.66 to 8214.99) |  | 146411 (117836 to 181759) | 5691.56 (4485.71 to 7172.32) | | -0.55 (-0.75 to -0.35) |
| Japan | 11246046 (9650457 to 12976284) | 8192.5 (6971.18 to 9598.36) |  | 5117128 (4314187 to 6091159) | 3865.42 (3118.67 to 4890.82) | | -2.74 (-2.95 to -2.53) |
| Jordan | 155571 (121602 to 199269) | 4273.34 (3560.58 to 5114.41) |  | 470579 (373465 to 600638) | 4044.69 (3266.12 to 5037.91) | | -0.12 (-0.24 to 0.01) |
| Kazakhstan | 251154 (208289 to 304227) | 1600.12 (1341.65 to 1907.62) |  | 286976 (232367 to 356099) | 1590.71 (1282.74 to 1958.23) | | 0.17 (-0.1 to 0.43) |
| Kenya | 775052 (610440 to 1000263) | 3221.24 (2698.51 to 3856.04) |  | 1418420 (1122230 to 1798200) | 2730.22 (2259.15 to 3321.32) | | -0.51 (-0.58 to -0.45) |
| Kiribati | 4574 (4159 to 5041) | 6715.19 (6192.73 to 7250.43) |  | 5277 (4787 to 5861) | 4759.28 (4377.4 to 5201.51) | | -1.18 (-1.22 to -1.14) |
| Kuwait | 74041 (59756 to 91927) | 4470.85 (3734.58 to 5337.94) |  | 174109 (138549 to 221831) | 4174.46 (3290.55 to 5265.12) | | -0.34 (-0.44 to -0.23) |
| Kyrgyzstan | 140899 (117605 to 172070) | 3441.9 (2937.41 to 4035.55) |  | 156012 (124978 to 197291) | 2417.2 (1960.71 to 3005.35) | | -1.34 (-1.48 to -1.2) |
| Lao People's Democratic Republic | 103986 (87489 to 126244) | 2401.57 (2100.24 to 2774) |  | 147309 (126746 to 175671) | 2140.92 (1867.95 to 2502.13) | | -0.49 (-0.64 to -0.34) |
| Latvia | 142340 (123131 to 163443) | 4930.2 (4268.5 to 5758.8) |  | 60696 (51539 to 72358) | 3184.73 (2584.81 to 4052.73) | | -1.46 (-1.53 to -1.4) |
| Lebanon | 143800 (119375 to 172411) | 4514.71 (3828.82 to 5299.21) |  | 227323 (186845 to 274256) | 4443.27 (3641.77 to 5385.77) | | -0.08 (-0.17 to 0.02) |
| Lesotho | 22889 (18971 to 27418) | 1341.01 (1162.03 to 1530.05) |  | 27208 (23277 to 31480) | 1377.07 (1185.73 to 1567.73) | | 0.14 (0.08 to 0.2) |
| Liberia | 59316 (48522 to 74831) | 2714.1 (2351.71 to 3235.39) |  | 115412 (91514 to 147962) | 2334.41 (1955.58 to 2853) | | -0.55 (-0.67 to -0.43) |
| Libya | 167769 (136990 to 209553) | 4259.24 (3718.16 to 4984.94) |  | 235519 (199456 to 282297) | 3725.34 (3122.59 to 4497.03) | | -0.51 (-0.64 to -0.39) |
| Lithuania | 126760 (107839 to 147317) | 3288.95 (2775.81 to 3878.73) |  | 73987 (61789 to 88189) | 2718.44 (2191.37 to 3363.57) | | -0.61 (-0.77 to -0.45) |
| Luxembourg | 38739 (32544 to 49713) | 9298.17 (7741.43 to 11642.58) |  | 44283 (36990 to 52364) | 6772.13 (5585.75 to 8149.6) | | -1.15 (-1.32 to -0.99) |
| Madagascar | 1321024 (1136958 to 1544897) | 9951.56 (8841.92 to 11236.94) |  | 1735901 (1490585 to 2034248) | 6355.94 (5633.56 to 7165.78) | | -1.62 (-1.83 to -1.41) |
| Malawi | 412315 (330979 to 519276) | 4023.52 (3426 to 4706) |  | 719451 (565657 to 901924) | 3707.18 (3090.3 to 4431.57) | | -0.23 (-0.32 to -0.15) |
| Malaysia | 531064 (460610 to 622801) | 3348.11 (2966.97 to 3795.64) |  | 792596 (662895 to 969016) | 2624.7 (2178.25 to 3253.87) | | -0.85 (-1.1 to -0.6) |
| Maldives | 8980 (7529 to 10820) | 3624.4 (3167.89 to 4154.63) |  | 11018 (8811 to 13955) | 2422.72 (1927.16 to 3083.75) | | -1.43 (-1.7 to -1.15) |
| Mali | 180948 (148099 to 227712) | 2128.29 (1835.48 to 2502.46) |  | 480575 (382960 to 628685) | 2140.49 (1829 to 2567.68) | | 0.05 (-0.07 to 0.18) |
| Malta | 35647 (30501 to 41969) | 9026.04 (7735.24 to 10612.02) |  | 35018 (29624 to 41788) | 7075.87 (5876.26 to 8569.64) | | -0.91 (-1.01 to -0.82) |
| Marshall Islands | 1647 (1393 to 1985) | 3894.18 (3513.52 to 4360.85) |  | 1682 (1383 to 2062) | 3015.2 (2495.91 to 3682.02) | | -0.76 (-0.98 to -0.53) |
| Mauritania | 105882 (86145 to 128861) | 5666.62 (4812.32 to 6590.38) |  | 180653 (147063 to 225869) | 4687.13 (4033.79 to 5506.4) | | -0.69 (-0.76 to -0.63) |
| Mauritius | 42204 (36763 to 49125) | 4222.42 (3732.13 to 4835.53) |  | 33596 (29238 to 39278) | 2774.03 (2334.5 to 3376.84) | | -1.45 (-1.54 to -1.37) |
| Mexico | 3193956 (2488868 to 4169496) | 3534.94 (2901.91 to 4376.13) |  | 3353355 (2591663 to 4377729) | 2765.09 (2128.83 to 3625.99) | | -0.93 (-1.14 to -0.73) |
| Micronesia (Federated States of) | 3764 (3246 to 4444) | 3883.06 (3497.07 to 4330.61) |  | 3140 (2583 to 3836) | 3144.08 (2602.21 to 3828.47) | | -0.54 (-0.79 to -0.3) |
| Monaco | 2034 (1721 to 2418) | 6174.48 (5087.98 to 7532.26) |  | 2407 (2012 to 2864) | 6014.03 (4939.68 to 7271.8) | | -0.07 (-0.12 to -0.02) |
| Mongolia | 47623 (38949 to 58904) | 2609.96 (2257.19 to 3036.67) |  | 67114 (55071 to 82906) | 2114.21 (1759.6 to 2568.47) | | -0.71 (-0.76 to -0.67) |
| Montenegro | 20387 (16549 to 25645) | 3256.97 (2650.2 to 4102.26) |  | 22896 (18278 to 28178) | 3620.93 (2883.62 to 4557.22) | | 0.45 (0.3 to 0.6) |
| Morocco | 665780 (550850 to 810008) | 2675.66 (2312.12 to 3124.72) |  | 1020288 (859393 to 1228507) | 2914.58 (2452.51 to 3485.52) | | 0.29 (0.23 to 0.34) |
| Mozambique | 622611 (515046 to 768083) | 4301.37 (3745.85 to 5011.2) |  | 1329709 (1075478 to 1671938) | 4312.89 (3736.06 to 5066.18) | | 0 (-0.03 to 0.02) |
| Myanmar | 843885 (731074 to 992833) | 2178.29 (1935.93 to 2475.64) |  | 1065997 (933366 to 1212653) | 2016.24 (1770.17 to 2293.06) | | -0.31 (-0.41 to -0.22) |
| Namibia | 31445 (26714 to 37669) | 2489.33 (2191.25 to 2850.52) |  | 47132 (39200 to 57783) | 2096.89 (1800.72 to 2468.94) | | -0.61 (-0.68 to -0.53) |
| Nauru | 345 (287 to 434) | 3479.47 (2998.37 to 4127.4) |  | 340 (261 to 452) | 3289.83 (2534.29 to 4239.2) | | 0.07 (-0.3 to 0.44) |
| Nepal | 191969 (168075 to 220532) | 1254.04 (1117.27 to 1405.47) |  | 289923 (249851 to 332070) | 1072.46 (932.39 to 1214.78) | | -0.52 (-0.81 to -0.23) |
| Netherlands | 1317147 (1051808 to 1607954) | 8134.51 (6444.96 to 10065.64) |  | 1328832 (1109852 to 1597936) | 6941.99 (5702.57 to 8418.9) | | -0.48 (-0.72 to -0.23) |
| New Zealand | 490464 (409341 to 591110) | 14102.17 (11736.51 to 17001.42) |  | 270231 (226570 to 327195) | 6325.87 (5156.98 to 7871.32) | | -2.81 (-2.97 to -2.64) |
| Nicaragua | 288605 (222270 to 367502) | 5784.44 (4734.52 to 7081.98) |  | 255251 (199176 to 328146) | 3790.58 (3008.67 to 4812.98) | | -1.5 (-1.64 to -1.37) |
| Niger | 273989 (227372 to 340374) | 3410.18 (3015.83 to 3942.99) |  | 654110 (519223 to 840418) | 2713.05 (2339 to 3202.37) | | -0.86 (-0.99 to -0.72) |
| Nigeria | 4054612 (3334023 to 5015411) | 4727.22 (4045.73 to 5589.32) |  | 7720070 (6110405 to 9942614) | 3626.95 (3065.9 to 4365.72) | | -1.1 (-1.39 to -0.82) |
| Niue | 86 (68 to 110) | 3569.06 (2794.22 to 4479.16) |  | 59 (39 to 73) | 3507.34 (2425.36 to 4402.84) | | 0.12 (-0.21 to 0.45) |
| North Macedonia | 135837 (105001 to 167770) | 7017.14 (5386.63 to 8674.96) |  | 123320 (105483 to 145172) | 5199.79 (4376.14 to 6200.46) | | -1.11 (-1.27 to -0.94) |
| Northern Mariana Islands | 1662 (1300 to 2101) | 4054.57 (3137.65 to 5061.41) |  | 1414 (940 to 1750) | 3574.83 (2623.32 to 4576.91) | | -0.33 (-0.75 to 0.09) |
| Norway | 568647 (489979 to 666025) | 11310.76 (9649.84 to 13320.01) |  | 372463 (309274 to 444225) | 6790.15 (5589.85 to 8270.13) | | -1.73 (-1.92 to -1.55) |
| Oman | 60553 (48051 to 78425) | 3084.05 (2504.79 to 3817.47) |  | 172075 (136810 to 218229) | 4053.95 (3279.06 to 5014.45) | | 1.06 (0.86 to 1.26) |
| Pakistan | 1982623 (1669903 to 2383033) | 2133.26 (1825.96 to 2475.09) |  | 3192552 (2659807 to 3848337) | 1702.08 (1448.11 to 1974.78) | | -0.6 (-0.79 to -0.4) |
| Palau | 601 (498 to 723) | 4096.5 (3422.99 to 4835.41) |  | 573 (460 to 676) | 3321.08 (2719.29 to 4038.01) | | -0.63 (-0.87 to -0.38) |
| Palestine | 72287 (57157 to 93317) | 3654.66 (3083.74 to 4340.73) |  | 167759 (130859 to 216935) | 3332.38 (2715.94 to 4088.46) | | -0.27 (-0.34 to -0.21) |
| Panama | 142097 (116904 to 175401) | 5396.86 (4514.54 to 6576.13) |  | 182693 (145350 to 229024) | 4431.83 (3509.89 to 5574.74) | | -0.69 (-0.8 to -0.57) |
| Papua New Guinea | 217931 (193532 to 247825) | 6044.03 (5460.11 to 6634.91) |  | 412884 (365292 to 465899) | 4585.56 (4143.28 to 5043.65) | | -0.96 (-1.01 to -0.92) |
| Paraguay | 219131 (170124 to 288666) | 4583.57 (3700.19 to 5780.54) |  | 375953 (293749 to 490425) | 5408.15 (4227.82 to 7017.87) | | 0.58 (0.53 to 0.62) |
| Peru | 1532446 (1192069 to 1956088) | 5669.91 (4486.72 to 7165.46) |  | 1424251 (1057717 to 1889703) | 4335.62 (3189.83 to 5773.3) | | -1.01 (-1.13 to -0.89) |
| Philippines | 3950818 (3223138 to 4989881) | 5870.1 (4962.4 to 7115.66) |  | 5218948 (4372997 to 6402826) | 4628.31 (3916.19 to 5595.11) | | -0.86 (-0.91 to -0.82) |
| Poland | 3982472 (3319829 to 4735834) | 9777.56 (8170.35 to 11707.33) |  | 2214693 (1848570 to 2654560) | 5412.72 (4413.97 to 6639.11) | | -2.16 (-2.3 to -2.02) |
| Portugal | 1261598 (1053218 to 1506637) | 11168.28 (9284.43 to 13524) |  | 1104294 (925175 to 1307774) | 9106.18 (7499.66 to 11069.6) | | -1.21 (-1.77 to -0.65) |
| Puerto Rico | 389421 (326050 to 461383) | 10828.83 (9074.55 to 12841.4) |  | 243455 (202296 to 292121) | 8571.06 (6736.88 to 10699.94) | | -0.85 (-1.07 to -0.64) |
| Qatar | 14086 (11479 to 17566) | 3410.6 (2829.31 to 4117.75) |  | 84800 (67146 to 108245) | 3460.53 (2793.84 to 4306.11) | | 0.08 (0.02 to 0.13) |
| Republic of Korea | 1673792 (1416284 to 2002798) | 4491.3 (3824.36 to 5330.33) |  | 1915846 (1611521 to 2324501) | 3608.56 (2959.47 to 4514.64) | | -0.82 (-1.06 to -0.59) |
| Republic of Moldova | 162782 (140214 to 193737) | 3628.57 (3119.35 to 4341.83) |  | 98058 (78493 to 119716) | 2750.93 (2200.51 to 3503.81) | | -0.97 (-1.07 to -0.87) |
| Romania | 1116656 (913031 to 1341825) | 4547.38 (3707.62 to 5545.12) |  | 844316 (694482 to 1015131) | 4179.67 (3402.26 to 5180.64) | | -0.23 (-0.39 to -0.07) |
| Russian Federation | 7745720 (6534273 to 9187026) | 4811.5 (4055.71 to 5734.01) |  | 3507708 (2866450 to 4254388) | 2520.13 (2004.47 to 3174.17) | | -2.26 (-2.41 to -2.12) |
| Rwanda | 869272 (723189 to 1040145) | 12041.83 (10348.66 to 13779.87) |  | 1013743 (843675 to 1238599) | 8203.91 (7081.25 to 9665.99) | | -1.36 (-1.65 to -1.08) |
| Saint Kitts and Nevis | 2662 (2124 to 3364) | 6109.12 (5012.31 to 7552.67) |  | 2897 (2322 to 3582) | 5647.33 (4364.73 to 7149.5) | | -0.28 (-0.41 to -0.15) |
| Saint Lucia | 10661 (8565 to 13294) | 7234.42 (6046.95 to 8719.05) |  | 10518 (8966 to 12360) | 6751.58 (5593.62 to 8238.9) | | -0.26 (-0.34 to -0.17) |
| Saint Vincent and the Grenadines | 7150 (5696 to 9035) | 5883.44 (4840.7 to 7248.07) |  | 5879 (4873 to 7271) | 5703.08 (4605.61 to 7224.47) | | -0.05 (-0.14 to 0.03) |
| Samoa | 5476 (4503 to 6713) | 3535.46 (3027.18 to 4102.23) |  | 6241 (5021 to 7810) | 2953.1 (2413.03 to 3599.31) | | -0.52 (-0.63 to -0.4) |
| San Marino | 1443 (1198 to 1719) | 5894.45 (4867.27 to 7102.57) |  | 1959 (1615 to 2369) | 5759.19 (4728.01 to 7134.22) | | -0.05 (-0.09 to -0.01) |
| Sao Tome and Principe | 6003 (5012 to 7137) | 4980.73 (4394.4 to 5641.56) |  | 8041 (6908 to 9563) | 4253.65 (3782.33 to 4849.6) | | -0.67 (-0.77 to -0.56) |
| Saudi Arabia | 344119 (275108 to 439248) | 2092.99 (1765.37 to 2507.05) |  | 853996 (705952 to 1027815) | 2642.17 (2154.91 to 3207.34) | | 0.6 (0.3 to 0.91) |
| Senegal | 186394 (153563 to 232840) | 2517.06 (2183.9 to 2939.24) |  | 348822 (278481 to 440631) | 2318.17 (1960.09 to 2779.97) | | -0.3 (-0.38 to -0.22) |
| Serbia | 305521 (264298 to 356220) | 3180.46 (2729.91 to 3769.3) |  | 260377 (224080 to 303471) | 2849.13 (2358.05 to 3452.56) | | -0.44 (-0.54 to -0.33) |
| Seychelles | 1785 (1492 to 2188) | 2476.52 (2120.59 to 2959.51) |  | 2394 (2041 to 2850) | 2475.75 (2052.28 to 3052.36) | | 0.07 (-0.01 to 0.15) |
| Sierra Leone | 117406 (98452 to 141760) | 3188.27 (2825.33 to 3660.66) |  | 229112 (187284 to 285128) | 2768.06 (2393.95 to 3284.09) | | -0.54 (-0.71 to -0.37) |
| Singapore | 188894 (156202 to 220536) | 6704.47 (5653.4 to 7742.12) |  | 199395 (165089 to 242229) | 3667.15 (3012.13 to 4564.72) | | -2.1 (-2.21 to -1.99) |
| Slovakia | 159981 (133542 to 192933) | 2996.21 (2487.21 to 3645.75) |  | 159787 (131167 to 192792) | 2914.98 (2363.25 to 3588.8) | | -0.14 (-0.31 to 0.04) |
| Slovenia | 125435 (106300 to 150286) | 5944.55 (5013.1 to 7137.35) |  | 102604 (84762 to 123241) | 4591.92 (3730.8 to 5677.77) | | -0.87 (-0.95 to -0.79) |
| Solomon Islands | 12059 (10102 to 14652) | 3903.98 (3445.61 to 4493.34) |  | 22074 (17931 to 27939) | 3471.97 (2872 to 4273.75) | | -0.19 (-0.47 to 0.09) |
| Somalia | 428873 (360946 to 524617) | 5833.37 (5168.39 to 6666.08) |  | 917608 (755095 to 1135566) | 4452.97 (3914.46 to 5133.05) | | -0.92 (-0.94 to -0.9) |
| South Africa | 1796364 (1181000 to 2313975) | 4970.22 (3355.21 to 6224.02) |  | 2219861 (1485832 to 2910877) | 4123.97 (2787.98 to 5399.18) | | -0.13 (-0.78 to 0.52) |
| South Sudan | 295384 (244982 to 362764) | 4839.06 (4210.63 to 5632.84) |  | 389615 (313999 to 490360) | 3963.92 (3389.98 to 4680.27) | | -0.65 (-0.72 to -0.58) |
| Spain | 2029684 (1721962 to 2420902) | 4757.39 (3990.13 to 5809.48) |  | 2235478 (1869134 to 2664775) | 4481.25 (3636.51 to 5512.17) | | -0.32 (-0.48 to -0.17) |
| Sri Lanka | 537049 (470011 to 619236) | 3611.24 (3208.86 to 4041.89) |  | 799526 (705682 to 914751) | 3541.69 (3091.39 to 4114.25) | | -0.16 (-0.27 to -0.06) |
| Sudan | 843168 (682566 to 1031844) | 4326.31 (3687.08 to 5084.28) |  | 1626705 (1297906 to 2051810) | 4174.67 (3493.15 to 4976.25) | | -0.15 (-0.2 to -0.1) |
| Suriname | 23142 (18871 to 28887) | 5557.84 (4633.21 to 6790.95) |  | 29417 (23781 to 36861) | 5360.3 (4298 to 6764.45) | | -0.14 (-0.26 to -0.01) |
| Sweden | 1190422 (999986 to 1417070) | 11444.22 (9558.65 to 13661.75) |  | 824426 (669881 to 1009469) | 7586.11 (6046.56 to 9402.98) | | -1.45 (-1.7 to -1.2) |
| Switzerland | 643531 (548930 to 753372) | 8440.95 (7133.54 to 9998.17) |  | 608821 (507686 to 733943) | 6615.85 (5412.6 to 8052.13) | | -0.83 (-0.92 to -0.75) |
| Syrian Arab Republic | 430804 (356993 to 525828) | 3348.28 (2925.57 to 3861.47) |  | 469399 (397789 to 559453) | 3268.89 (2774.54 to 3868.76) | | -0.19 (-0.46 to 0.09) |
| Taiwan (Province of China) | 796766 (670325 to 980824) | 4235.82 (3579.83 to 5155.33) |  | 737209 (628778 to 879469) | 3385.72 (2740.55 to 4296.11) | | -1.2 (-1.69 to -0.7) |
| Tajikistan | 122501 (98602 to 155195) | 2610.93 (2233.73 to 3065.59) |  | 194584 (154930 to 248712) | 2264.74 (1862.91 to 2762.31) | | -0.46 (-0.57 to -0.35) |
| Thailand | 2009331 (1734418 to 2354719) | 3917.91 (3442.68 to 4486) |  | 2309631 (2005712 to 2688542) | 3411.53 (2863.7 to 4141.62) | | -0.67 (-0.9 to -0.44) |
| Timor-Leste | 26325 (21879 to 31618) | 3155.12 (2752.16 to 3619.85) |  | 39729 (33710 to 47061) | 2904.97 (2544.44 to 3332.76) | | -0.33 (-0.42 to -0.24) |
| Togo | 142993 (115812 to 178027) | 4210.07 (3608.03 to 4910.99) |  | 276092 (225766 to 346222) | 3616.65 (3068.84 to 4309.65) | | -0.49 (-0.73 to -0.24) |
| Tokelau | 59 (45 to 81) | 3385.19 (2567.81 to 4566.16) |  | 50 (35 to 64) | 3426.61 (2381.94 to 4338.56) | | 0.25 (-0.11 to 0.6) |
| Tonga | 4217 (3429 to 5331) | 4503.5 (3827.75 to 5376.12) |  | 3798 (3042 to 4782) | 3661.38 (2980.68 to 4537.51) | | -0.51 (-0.78 to -0.25) |
| Trinidad and Tobago | 54227 (43661 to 67449) | 4189.31 (3457.37 to 5149.25) |  | 54667 (43023 to 68100) | 4631.31 (3578.78 to 5962.65) | | 0.68 (0.39 to 0.96) |
| Tunisia | 290736 (241636 to 356963) | 3603.34 (3107.95 to 4296.02) |  | 412522 (348082 to 491379) | 3640.19 (3020.49 to 4393.76) | | 0.04 (0 to 0.08) |
| Turkey | 3029326 (2586991 to 3670400) | 5503.39 (4813.36 to 6430.52) |  | 4082370 (3517474 to 4743627) | 5102.54 (4401.62 to 6040.67) | | -0.21 (-0.41 to -0.01) |
| Turkmenistan | 100661 (84042 to 122848) | 2936.57 (2566.79 to 3387.63) |  | 101614 (78999 to 129040) | 2011.28 (1553.21 to 2532.4) | | -1.28 (-1.59 to -0.98) |
| Tuvalu | 286 (243 to 342) | 3107.53 (2681.79 to 3649.81) |  | 396 (272 to 504) | 3395.45 (2319.94 to 4292.1) | | 0.43 (-0.13 to 1) |
| Uganda | 1060939 (864545 to 1333995) | 5822.26 (5049.92 to 6843.37) |  | 2136099 (1682331 to 2750153) | 4952.62 (4216.71 to 5881.59) | | -0.6 (-0.68 to -0.52) |
| Ukraine | 2517578 (2098292 to 3020646) | 4703.49 (3848.75 to 5741.11) |  | 1268399 (1019017 to 1553151) | 3227.13 (2528.4 to 4076.43) | | -1.23 (-1.49 to -0.98) |
| United Arab Emirates | 126457 (105116 to 150913) | 8100.42 (6883.34 to 9295.82) |  | 614690 (514498 to 719007) | 7179.91 (6188.25 to 8345.37) | | -0.72 (-1.32 to -0.12) |
| United Kingdom | 8667759 (7453177 to 10051051) | 13998.59 (11939.17 to 16447.44) |  | 6459573 (5468359 to 7563834) | 9166.57 (7645.04 to 11034.51) | | -1.67 (-2.07 to -1.27) |
| United Republic of Tanzania | 1661671 (1340860 to 2072273) | 6189.98 (5266.16 to 7300.72) |  | 3509358 (2815093 to 4429076) | 5923.46 (5047.86 to 7127.75) | | -0.11 (-0.38 to 0.16) |
| United States of America | 23477765 (20002836 to 28076779) | 9374.01 (7857.79 to 11294.15) |  | 33954468 (30347008 to 38109958) | 10399.27 (9140.29 to 11903.19) | | 0.7 (0.25 to 1.16) |
| United States Virgin Islands | 5387 (4343 to 6764) | 5019.94 (4065.55 to 6267.63) |  | 4724 (3919 to 5750) | 5366.08 (4255.76 to 6754.18) | | 0.32 (0.21 to 0.43) |
| Uruguay | 221568 (195396 to 251630) | 6693.56 (5875.56 to 7703.27) |  | 205339 (178243 to 237532) | 5663.19 (4778.55 to 6765.04) | | -0.63 (-0.78 to -0.48) |
| Uzbekistan | 737480 (617144 to 882556) | 4267.92 (3652.84 to 4907.67) |  | 944062 (779576 to 1162863) | 3058.75 (2581.57 to 3661.02) | | -1.22 (-1.38 to -1.06) |
| Vanuatu | 4902 (4121 to 5869) | 3425.91 (3019.57 to 3884.87) |  | 8924 (6535 to 11929) | 3047.07 (2203.69 to 3990.17) | | 0.05 (-0.46 to 0.56) |
| Venezuela (Bolivarian Republic of) | 999661 (785216 to 1272634) | 4672.99 (3795.94 to 5791.31) |  | 1041985 (829836 to 1304105) | 3882.41 (3058.39 to 4893.49) | | -0.7 (-0.87 to -0.53) |
| Viet Nam | 1708255 (1411737 to 2146063) | 2603.15 (2231.71 to 3086.15) |  | 2849227 (2429245 to 3358420) | 3092.64 (2613.07 to 3713.87) | | 0.69 (0.43 to 0.95) |
| Yemen | 599901 (498756 to 737931) | 4807.31 (4277.85 to 5430.54) |  | 1189810 (968154 to 1487503) | 3946.64 (3401.3 to 4620.19) | | -0.69 (-0.73 to -0.64) |
| Zambia | 199391 (160973 to 250481) | 2340.79 (2011.5 to 2747.57) |  | 459061 (353763 to 602972) | 2304.07 (1889.08 to 2844.53) | | 0.01 (-0.1 to 0.11) |
| Zimbabwe | 173744 (140921 to 218376) | 1915.39 (1669.6 to 2217.42) |  | 265194 (219778 to 321809) | 1989.98 (1728.06 to 2286.69) | | 0.17 (0.12 to 0.21) |

AAPC: average annual percent change; UI: uncertainty interval; CI: confidence interval; SDI: Socio-demographic index.

Table S5. Prevalence of ILD&PS (1990/2019) for sexes, SDI and locations, with AAPC (1990-2019)

| GBD data | 1990 | |  | 2019 | | AAPC % (95% CI)  1990-2019 |
| --- | --- | --- | --- | --- | --- | --- |
|  | Cases (95% UI) | Age-standardised prevalence per  100 000 population (95% UI) |  | Cases (95% UI) | Age-standardised prevalence per  100 000 population (95% UI) |  |
| Global | 2199426 (1857409 to 2566793) | 52.66 (44.49 to 61.07) |  | 4710180 (4020397 to 5401700) | 57.62 (49.42 to 65.67) | 0.33 (0.25 to 0.4) |
| **Sex** |  |  |  |  |  |  |
| Female | 1148716 (968103 to 1344666) | 52.2 (43.98 to 60.98) |  | 2432444 (2074827 to 2797609) | 56.45 (48.18 to 64.8) | 0.28 (0.19 to 0.37) |
| Male | 1050710 (887936 to 1223533) | 53.93 (45.41 to 62.61) |  | 2277737 (1946302 to 2613887) | 59.67 (51.21 to 68.03) | 0.37 (0.29 to 0.45) |
| **SDI** |  |  |  |  |  |  |
| High SDI | 876582 (740835 to 1023826) | 86.63 (73.26 to 100.96) |  | 1781755 (1530390 to 2022250) | 101.21 (87.66 to 113.79) | 0.57 (0.49 to 0.65) |
| High-middle SDI | 463437 (388732 to 543080) | 41.64 (34.96 to 48.65) |  | 895876 (759669 to 1032835) | 45.59 (38.85 to 52.37) | 0.32 (0.23 to 0.42) |
| Middle SDI | 382129 (318371 to 454100) | 32.36 (26.82 to 38.3) |  | 987915 (828680 to 1159449) | 38.99 (32.77 to 45.41) | 0.66 (0.51 to 0.82) |
| Low-middle SDI | 362975 (305222 to 426465) | 53.21 (44.29 to 62.64) |  | 786025 (663482 to 913233) | 54.34 (45.83 to 63.34) | 0.1 (0.04 to 0.16) |
| Low SDI | 113613 (96322 to 132564) | 38.42 (32.47 to 44.88) |  | 256901 (220001 to 298040) | 39.55 (33.5 to 46.06) | 0.16 (0.09 to 0.23) |
| **Region** |  |  |  |  |  |  |
| High-income Asia Pacific | 278861 (227833 to 333064) | 137.92 (113.32 to 164.29) |  | 609329 (506683 to 721140) | 147.91 (124.96 to 171.65) | 0.27 (0.19 to 0.36) |
| High-income North America | 385897 (322264 to 454482) | 114.5 (96.05 to 134.49) |  | 728252 (631563 to 826185) | 124.97 (109.31 to 140.35) | 0.22 (-0.3 to 0.74) |
| Western Europe | 251938 (216669 to 291111) | 46.63 (39.83 to 53.98) |  | 479935 (415783 to 546411) | 58.55 (50.53 to 66.9) | 0.92 (0.79 to 1.06) |
| Australasia | 10076 (8696 to 11529) | 43.27 (37.42 to 49.3) |  | 30332 (26256 to 34319) | 63.24 (55.07 to 71.19) | 1.33 (1.26 to 1.41) |
| Andean Latin America | 15296 (13585 to 16923) | 72.35 (64.38 to 80.1) |  | 68943 (62118 to 75174) | 124.12 (111.75 to 135.46) | 1.92 (1.75 to 2.1) |
| Tropical Latin America | 33544 (27622 to 40149) | 31.08 (25.71 to 37.1) |  | 49906 (41543 to 58946) | 20.66 (17.27 to 24.32) | -1.33 (-1.49 to -1.18) |
| Central Latin America | 42304 (35959 to 49664) | 44.25 (36.95 to 52.07) |  | 113640 (97318 to 131294) | 47.66 (40.82 to 55.11) | 0.25 (0.23 to 0.26) |
| Southern Latin America | 27562 (24231 to 30822) | 58.81 (51.91 to 65.82) |  | 80144 (71621 to 88294) | 97.12 (86.72 to 107.02) | 1.72 (1.62 to 1.82) |
| Caribbean | 5443 (4601 to 6377) | 19.18 (16.1 to 22.61) |  | 12412 (10686 to 14340) | 24.3 (20.98 to 28.2) | 0.84 (0.81 to 0.86) |
| Central Europe | 75023 (63917 to 87114) | 52.83 (45.12 to 61.42) |  | 95899 (82457 to 110660) | 56 (48.28 to 65.05) | 0.2 (0.04 to 0.36) |
| Eastern Europe | 124714 (102808 to 150614) | 47.14 (39.07 to 56.72) |  | 109496 (87640 to 136249) | 39.9 (31.99 to 49.78) | -0.41 (-0.63 to -0.2) |
| Central Asia | 21997 (19301 to 24891) | 43 (37.96 to 48.62) |  | 31415 (26918 to 36760) | 37.62 (32.58 to 43.17) | -0.47 (-0.59 to -0.36) |
| North Africa and Middle East | 71796 (60432 to 85117) | 31.4 (26.55 to 36.87) |  | 228150 (193608 to 268828) | 44.49 (38.2 to 51.28) | 1.28 (1.21 to 1.35) |
| South Asia | 444113 (371836 to 521870) | 69.72 (57.87 to 82.2) |  | 1029814 (863473 to 1203639) | 69.31 (58.01 to 81.62) | -0.01 (-0.07 to 0.04) |
| Southeast Asia | 39809 (33059 to 47456) | 13.34 (11 to 15.81) |  | 112400 (92693 to 133278) | 17.55 (14.54 to 20.64) | 0.95 (0.93 to 0.97) |
| East Asia | 289138 (232392 to 353783) | 29.68 (23.88 to 36.04) |  | 741803 (603595 to 895117) | 35.35 (28.99 to 41.76) | 0.76 (0.42 to 1.1) |
| Oceania | 3076 (2713 to 3481) | 66.03 (58.81 to 73.33) |  | 9060 (8062 to 10168) | 85.25 (76.15 to 94.25) | 0.88 (0.84 to 0.92) |
| Western Sub-Saharan Africa | 28883 (24161 to 34263) | 22.85 (19.27 to 27.1) |  | 68059 (56149 to 82502) | 22.07 (18.26 to 26.62) | -0.06 (-0.15 to 0.03) |
| Eastern Sub-Saharan Africa | 23352 (19638 to 27860) | 20.41 (17.23 to 24.15) |  | 57478 (47834 to 69033) | 22.65 (19.05 to 26.75) | 0.41 (0.34 to 0.48) |
| Central Sub-Saharan Africa | 8045 (6798 to 9500) | 24.66 (20.99 to 28.79) |  | 21483 (18057 to 25635) | 27.58 (23.5 to 32.08) | 0.44 (0.37 to 0.51) |
| Southern Sub-Saharan Africa | 18558 (15787 to 21563) | 56.11 (47.29 to 65.48) |  | 32232 (27299 to 38046) | 49.94 (42.07 to 58.86) | -0.36 (-0.41 to -0.31) |
| **Countries** |  |  |  |  |  |  |
| Afghanistan | 106 (84 to 132) | 1.41 (1.14 to 1.74) |  | 592 (442 to 778) | 2.88 (2.28 to 3.56) | 2.48 (2.12 to 2.85) |
| Albania | 310 (252 to 387) | 14.29 (11.66 to 17.5) |  | 700 (553 to 897) | 16.45 (13.14 to 20.94) | 0.61 (0.2 to 1.03) |
| Algeria | 52 (41 to 66) | 0.41 (0.34 to 0.51) |  | 231 (192 to 280) | 0.72 (0.61 to 0.87) | 2.03 (1.85 to 2.2) |
| American Samoa | 1 (1 to 1) | 3.96 (3.16 to 5.06) |  | 3 (2 to 4) | 6.29 (4.86 to 7.98) | 1.58 (1.47 to 1.69) |
| Andorra | 0 (0 to 0) | 0.15 (0.12 to 0.2) |  | 0 (0 to 0) | 0.12 (0.1 to 0.16) | -1.15 (-1.76 to -0.53) |
| Angola | 115 (97 to 135) | 3.31 (2.84 to 3.87) |  | 350 (294 to 410) | 3.7 (3.11 to 4.33) | 0.31 (0.16 to 0.47) |
| Antigua and Barbuda | 1 (0 to 1) | 1.05 (0.83 to 1.33) |  | 1 (1 to 1) | 0.88 (0.69 to 1.09) | -0.72 (-1.03 to -0.41) |
| Argentina | 720 (559 to 918) | 2.24 (1.75 to 2.84) |  | 2077 (1626 to 2683) | 3.74 (2.94 to 4.83) | 1.76 (1.7 to 1.83) |
| Armenia | 100 (83 to 120) | 3.74 (3.1 to 4.5) |  | 204 (165 to 256) | 4.89 (3.99 to 6.15) | 0.94 (0.85 to 1.02) |
| Australia | 404 (334 to 485) | 2.01 (1.68 to 2.41) |  | 1164 (926 to 1596) | 2.58 (2.06 to 3.52) | 0.96 (0.46 to 1.47) |
| Austria | 66 (53 to 81) | 0.56 (0.45 to 0.69) |  | 140 (110 to 178) | 0.78 (0.62 to 0.99) | 1.2 (0.84 to 1.57) |
| Azerbaijan | 99 (76 to 126) | 2.08 (1.6 to 2.68) |  | 300 (235 to 381) | 3.53 (2.77 to 4.54) | 1.88 (1.76 to 1.99) |
| Bahamas | 1 (1 to 1) | 0.68 (0.54 to 0.86) |  | 3 (2 to 3) | 0.7 (0.55 to 0.86) | 0.01 (-0.15 to 0.18) |
| Bahrain | 3 (2 to 4) | 1.01 (0.84 to 1.21) |  | 14 (11 to 18) | 1.26 (1.05 to 1.5) | 0.3 (-1.08 to 1.71) |
| Bangladesh | 1612 (1330 to 1954) | 3.52 (2.89 to 4.25) |  | 5475 (4381 to 6748) | 4.39 (3.51 to 5.37) | 0.77 (0.53 to 1.02) |
| Barbados | 2 (1 to 2) | 0.54 (0.42 to 0.69) |  | 3 (2 to 4) | 0.63 (0.49 to 0.81) | 0.49 (0.41 to 0.57) |
| Belarus | 768 (624 to 938) | 6.02 (4.89 to 7.34) |  | 1177 (944 to 1444) | 7.67 (6.17 to 9.46) | 0.84 (0.79 to 0.89) |
| Belgium | 355 (300 to 416) | 2.22 (1.88 to 2.61) |  | 110 (90 to 134) | 0.42 (0.35 to 0.51) | -6.32 (-7.11 to -5.53) |
| Belize | 1 (1 to 1) | 0.72 (0.59 to 0.89) |  | 3 (2 to 3) | 0.85 (0.69 to 1.04) | 0.46 (0.27 to 0.65) |
| Benin | 20 (17 to 24) | 0.91 (0.76 to 1.1) |  | 53 (43 to 63) | 0.96 (0.81 to 1.14) | 0.18 (0.12 to 0.24) |
| Bermuda | 1 (1 to 1) | 1.17 (0.98 to 1.41) |  | 1 (1 to 1) | 0.77 (0.62 to 0.96) | -1.56 (-1.68 to -1.43) |
| Bhutan | 6 (5 to 8) | 2.69 (2.23 to 3.31) |  | 21 (17 to 26) | 3.97 (3.23 to 4.81) | 1.24 (1.1 to 1.37) |
| Bolivia (Plurinational State of) | 89 (71 to 122) | 2.77 (2.2 to 3.8) |  | 388 (307 to 495) | 4.61 (3.66 to 5.88) | 1.67 (1.41 to 1.93) |
| Bosnia and Herzegovina | 231 (184 to 289) | 5.5 (4.46 to 6.78) |  | 541 (428 to 674) | 9.45 (7.57 to 11.78) | 1.93 (1.81 to 2.04) |
| Botswana | 60 (49 to 77) | 10.72 (8.84 to 13.65) |  | 149 (123 to 177) | 11.61 (9.68 to 13.73) | -0.05 (-0.41 to 0.3) |
| Brazil | 22221 (18797 to 27665) | 21.41 (18 to 26.55) |  | 49744 (41788 to 60362) | 20.6 (17.33 to 24.95) | -0.37 (-0.83 to 0.1) |
| Brunei Darussalam | 3 (2 to 4) | 3.71 (2.99 to 4.65) |  | 9 (8 to 12) | 4.16 (3.31 to 5.28) | 0.51 (0.35 to 0.67) |
| Bulgaria | 2128 (1761 to 2588) | 16.68 (13.85 to 20.16) |  | 1833 (1499 to 2211) | 13.13 (10.76 to 15.91) | -0.99 (-1.36 to -0.62) |
| Burkina Faso | 48 (39 to 58) | 1.01 (0.84 to 1.22) |  | 86 (70 to 104) | 0.86 (0.72 to 1.03) | -0.6 (-0.7 to -0.49) |
| Burundi | 91 (77 to 106) | 3.87 (3.28 to 4.51) |  | 154 (127 to 185) | 3.83 (3.14 to 4.58) | -0.3 (-0.6 to 0.01) |
| Cabo Verde | 1 (1 to 2) | 0.55 (0.45 to 0.66) |  | 3 (2 to 3) | 0.64 (0.52 to 0.77) | 0.41 (0.25 to 0.57) |
| Cambodia | 116 (86 to 153) | 2.35 (1.73 to 3.08) |  | 494 (363 to 652) | 3.9 (2.9 to 5.11) | 1.82 (1.73 to 1.92) |
| Cameroon | 66 (54 to 82) | 1.3 (1.08 to 1.58) |  | 175 (143 to 212) | 1.24 (1.04 to 1.48) | -0.23 (-0.33 to -0.13) |
| Canada | 3069 (2538 to 3723) | 9.29 (7.72 to 11.15) |  | 5705 (4761 to 6855) | 8.3 (6.97 to 9.9) | -0.41 (-0.54 to -0.27) |
| Central African Republic | 39 (33 to 45) | 3.65 (3.11 to 4.23) |  | 74 (63 to 87) | 3.94 (3.37 to 4.59) | 0.12 (0.01 to 0.22) |
| Chad | 27 (22 to 32) | 0.88 (0.73 to 1.06) |  | 55 (45 to 67) | 0.9 (0.74 to 1.07) | 0.04 (0 to 0.08) |
| Chile | 1155 (938 to 1427) | 11.97 (9.75 to 14.76) |  | 4018 (3168 to 5129) | 16.61 (13.1 to 21.2) | 0.86 (0.32 to 1.4) |
| China | 1330860 (1083676 to 1672573) | 143.1 (117.11 to 178.64) |  | 2587176 (2153538 to 3082660) | 123.12 (102.96 to 146.36) | -0.59 (-0.96 to -0.21) |
| Colombia | 940 (779 to 1126) | 5.12 (4.28 to 6.07) |  | 2737 (2231 to 3398) | 5.2 (4.23 to 6.45) | 0.02 (-0.14 to 0.18) |
| Comoros | 6 (6 to 8) | 2.98 (2.54 to 3.47) |  | 15 (13 to 18) | 3.23 (2.72 to 3.81) | 0.11 (-0.09 to 0.31) |
| Congo | 40 (34 to 47) | 3.9 (3.37 to 4.5) |  | 103 (88 to 120) | 4.4 (3.77 to 5.14) | 0.42 (0.22 to 0.62) |
| Cook Islands | 1 (0 to 1) | 4.09 (3.16 to 5.3) |  | 2 (1 to 2) | 6.08 (4.66 to 7.82) | 1.43 (1.29 to 1.58) |
| Costa Rica | 62 (50 to 78) | 3.24 (2.59 to 4.08) |  | 221 (179 to 277) | 4.29 (3.46 to 5.38) | 0.96 (0.92 to 1) |
| Croatia | 596 (490 to 726) | 9.39 (7.76 to 11.41) |  | 915 (742 to 1104) | 11.46 (9.38 to 13.86) | 0.69 (0.64 to 0.74) |
| Cuba | 58 (45 to 73) | 0.56 (0.43 to 0.7) |  | 119 (92 to 152) | 0.65 (0.51 to 0.83) | 0.52 (0.47 to 0.56) |
| Cyprus | 1 (1 to 1) | 0.12 (0.1 to 0.16) |  | 1 (1 to 2) | 0.09 (0.07 to 0.12) | -1.28 (-2.21 to -0.34) |
| Czechia | 804 (633 to 1063) | 5.92 (4.66 to 7.76) |  | 1711 (1372 to 2094) | 8.78 (6.98 to 10.95) | 1.38 (1.04 to 1.72) |
| Côte d’Ivoire | 63 (50 to 78) | 1.24 (1.01 to 1.5) |  | 156 (125 to 190) | 1.22 (1.02 to 1.45) | -0.05 (-0.17 to 0.08) |
| Democratic People's Republic of Korea | 6928 (5440 to 12604) | 38.36 (30.45 to 67.58) |  | 20522 (16087 to 28148) | 61.5 (48.34 to 83.93) | 1.07 (0.54 to 1.6) |
| Democratic Republic of the Congo | 461 (390 to 541) | 3.35 (2.84 to 3.9) |  | 1249 (1064 to 1452) | 4.08 (3.48 to 4.76) | 0.58 (0.42 to 0.73) |
| Denmark | 15 (12 to 18) | 0.18 (0.14 to 0.23) |  | 11 (8 to 14) | 0.1 (0.07 to 0.14) | -2.38 (-2.89 to -1.87) |
| Djibouti | 4 (3 to 4) | 2.93 (2.46 to 3.45) |  | 19 (16 to 23) | 3.64 (3.01 to 4.33) | 0.56 (0.38 to 0.75) |
| Dominica | 0 (0 to 0) | 0.55 (0.43 to 0.7) |  | 1 (0 to 1) | 0.66 (0.52 to 0.84) | 0.64 (0.6 to 0.68) |
| Dominican Republic | 22 (17 to 28) | 0.53 (0.41 to 0.68) |  | 59 (46 to 74) | 0.62 (0.49 to 0.79) | 0.52 (0.5 to 0.54) |
| Ecuador | 113 (92 to 143) | 2.1 (1.69 to 2.64) |  | 510 (415 to 624) | 3.44 (2.79 to 4.21) | 1.87 (1.64 to 2.11) |
| Egypt | 148 (117 to 183) | 0.47 (0.38 to 0.56) |  | 616 (494 to 746) | 0.96 (0.79 to 1.14) | 2.54 (2.33 to 2.76) |
| El Salvador | 100 (80 to 125) | 3.21 (2.54 to 4.02) |  | 236 (190 to 298) | 3.95 (3.17 to 5.01) | 0.73 (0.68 to 0.77) |
| Equatorial Guinea | 6 (5 to 7) | 3.43 (2.96 to 3.99) |  | 14 (12 to 17) | 3.44 (2.93 to 4.04) | -0.11 (-0.32 to 0.09) |
| Eritrea | 33 (28 to 39) | 3.24 (2.75 to 3.8) |  | 89 (75 to 105) | 3.48 (2.94 to 4.07) | 0.13 (0.01 to 0.26) |
| Estonia | 106 (85 to 131) | 5.3 (4.27 to 6.56) |  | 181 (143 to 225) | 7.59 (6.01 to 9.55) | 1.26 (1.17 to 1.35) |
| Eswatini | 42 (35 to 53) | 14.92 (12.46 to 18.88) |  | 84 (70 to 102) | 14.86 (12.54 to 18.1) | 0.04 (-0.44 to 0.51) |
| Ethiopia | 895 (758 to 1056) | 4.15 (3.53 to 4.88) |  | 1180 (1002 to 1399) | 2.88 (2.45 to 3.42) | -1.34 (-1.41 to -1.26) |
| Fiji | 15 (11 to 19) | 3.97 (3.11 to 5.16) |  | 44 (33 to 57) | 5.76 (4.38 to 7.39) | 1.31 (1.22 to 1.39) |
| Finland | 20 (16 to 24) | 0.28 (0.23 to 0.34) |  | 30 (24 to 37) | 0.25 (0.2 to 0.31) | 0.07 (-0.73 to 0.87) |
| France | 797 (666 to 959) | 0.92 (0.77 to 1.1) |  | 563 (455 to 693) | 0.36 (0.29 to 0.44) | -4.21 (-5.62 to -2.77) |
| Gabon | 17 (15 to 20) | 3.19 (2.74 to 3.73) |  | 37 (31 to 43) | 3.82 (3.23 to 4.5) | 0.52 (0.44 to 0.59) |
| Gambia | 4 (3 to 5) | 1.03 (0.85 to 1.24) |  | 11 (9 to 13) | 1.05 (0.89 to 1.25) | -0.02 (-0.15 to 0.12) |
| Georgia | 126 (98 to 163) | 2.07 (1.62 to 2.63) |  | 242 (197 to 298) | 3.99 (3.26 to 4.86) | 2.41 (2.25 to 2.58) |
| Germany | 623 (500 to 775) | 0.48 (0.38 to 0.59) |  | 609 (479 to 770) | 0.28 (0.22 to 0.34) | -2.45 (-3.48 to -1.41) |
| Ghana | 116 (92 to 143) | 1.53 (1.26 to 1.87) |  | 179 (145 to 216) | 0.99 (0.83 to 1.17) | -1.55 (-1.74 to -1.35) |
| Greece | 9 (6 to 12) | 0.07 (0.05 to 0.1) |  | 11 (8 to 15) | 0.07 (0.04 to 0.09) | -0.11 (-0.47 to 0.25) |
| Greenland | 3 (3 to 4) | 10.92 (8.51 to 13.9) |  | 6 (4 to 7) | 8.49 (6.65 to 10.72) | -1.14 (-1.96 to -0.31) |
| Grenada | 1 (1 to 1) | 0.92 (0.77 to 1.1) |  | 1 (1 to 1) | 0.84 (0.69 to 1.04) | -0.51 (-0.73 to -0.29) |
| Guam | 3 (2 to 4) | 3.99 (3.18 to 5.11) |  | 12 (9 to 15) | 6.05 (4.63 to 7.72) | 1.44 (1.32 to 1.57) |
| Guatemala | 153 (122 to 188) | 3.75 (3.05 to 4.62) |  | 478 (384 to 590) | 4.03 (3.23 to 4.96) | 0.12 (-0.14 to 0.37) |
| Guinea | 36 (29 to 43) | 1.01 (0.84 to 1.22) |  | 71 (58 to 85) | 1.16 (0.97 to 1.37) | 0.42 (0.35 to 0.5) |
| Guinea-Bissau | 8 (6 to 10) | 1.65 (1.35 to 1.99) |  | 13 (10 to 16) | 1.42 (1.18 to 1.72) | -0.51 (-0.59 to -0.43) |
| Guyana | 3 (2 to 4) | 0.7 (0.56 to 0.87) |  | 5 (4 to 6) | 0.73 (0.59 to 0.92) | 0.11 (0.04 to 0.19) |
| Haiti | 25 (20 to 31) | 0.7 (0.58 to 0.86) |  | 62 (50 to 77) | 0.82 (0.67 to 1.02) | 0.48 (0.37 to 0.6) |
| Honduras | 83 (68 to 102) | 3.67 (3.02 to 4.49) |  | 284 (228 to 349) | 4.44 (3.62 to 5.48) | 0.62 (0.54 to 0.69) |
| Hungary | 1380 (1114 to 1681) | 9.47 (7.66 to 11.53) |  | 2283 (1846 to 2807) | 12.57 (10.16 to 15.45) | 0.9 (0.48 to 1.32) |
| Iceland | 0 (0 to 0) | 0.07 (0.04 to 0.1) |  | 0 (0 to 0) | 0.06 (0.03 to 0.09) | -0.65 (-0.9 to -0.39) |
| India | 35898 (29665 to 43411) | 7.98 (6.65 to 9.56) |  | 79358 (66736 to 96189) | 6.87 (5.82 to 8.3) | -0.55 (-0.65 to -0.45) |
| Indonesia | 7827 (5933 to 10751) | 6.66 (5.08 to 8.87) |  | 18865 (15028 to 24300) | 7.74 (6.21 to 9.78) | 0.6 (0.2 to 1) |
| Iran (Islamic Republic of) | 575 (457 to 711) | 1.75 (1.46 to 2.08) |  | 5749 (4497 to 7201) | 6.93 (5.57 to 8.46) | 4.7 (4.12 to 5.28) |
| Iraq | 121 (98 to 147) | 1.3 (1.08 to 1.55) |  | 249 (202 to 300) | 1.05 (0.89 to 1.25) | -0.96 (-1.25 to -0.68) |
| Ireland | 3 (2 to 5) | 0.09 (0.06 to 0.12) |  | 6 (4 to 8) | 0.08 (0.06 to 0.12) | -0.33 (-0.74 to 0.08) |
| Israel | 3 (2 to 5) | 0.07 (0.05 to 0.1) |  | 6 (4 to 9) | 0.06 (0.04 to 0.09) | -0.45 (-0.61 to -0.3) |
| Italy | 58800 (45508 to 78288) | 63.64 (49.34 to 85.01) |  | 26559 (22575 to 31730) | 18.06 (15.36 to 21.21) | -4.5 (-4.89 to -4.1) |
| Jamaica | 16 (13 to 20) | 0.87 (0.7 to 1.09) |  | 19 (15 to 25) | 0.63 (0.48 to 0.81) | -1.48 (-2.18 to -0.78) |
| Japan | 60886 (48912 to 77310) | 35.77 (28.87 to 45.19) |  | 52224 (44054 to 61549) | 12.79 (10.81 to 15.2) | -4.02 (-4.55 to -3.49) |
| Jordan | 20 (15 to 26) | 1.02 (0.83 to 1.23) |  | 59 (47 to 73) | 0.78 (0.64 to 0.94) | -0.97 (-1.16 to -0.77) |
| Kazakhstan | 647 (527 to 794) | 5.31 (4.32 to 6.52) |  | 1152 (934 to 1463) | 7.02 (5.65 to 8.84) | 0.54 (0.03 to 1.05) |
| Kenya | 297 (255 to 348) | 3.76 (3.2 to 4.41) |  | 770 (659 to 901) | 3.64 (3.11 to 4.3) | -0.26 (-0.48 to -0.03) |
| Kiribati | 2 (2 to 3) | 5.36 (4.2 to 7.13) |  | 5 (4 to 7) | 7.84 (6.07 to 10.49) | 1.19 (1.03 to 1.36) |
| Kuwait | 5 (4 to 7) | 0.55 (0.45 to 0.66) |  | 33 (25 to 41) | 0.91 (0.75 to 1.09) | 1.47 (0.95 to 2) |
| Kyrgyzstan | 62 (49 to 80) | 2.09 (1.62 to 2.7) |  | 147 (116 to 188) | 3.49 (2.75 to 4.53) | 1.83 (1.71 to 1.94) |
| Lao People's Democratic Republic | 58 (42 to 79) | 2.56 (1.89 to 3.46) |  | 211 (154 to 281) | 4.49 (3.3 to 6.01) | 1.99 (1.92 to 2.07) |
| Latvia | 290 (225 to 388) | 8.22 (6.37 to 10.98) |  | 258 (205 to 322) | 7.45 (5.77 to 9.35) | -0.32 (-0.44 to -0.21) |
| Lebanon | 12 (10 to 15) | 0.53 (0.43 to 0.63) |  | 36 (30 to 43) | 0.69 (0.58 to 0.83) | 1 (0.76 to 1.24) |
| Lesotho | 131 (108 to 173) | 13.51 (11.2 to 17.63) |  | 262 (216 to 324) | 20.64 (17.17 to 25.41) | 1.23 (0.96 to 1.49) |
| Liberia | 10 (8 to 12) | 0.87 (0.72 to 1.04) |  | 21 (17 to 25) | 0.91 (0.76 to 1.08) | 0.21 (0.05 to 0.37) |
| Libya | 10 (8 to 13) | 0.48 (0.4 to 0.59) |  | 61 (49 to 74) | 1.06 (0.89 to 1.27) | 2.78 (2.6 to 2.97) |
| Lithuania | 355 (297 to 421) | 7.98 (6.7 to 9.48) |  | 492 (406 to 596) | 9.09 (7.5 to 10.94) | 0.27 (0.09 to 0.45) |
| Luxembourg | 2 (1 to 2) | 0.27 (0.21 to 0.35) |  | 1 (1 to 2) | 0.13 (0.1 to 0.17) | -3.17 (-4.08 to -2.25) |
| Madagascar | 162 (134 to 193) | 3.41 (2.82 to 4.05) |  | 346 (282 to 420) | 3.81 (3.12 to 4.62) | 0.23 (-0.06 to 0.51) |
| Malawi | 97 (81 to 114) | 2.66 (2.25 to 3.15) |  | 216 (180 to 256) | 3.16 (2.63 to 3.79) | 0.45 (0.28 to 0.62) |
| Malaysia | 256 (191 to 343) | 2.55 (1.92 to 3.39) |  | 1337 (965 to 1766) | 4.85 (3.52 to 6.37) | 2.24 (2.17 to 2.31) |
| Maldives | 4 (3 to 6) | 4.18 (3.28 to 5.4) |  | 19 (14 to 25) | 5.4 (3.95 to 7.14) | 0.92 (0.85 to 0.99) |
| Mali | 48 (39 to 59) | 1.07 (0.89 to 1.3) |  | 77 (64 to 93) | 0.85 (0.72 to 1.02) | -0.8 (-0.92 to -0.69) |
| Malta | 0 (0 to 1) | 0.12 (0.09 to 0.15) |  | 1 (1 to 1) | 0.1 (0.07 to 0.13) | -1.01 (-1.54 to -0.47) |
| Marshall Islands | 1 (1 to 1) | 4.15 (3.31 to 5.25) |  | 2 (2 to 3) | 6.43 (5.05 to 8.08) | 1.52 (1.46 to 1.58) |
| Mauritania | 15 (12 to 18) | 1.32 (1.1 to 1.59) |  | 21 (17 to 25) | 0.95 (0.79 to 1.12) | -1.17 (-1.37 to -0.97) |
| Mauritius | 18 (14 to 24) | 2.3 (1.72 to 3.02) |  | 79 (56 to 106) | 4.4 (3.18 to 5.93) | 2.3 (2.22 to 2.38) |
| Mexico | 13241 (11018 to 15758) | 29.75 (24.58 to 35.98) |  | 24062 (20355 to 28617) | 19.98 (16.9 to 23.91) | -1.37 (-1.71 to -1.03) |
| Micronesia (Federated States of) | 2 (2 to 2) | 4.18 (3.37 to 5.24) |  | 4 (3 to 6) | 5.98 (4.67 to 7.53) | 1.25 (1.21 to 1.29) |
| Monaco | 0 (0 to 0) | 0.38 (0.3 to 0.47) |  | 0 (0 to 1) | 0.39 (0.31 to 0.48) | -0.46 (-1.67 to 0.76) |
| Mongolia | 35 (28 to 44) | 3.3 (2.62 to 4.14) |  | 84 (68 to 104) | 4.12 (3.33 to 5.12) | 0.78 (0.68 to 0.89) |
| Montenegro | 37 (31 to 46) | 5.93 (4.88 to 7.25) |  | 90 (72 to 110) | 9.44 (7.58 to 11.54) | 1.67 (1.59 to 1.76) |
| Morocco | 65 (51 to 81) | 0.44 (0.36 to 0.53) |  | 254 (206 to 308) | 0.84 (0.69 to 0.99) | 2.23 (2.05 to 2.41) |
| Mozambique | 153 (128 to 181) | 2.73 (2.29 to 3.22) |  | 343 (278 to 427) | 3.39 (2.74 to 4.24) | 0.61 (0.42 to 0.79) |
| Myanmar | 837 (659 to 1066) | 3.14 (2.48 to 3.98) |  | 2124 (1614 to 2747) | 4.32 (3.26 to 5.55) | 1.13 (1.02 to 1.23) |
| Namibia | 79 (67 to 93) | 11 (9.3 to 12.8) |  | 150 (127 to 175) | 10.98 (9.26 to 12.8) | -0.03 (-0.16 to 0.09) |
| Nauru | 0 (0 to 0) | 4.01 (3.15 to 5.22) |  | 0 (0 to 0) | 5.65 (4.39 to 7.11) | 1.28 (1.18 to 1.38) |
| Nepal | 306 (244 to 381) | 3.43 (2.78 to 4.22) |  | 890 (728 to 1086) | 4.23 (3.48 to 5.13) | 0.67 (0.49 to 0.86) |
| Netherlands | 118 (93 to 150) | 0.56 (0.44 to 0.71) |  | 31 (24 to 40) | 0.1 (0.08 to 0.14) | -6.03 (-6.74 to -5.31) |
| New Zealand | 262 (214 to 317) | 6.49 (5.34 to 7.82) |  | 495 (418 to 583) | 6.17 (5.25 to 7.24) | -0.3 (-0.92 to 0.32) |
| Nicaragua | 59 (48 to 73) | 3.5 (2.81 to 4.29) |  | 192 (152 to 242) | 4.1 (3.26 to 5.19) | 0.5 (0.4 to 0.59) |
| Niger | 30 (25 to 36) | 0.93 (0.78 to 1.11) |  | 68 (56 to 81) | 0.81 (0.69 to 0.96) | -0.49 (-0.61 to -0.37) |
| Nigeria | 686 (564 to 830) | 1.37 (1.15 to 1.64) |  | 889 (741 to 1055) | 0.92 (0.77 to 1.08) | -1.37 (-1.58 to -1.15) |
| Niue | 0 (0 to 0) | 3.48 (2.72 to 4.51) |  | 0 (0 to 0) | 5.68 (4.35 to 7.24) | 1.77 (1.68 to 1.87) |
| North Macedonia | 175 (140 to 243) | 9.16 (7.35 to 12.57) |  | 298 (239 to 369) | 9.44 (7.63 to 11.58) | -0.04 (-0.2 to 0.12) |
| Northern Mariana Islands | 1 (1 to 1) | 4.35 (3.44 to 5.61) |  | 3 (2 to 4) | 6.05 (4.6 to 7.8) | 1.19 (0.97 to 1.41) |
| Norway | 728 (587 to 895) | 10.67 (8.67 to 13.04) |  | 699 (571 to 861) | 8.08 (6.62 to 10.07) | -1.09 (-1.28 to -0.9) |
| Oman | 5 (4 to 7) | 0.57 (0.47 to 0.69) |  | 18 (14 to 23) | 0.79 (0.66 to 0.94) | 1.03 (0.82 to 1.23) |
| Pakistan | 2008 (1634 to 2434) | 3.58 (2.91 to 4.36) |  | 3251 (2681 to 3897) | 3 (2.49 to 3.6) | -0.7 (-0.81 to -0.59) |
| Palau | 1 (1 to 1) | 10.28 (8.3 to 12.57) |  | 3 (3 to 4) | 15.02 (12.01 to 18.4) | 0.94 (0.41 to 1.48) |
| Palestine | 5 (4 to 6) | 0.5 (0.41 to 0.6) |  | 14 (11 to 18) | 0.54 (0.45 to 0.66) | 0.36 (0.16 to 0.56) |
| Panama | 53 (43 to 66) | 3.31 (2.65 to 4.07) |  | 176 (141 to 222) | 4.24 (3.4 to 5.35) | 0.89 (0.84 to 0.94) |
| Papua New Guinea | 90 (71 to 113) | 4.84 (3.87 to 5.95) |  | 329 (256 to 416) | 6.94 (5.46 to 8.64) | 1.23 (1.14 to 1.33) |
| Paraguay | 341 (281 to 453) | 13.64 (11.26 to 18.56) |  | 884 (740 to 1061) | 14.88 (12.47 to 17.75) | -0.04 (-0.46 to 0.38) |
| Peru | 479 (390 to 615) | 4.03 (3.25 to 5.19) |  | 1832 (1450 to 2275) | 5.82 (4.59 to 7.24) | 0.99 (0.4 to 1.59) |
| Philippines | 830 (641 to 1074) | 2.5 (1.94 to 3.26) |  | 3170 (2373 to 4217) | 3.73 (2.84 to 4.98) | 1.5 (1.31 to 1.69) |
| Poland | 11240 (9073 to 14610) | 25.73 (20.74 to 33.5) |  | 8970 (7678 to 10485) | 13.44 (11.57 to 15.64) | -2.39 (-2.68 to -2.08) |
| Portugal | 182 (145 to 225) | 1.25 (1 to 1.55) |  | 144 (112 to 186) | 0.59 (0.46 to 0.77) | -3.43 (-4.77 to -2.07) |
| Puerto Rico | 20 (16 to 26) | 0.56 (0.44 to 0.7) |  | 43 (33 to 57) | 0.65 (0.51 to 0.84) | 0.52 (0.48 to 0.57) |
| Qatar | 1 (1 to 1) | 0.44 (0.36 to 0.53) |  | 8 (6 to 11) | 0.62 (0.51 to 0.77) | 1.32 (0.9 to 1.74) |
| Republic of Korea | 2317 (1953 to 2800) | 8.33 (7.03 to 9.98) |  | 6701 (5537 to 8273) | 7.51 (6.23 to 9.26) | -0.69 (-1.28 to -0.1) |
| Republic of Moldova | 230 (182 to 290) | 5.22 (4.18 to 6.51) |  | 432 (343 to 547) | 7.68 (6.06 to 9.77) | 1.44 (1.33 to 1.54) |
| Romania | 3587 (2989 to 4332) | 12.73 (10.66 to 15.22) |  | 4059 (3255 to 4959) | 11.63 (9.35 to 14.3) | -0.49 (-0.74 to -0.24) |
| Russian Federation | 9361 (7623 to 11552) | 5.28 (4.33 to 6.5) |  | 19273 (15919 to 23343) | 8.47 (7 to 10.26) | 1.66 (1.42 to 1.91) |
| Rwanda | 100 (82 to 119) | 3.51 (2.88 to 4.19) |  | 177 (144 to 228) | 3.26 (2.65 to 4.17) | -0.3 (-0.51 to -0.09) |
| Saint Kitts and Nevis | 0 (0 to 0) | 0.68 (0.53 to 0.86) |  | 0 (0 to 1) | 0.68 (0.53 to 0.87) | -0.03 (-0.12 to 0.05) |
| Saint Lucia | 1 (1 to 1) | 0.86 (0.69 to 1.08) |  | 1 (1 to 2) | 0.71 (0.56 to 0.92) | -0.77 (-0.95 to -0.6) |
| Saint Vincent and the Grenadines | 1 (1 to 1) | 0.89 (0.71 to 1.11) |  | 1 (1 to 2) | 1.08 (0.86 to 1.38) | 0.42 (-0.02 to 0.86) |
| Samoa | 3 (3 to 4) | 3.92 (3.08 to 5.05) |  | 9 (7 to 11) | 5.92 (4.54 to 7.57) | 1.43 (1.33 to 1.54) |
| San Marino | 0 (0 to 0) | 0.12 (0.09 to 0.16) |  | 0 (0 to 0) | 0.11 (0.08 to 0.14) | -1 (-1.55 to -0.44) |
| Sao Tome and Principe | 0 (0 to 1) | 0.72 (0.6 to 0.86) |  | 1 (1 to 1) | 0.95 (0.8 to 1.12) | 0.91 (0.81 to 1.02) |
| Saudi Arabia | 29 (22 to 36) | 0.41 (0.34 to 0.51) |  | 130 (102 to 164) | 0.67 (0.55 to 0.82) | 1.63 (1.53 to 1.74) |
| Senegal | 36 (29 to 44) | 1 (0.83 to 1.2) |  | 93 (76 to 111) | 1.12 (0.94 to 1.33) | 0.41 (0.28 to 0.53) |
| Serbia | 556 (450 to 684) | 4.93 (4.01 to 6.04) |  | 1561 (1255 to 1923) | 10.38 (8.43 to 12.69) | 2.61 (2.54 to 2.69) |
| Seychelles | 1 (1 to 2) | 2.37 (1.77 to 3.14) |  | 5 (4 to 7) | 4.51 (3.25 to 6.04) | 2.3 (2.21 to 2.38) |
| Sierra Leone | 16 (14 to 20) | 0.81 (0.68 to 0.97) |  | 41 (34 to 49) | 1.03 (0.86 to 1.21) | 0.86 (0.79 to 0.93) |
| Singapore | 82 (68 to 101) | 4.18 (3.44 to 5.12) |  | 369 (294 to 463) | 5.02 (4.01 to 6.31) | 0.65 (0.54 to 0.75) |
| Slovakia | 678 (538 to 993) | 11.38 (9.09 to 16.45) |  | 1141 (907 to 1409) | 12.71 (10.18 to 15.74) | 0.29 (0.09 to 0.5) |
| Slovenia | 331 (267 to 421) | 13.61 (11.01 to 17.25) |  | 559 (451 to 692) | 14.58 (11.68 to 17.86) | 0.18 (0.02 to 0.34) |
| Solomon Islands | 6 (5 to 8) | 4.6 (3.67 to 5.84) |  | 19 (15 to 24) | 6.29 (4.96 to 7.93) | 1.11 (0.98 to 1.23) |
| Somalia | 80 (66 to 99) | 3.5 (2.86 to 4.35) |  | 217 (176 to 268) | 3.55 (2.88 to 4.42) | -0.04 (-0.44 to 0.37) |
| South Africa | 3812 (3247 to 4448) | 18.31 (15.6 to 21.23) |  | 9745 (8278 to 11372) | 21.5 (18.38 to 25.02) | 0.36 (0.06 to 0.66) |
| South Sudan | 63 (53 to 75) | 2.77 (2.32 to 3.29) |  | 101 (86 to 120) | 3.01 (2.56 to 3.53) | 0.23 (0.11 to 0.35) |
| Spain | 543 (424 to 691) | 0.96 (0.75 to 1.22) |  | 350 (279 to 433) | 0.31 (0.25 to 0.39) | -4.74 (-5.98 to -3.48) |
| Sri Lanka | 346 (264 to 454) | 2.92 (2.22 to 3.85) |  | 1171 (850 to 1567) | 4.51 (3.28 to 6) | 1.53 (1.49 to 1.58) |
| Sudan | 43 (33 to 55) | 0.42 (0.34 to 0.52) |  | 156 (123 to 194) | 0.72 (0.6 to 0.87) | 1.93 (1.81 to 2.05) |
| Suriname | 4 (3 to 5) | 1.59 (1.28 to 2) |  | 6 (5 to 8) | 1.05 (0.82 to 1.36) | -1.65 (-2.41 to -0.89) |
| Sweden | 126 (92 to 250) | 0.75 (0.57 to 1.42) |  | 70 (56 to 90) | 0.34 (0.28 to 0.43) | -2.77 (-3.65 to -1.89) |
| Switzerland | 59 (49 to 72) | 0.53 (0.44 to 0.64) |  | 43 (34 to 54) | 0.25 (0.19 to 0.31) | -2.1 (-2.85 to -1.34) |
| Syrian Arab Republic | 39 (30 to 49) | 0.61 (0.5 to 0.73) |  | 87 (70 to 106) | 0.75 (0.62 to 0.89) | 0.69 (0.49 to 0.89) |
| Taiwan (Province of China) | 6141 (5164 to 7288) | 38.09 (32.23 to 45) |  | 9756 (8614 to 11037) | 24.68 (21.78 to 27.91) | -2.17 (-3.1 to -1.23) |
| Tajikistan | 63 (49 to 80) | 2.31 (1.79 to 2.98) |  | 168 (131 to 213) | 3.83 (2.95 to 4.91) | 1.8 (1.67 to 1.93) |
| Thailand | 1106 (844 to 1467) | 2.75 (2.12 to 3.65) |  | 4828 (3563 to 6472) | 4.65 (3.46 to 6.21) | 1.75 (1.64 to 1.85) |
| Timor-Leste | 9 (6 to 12) | 2.66 (1.95 to 3.61) |  | 39 (28 to 51) | 4.66 (3.45 to 6.11) | 2.01 (1.92 to 2.09) |
| Togo | 16 (13 to 20) | 1.09 (0.91 to 1.3) |  | 42 (34 to 51) | 1.02 (0.86 to 1.21) | -0.24 (-0.32 to -0.16) |
| Tokelau | 0 (0 to 0) | 3.52 (2.78 to 4.47) |  | 0 (0 to 0) | 6.12 (4.69 to 7.88) | 2 (1.89 to 2.1) |
| Tonga | 2 (2 to 3) | 3.99 (3.17 to 5.12) |  | 5 (4 to 6) | 5.71 (4.45 to 7.25) | 1.27 (1.15 to 1.39) |
| Trinidad and Tobago | 5 (4 to 6) | 0.53 (0.41 to 0.68) |  | 11 (9 to 14) | 0.62 (0.48 to 0.8) | 0.51 (0.31 to 0.71) |
| Tunisia | 20 (16 to 25) | 0.4 (0.32 to 0.48) |  | 80 (67 to 97) | 0.69 (0.57 to 0.82) | 1.94 (1.78 to 2.09) |
| Turkey | 2051 (1469 to 2760) | 4.59 (3.44 to 6.02) |  | 3859 (3022 to 4822) | 4.32 (3.42 to 5.37) | -0.47 (-0.83 to -0.11) |
| Turkmenistan | 38 (29 to 49) | 2.08 (1.61 to 2.68) |  | 120 (95 to 152) | 3.37 (2.65 to 4.24) | 1.69 (1.54 to 1.83) |
| Tuvalu | 0 (0 to 0) | 3.24 (2.55 to 4.19) |  | 1 (0 to 1) | 5.62 (4.36 to 7.29) | 1.97 (1.86 to 2.08) |
| Uganda | 175 (145 to 208) | 2.87 (2.4 to 3.42) |  | 377 (317 to 444) | 2.96 (2.47 to 3.49) | -0.01 (-0.21 to 0.2) |
| Ukraine | 13678 (11147 to 17414) | 19.05 (15.61 to 24.17) |  | 6605 (5407 to 8069) | 8.99 (7.35 to 11.01) | -2.83 (-3.25 to -2.41) |
| United Arab Emirates | 4 (3 to 5) | 0.49 (0.4 to 0.59) |  | 38 (29 to 48) | 0.91 (0.76 to 1.09) | 2.08 (1.93 to 2.22) |
| United Kingdom | 6679 (5735 to 7803) | 7.26 (6.26 to 8.51) |  | 8777 (7587 to 10089) | 6.79 (5.89 to 7.75) | -0.51 (-0.73 to -0.29) |
| United Republic of Tanzania | 263 (218 to 314) | 2.5 (2.1 to 2.98) |  | 659 (553 to 787) | 2.88 (2.42 to 3.43) | 0.42 (0.33 to 0.52) |
| United States of America | 37580 (31804 to 44364) | 11.72 (9.94 to 13.83) |  | 46307 (40897 to 52301) | 8.34 (7.39 to 9.38) | -1.31 (-1.55 to -1.07) |
| United States Virgin Islands | 1 (1 to 1) | 0.86 (0.7 to 1.07) |  | 2 (1 to 2) | 1.02 (0.84 to 1.25) | 0.59 (0.39 to 0.8) |
| Uruguay | 78 (58 to 104) | 1.93 (1.46 to 2.55) |  | 196 (146 to 263) | 3.4 (2.52 to 4.56) | 1.96 (1.85 to 2.07) |
| Uzbekistan | 278 (224 to 343) | 2.55 (2.04 to 3.14) |  | 828 (655 to 1045) | 4.77 (3.83 to 5.93) | 2.13 (1.98 to 2.28) |
| Vanuatu | 3 (2 to 4) | 4.4 (3.49 to 5.63) |  | 11 (8 to 14) | 6.32 (4.97 to 8.11) | 1.33 (1.21 to 1.44) |
| Venezuela (Bolivarian Republic of) | 370 (300 to 455) | 3.41 (2.78 to 4.16) |  | 1235 (1008 to 1517) | 4.23 (3.46 to 5.18) | 0.73 (0.7 to 0.76) |
| Viet Nam | 927 (694 to 1245) | 2.22 (1.66 to 2.99) |  | 4071 (2986 to 5444) | 4.08 (3.01 to 5.41) | 2.17 (2.09 to 2.25) |
| Yemen | 27 (21 to 34) | 0.47 (0.39 to 0.57) |  | 176 (136 to 220) | 1.03 (0.84 to 1.22) | 2.79 (2.62 to 2.95) |
| Zambia | 89 (76 to 105) | 3.29 (2.81 to 3.88) |  | 240 (204 to 278) | 3.84 (3.27 to 4.46) | 0.47 (0.3 to 0.63) |
| Zimbabwe | 192 (154 to 241) | 4.91 (3.99 to 6.07) |  | 396 (327 to 471) | 6.05 (5.06 to 7.15) | 0.69 (0.58 to 0.8) |

ILD&PS: Interstitial lung disease and pulmonary sarcoidosis; AAPC: average annual percent change; UI: uncertainty interval; CI: confidence interval; SDI: Socio-demographic index.

Table S6. Prevalence of PNE (1990/2019) for sexes, SDI and locations, with AAPC (1990-2019)

| GBD data | 1990 | |  | 2019 | | AAPC % (95% CI)  1990-2019 |
| --- | --- | --- | --- | --- | --- | --- |
|  | Cases (95% UI) | Age-standardised prevalence per  100 000 population (95% UI) |  | Cases (95% UI) | Age-standardised prevalence per  100 000 population (95% UI) |  |
| Global | 1670851 (1395137 to 2058099) | 40.14 (33.62 to 49.32) |  | 3072550 (2596999 to 3596518) | 36.78 (31.1 to 43.08) | -0.38 (-0.71 to -0.04) |
| **Sex** |  |  |  |  |  |  |
| Female | 124612 (106381 to 145816) | 5.76 (4.92 to 6.74) |  | 228490 (197181 to 262387) | 5.28 (4.57 to 6.05) | -0.46 (-0.89 to -0.03) |
| Male | 1546240 (1281821 to 1923428) | 78.54 (65.35 to 96.73) |  | 2844060 (2389878 to 3356589) | 71.36 (60.05 to 83.86) | -0.4 (-0.74 to -0.06) |
| **SDI** |  |  |  |  |  |  |
| High SDI | 151031 (129163 to 180327) | 14.43 (12.37 to 17.34) |  | 202751 (181972 to 226851) | 11.02 (9.87 to 12.34) | -1.33 (-1.67 to -0.98) |
| High-middle SDI | 600930 (498055 to 736263) | 54.81 (45.56 to 66.99) |  | 1050750 (885843 to 1243306) | 51.56 (43.47 to 60.99) | -0.33 (-0.68 to 0.02) |
| Middle SDI | 783362 (642056 to 998726) | 68.27 (56.37 to 85.79) |  | 1537554 (1280809 to 1829408) | 58.47 (48.81 to 69.34) | -0.56 (-0.96 to -0.16) |
| Low-middle SDI | 125995 (106317 to 148430) | 18.98 (16.05 to 22.14) |  | 261386 (222630 to 307298) | 18.26 (15.59 to 21.45) | -0.24 (-0.39 to -0.09) |
| Low SDI | 9442 (8101 to 10977) | 3.92 (3.36 to 4.55) |  | 19976 (17239 to 23255) | 3.85 (3.32 to 4.49) | -0.1 (-0.23 to 0.02) |
| **Region** |  |  |  |  |  |  |
| High-income Asia Pacific | 63288 (51216 to 80082) | 32.05 (26.01 to 40.22) |  | 59303 (49820 to 70231) | 11.63 (9.82 to 13.82) | -3.97 (-4.48 to -3.45) |
| High-income North America | 40654 (34460 to 48023) | 11.48 (9.76 to 13.57) |  | 52018 (45759 to 59068) | 8.34 (7.37 to 9.4) | -1.11 (-1.42 to -0.8) |
| Western Europe | 69186 (55030 to 89801) | 11.57 (9.21 to 15.14) |  | 38197 (33391 to 44339) | 4.07 (3.56 to 4.69) | -3.79 (-4.17 to -3.41) |
| Australasia | 666 (561 to 783) | 2.76 (2.34 to 3.24) |  | 1659 (1356 to 2152) | 3.14 (2.59 to 4.06) | 0.21 (-0.23 to 0.65) |
| Andean Latin America | 681 (561 to 880) | 3.33 (2.72 to 4.31) |  | 2730 (2201 to 3344) | 5.01 (4.04 to 6.16) | 1.18 (0.75 to 1.62) |
| Tropical Latin America | 22563 (19098 to 28051) | 21.22 (17.86 to 26.3) |  | 50628 (42560 to 61458) | 20.46 (17.22 to 24.76) | -0.35 (-0.82 to 0.11) |
| Central Latin America | 15062 (12646 to 17695) | 17.27 (14.38 to 20.55) |  | 29621 (25295 to 34775) | 12.31 (10.52 to 14.47) | -1.2 (-1.49 to -0.9) |
| Southern Latin America | 1952 (1588 to 2427) | 4.22 (3.44 to 5.24) |  | 6291 (5049 to 7905) | 7.31 (5.88 to 9.16) | 1.67 (1.3 to 2.04) |
| Caribbean | 167 (135 to 206) | 0.62 (0.5 to 0.77) |  | 351 (281 to 440) | 0.68 (0.55 to 0.86) | 0.29 (0.2 to 0.37) |
| Central Europe | 22052 (18277 to 27025) | 14.86 (12.36 to 18.25) |  | 24659 (21002 to 28896) | 12.11 (10.35 to 14.12) | -0.85 (-1.01 to -0.69) |
| Eastern Europe | 24787 (20583 to 30090) | 8.94 (7.46 to 10.8) |  | 28418 (23528 to 34452) | 8.52 (7.08 to 10.32) | -0.22 (-0.41 to -0.04) |
| Central Asia | 1448 (1190 to 1761) | 3.2 (2.62 to 3.91) |  | 3245 (2643 to 3978) | 4.83 (3.92 to 5.99) | 1.31 (1.16 to 1.45) |
| North Africa and Middle East | 3343 (2572 to 4263) | 1.58 (1.26 to 1.96) |  | 12474 (10005 to 15275) | 2.57 (2.12 to 3.08) | 1.49 (1.16 to 1.83) |
| South Asia | 39831 (32941 to 47883) | 6.98 (5.83 to 8.37) |  | 88996 (75029 to 107476) | 6.29 (5.33 to 7.55) | -0.39 (-0.5 to -0.29) |
| Southeast Asia | 12352 (9867 to 15629) | 4.18 (3.34 to 5.23) |  | 36460 (28596 to 45989) | 5.56 (4.35 to 6.99) | 1.05 (0.89 to 1.21) |
| East Asia | 1343929 (1095621 to 1690162) | 139.26 (114.08 to 173.86) |  | 2617455 (2180162 to 3115778) | 120.41 (100.75 to 142.96) | -0.57 (-0.94 to -0.2) |
| Oceania | 139 (110 to 174) | 4.61 (3.72 to 5.71) |  | 474 (369 to 601) | 6.68 (5.24 to 8.34) | 1.28 (1.17 to 1.38) |
| Western Sub-Saharan Africa | 1246 (1024 to 1511) | 1.25 (1.05 to 1.49) |  | 2054 (1699 to 2439) | 0.97 (0.82 to 1.14) | -0.85 (-0.91 to -0.78) |
| Eastern Sub-Saharan Africa | 2511 (2157 to 2919) | 3.4 (2.91 to 3.96) |  | 4906 (4167 to 5746) | 3.21 (2.73 to 3.78) | -0.24 (-0.41 to -0.07) |
| Central Sub-Saharan Africa | 678 (578 to 794) | 3.37 (2.89 to 3.9) |  | 1827 (1564 to 2120) | 4.01 (3.43 to 4.65) | 0.53 (0.24 to 0.83) |
| Southern Sub-Saharan Africa | 4317 (3671 to 5052) | 15.86 (13.53 to 18.51) |  | 10785 (9193 to 12576) | 18.99 (16.34 to 22.12) | 0.44 (0.18 to 0.7) |
| **Countries** |  |  |  |  |  |  |
| Afghanistan | 106 (84 to 132) | 1.41 (1.14 to 1.74) |  | 592 (442 to 778) | 2.88 (2.28 to 3.56) | 2.48 (2.12 to 2.85) |
| Albania | 310 (252 to 387) | 14.29 (11.66 to 17.5) |  | 700 (553 to 897) | 16.45 (13.14 to 20.94) | 0.61 (0.2 to 1.03) |
| Algeria | 52 (41 to 66) | 0.41 (0.34 to 0.51) |  | 231 (192 to 280) | 0.72 (0.61 to 0.87) | 2.03 (1.85 to 2.2) |
| American Samoa | 1 (1 to 1) | 3.96 (3.16 to 5.06) |  | 3 (2 to 4) | 6.29 (4.86 to 7.98) | 1.58 (1.47 to 1.69) |
| Andorra | 0 (0 to 0) | 0.15 (0.12 to 0.2) |  | 0 (0 to 0) | 0.12 (0.1 to 0.16) | -1.15 (-1.76 to -0.53) |
| Angola | 115 (97 to 135) | 3.31 (2.84 to 3.87) |  | 350 (294 to 410) | 3.7 (3.11 to 4.33) | 0.31 (0.16 to 0.47) |
| Antigua and Barbuda | 1 (0 to 1) | 1.05 (0.83 to 1.33) |  | 1 (1 to 1) | 0.88 (0.69 to 1.09) | -0.72 (-1.03 to -0.41) |
| Argentina | 720 (559 to 918) | 2.24 (1.75 to 2.84) |  | 2077 (1626 to 2683) | 3.74 (2.94 to 4.83) | 1.76 (1.7 to 1.83) |
| Armenia | 100 (83 to 120) | 3.74 (3.1 to 4.5) |  | 204 (165 to 256) | 4.89 (3.99 to 6.15) | 0.94 (0.85 to 1.02) |
| Australia | 404 (334 to 485) | 2.01 (1.68 to 2.41) |  | 1164 (926 to 1596) | 2.58 (2.06 to 3.52) | 0.96 (0.46 to 1.47) |
| Austria | 66 (53 to 81) | 0.56 (0.45 to 0.69) |  | 140 (110 to 178) | 0.78 (0.62 to 0.99) | 1.2 (0.84 to 1.57) |
| Azerbaijan | 99 (76 to 126) | 2.08 (1.6 to 2.68) |  | 300 (235 to 381) | 3.53 (2.77 to 4.54) | 1.88 (1.76 to 1.99) |
| Bahamas | 1 (1 to 1) | 0.68 (0.54 to 0.86) |  | 3 (2 to 3) | 0.7 (0.55 to 0.86) | 0.01 (-0.15 to 0.18) |
| Bahrain | 3 (2 to 4) | 1.01 (0.84 to 1.21) |  | 14 (11 to 18) | 1.26 (1.05 to 1.5) | 0.3 (-1.08 to 1.71) |
| Bangladesh | 1612 (1330 to 1954) | 3.52 (2.89 to 4.25) |  | 5475 (4381 to 6748) | 4.39 (3.51 to 5.37) | 0.77 (0.53 to 1.02) |
| Barbados | 2 (1 to 2) | 0.54 (0.42 to 0.69) |  | 3 (2 to 4) | 0.63 (0.49 to 0.81) | 0.49 (0.41 to 0.57) |
| Belarus | 768 (624 to 938) | 6.02 (4.89 to 7.34) |  | 1177 (944 to 1444) | 7.67 (6.17 to 9.46) | 0.84 (0.79 to 0.89) |
| Belgium | 355 (300 to 416) | 2.22 (1.88 to 2.61) |  | 110 (90 to 134) | 0.42 (0.35 to 0.51) | -6.32 (-7.11 to -5.53) |
| Belize | 1 (1 to 1) | 0.72 (0.59 to 0.89) |  | 3 (2 to 3) | 0.85 (0.69 to 1.04) | 0.46 (0.27 to 0.65) |
| Benin | 20 (17 to 24) | 0.91 (0.76 to 1.1) |  | 53 (43 to 63) | 0.96 (0.81 to 1.14) | 0.18 (0.12 to 0.24) |
| Bermuda | 1 (1 to 1) | 1.17 (0.98 to 1.41) |  | 1 (1 to 1) | 0.77 (0.62 to 0.96) | -1.56 (-1.68 to -1.43) |
| Bhutan | 6 (5 to 8) | 2.69 (2.23 to 3.31) |  | 21 (17 to 26) | 3.97 (3.23 to 4.81) | 1.24 (1.1 to 1.37) |
| Bolivia (Plurinational State of) | 89 (71 to 122) | 2.77 (2.2 to 3.8) |  | 388 (307 to 495) | 4.61 (3.66 to 5.88) | 1.67 (1.41 to 1.93) |
| Bosnia and Herzegovina | 231 (184 to 289) | 5.5 (4.46 to 6.78) |  | 541 (428 to 674) | 9.45 (7.57 to 11.78) | 1.93 (1.81 to 2.04) |
| Botswana | 60 (49 to 77) | 10.72 (8.84 to 13.65) |  | 149 (123 to 177) | 11.61 (9.68 to 13.73) | -0.05 (-0.41 to 0.3) |
| Brazil | 22221 (18797 to 27665) | 21.41 (18 to 26.55) |  | 49744 (41788 to 60362) | 20.6 (17.33 to 24.95) | -0.37 (-0.83 to 0.1) |
| Brunei Darussalam | 3 (2 to 4) | 3.71 (2.99 to 4.65) |  | 9 (8 to 12) | 4.16 (3.31 to 5.28) | 0.51 (0.35 to 0.67) |
| Bulgaria | 2128 (1761 to 2588) | 16.68 (13.85 to 20.16) |  | 1833 (1499 to 2211) | 13.13 (10.76 to 15.91) | -0.99 (-1.36 to -0.62) |
| Burkina Faso | 48 (39 to 58) | 1.01 (0.84 to 1.22) |  | 86 (70 to 104) | 0.86 (0.72 to 1.03) | -0.6 (-0.7 to -0.49) |
| Burundi | 91 (77 to 106) | 3.87 (3.28 to 4.51) |  | 154 (127 to 185) | 3.83 (3.14 to 4.58) | -0.3 (-0.6 to 0.01) |
| Cabo Verde | 1 (1 to 2) | 0.55 (0.45 to 0.66) |  | 3 (2 to 3) | 0.64 (0.52 to 0.77) | 0.41 (0.25 to 0.57) |
| Cambodia | 116 (86 to 153) | 2.35 (1.73 to 3.08) |  | 494 (363 to 652) | 3.9 (2.9 to 5.11) | 1.82 (1.73 to 1.92) |
| Cameroon | 66 (54 to 82) | 1.3 (1.08 to 1.58) |  | 175 (143 to 212) | 1.24 (1.04 to 1.48) | -0.23 (-0.33 to -0.13) |
| Canada | 3069 (2538 to 3723) | 9.29 (7.72 to 11.15) |  | 5705 (4761 to 6855) | 8.3 (6.97 to 9.9) | -0.41 (-0.54 to -0.27) |
| Central African Republic | 39 (33 to 45) | 3.65 (3.11 to 4.23) |  | 74 (63 to 87) | 3.94 (3.37 to 4.59) | 0.12 (0.01 to 0.22) |
| Chad | 27 (22 to 32) | 0.88 (0.73 to 1.06) |  | 55 (45 to 67) | 0.9 (0.74 to 1.07) | 0.04 (0 to 0.08) |
| Chile | 1155 (938 to 1427) | 11.97 (9.75 to 14.76) |  | 4018 (3168 to 5129) | 16.61 (13.1 to 21.2) | 0.86 (0.32 to 1.4) |
| China | 1330860 (1083676 to 1672573) | 143.1 (117.11 to 178.64) |  | 2587176 (2153538 to 3082660) | 123.12 (102.96 to 146.36) | -0.59 (-0.96 to -0.21) |
| Colombia | 940 (779 to 1126) | 5.12 (4.28 to 6.07) |  | 2737 (2231 to 3398) | 5.2 (4.23 to 6.45) | 0.02 (-0.14 to 0.18) |
| Comoros | 6 (6 to 8) | 2.98 (2.54 to 3.47) |  | 15 (13 to 18) | 3.23 (2.72 to 3.81) | 0.11 (-0.09 to 0.31) |
| Congo | 40 (34 to 47) | 3.9 (3.37 to 4.5) |  | 103 (88 to 120) | 4.4 (3.77 to 5.14) | 0.42 (0.22 to 0.62) |
| Cook Islands | 1 (0 to 1) | 4.09 (3.16 to 5.3) |  | 2 (1 to 2) | 6.08 (4.66 to 7.82) | 1.43 (1.29 to 1.58) |
| Costa Rica | 62 (50 to 78) | 3.24 (2.59 to 4.08) |  | 221 (179 to 277) | 4.29 (3.46 to 5.38) | 0.96 (0.92 to 1) |
| Croatia | 596 (490 to 726) | 9.39 (7.76 to 11.41) |  | 915 (742 to 1104) | 11.46 (9.38 to 13.86) | 0.69 (0.64 to 0.74) |
| Cuba | 58 (45 to 73) | 0.56 (0.43 to 0.7) |  | 119 (92 to 152) | 0.65 (0.51 to 0.83) | 0.52 (0.47 to 0.56) |
| Cyprus | 1 (1 to 1) | 0.12 (0.1 to 0.16) |  | 1 (1 to 2) | 0.09 (0.07 to 0.12) | -1.28 (-2.21 to -0.34) |
| Czechia | 804 (633 to 1063) | 5.92 (4.66 to 7.76) |  | 1711 (1372 to 2094) | 8.78 (6.98 to 10.95) | 1.38 (1.04 to 1.72) |
| Côte d’Ivoire | 63 (50 to 78) | 1.24 (1.01 to 1.5) |  | 156 (125 to 190) | 1.22 (1.02 to 1.45) | -0.05 (-0.17 to 0.08) |
| Democratic People's Republic of Korea | 6928 (5440 to 12604) | 38.36 (30.45 to 67.58) |  | 20522 (16087 to 28148) | 61.5 (48.34 to 83.93) | 1.07 (0.54 to 1.6) |
| Democratic Republic of the Congo | 461 (390 to 541) | 3.35 (2.84 to 3.9) |  | 1249 (1064 to 1452) | 4.08 (3.48 to 4.76) | 0.58 (0.42 to 0.73) |
| Denmark | 15 (12 to 18) | 0.18 (0.14 to 0.23) |  | 11 (8 to 14) | 0.1 (0.07 to 0.14) | -2.38 (-2.89 to -1.87) |
| Djibouti | 4 (3 to 4) | 2.93 (2.46 to 3.45) |  | 19 (16 to 23) | 3.64 (3.01 to 4.33) | 0.56 (0.38 to 0.75) |
| Dominica | 0 (0 to 0) | 0.55 (0.43 to 0.7) |  | 1 (0 to 1) | 0.66 (0.52 to 0.84) | 0.64 (0.6 to 0.68) |
| Dominican Republic | 22 (17 to 28) | 0.53 (0.41 to 0.68) |  | 59 (46 to 74) | 0.62 (0.49 to 0.79) | 0.52 (0.5 to 0.54) |
| Ecuador | 113 (92 to 143) | 2.1 (1.69 to 2.64) |  | 510 (415 to 624) | 3.44 (2.79 to 4.21) | 1.87 (1.64 to 2.11) |
| Egypt | 148 (117 to 183) | 0.47 (0.38 to 0.56) |  | 616 (494 to 746) | 0.96 (0.79 to 1.14) | 2.54 (2.33 to 2.76) |
| El Salvador | 100 (80 to 125) | 3.21 (2.54 to 4.02) |  | 236 (190 to 298) | 3.95 (3.17 to 5.01) | 0.73 (0.68 to 0.77) |
| Equatorial Guinea | 6 (5 to 7) | 3.43 (2.96 to 3.99) |  | 14 (12 to 17) | 3.44 (2.93 to 4.04) | -0.11 (-0.32 to 0.09) |
| Eritrea | 33 (28 to 39) | 3.24 (2.75 to 3.8) |  | 89 (75 to 105) | 3.48 (2.94 to 4.07) | 0.13 (0.01 to 0.26) |
| Estonia | 106 (85 to 131) | 5.3 (4.27 to 6.56) |  | 181 (143 to 225) | 7.59 (6.01 to 9.55) | 1.26 (1.17 to 1.35) |
| Eswatini | 42 (35 to 53) | 14.92 (12.46 to 18.88) |  | 84 (70 to 102) | 14.86 (12.54 to 18.1) | 0.04 (-0.44 to 0.51) |
| Ethiopia | 895 (758 to 1056) | 4.15 (3.53 to 4.88) |  | 1180 (1002 to 1399) | 2.88 (2.45 to 3.42) | -1.34 (-1.41 to -1.26) |
| Fiji | 15 (11 to 19) | 3.97 (3.11 to 5.16) |  | 44 (33 to 57) | 5.76 (4.38 to 7.39) | 1.31 (1.22 to 1.39) |
| Finland | 20 (16 to 24) | 0.28 (0.23 to 0.34) |  | 30 (24 to 37) | 0.25 (0.2 to 0.31) | 0.07 (-0.73 to 0.87) |
| France | 797 (666 to 959) | 0.92 (0.77 to 1.1) |  | 563 (455 to 693) | 0.36 (0.29 to 0.44) | -4.21 (-5.62 to -2.77) |
| Gabon | 17 (15 to 20) | 3.19 (2.74 to 3.73) |  | 37 (31 to 43) | 3.82 (3.23 to 4.5) | 0.52 (0.44 to 0.59) |
| Gambia | 4 (3 to 5) | 1.03 (0.85 to 1.24) |  | 11 (9 to 13) | 1.05 (0.89 to 1.25) | -0.02 (-0.15 to 0.12) |
| Georgia | 126 (98 to 163) | 2.07 (1.62 to 2.63) |  | 242 (197 to 298) | 3.99 (3.26 to 4.86) | 2.41 (2.25 to 2.58) |
| Germany | 623 (500 to 775) | 0.48 (0.38 to 0.59) |  | 609 (479 to 770) | 0.28 (0.22 to 0.34) | -2.45 (-3.48 to -1.41) |
| Ghana | 116 (92 to 143) | 1.53 (1.26 to 1.87) |  | 179 (145 to 216) | 0.99 (0.83 to 1.17) | -1.55 (-1.74 to -1.35) |
| Greece | 9 (6 to 12) | 0.07 (0.05 to 0.1) |  | 11 (8 to 15) | 0.07 (0.04 to 0.09) | -0.11 (-0.47 to 0.25) |
| Greenland | 3 (3 to 4) | 10.92 (8.51 to 13.9) |  | 6 (4 to 7) | 8.49 (6.65 to 10.72) | -1.14 (-1.96 to -0.31) |
| Grenada | 1 (1 to 1) | 0.92 (0.77 to 1.1) |  | 1 (1 to 1) | 0.84 (0.69 to 1.04) | -0.51 (-0.73 to -0.29) |
| Guam | 3 (2 to 4) | 3.99 (3.18 to 5.11) |  | 12 (9 to 15) | 6.05 (4.63 to 7.72) | 1.44 (1.32 to 1.57) |
| Guatemala | 153 (122 to 188) | 3.75 (3.05 to 4.62) |  | 478 (384 to 590) | 4.03 (3.23 to 4.96) | 0.12 (-0.14 to 0.37) |
| Guinea | 36 (29 to 43) | 1.01 (0.84 to 1.22) |  | 71 (58 to 85) | 1.16 (0.97 to 1.37) | 0.42 (0.35 to 0.5) |
| Guinea-Bissau | 8 (6 to 10) | 1.65 (1.35 to 1.99) |  | 13 (10 to 16) | 1.42 (1.18 to 1.72) | -0.51 (-0.59 to -0.43) |
| Guyana | 3 (2 to 4) | 0.7 (0.56 to 0.87) |  | 5 (4 to 6) | 0.73 (0.59 to 0.92) | 0.11 (0.04 to 0.19) |
| Haiti | 25 (20 to 31) | 0.7 (0.58 to 0.86) |  | 62 (50 to 77) | 0.82 (0.67 to 1.02) | 0.48 (0.37 to 0.6) |
| Honduras | 83 (68 to 102) | 3.67 (3.02 to 4.49) |  | 284 (228 to 349) | 4.44 (3.62 to 5.48) | 0.62 (0.54 to 0.69) |
| Hungary | 1380 (1114 to 1681) | 9.47 (7.66 to 11.53) |  | 2283 (1846 to 2807) | 12.57 (10.16 to 15.45) | 0.9 (0.48 to 1.32) |
| Iceland | 0 (0 to 0) | 0.07 (0.04 to 0.1) |  | 0 (0 to 0) | 0.06 (0.03 to 0.09) | -0.65 (-0.9 to -0.39) |
| India | 35898 (29665 to 43411) | 7.98 (6.65 to 9.56) |  | 79358 (66736 to 96189) | 6.87 (5.82 to 8.3) | -0.55 (-0.65 to -0.45) |
| Indonesia | 7827 (5933 to 10751) | 6.66 (5.08 to 8.87) |  | 18865 (15028 to 24300) | 7.74 (6.21 to 9.78) | 0.6 (0.2 to 1) |
| Iran (Islamic Republic of) | 575 (457 to 711) | 1.75 (1.46 to 2.08) |  | 5749 (4497 to 7201) | 6.93 (5.57 to 8.46) | 4.7 (4.12 to 5.28) |
| Iraq | 121 (98 to 147) | 1.3 (1.08 to 1.55) |  | 249 (202 to 300) | 1.05 (0.89 to 1.25) | -0.96 (-1.25 to -0.68) |
| Ireland | 3 (2 to 5) | 0.09 (0.06 to 0.12) |  | 6 (4 to 8) | 0.08 (0.06 to 0.12) | -0.33 (-0.74 to 0.08) |
| Israel | 3 (2 to 5) | 0.07 (0.05 to 0.1) |  | 6 (4 to 9) | 0.06 (0.04 to 0.09) | -0.45 (-0.61 to -0.3) |
| Italy | 58800 (45508 to 78288) | 63.64 (49.34 to 85.01) |  | 26559 (22575 to 31730) | 18.06 (15.36 to 21.21) | -4.5 (-4.89 to -4.1) |
| Jamaica | 16 (13 to 20) | 0.87 (0.7 to 1.09) |  | 19 (15 to 25) | 0.63 (0.48 to 0.81) | -1.48 (-2.18 to -0.78) |
| Japan | 60886 (48912 to 77310) | 35.77 (28.87 to 45.19) |  | 52224 (44054 to 61549) | 12.79 (10.81 to 15.2) | -4.02 (-4.55 to -3.49) |
| Jordan | 20 (15 to 26) | 1.02 (0.83 to 1.23) |  | 59 (47 to 73) | 0.78 (0.64 to 0.94) | -0.97 (-1.16 to -0.77) |
| Kazakhstan | 647 (527 to 794) | 5.31 (4.32 to 6.52) |  | 1152 (934 to 1463) | 7.02 (5.65 to 8.84) | 0.54 (0.03 to 1.05) |
| Kenya | 297 (255 to 348) | 3.76 (3.2 to 4.41) |  | 770 (659 to 901) | 3.64 (3.11 to 4.3) | -0.26 (-0.48 to -0.03) |
| Kiribati | 2 (2 to 3) | 5.36 (4.2 to 7.13) |  | 5 (4 to 7) | 7.84 (6.07 to 10.49) | 1.19 (1.03 to 1.36) |
| Kuwait | 5 (4 to 7) | 0.55 (0.45 to 0.66) |  | 33 (25 to 41) | 0.91 (0.75 to 1.09) | 1.47 (0.95 to 2) |
| Kyrgyzstan | 62 (49 to 80) | 2.09 (1.62 to 2.7) |  | 147 (116 to 188) | 3.49 (2.75 to 4.53) | 1.83 (1.71 to 1.94) |
| Lao People's Democratic Republic | 58 (42 to 79) | 2.56 (1.89 to 3.46) |  | 211 (154 to 281) | 4.49 (3.3 to 6.01) | 1.99 (1.92 to 2.07) |
| Latvia | 290 (225 to 388) | 8.22 (6.37 to 10.98) |  | 258 (205 to 322) | 7.45 (5.77 to 9.35) | -0.32 (-0.44 to -0.21) |
| Lebanon | 12 (10 to 15) | 0.53 (0.43 to 0.63) |  | 36 (30 to 43) | 0.69 (0.58 to 0.83) | 1 (0.76 to 1.24) |
| Lesotho | 131 (108 to 173) | 13.51 (11.2 to 17.63) |  | 262 (216 to 324) | 20.64 (17.17 to 25.41) | 1.23 (0.96 to 1.49) |
| Liberia | 10 (8 to 12) | 0.87 (0.72 to 1.04) |  | 21 (17 to 25) | 0.91 (0.76 to 1.08) | 0.21 (0.05 to 0.37) |
| Libya | 10 (8 to 13) | 0.48 (0.4 to 0.59) |  | 61 (49 to 74) | 1.06 (0.89 to 1.27) | 2.78 (2.6 to 2.97) |
| Lithuania | 355 (297 to 421) | 7.98 (6.7 to 9.48) |  | 492 (406 to 596) | 9.09 (7.5 to 10.94) | 0.27 (0.09 to 0.45) |
| Luxembourg | 2 (1 to 2) | 0.27 (0.21 to 0.35) |  | 1 (1 to 2) | 0.13 (0.1 to 0.17) | -3.17 (-4.08 to -2.25) |
| Madagascar | 162 (134 to 193) | 3.41 (2.82 to 4.05) |  | 346 (282 to 420) | 3.81 (3.12 to 4.62) | 0.23 (-0.06 to 0.51) |
| Malawi | 97 (81 to 114) | 2.66 (2.25 to 3.15) |  | 216 (180 to 256) | 3.16 (2.63 to 3.79) | 0.45 (0.28 to 0.62) |
| Malaysia | 256 (191 to 343) | 2.55 (1.92 to 3.39) |  | 1337 (965 to 1766) | 4.85 (3.52 to 6.37) | 2.24 (2.17 to 2.31) |
| Maldives | 4 (3 to 6) | 4.18 (3.28 to 5.4) |  | 19 (14 to 25) | 5.4 (3.95 to 7.14) | 0.92 (0.85 to 0.99) |
| Mali | 48 (39 to 59) | 1.07 (0.89 to 1.3) |  | 77 (64 to 93) | 0.85 (0.72 to 1.02) | -0.8 (-0.92 to -0.69) |
| Malta | 0 (0 to 1) | 0.12 (0.09 to 0.15) |  | 1 (1 to 1) | 0.1 (0.07 to 0.13) | -1.01 (-1.54 to -0.47) |
| Marshall Islands | 1 (1 to 1) | 4.15 (3.31 to 5.25) |  | 2 (2 to 3) | 6.43 (5.05 to 8.08) | 1.52 (1.46 to 1.58) |
| Mauritania | 15 (12 to 18) | 1.32 (1.1 to 1.59) |  | 21 (17 to 25) | 0.95 (0.79 to 1.12) | -1.17 (-1.37 to -0.97) |
| Mauritius | 18 (14 to 24) | 2.3 (1.72 to 3.02) |  | 79 (56 to 106) | 4.4 (3.18 to 5.93) | 2.3 (2.22 to 2.38) |
| Mexico | 13241 (11018 to 15758) | 29.75 (24.58 to 35.98) |  | 24062 (20355 to 28617) | 19.98 (16.9 to 23.91) | -1.37 (-1.71 to -1.03) |
| Micronesia (Federated States of) | 2 (2 to 2) | 4.18 (3.37 to 5.24) |  | 4 (3 to 6) | 5.98 (4.67 to 7.53) | 1.25 (1.21 to 1.29) |
| Monaco | 0 (0 to 0) | 0.38 (0.3 to 0.47) |  | 0 (0 to 1) | 0.39 (0.31 to 0.48) | -0.46 (-1.67 to 0.76) |
| Mongolia | 35 (28 to 44) | 3.3 (2.62 to 4.14) |  | 84 (68 to 104) | 4.12 (3.33 to 5.12) | 0.78 (0.68 to 0.89) |
| Montenegro | 37 (31 to 46) | 5.93 (4.88 to 7.25) |  | 90 (72 to 110) | 9.44 (7.58 to 11.54) | 1.67 (1.59 to 1.76) |
| Morocco | 65 (51 to 81) | 0.44 (0.36 to 0.53) |  | 254 (206 to 308) | 0.84 (0.69 to 0.99) | 2.23 (2.05 to 2.41) |
| Mozambique | 153 (128 to 181) | 2.73 (2.29 to 3.22) |  | 343 (278 to 427) | 3.39 (2.74 to 4.24) | 0.61 (0.42 to 0.79) |
| Myanmar | 837 (659 to 1066) | 3.14 (2.48 to 3.98) |  | 2124 (1614 to 2747) | 4.32 (3.26 to 5.55) | 1.13 (1.02 to 1.23) |
| Namibia | 79 (67 to 93) | 11 (9.3 to 12.8) |  | 150 (127 to 175) | 10.98 (9.26 to 12.8) | -0.03 (-0.16 to 0.09) |
| Nauru | 0 (0 to 0) | 4.01 (3.15 to 5.22) |  | 0 (0 to 0) | 5.65 (4.39 to 7.11) | 1.28 (1.18 to 1.38) |
| Nepal | 306 (244 to 381) | 3.43 (2.78 to 4.22) |  | 890 (728 to 1086) | 4.23 (3.48 to 5.13) | 0.67 (0.49 to 0.86) |
| Netherlands | 118 (93 to 150) | 0.56 (0.44 to 0.71) |  | 31 (24 to 40) | 0.1 (0.08 to 0.14) | -6.03 (-6.74 to -5.31) |
| New Zealand | 262 (214 to 317) | 6.49 (5.34 to 7.82) |  | 495 (418 to 583) | 6.17 (5.25 to 7.24) | -0.3 (-0.92 to 0.32) |
| Nicaragua | 59 (48 to 73) | 3.5 (2.81 to 4.29) |  | 192 (152 to 242) | 4.1 (3.26 to 5.19) | 0.5 (0.4 to 0.59) |
| Niger | 30 (25 to 36) | 0.93 (0.78 to 1.11) |  | 68 (56 to 81) | 0.81 (0.69 to 0.96) | -0.49 (-0.61 to -0.37) |
| Nigeria | 686 (564 to 830) | 1.37 (1.15 to 1.64) |  | 889 (741 to 1055) | 0.92 (0.77 to 1.08) | -1.37 (-1.58 to -1.15) |
| Niue | 0 (0 to 0) | 3.48 (2.72 to 4.51) |  | 0 (0 to 0) | 5.68 (4.35 to 7.24) | 1.77 (1.68 to 1.87) |
| North Macedonia | 175 (140 to 243) | 9.16 (7.35 to 12.57) |  | 298 (239 to 369) | 9.44 (7.63 to 11.58) | -0.04 (-0.2 to 0.12) |
| Northern Mariana Islands | 1 (1 to 1) | 4.35 (3.44 to 5.61) |  | 3 (2 to 4) | 6.05 (4.6 to 7.8) | 1.19 (0.97 to 1.41) |
| Norway | 728 (587 to 895) | 10.67 (8.67 to 13.04) |  | 699 (571 to 861) | 8.08 (6.62 to 10.07) | -1.09 (-1.28 to -0.9) |
| Oman | 5 (4 to 7) | 0.57 (0.47 to 0.69) |  | 18 (14 to 23) | 0.79 (0.66 to 0.94) | 1.03 (0.82 to 1.23) |
| Pakistan | 2008 (1634 to 2434) | 3.58 (2.91 to 4.36) |  | 3251 (2681 to 3897) | 3 (2.49 to 3.6) | -0.7 (-0.81 to -0.59) |
| Palau | 1 (1 to 1) | 10.28 (8.3 to 12.57) |  | 3 (3 to 4) | 15.02 (12.01 to 18.4) | 0.94 (0.41 to 1.48) |
| Palestine | 5 (4 to 6) | 0.5 (0.41 to 0.6) |  | 14 (11 to 18) | 0.54 (0.45 to 0.66) | 0.36 (0.16 to 0.56) |
| Panama | 53 (43 to 66) | 3.31 (2.65 to 4.07) |  | 176 (141 to 222) | 4.24 (3.4 to 5.35) | 0.89 (0.84 to 0.94) |
| Papua New Guinea | 90 (71 to 113) | 4.84 (3.87 to 5.95) |  | 329 (256 to 416) | 6.94 (5.46 to 8.64) | 1.23 (1.14 to 1.33) |
| Paraguay | 341 (281 to 453) | 13.64 (11.26 to 18.56) |  | 884 (740 to 1061) | 14.88 (12.47 to 17.75) | -0.04 (-0.46 to 0.38) |
| Peru | 479 (390 to 615) | 4.03 (3.25 to 5.19) |  | 1832 (1450 to 2275) | 5.82 (4.59 to 7.24) | 0.99 (0.4 to 1.59) |
| Philippines | 830 (641 to 1074) | 2.5 (1.94 to 3.26) |  | 3170 (2373 to 4217) | 3.73 (2.84 to 4.98) | 1.5 (1.31 to 1.69) |
| Poland | 11240 (9073 to 14610) | 25.73 (20.74 to 33.5) |  | 8970 (7678 to 10485) | 13.44 (11.57 to 15.64) | -2.39 (-2.68 to -2.08) |
| Portugal | 182 (145 to 225) | 1.25 (1 to 1.55) |  | 144 (112 to 186) | 0.59 (0.46 to 0.77) | -3.43 (-4.77 to -2.07) |
| Puerto Rico | 20 (16 to 26) | 0.56 (0.44 to 0.7) |  | 43 (33 to 57) | 0.65 (0.51 to 0.84) | 0.52 (0.48 to 0.57) |
| Qatar | 1 (1 to 1) | 0.44 (0.36 to 0.53) |  | 8 (6 to 11) | 0.62 (0.51 to 0.77) | 1.32 (0.9 to 1.74) |
| Republic of Korea | 2317 (1953 to 2800) | 8.33 (7.03 to 9.98) |  | 6701 (5537 to 8273) | 7.51 (6.23 to 9.26) | -0.69 (-1.28 to -0.1) |
| Republic of Moldova | 230 (182 to 290) | 5.22 (4.18 to 6.51) |  | 432 (343 to 547) | 7.68 (6.06 to 9.77) | 1.44 (1.33 to 1.54) |
| Romania | 3587 (2989 to 4332) | 12.73 (10.66 to 15.22) |  | 4059 (3255 to 4959) | 11.63 (9.35 to 14.3) | -0.49 (-0.74 to -0.24) |
| Russian Federation | 9361 (7623 to 11552) | 5.28 (4.33 to 6.5) |  | 19273 (15919 to 23343) | 8.47 (7 to 10.26) | 1.66 (1.42 to 1.91) |
| Rwanda | 100 (82 to 119) | 3.51 (2.88 to 4.19) |  | 177 (144 to 228) | 3.26 (2.65 to 4.17) | -0.3 (-0.51 to -0.09) |
| Saint Kitts and Nevis | 0 (0 to 0) | 0.68 (0.53 to 0.86) |  | 0 (0 to 1) | 0.68 (0.53 to 0.87) | -0.03 (-0.12 to 0.05) |
| Saint Lucia | 1 (1 to 1) | 0.86 (0.69 to 1.08) |  | 1 (1 to 2) | 0.71 (0.56 to 0.92) | -0.77 (-0.95 to -0.6) |
| Saint Vincent and the Grenadines | 1 (1 to 1) | 0.89 (0.71 to 1.11) |  | 1 (1 to 2) | 1.08 (0.86 to 1.38) | 0.42 (-0.02 to 0.86) |
| Samoa | 3 (3 to 4) | 3.92 (3.08 to 5.05) |  | 9 (7 to 11) | 5.92 (4.54 to 7.57) | 1.43 (1.33 to 1.54) |
| San Marino | 0 (0 to 0) | 0.12 (0.09 to 0.16) |  | 0 (0 to 0) | 0.11 (0.08 to 0.14) | -1 (-1.55 to -0.44) |
| Sao Tome and Principe | 0 (0 to 1) | 0.72 (0.6 to 0.86) |  | 1 (1 to 1) | 0.95 (0.8 to 1.12) | 0.91 (0.81 to 1.02) |
| Saudi Arabia | 29 (22 to 36) | 0.41 (0.34 to 0.51) |  | 130 (102 to 164) | 0.67 (0.55 to 0.82) | 1.63 (1.53 to 1.74) |
| Senegal | 36 (29 to 44) | 1 (0.83 to 1.2) |  | 93 (76 to 111) | 1.12 (0.94 to 1.33) | 0.41 (0.28 to 0.53) |
| Serbia | 556 (450 to 684) | 4.93 (4.01 to 6.04) |  | 1561 (1255 to 1923) | 10.38 (8.43 to 12.69) | 2.61 (2.54 to 2.69) |
| Seychelles | 1 (1 to 2) | 2.37 (1.77 to 3.14) |  | 5 (4 to 7) | 4.51 (3.25 to 6.04) | 2.3 (2.21 to 2.38) |
| Sierra Leone | 16 (14 to 20) | 0.81 (0.68 to 0.97) |  | 41 (34 to 49) | 1.03 (0.86 to 1.21) | 0.86 (0.79 to 0.93) |
| Singapore | 82 (68 to 101) | 4.18 (3.44 to 5.12) |  | 369 (294 to 463) | 5.02 (4.01 to 6.31) | 0.65 (0.54 to 0.75) |
| Slovakia | 678 (538 to 993) | 11.38 (9.09 to 16.45) |  | 1141 (907 to 1409) | 12.71 (10.18 to 15.74) | 0.29 (0.09 to 0.5) |
| Slovenia | 331 (267 to 421) | 13.61 (11.01 to 17.25) |  | 559 (451 to 692) | 14.58 (11.68 to 17.86) | 0.18 (0.02 to 0.34) |
| Solomon Islands | 6 (5 to 8) | 4.6 (3.67 to 5.84) |  | 19 (15 to 24) | 6.29 (4.96 to 7.93) | 1.11 (0.98 to 1.23) |
| Somalia | 80 (66 to 99) | 3.5 (2.86 to 4.35) |  | 217 (176 to 268) | 3.55 (2.88 to 4.42) | -0.04 (-0.44 to 0.37) |
| South Africa | 3812 (3247 to 4448) | 18.31 (15.6 to 21.23) |  | 9745 (8278 to 11372) | 21.5 (18.38 to 25.02) | 0.36 (0.06 to 0.66) |
| South Sudan | 63 (53 to 75) | 2.77 (2.32 to 3.29) |  | 101 (86 to 120) | 3.01 (2.56 to 3.53) | 0.23 (0.11 to 0.35) |
| Spain | 543 (424 to 691) | 0.96 (0.75 to 1.22) |  | 350 (279 to 433) | 0.31 (0.25 to 0.39) | -4.74 (-5.98 to -3.48) |
| Sri Lanka | 346 (264 to 454) | 2.92 (2.22 to 3.85) |  | 1171 (850 to 1567) | 4.51 (3.28 to 6) | 1.53 (1.49 to 1.58) |
| Sudan | 43 (33 to 55) | 0.42 (0.34 to 0.52) |  | 156 (123 to 194) | 0.72 (0.6 to 0.87) | 1.93 (1.81 to 2.05) |
| Suriname | 4 (3 to 5) | 1.59 (1.28 to 2) |  | 6 (5 to 8) | 1.05 (0.82 to 1.36) | -1.65 (-2.41 to -0.89) |
| Sweden | 126 (92 to 250) | 0.75 (0.57 to 1.42) |  | 70 (56 to 90) | 0.34 (0.28 to 0.43) | -2.77 (-3.65 to -1.89) |
| Switzerland | 59 (49 to 72) | 0.53 (0.44 to 0.64) |  | 43 (34 to 54) | 0.25 (0.19 to 0.31) | -2.1 (-2.85 to -1.34) |
| Syrian Arab Republic | 39 (30 to 49) | 0.61 (0.5 to 0.73) |  | 87 (70 to 106) | 0.75 (0.62 to 0.89) | 0.69 (0.49 to 0.89) |
| Taiwan (Province of China) | 6141 (5164 to 7288) | 38.09 (32.23 to 45) |  | 9756 (8614 to 11037) | 24.68 (21.78 to 27.91) | -2.17 (-3.1 to -1.23) |
| Tajikistan | 63 (49 to 80) | 2.31 (1.79 to 2.98) |  | 168 (131 to 213) | 3.83 (2.95 to 4.91) | 1.8 (1.67 to 1.93) |
| Thailand | 1106 (844 to 1467) | 2.75 (2.12 to 3.65) |  | 4828 (3563 to 6472) | 4.65 (3.46 to 6.21) | 1.75 (1.64 to 1.85) |
| Timor-Leste | 9 (6 to 12) | 2.66 (1.95 to 3.61) |  | 39 (28 to 51) | 4.66 (3.45 to 6.11) | 2.01 (1.92 to 2.09) |
| Togo | 16 (13 to 20) | 1.09 (0.91 to 1.3) |  | 42 (34 to 51) | 1.02 (0.86 to 1.21) | -0.24 (-0.32 to -0.16) |
| Tokelau | 0 (0 to 0) | 3.52 (2.78 to 4.47) |  | 0 (0 to 0) | 6.12 (4.69 to 7.88) | 2 (1.89 to 2.1) |
| Tonga | 2 (2 to 3) | 3.99 (3.17 to 5.12) |  | 5 (4 to 6) | 5.71 (4.45 to 7.25) | 1.27 (1.15 to 1.39) |
| Trinidad and Tobago | 5 (4 to 6) | 0.53 (0.41 to 0.68) |  | 11 (9 to 14) | 0.62 (0.48 to 0.8) | 0.51 (0.31 to 0.71) |
| Tunisia | 20 (16 to 25) | 0.4 (0.32 to 0.48) |  | 80 (67 to 97) | 0.69 (0.57 to 0.82) | 1.94 (1.78 to 2.09) |
| Turkey | 2051 (1469 to 2760) | 4.59 (3.44 to 6.02) |  | 3859 (3022 to 4822) | 4.32 (3.42 to 5.37) | -0.47 (-0.83 to -0.11) |
| Turkmenistan | 38 (29 to 49) | 2.08 (1.61 to 2.68) |  | 120 (95 to 152) | 3.37 (2.65 to 4.24) | 1.69 (1.54 to 1.83) |
| Tuvalu | 0 (0 to 0) | 3.24 (2.55 to 4.19) |  | 1 (0 to 1) | 5.62 (4.36 to 7.29) | 1.97 (1.86 to 2.08) |
| Uganda | 175 (145 to 208) | 2.87 (2.4 to 3.42) |  | 377 (317 to 444) | 2.96 (2.47 to 3.49) | -0.01 (-0.21 to 0.2) |
| Ukraine | 13678 (11147 to 17414) | 19.05 (15.61 to 24.17) |  | 6605 (5407 to 8069) | 8.99 (7.35 to 11.01) | -2.83 (-3.25 to -2.41) |
| United Arab Emirates | 4 (3 to 5) | 0.49 (0.4 to 0.59) |  | 38 (29 to 48) | 0.91 (0.76 to 1.09) | 2.08 (1.93 to 2.22) |
| United Kingdom | 6679 (5735 to 7803) | 7.26 (6.26 to 8.51) |  | 8777 (7587 to 10089) | 6.79 (5.89 to 7.75) | -0.51 (-0.73 to -0.29) |
| United Republic of Tanzania | 263 (218 to 314) | 2.5 (2.1 to 2.98) |  | 659 (553 to 787) | 2.88 (2.42 to 3.43) | 0.42 (0.33 to 0.52) |
| United States of America | 37580 (31804 to 44364) | 11.72 (9.94 to 13.83) |  | 46307 (40897 to 52301) | 8.34 (7.39 to 9.38) | -1.31 (-1.55 to -1.07) |
| United States Virgin Islands | 1 (1 to 1) | 0.86 (0.7 to 1.07) |  | 2 (1 to 2) | 1.02 (0.84 to 1.25) | 0.59 (0.39 to 0.8) |
| Uruguay | 78 (58 to 104) | 1.93 (1.46 to 2.55) |  | 196 (146 to 263) | 3.4 (2.52 to 4.56) | 1.96 (1.85 to 2.07) |
| Uzbekistan | 278 (224 to 343) | 2.55 (2.04 to 3.14) |  | 828 (655 to 1045) | 4.77 (3.83 to 5.93) | 2.13 (1.98 to 2.28) |
| Vanuatu | 3 (2 to 4) | 4.4 (3.49 to 5.63) |  | 11 (8 to 14) | 6.32 (4.97 to 8.11) | 1.33 (1.21 to 1.44) |
| Venezuela (Bolivarian Republic of) | 370 (300 to 455) | 3.41 (2.78 to 4.16) |  | 1235 (1008 to 1517) | 4.23 (3.46 to 5.18) | 0.73 (0.7 to 0.76) |
| Viet Nam | 927 (694 to 1245) | 2.22 (1.66 to 2.99) |  | 4071 (2986 to 5444) | 4.08 (3.01 to 5.41) | 2.17 (2.09 to 2.25) |
| Yemen | 27 (21 to 34) | 0.47 (0.39 to 0.57) |  | 176 (136 to 220) | 1.03 (0.84 to 1.22) | 2.79 (2.62 to 2.95) |
| Zambia | 89 (76 to 105) | 3.29 (2.81 to 3.88) |  | 240 (204 to 278) | 3.84 (3.27 to 4.46) | 0.47 (0.3 to 0.63) |
| Zimbabwe | 192 (154 to 241) | 4.91 (3.99 to 6.07) |  | 396 (327 to 471) | 6.05 (5.06 to 7.15) | 0.69 (0.58 to 0.8) |

PNE: pneumoconiosis; AAPC: average annual percent change; UI: uncertainty interval; CI: confidence interval; SDI: Socio-demographic index.

Table S7. Mortality of specific CRDs in 2019 for sexes, SDI and regions, with AAPC (1990-2019).

|  | CRDs | |  | COPD | |  | Asthma | |  | ILD&PS | |  | PNE | |  | Other CRDs | |
| --- | --- | --- | --- | --- | --- | --- | --- | --- | --- | --- | --- | --- | --- | --- | --- | --- | --- |
| GBD data | Age-standardised mortality per 100000 population (95% UI) | AAPC % (95% CI) 1990-2019 |  | Age-standardised mortality per 100000 population (95% UI) | AAPC % (95% CI) 1990-2019 |  | Age-standardised mortality per 100000 population (95% UI) | AAPC % (95% CI) 1990-2019 |  | Age-standardised mortality per 100000 population (95% UI) | AAPC % (95% CI) 1990-2019 |  | Age-standardised mortality per 100000 population (95% UI) | AAPC % (95% CI) 1990-2019 |  | Age-standardised mortality per 100000 population (95% UI) | AAPC % (95% CI) 1990-2019 |
| Global | 51.28 (45.9 to 55.51) | -1.92 (-2 to -1.84) |  | 42.52 (37.63 to 46.31) | -1.93 (-2.03 to -1.84) |  | 5.8 (4.62 to 7.03) | -2.47 (-2.55 to -2.39) |  | 2.17 (1.5 to 2.62) | 0.73 (0.61 to 0.84) |  | 0.29 (0.26 to 0.33) | -2.6 (-2.74 to -2.46) |  | 0.5 (0.39 to 0.59) | -0.65 (-0.8 to -0.49) |
| **Sex** |  |  |  |  |  |  |  |  |  |  |  |  |  |  |  |  |  |
| Female | 39.73 (33.24 to 44.75) | -1.92 (-2 to -1.84) |  | 31.84 (25.95 to 36.46) | -1.99 (-2.08 to -1.9) |  | 5.72 (4.16 to 7.39) | -2.13 (-2.23 to -2.04) |  | 1.76 (1.11 to 2.16) | 1 (0.93 to 1.08) |  | 0.05 (0.04 to 0.05) | -1.14 (-1.29 to -0.99) |  | 0.36 (0.25 to 0.45) | -0.6 (-0.75 to -0.45) |
| Male | 66.72 (60.55 to 73.06) | -1.97 (-2.06 to -1.89) |  | 56.8 (51.17 to 62.51) | -1.95 (-2.04 to -1.85) |  | 5.93 (4.84 to 7.21) | -2.87 (-2.98 to -2.77) |  | 2.72 (1.66 to 3.55) | 0.53 (0.43 to 0.64) |  | 0.6 (0.53 to 0.69) | -2.77 (-2.93 to -2.61) |  | 0.66 (0.49 to 0.8) | -0.77 (-0.95 to -0.59) |
| **SDI** |  |  |  |  |  |  |  |  |  |  |  |  |  |  |  |  |  |
| High SDI | 24.64 (21.49 to 26.07) | -0.73 (-0.8 to -0.66) |  | 20.38 (18.01 to 22.23) | -0.58 (-0.65 to -0.5) |  | 0.96 (0.86 to 1.07) | -4.47 (-4.68 to -4.27) |  | 2.72 (1.52 to 3.5) | 1.35 (1.19 to 1.51) |  | 0.26 (0.23 to 0.3) | -2.47 (-2.68 to -2.26) |  | 0.31 (0.24 to 0.37) | 0.16 (-0.03 to 0.34) |
| High-middle SDI | 33.22 (29.45 to 39.31) | -3.2 (-3.42 to -2.97) |  | 29.76 (26.19 to 35.75) | -3.23 (-3.47 to -3) |  | 1.83 (1.57 to 2.07) | -3.68 (-3.85 to -3.5) |  | 1.14 (0.81 to 1.34) | 0.49 (0.39 to 0.59) |  | 0.23 (0.19 to 0.32) | -3.43 (-3.72 to -3.14) |  | 0.27 (0.24 to 0.34) | -3.25 (-3.42 to -3.08) |
| Middle SDI | 59.82 (52.3 to 66.61) | -2.83 (-2.98 to -2.67) |  | 52.27 (46.05 to 58.7) | -2.86 (-3.04 to -2.69) |  | 5.33 (4.55 to 6.02) | -3.13 (-3.18 to -3.07) |  | 1.44 (1.14 to 1.74) | 0.49 (0.4 to 0.57) |  | 0.33 (0.27 to 0.4) | -2.75 (-2.98 to -2.51) |  | 0.45 (0.34 to 0.52) | -0.09 (-0.17 to 0) |
| Low-middle SDI | 107.29 (90.1 to 120.82) | -1.36 (-1.57 to -1.15) |  | 86.07 (71.71 to 97.03) | -1.27 (-1.44 to -1.09) |  | 16.29 (11.6 to 21.33) | -2.31 (-2.52 to -2.1) |  | 3.75 (2.51 to 5.15) | 0.29 (0.16 to 0.42) |  | 0.31 (0.22 to 0.38) | -2.16 (-2.26 to -2.05) |  | 0.88 (0.63 to 1.1) | -0.42 (-0.46 to -0.38) |
| Low SDI | 87.82 (74.17 to 97.67) | -0.95 (-1.16 to -0.73) |  | 64.43 (54.44 to 73.33) | -0.63 (-0.87 to -0.39) |  | 18.94 (13.35 to 28.66) | -2 (-2.07 to -1.93) |  | 3.24 (2.04 to 4.42) | 0.53 (0.28 to 0.78) |  | 0.26 (0.12 to 0.37) | -1.57 (-1.71 to -1.42) |  | 0.95 (0.65 to 1.2) | -0.28 (-0.38 to -0.19) |
| **Region** |  |  |  |  |  |  |  |  |  |  |  |  |  |  |  |  |  |
| High-income Asia Pacific | 12.31 (10.4 to 13.74) | -2.37 (-2.52 to -2.22) |  | 8.16 (6.36 to 10.38) | -2.02 (-2.2 to -1.83) |  | 0.83 (0.66 to 1.04) | -7.38 (-7.75 to -7.01) |  | 2.87 (1.39 to 3.97) | 1.02 (0.87 to 1.18) |  | 0.28 (0.23 to 0.35) | -2.81 (-3.12 to -2.51) |  | 0.17 (0.14 to 0.23) | -1.18 (-1.28 to -1.08) |
| High-income North America | 36.24 (29.59 to 38.58) | 0.47 (0.41 to 0.53) |  | 31.17 (25.89 to 33.78) | 0.56 (0.5 to 0.63) |  | 0.83 (0.7 to 0.88) | -2.31 (-2.44 to -2.18) |  | 3.59 (2.08 to 4.43) | 1.33 (1.23 to 1.43) |  | 0.19 (0.17 to 0.2) | -2.01 (-2.25 to -1.78) |  | 0.47 (0.3 to 0.52) | 1.34 (1.09 to 1.6) |
| Western Europe | 22.45 (19.62 to 24.03) | -1.11 (-1.21 to -1) |  | 19.25 (17.01 to 21.06) | -0.91 (-1.02 to -0.8) |  | 0.7 (0.61 to 0.79) | -5.08 (-5.29 to -4.88) |  | 2 (1.08 to 2.6) | 1.64 (1.42 to 1.87) |  | 0.26 (0.23 to 0.29) | -3.41 (-3.64 to -3.19) |  | 0.24 (0.21 to 0.35) | -3.7 (-3.94 to -3.45) |
| Australasia | 24.82 (20.92 to 27.5) | -1.33 (-1.44 to -1.21) |  | 20.57 (17.41 to 23.31) | -1.34 (-1.47 to -1.2) |  | 1.14 (0.96 to 1.31) | -4.59 (-4.78 to -4.4) |  | 2.46 (1.1 to 3.58) | 2.6 (2.27 to 2.93) |  | 0.32 (0.27 to 0.39) | 1.84 (1.55 to 2.13) |  | 0.34 (0.27 to 0.42) | -0.87 (-1.04 to -0.7) |
| Andean Latin America | 26.84 (20.79 to 32.1) | -0.74 (-0.97 to -0.51) |  | 15.37 (12.18 to 18.95) | -0.96 (-1.2 to -0.72) |  | 0.95 (0.73 to 1.19) | -5.06 (-5.34 to -4.77) |  | 9.73 (6.72 to 12.47) | 1.08 (0.85 to 1.31) |  | 0.22 (0.16 to 0.3) | -1.98 (-4.06 to 0.14) |  | 0.57 (0.42 to 0.77) | -2.77 (-3.02 to -2.52) |
| Tropical Latin America | 34.1 (30.5 to 38.25) | -1.65 (-1.81 to -1.49) |  | 30.62 (27.19 to 34.65) | -1.68 (-1.85 to -1.51) |  | 1.28 (1.14 to 1.5) | -3.3 (-3.51 to -3.09) |  | 1.44 (0.84 to 1.81) | 1.28 (1.13 to 1.43) |  | 0.21 (0.19 to 0.23) | -0.04 (-0.24 to 0.17) |  | 0.55 (0.42 to 0.66) | -0.08 (-0.21 to 0.05) |
| Central Latin America | 33.69 (28.27 to 38.66) | -0.8 (-0.97 to -0.64) |  | 29.24 (24.87 to 33.71) | -0.44 (-0.69 to -0.19) |  | 1.49 (1.23 to 1.76) | -5.12 (-5.35 to -4.88) |  | 2.18 (1.29 to 2.91) | 1.46 (1.37 to 1.55) |  | 0.11 (0.1 to 0.13) | -3.82 (-4.13 to -3.51) |  | 0.67 (0.44 to 0.84) | 0.37 (-0.08 to 0.82) |
| Southern Latin America | 32.56 (28.24 to 35.95) | -0.04 (-0.36 to 0.28) |  | 26.38 (22.97 to 29.48) | 0.04 (-0.32 to 0.39) |  | 1.07 (0.89 to 1.21) | -3.25 (-3.52 to -2.98) |  | 4.22 (2.88 to 5.05) | 1.46 (1.35 to 1.57) |  | 0.2 (0.17 to 0.23) | -1.48 (-1.98 to -0.97) |  | 0.7 (0.55 to 0.84) | -0.85 (-0.99 to -0.7) |
| Caribbean | 26.87 (22.23 to 31.67) | -0.05 (-0.21 to 0.11) |  | 20.56 (17.16 to 23.89) | 0.4 (0.29 to 0.52) |  | 4.54 (3.43 to 5.83) | -1.84 (-1.99 to -1.68) |  | 1.08 (0.73 to 1.44) | 1.21 (1.08 to 1.33) |  | 0.04 (0.03 to 0.06) | -1.79 (-2.12 to -1.46) |  | 0.65 (0.43 to 0.99) | -0.02 (-0.14 to 0.11) |
| Central Europe | 19.12 (16.63 to 22.05) | -2.22 (-2.37 to -2.06) |  | 17.26 (14.99 to 19.88) | -1.91 (-2.06 to -1.77) |  | 0.81 (0.69 to 0.98) | -5.91 (-6.06 to -5.75) |  | 0.8 (0.64 to 0.99) | -1.11 (-1.26 to -0.96) |  | 0.11 (0.09 to 0.13) | -5.37 (-5.6 to -5.14) |  | 0.14 (0.11 to 0.2) | -2.65 (-2.87 to -2.43) |
| Eastern Europe | 16.23 (14.22 to 20.72) | -2.9 (-3.65 to -2.14) |  | 14.98 (13.12 to 18.89) | -2.8 (-3.5 to -2.1) |  | 0.7 (0.59 to 1.02) | -5.96 (-6.92 to -4.99) |  | 0.33 (0.26 to 0.49) | -2.66 (-2.95 to -2.37) |  | 0.08 (0.07 to 0.1) | -3.42 (-3.88 to -2.96) |  | 0.14 (0.12 to 0.21) | -3.68 (-4.21 to -3.15) |
| Central Asia | 39.41 (35.47 to 46.52) | -0.62 (-1.03 to -0.21) |  | 31.46 (27.94 to 36.79) | -0.34 (-0.79 to 0.12) |  | 2277.44 (1883.33 to 2787.8) | -0.57 (-0.73 to -0.41) |  | 1.5 (1.18 to 1.86) | -0.73 (-0.94 to -0.52) |  | 0.15 (0.12 to 0.19) | 0.09 (-1.01 to 1.21) |  | 0.88 (0.68 to 1.06) | -0.77 (-0.98 to -0.56) |
| North Africa and Middle East | 36.1 (30.9 to 40.31) | -1.38 (-1.51 to -1.25) |  | 26.07 (22.22 to 29.5) | -0.67 (-0.79 to -0.56) |  | 3819.33 (3262.52 to 4512.65) | -0.3 (-0.36 to -0.25) |  | 1.24 (0.88 to 2.04) | 0.16 (-0.04 to 0.36) |  | 0.07 (0.06 to 0.09) | -0.61 (-0.75 to -0.47) |  | 0.32 (0.23 to 0.39) | 0.39 (0.08 to 0.69) |
| South Asia | 118.75 (97.56 to 135.84) | -1.49 (-1.72 to -1.26) |  | 93.21 (74.55 to 107.84) | -1.3 (-1.57 to -1.04) |  | 2443.4 (2029.82 to 2909.76) | -0.3 (-0.7 to 0.1) |  | 5.35 (3.48 to 7.33) | 0.09 (-0.16 to 0.35) |  | 0.27 (0.16 to 0.37) | -2.37 (-2.58 to -2.15) |  | 0.96 (0.67 to 1.24) | -0.76 (-0.89 to -0.64) |
| Southeast Asia | 53.72 (46.49 to 59.45) | -1.83 (-1.92 to -1.74) |  | 38.76 (33.24 to 43.04) | -1.43 (-1.54 to -1.31) |  | 3431.82 (2926.72 to 4059.76) | -0.31 (-0.44 to -0.18) |  | 0.74 (0.41 to 1.48) | -0.14 (-0.22 to -0.06) |  | 0.02 (0.02 to 0.03) | -1.95 (-2.07 to -1.83) |  | 0.41 (0.32 to 0.62) | -0.8 (-0.86 to -0.73) |
| East Asia | 67.48 (57.8 to 82.27) | -4.15 (-4.49 to -3.81) |  | 64.59 (55.33 to 78.95) | -4.16 (-4.51 to -3.82) |  | 2025.52 (1577.43 to 2631.41) | -0.31 (-0.9 to 0.28) |  | 0.46 (0.32 to 0.58) | -0.02 (-0.16 to 0.13) |  | 0.54 (0.44 to 0.71) | -3.02 (-3.33 to -2.72) |  | 0.29 (0.18 to 0.36) | 0 (-0.18 to 0.17) |
| Oceania | 166.28 (133.34 to 202.63) | -0.68 (-0.71 to -0.66) |  | 112.15 (86.8 to 139.9) | -0.47 (-0.5 to -0.44) |  | 4265.16 (3834.97 to 4731.89) | -0.83 (-0.89 to -0.77) |  | 2.74 (1.87 to 3.88) | 0.15 (0.08 to 0.22) |  | 0.28 (0.15 to 0.4) | -0.92 (-1.01 to -0.83) |  | 4.35 (2.08 to 7.04) | 0.45 (0.38 to 0.53) |
| Western Sub-Saharan Africa | 39.14 (33.48 to 44.62) | -1.15 (-1.21 to -1.09) |  | 24.34 (20.52 to 27.78) | -0.67 (-0.77 to -0.57) |  | 3087.32 (2629.03 to 3669.71) | -0.78 (-0.96 to -0.59) |  | 0.66 (0.42 to 0.9) | -1.68 (-1.95 to -1.41) |  | 0.04 (0.03 to 0.05) | -0.52 (-0.7 to -0.34) |  | 0.98 (0.67 to 1.28) | -0.02 (-0.17 to 0.12) |
| Eastern Sub-Saharan Africa | 42.4 (36.86 to 48.27) | -1.39 (-1.43 to -1.35) |  | 29.28 (25.07 to 33.71) | -1.1 (-1.16 to -1.04) |  | 4151.16 (3582.13 to 4898.7) | -0.71 (-0.77 to -0.66) |  | 0.99 (0.4 to 1.63) | -0.36 (-0.43 to -0.3) |  | 0.28 (0.11 to 0.41) | -1.7 (-1.79 to -1.61) |  | 0.56 (0.33 to 0.82) | -0.65 (-0.76 to -0.55) |
| Central Sub-Saharan Africa | 65.8 (44.51 to 104.69) | -0.99 (-1.09 to -0.89) |  | 42.58 (29.63 to 66.67) | -0.75 (-0.82 to -0.68) |  | 3081.67 (2653.81 to 3633.35) | -0.62 (-0.67 to -0.57) |  | 1.53 (0.55 to 3.15) | -0.37 (-0.49 to -0.25) |  | 0.37 (0.21 to 0.59) | -1.18 (-1.32 to -1.03) |  | 0.69 (0.37 to 1.14) | -0.54 (-0.6 to -0.49) |
| Southern Sub-Saharan Africa | 49.21 (44.37 to 54.24) | -0.79 (-1.11 to -0.47) |  | 32.39 (28.97 to 35.37) | -0.41 (-0.65 to -0.17) |  | 3476.21 (2532.37 to 4396.58) | -0.2 (-0.73 to 0.33) |  | 2.01 (1.6 to 2.66) | -0.71 (-0.88 to -0.53) |  | 0.44 (0.39 to 0.49) | -0.54 (-0.97 to -0.11) |  | 0.59 (0.49 to 0.82) | -0.95 (-1.12 to -0.77) |

CRDs: chronic respiratory diseases; COPD: chronic obstructive pulmonary disease; ILD&PS: Interstitial lung disease and pulmonary sarcoidosis; PNE: pneumoconiosis; AAPC: average annual percent change; UI: uncertainty interval; CI: confidence interval; SDI: Socio-demographic index.

Table S8. Mortality of CRDs (1990/2019) for sexes, SDI and locations, with AAPC (1990-2019)

| GBD data | 1990 | |  | 2019 | | AAPC % (95% CI)  1990-2019 |
| --- | --- | --- | --- | --- | --- | --- |
|  | Cases (95% UI) | Age-standardised mortality per  100 000 population (95% UI) |  | Cases (95% UI) | Age-standardised mortality per  100 000 population (95% UI) |  |
| Global | 3093755 (2583800 to 3339171) | 87.89 (73.87 to 95.1) |  | 3974315 (3581757 to 4303823) | 51.28 (45.9 to 55.51) | -1.92 (-2 to -1.84) |
| **Sex** |  |  |  |  |  |  |
| Female | 1360492 (1023327 to 1507670) | 67.84 (51.59 to 75.41) |  | 1740667 (1456918 to 1961313) | 39.73 (33.24 to 44.75) | -1.92 (-2 to -1.84) |
| Male | 1733263 (1508440 to 1884436) | 116.75 (102.61 to 126.76) |  | 2233647 (2029782 to 2452532) | 66.72 (60.55 to 73.06) | -1.97 (-2.06 to -1.89) |
| **SDI** |  |  |  |  |  |  |
| High SDI | 322191 (301748 to 352570) | 30.3 (28.3 to 33.21) |  | 525019 (453715 to 559024) | 24.64 (21.49 to 26.07) | -0.73 (-0.8 to -0.66) |
| High-middle SDI | 744067 (598458 to 810269) | 80.81 (64.52 to 87.92) |  | 651323 (580032 to 772576) | 33.22 (29.45 to 39.31) | -3.2 (-3.42 to -2.97) |
| Middle SDI | 1042205 (817157 to 1147392) | 134.26 (106.75 to 147) |  | 1206232 (1062900 to 1340546) | 59.82 (52.3 to 66.61) | -2.83 (-2.98 to -2.67) |
| Low-middle SDI | 761018 (634082 to 845278) | 160.43 (135.86 to 179.49) |  | 1226245 (1031957 to 1380038) | 107.29 (90.1 to 120.82) | -1.36 (-1.57 to -1.15) |
| Low SDI | 223276 (187251 to 253304) | 114.69 (96.34 to 133.08) |  | 363857 (310934 to 403593) | 87.82 (74.17 to 97.67) | -0.95 (-1.16 to -0.73) |
| **Region** |  |  |  |  |  |  |
| High-income Asia Pacific | 42370 (37231 to 44366) | 24 (20.85 to 25.25) |  | 72143 (59578 to 81146) | 12.31 (10.4 to 13.74) | -2.37 (-2.52 to -2.22) |
| High-income North America | 115443 (107761 to 133643) | 31.29 (29.23 to 36.15) |  | 243409 (196086 to 260316) | 36.24 (29.59 to 38.58) | 0.47 (0.41 to 0.53) |
| Western Europe | 185311 (173116 to 200615) | 31 (28.87 to 33.56) |  | 244861 (210219 to 263528) | 22.45 (19.62 to 24.03) | -1.11 (-1.21 to -1) |
| Australasia | 8508 (7953 to 9006) | 36.52 (33.93 to 38.72) |  | 13599 (11348 to 15144) | 24.82 (20.92 to 27.5) | -1.33 (-1.44 to -1.21) |
| Andean Latin America | 6889 (6131 to 7876) | 33.72 (30.1 to 40.56) |  | 14209 (11012 to 17013) | 26.84 (20.79 to 32.1) | -0.74 (-0.97 to -0.51) |
| Tropical Latin America | 40684 (37275 to 42704) | 54.94 (49.56 to 58.05) |  | 77698 (69788 to 86968) | 34.1 (30.5 to 38.25) | -1.65 (-1.81 to -1.49) |
| Central Latin America | 30365 (27299 to 31656) | 42.34 (37.85 to 44.43) |  | 74582 (62845 to 85574) | 33.69 (28.27 to 38.66) | -0.8 (-0.97 to -0.64) |
| Southern Latin America | 13966 (13014 to 15616) | 32.72 (30.22 to 37.11) |  | 27893 (24150 to 30844) | 32.56 (28.24 to 35.95) | -0.04 (-0.36 to 0.28) |
| Caribbean | 7309 (6432 to 8172) | 28.3 (24.92 to 31.68) |  | 13766 (11412 to 16209) | 26.87 (22.23 to 31.67) | -0.05 (-0.21 to 0.11) |
| Central Europe | 50505 (46626 to 52058) | 36.96 (33.94 to 38.21) |  | 42773 (37191 to 49286) | 19.12 (16.63 to 22.05) | -2.22 (-2.37 to -2.06) |
| Eastern Europe | 102468 (81695 to 109757) | 39.44 (30.85 to 42.36) |  | 56496 (49473 to 71897) | 16.23 (14.22 to 20.72) | -2.9 (-3.65 to -2.14) |
| Central Asia | 21753 (18617 to 22909) | 51.12 (43.53 to 54.07) |  | 22237 (20056 to 26149) | 39.41 (35.47 to 46.52) | -0.62 (-1.03 to -0.21) |
| North Africa and Middle East | 78174 (68863 to 87436) | 53.8 (47.11 to 61.58) |  | 128513 (110781 to 144351) | 36.1 (30.9 to 40.31) | -1.38 (-1.51 to -1.25) |
| South Asia | 748038 (644493 to 838307) | 179.62 (154.45 to 204.39) |  | 1363402 (1125162 to 1563756) | 118.75 (97.56 to 135.84) | -1.49 (-1.72 to -1.26) |
| Southeast Asia | 190752 (151873 to 213268) | 90.37 (70.75 to 102.45) |  | 267847 (233250 to 296152) | 53.72 (46.49 to 59.45) | -1.83 (-1.92 to -1.74) |
| East Asia | 1325881 (975800 to 1485484) | 221.48 (166.31 to 246.45) |  | 1126946 (969591 to 1362127) | 67.48 (57.8 to 82.27) | -4.15 (-4.49 to -3.81) |
| Oceania | 4656 (3880 to 5451) | 201.85 (164.72 to 235.52) |  | 8940 (7142 to 11103) | 166.28 (133.34 to 202.63) | -0.68 (-0.71 to -0.66) |
| Western Sub-Saharan Africa | 43327 (35835 to 50442) | 54.64 (45.14 to 63.75) |  | 64223 (54089 to 74359) | 39.14 (33.48 to 44.62) | -1.15 (-1.21 to -1.09) |
| Eastern Sub-Saharan Africa | 44814 (37876 to 51042) | 63.2 (53.59 to 75.14) |  | 60212 (51794 to 69611) | 42.4 (36.86 to 48.27) | -1.39 (-1.43 to -1.35) |
| Central Sub-Saharan Africa | 16116 (11564 to 21424) | 86.5 (60.43 to 123.44) |  | 26972 (18531 to 40753) | 65.8 (44.51 to 104.69) | -0.99 (-1.09 to -0.89) |
| Southern Sub-Saharan Africa | 16429 (14124 to 19095) | 65.65 (55.94 to 77.28) |  | 23593 (21463 to 26023) | 49.21 (44.37 to 54.24) | -0.79 (-1.11 to -0.47) |
| **Countries** |  |  |  |  |  |  |
| Afghanistan | 5945 (4809 to 7128) | 94.84 (78.02 to 112.96) |  | 7082 (5444 to 8699) | 67.82 (51.99 to 81.33) | -1.11 (-1.21 to -1) |
| Albania | 1006 (695 to 1096) | 59.1 (39.61 to 64.7) |  | 815 (608 to 1053) | 19.54 (14.61 to 25.16) | -3.72 (-4.42 to -3.03) |
| Algeria | 4410 (3378 to 5596) | 53.82 (41.75 to 66.94) |  | 7528 (6030 to 9347) | 30.36 (24.5 to 37.49) | -1.96 (-2.03 to -1.89) |
| American Samoa | 17 (15 to 19) | 96.52 (82.7 to 109.26) |  | 23 (19 to 28) | 57.84 (48.68 to 69.95) | -1.8 (-1.91 to -1.69) |
| Andorra | 16 (12 to 21) | 35.77 (27.88 to 46.21) |  | 39 (31 to 49) | 25.66 (20.11 to 32.13) | -1.17 (-1.27 to -1.08) |
| Angola | 2794 (2084 to 3626) | 82.55 (59.96 to 118.87) |  | 3934 (2886 to 5046) | 46.92 (35.19 to 62.07) | -1.99 (-2.13 to -1.85) |
| Antigua and Barbuda | 7 (6 to 8) | 11.61 (10.33 to 13.08) |  | 11 (9 to 13) | 11.93 (9.71 to 14.2) | 0.25 (-0.52 to 1.02) |
| Argentina | 9894 (9154 to 11385) | 33.02 (30.24 to 38.6) |  | 19348 (16775 to 21811) | 34.85 (30.28 to 39.29) | 0.34 (0.08 to 0.59) |
| Armenia | 1078 (902 to 1154) | 48.39 (39.39 to 52.16) |  | 1323 (1016 to 1581) | 33.77 (25.81 to 40.44) | -1.18 (-1.62 to -0.74) |
| Australia | 6966 (6488 to 7402) | 35.97 (33.28 to 38.3) |  | 11203 (9284 to 12661) | 24.18 (20.3 to 27.12) | -1.32 (-1.43 to -1.21) |
| Austria | 2524 (2339 to 2898) | 20.33 (18.81 to 23.3) |  | 3406 (2914 to 3825) | 16.92 (14.47 to 18.79) | -0.71 (-1.04 to -0.39) |
| Azerbaijan | 1934 (1728 to 2169) | 41.91 (37.35 to 47.59) |  | 2006 (1556 to 3036) | 31.94 (23.85 to 49.48) | -0.65 (-0.93 to -0.37) |
| Bahamas | 24 (21 to 27) | 16.36 (14.42 to 18.53) |  | 51 (41 to 63) | 14.58 (11.67 to 18.06) | -0.36 (-0.55 to -0.17) |
| Bahrain | 86 (74 to 98) | 78.35 (67.14 to 88.92) |  | 161 (129 to 207) | 37.15 (30.47 to 45.41) | -2.98 (-3.63 to -2.32) |
| Bangladesh | 61137 (52166 to 78436) | 147.55 (125.64 to 198.33) |  | 70893 (53727 to 117828) | 62.02 (47.21 to 106.8) | -2.74 (-3.38 to -2.1) |
| Barbados | 40 (35 to 45) | 12.9 (11.42 to 14.62) |  | 63 (50 to 76) | 12.96 (10.4 to 15.57) | -0.08 (-0.46 to 0.31) |
| Belarus | 6648 (4906 to 7316) | 53.71 (39.04 to 59.38) |  | 2438 (1838 to 3910) | 14.98 (11.29 to 24.05) | -4.49 (-5.25 to -3.72) |
| Belgium | 6494 (6017 to 7017) | 41.08 (38.01 to 44.29) |  | 7400 (6282 to 8256) | 27.63 (23.71 to 30.62) | -1.4 (-1.58 to -1.22) |
| Belize | 22 (19 to 25) | 22.42 (19.47 to 25.37) |  | 74 (62 to 87) | 28.82 (24.06 to 33.98) | 1.3 (0.69 to 1.92) |
| Benin | 1340 (1082 to 1589) | 69.2 (55.9 to 82.02) |  | 1888 (1458 to 2449) | 42.45 (34.03 to 53.78) | -1.69 (-1.79 to -1.59) |
| Bermuda | 11 (10 to 12) | 19.04 (16.91 to 21.48) |  | 18 (15 to 22) | 13.21 (10.89 to 15.97) | -1.15 (-1.31 to -0.99) |
| Bhutan | 300 (218 to 394) | 162.86 (120.78 to 216.77) |  | 571 (434 to 781) | 123.17 (94.18 to 164.64) | -0.96 (-1.03 to -0.9) |
| Bolivia (Plurinational State of) | 1856 (1475 to 2221) | 62.07 (51.29 to 73.06) |  | 3578 (2812 to 4445) | 51.07 (40.15 to 63.07) | -0.65 (-0.71 to -0.59) |
| Bosnia and Herzegovina | 1162 (997 to 1244) | 36.31 (30.79 to 39.07) |  | 1204 (953 to 1487) | 20.97 (16.59 to 25.73) | -2.11 (-2.29 to -1.92) |
| Botswana | 463 (335 to 650) | 95.12 (68.85 to 132.98) |  | 649 (481 to 847) | 58.45 (43.86 to 74) | -1.67 (-1.79 to -1.55) |
| Brazil | 40254 (36784 to 42288) | 56.04 (50.36 to 59.18) |  | 76551 (68782 to 85616) | 34.38 (30.76 to 38.59) | -1.7 (-1.85 to -1.54) |
| Brunei Darussalam | 73 (53 to 82) | 128.47 (85.53 to 147.9) |  | 93 (81 to 107) | 64.5 (54.57 to 72.9) | -2.27 (-2.59 to -1.96) |
| Bulgaria | 3786 (3436 to 4056) | 35.16 (31.47 to 37.74) |  | 3036 (2405 to 3809) | 20.51 (16.24 to 25.64) | -1.75 (-2.05 to -1.45) |
| Burkina Faso | 1658 (1350 to 1989) | 41.99 (34.73 to 50.35) |  | 2714 (2232 to 3291) | 31.75 (26.44 to 38.01) | -0.9 (-1.15 to -0.65) |
| Burundi | 2249 (1633 to 2806) | 94.5 (69.75 to 116.48) |  | 2399 (1769 to 3214) | 61.07 (45.92 to 79.19) | -1.54 (-1.67 to -1.41) |
| Cabo Verde | 133 (86 to 152) | 55.5 (36.06 to 63.02) |  | 107 (86 to 145) | 25.59 (20.74 to 34.64) | -2.82 (-3.31 to -2.31) |
| Cambodia | 2738 (2257 to 3164) | 73.54 (59.91 to 85.76) |  | 4959 (3936 to 5791) | 53.76 (42.74 to 62.11) | -1.07 (-1.11 to -1.03) |
| Cameroon | 2630 (2116 to 3139) | 67.97 (55.12 to 80.56) |  | 4166 (3053 to 5526) | 40.02 (30.06 to 51.76) | -1.84 (-1.91 to -1.76) |
| Canada | 9319 (8590 to 10076) | 28.77 (26.44 to 31.03) |  | 18385 (14506 to 20999) | 24.22 (19.52 to 27.56) | -0.62 (-0.72 to -0.53) |
| Central African Republic | 1074 (766 to 1444) | 109.22 (73.88 to 161.94) |  | 1579 (1054 to 2342) | 91.58 (58.78 to 146.99) | -0.59 (-0.66 to -0.52) |
| Chad | 1886 (1454 to 2493) | 69.78 (53.61 to 92.4) |  | 2761 (2143 to 3486) | 52.82 (40.86 to 65.42) | -0.95 (-1.03 to -0.87) |
| Chile | 2781 (2444 to 2956) | 32.28 (28.09 to 34.53) |  | 6389 (4943 to 7227) | 26.87 (20.81 to 30.37) | -0.63 (-0.82 to -0.45) |
| China | 1301224 (955216 to 1458961) | 226.43 (170.16 to 251.43) |  | 1085273 (929797 to 1320157) | 67.98 (57.8 to 83.43) | -4.2 (-4.56 to -3.84) |
| Colombia | 6211 (5414 to 6638) | 41.82 (36.49 to 44.92) |  | 17772 (12952 to 22724) | 31.9 (23.4 to 40.81) | -1.23 (-1.41 to -1.05) |
| Comoros | 129 (80 to 168) | 62.14 (41.96 to 79.47) |  | 167 (131 to 208) | 38.28 (30.52 to 47.36) | -1.6 (-1.79 to -1.41) |
| Congo | 789 (568 to 1089) | 90.3 (63.54 to 132.36) |  | 997 (723 to 1305) | 51.39 (37.85 to 68.2) | -1.88 (-2.03 to -1.74) |
| Cook Islands | 8 (6 to 9) | 71.88 (60.06 to 82.58) |  | 7 (6 to 9) | 32.74 (27.05 to 39.98) | -2.64 (-2.84 to -2.44) |
| Costa Rica | 477 (420 to 526) | 29.57 (25.9 to 32.79) |  | 1425 (1086 to 1794) | 27.59 (21.1 to 34.87) | -0.15 (-0.7 to 0.41) |
| Croatia | 1282 (1173 to 1410) | 21.97 (19.97 to 24.1) |  | 1878 (1467 to 2322) | 19.53 (15.31 to 24.14) | -0.3 (-0.73 to 0.14) |
| Cuba | 1866 (1712 to 1989) | 18.66 (17.09 to 19.98) |  | 4586 (3549 to 5603) | 23.29 (17.9 to 28.42) | 0.99 (0.71 to 1.27) |
| Cyprus | 327 (270 to 374) | 57.95 (46.17 to 66.57) |  | 541 (400 to 626) | 32.53 (22.62 to 37.96) | -1.92 (-2.24 to -1.6) |
| Czechia | 3193 (2968 to 3760) | 23.64 (21.87 to 28) |  | 4062 (3295 to 4918) | 18.4 (14.9 to 22.28) | -0.74 (-1.3 to -0.18) |
| Côte d’Ivoire | 2305 (1790 to 2764) | 67.46 (52.91 to 80.4) |  | 3642 (2763 to 4612) | 40.43 (32.05 to 49.5) | -1.8 (-1.95 to -1.66) |
| Democratic People's Republic of Korea | 19507 (14226 to 25359) | 174.31 (126.7 to 231.33) |  | 31704 (25210 to 37877) | 112.77 (90.31 to 136.52) | -1.52 (-1.59 to -1.46) |
| Democratic Republic of the Congo | 10993 (7447 to 15008) | 86.78 (58.21 to 126.81) |  | 19997 (12900 to 32274) | 71.96 (45.13 to 121.72) | -0.67 (-0.77 to -0.57) |
| Denmark | 3265 (3026 to 3486) | 38.24 (35.28 to 40.64) |  | 4572 (3610 to 5186) | 36 (28.4 to 40.8) | -0.2 (-0.41 to 0) |
| Djibouti | 60 (40 to 84) | 48.45 (32.72 to 63.64) |  | 146 (97 to 209) | 31.66 (21.13 to 44.06) | -1.52 (-1.61 to -1.42) |
| Dominica | 17 (15 to 21) | 23.28 (20.69 to 27.77) |  | 22 (17 to 27) | 24.28 (19.03 to 29.67) | 0.17 (0.08 to 0.25) |
| Dominican Republic | 915 (774 to 1029) | 24.32 (20.63 to 27.38) |  | 1937 (1436 to 2516) | 22.19 (16.46 to 28.7) | 0.01 (-0.3 to 0.31) |
| Ecuador | 1713 (1424 to 1860) | 34.95 (30.21 to 37.58) |  | 3670 (2838 to 4523) | 29.81 (22.53 to 36.57) | -0.45 (-0.78 to -0.12) |
| Egypt | 15225 (13669 to 16935) | 58.83 (53.37 to 68.33) |  | 22560 (16449 to 29261) | 43.58 (32.27 to 55.42) | -0.96 (-1.39 to -0.53) |
| El Salvador | 1042 (885 to 1134) | 33.9 (29.33 to 37.23) |  | 1514 (1116 to 1881) | 23.1 (17.08 to 28.72) | -1.31 (-1.72 to -0.9) |
| Equatorial Guinea | 160 (109 to 223) | 93.43 (61.07 to 142.33) |  | 157 (103 to 262) | 42.84 (28.57 to 74.23) | -2.72 (-2.89 to -2.56) |
| Eritrea | 704 (475 to 908) | 71.55 (49.99 to 94.29) |  | 1117 (784 to 1437) | 49.95 (34.38 to 62.92) | -1.19 (-1.32 to -1.07) |
| Estonia | 332 (308 to 369) | 16.6 (15.35 to 18.52) |  | 303 (238 to 388) | 10.27 (8.01 to 13.09) | -1.6 (-2.35 to -0.85) |
| Eswatini | 251 (185 to 345) | 97.63 (71.75 to 135.29) |  | 319 (238 to 417) | 65.53 (49.9 to 83.65) | -1.42 (-1.59 to -1.25) |
| Ethiopia | 13499 (10819 to 15548) | 71.35 (58.57 to 84.83) |  | 13727 (11713 to 15813) | 38.43 (32.63 to 44.17) | -2.18 (-2.28 to -2.09) |
| Fiji | 400 (320 to 495) | 143.05 (115.79 to 177.69) |  | 412 (321 to 534) | 73.44 (58.69 to 92.71) | -2.37 (-2.65 to -2.09) |
| Finland | 1253 (1163 to 1405) | 17.27 (15.97 to 19.48) |  | 2043 (1736 to 2286) | 14.6 (12.5 to 16.3) | -0.6 (-0.84 to -0.36) |
| France | 22050 (19530 to 24041) | 24.91 (21.98 to 27.12) |  | 22060 (18180 to 26355) | 12.76 (10.79 to 15.11) | -2.32 (-2.52 to -2.12) |
| Gabon | 306 (220 to 386) | 63.62 (44.58 to 82.04) |  | 308 (218 to 400) | 37.08 (26.2 to 47.01) | -1.83 (-1.94 to -1.72) |
| Gambia | 196 (143 to 255) | 62.56 (46.95 to 79.47) |  | 424 (325 to 536) | 48.78 (37.97 to 61.02) | -0.97 (-1.26 to -0.69) |
| Georgia | 1196 (1056 to 1596) | 22.53 (19.89 to 30.48) |  | 1222 (975 to 1675) | 19.36 (15.5 to 26.07) | -0.52 (-0.95 to -0.1) |
| Germany | 39888 (36879 to 45489) | 30.3 (27.96 to 34.48) |  | 45165 (39408 to 51730) | 20.75 (18.24 to 23.49) | -1.42 (-1.73 to -1.11) |
| Ghana | 3076 (1929 to 3784) | 55.71 (35.32 to 68.48) |  | 5938 (3587 to 7459) | 42.08 (25.74 to 51.96) | -0.89 (-0.98 to -0.81) |
| Greece | 2627 (2385 to 2863) | 18.25 (16.37 to 19.85) |  | 6399 (4869 to 7428) | 20.39 (16.17 to 23.24) | 0.23 (-0.17 to 0.64) |
| Greenland | 24 (19 to 27) | 92.77 (73 to 105.6) |  | 32 (24 to 38) | 56.34 (40.93 to 66.46) | -1.67 (-2.07 to -1.27) |
| Grenada | 18 (16 to 20) | 22.34 (19.59 to 25.35) |  | 19 (16 to 22) | 19.1 (16.42 to 21.73) | -0.74 (-1.28 to -0.19) |
| Guam | 30 (27 to 34) | 60.45 (52.33 to 68.12) |  | 53 (43 to 65) | 28.73 (23.49 to 35.23) | -2.81 (-3.29 to -2.33) |
| Guatemala | 1627 (1382 to 1819) | 45.88 (40.55 to 51.28) |  | 2529 (1988 to 3209) | 27.19 (21.66 to 34.14) | -1.91 (-2.43 to -1.38) |
| Guinea | 2215 (1776 to 2770) | 70.92 (56.81 to 89.72) |  | 2787 (2134 to 3446) | 53.06 (41.08 to 65.19) | -0.97 (-1.02 to -0.92) |
| Guinea-Bissau | 370 (293 to 452) | 96.12 (76.33 to 115.38) |  | 386 (297 to 485) | 59.15 (45.8 to 73.57) | -1.65 (-1.72 to -1.58) |
| Guyana | 90 (77 to 104) | 25.58 (22.02 to 29.55) |  | 119 (92 to 148) | 21.57 (16.89 to 26.57) | -0.48 (-1.14 to 0.2) |
| Haiti | 2420 (1642 to 3185) | 72.44 (44.93 to 105.25) |  | 3420 (2266 to 4792) | 54.34 (35 to 77.46) | -0.97 (-1.01 to -0.92) |
| Honduras | 1274 (1036 to 1464) | 57.71 (43.75 to 68.1) |  | 3536 (2229 to 4628) | 68.62 (41.59 to 88.94) | 0.96 (0.56 to 1.36) |
| Hungary | 5385 (5065 to 6151) | 39.1 (36.58 to 44.73) |  | 5936 (4888 to 7093) | 29.7 (24.36 to 35.39) | -0.91 (-1.52 to -0.29) |
| Iceland | 70 (62 to 80) | 23.04 (20.52 to 26.32) |  | 111 (89 to 127) | 17.5 (14.24 to 20.02) | -0.87 (-1.11 to -0.63) |
| India | 601995 (491310 to 683144) | 195.96 (159.48 to 226) |  | 1168381 (899260 to 1363609) | 125.11 (95.2 to 145.25) | -1.61 (-1.9 to -1.32) |
| Indonesia | 65596 (56281 to 74089) | 83.86 (71.34 to 96.81) |  | 100930 (85012 to 113831) | 61.93 (52.48 to 70.18) | -1.05 (-1.19 to -0.91) |
| Iran (Islamic Republic of) | 8207 (7169 to 9874) | 42.64 (36.06 to 52.57) |  | 16835 (14588 to 18193) | 26.93 (23.24 to 29.14) | -1.52 (-1.61 to -1.43) |
| Iraq | 2101 (1714 to 2584) | 28.16 (22.82 to 35.34) |  | 3292 (2577 to 4153) | 17.32 (13.82 to 21.98) | -1.68 (-2.16 to -1.19) |
| Ireland | 2301 (2043 to 2429) | 56.83 (50.44 to 60.09) |  | 2548 (2135 to 2887) | 32.41 (27.25 to 36.7) | -1.91 (-2.08 to -1.73) |
| Israel | 1328 (1222 to 1429) | 29.35 (26.79 to 31.79) |  | 2027 (1763 to 2482) | 16.18 (14.17 to 19.79) | -2.13 (-2.35 to -1.92) |
| Italy | 24263 (22611 to 25175) | 27.24 (25.05 to 28.33) |  | 31345 (26440 to 34246) | 16.76 (14.51 to 18.17) | -1.67 (-1.85 to -1.5) |
| Jamaica | 345 (310 to 392) | 18.45 (16.6 to 21.14) |  | 642 (494 to 800) | 20.73 (15.93 to 26.08) | 0.5 (-0.17 to 1.18) |
| Japan | 29862 (27076 to 31160) | 19.46 (17.52 to 20.39) |  | 55794 (45378 to 63876) | 11.41 (9.61 to 12.91) | -1.87 (-2.02 to -1.72) |
| Jordan | 397 (325 to 469) | 38.78 (31.73 to 46.17) |  | 856 (700 to 1028) | 17.79 (14.57 to 21.48) | -2.71 (-3.14 to -2.29) |
| Kazakhstan | 6905 (6041 to 7412) | 60.49 (52.52 to 65.11) |  | 9772 (8198 to 11527) | 68.18 (56.63 to 80.18) | 0.87 (0.25 to 1.49) |
| Kenya | 3501 (2573 to 5301) | 47.87 (34.9 to 73.98) |  | 7705 (5822 to 10391) | 42.9 (32.45 to 57.47) | -0.39 (-0.47 to -0.31) |
| Kiribati | 58 (47 to 70) | 184.25 (152.41 to 230) |  | 73 (57 to 90) | 144.1 (112.54 to 176.67) | -0.87 (-0.98 to -0.76) |
| Kuwait | 81 (70 to 91) | 17.21 (14.38 to 19.64) |  | 230 (184 to 277) | 12.22 (9.69 to 14.91) | -0.83 (-2.12 to 0.47) |
| Kyrgyzstan | 2658 (2017 to 2937) | 92.09 (69.32 to 102.1) |  | 1513 (1283 to 1889) | 41.08 (34.89 to 51.25) | -3 (-3.57 to -2.41) |
| Lao People's Democratic Republic | 2563 (1993 to 3123) | 135.08 (106.06 to 165.81) |  | 2663 (2092 to 3360) | 76.7 (61.34 to 96.08) | -1.98 (-2.04 to -1.91) |
| Latvia | 750 (695 to 803) | 21.2 (19.63 to 22.64) |  | 433 (350 to 599) | 9.92 (7.94 to 13.53) | -2.51 (-3.25 to -1.76) |
| Lebanon | 695 (564 to 865) | 37.81 (30.6 to 47.38) |  | 1185 (918 to 1499) | 23.35 (18.13 to 29.69) | -1.66 (-1.7 to -1.61) |
| Lesotho | 1000 (674 to 1438) | 114.66 (77.92 to 164.91) |  | 1116 (787 to 1526) | 104.4 (74.25 to 141.45) | -0.22 (-0.45 to 0.01) |
| Liberia | 437 (361 to 521) | 40.93 (34.27 to 49.57) |  | 534 (398 to 715) | 29.75 (23.04 to 39.56) | -0.96 (-1.36 to -0.56) |
| Libya | 559 (433 to 722) | 33.34 (25.32 to 43.46) |  | 1173 (882 to 1477) | 26.61 (19.92 to 33.35) | -0.75 (-1.32 to -0.17) |
| Lithuania | 1473 (1256 to 1568) | 32.82 (27.97 to 34.93) |  | 779 (631 to 1010) | 12.21 (9.9 to 15.8) | -3.38 (-4.1 to -2.66) |
| Luxembourg | 176 (163 to 195) | 32.59 (29.99 to 36.01) |  | 221 (183 to 260) | 19.91 (16.54 to 23.25) | -1.7 (-1.87 to -1.54) |
| Madagascar | 4165 (3657 to 4705) | 78.35 (68.98 to 90.3) |  | 6030 (4601 to 7698) | 65.01 (49.59 to 82.25) | -0.61 (-0.72 to -0.49) |
| Malawi | 1816 (1460 to 2165) | 50.14 (40.9 to 58.85) |  | 2402 (1967 to 2842) | 37.21 (30.74 to 43.72) | -1.01 (-1.07 to -0.94) |
| Malaysia | 5101 (4196 to 6029) | 65.33 (53.95 to 78.15) |  | 7473 (5862 to 10018) | 34.38 (27 to 46.19) | -2.4 (-3.27 to -1.52) |
| Maldives | 96 (73 to 113) | 143.31 (113.82 to 164.18) |  | 123 (102 to 147) | 51.64 (42.42 to 61.43) | -3.74 (-4.04 to -3.43) |
| Mali | 2894 (2199 to 3420) | 74.45 (57.59 to 87.6) |  | 4574 (3389 to 5877) | 56.29 (43.08 to 70.7) | -0.91 (-1 to -0.83) |
| Malta | 106 (96 to 117) | 26.29 (23.72 to 28.86) |  | 157 (132 to 181) | 15.4 (12.97 to 17.65) | -1.98 (-2.12 to -1.84) |
| Marshall Islands | 22 (18 to 30) | 155.34 (124.04 to 214.45) |  | 26 (19 to 35) | 104.71 (77.56 to 136.15) | -1.45 (-1.64 to -1.26) |
| Mauritania | 526 (380 to 644) | 57.35 (41.27 to 70.01) |  | 525 (402 to 670) | 28.76 (22.5 to 36.13) | -2.45 (-2.57 to -2.32) |
| Mauritius | 450 (398 to 482) | 73.83 (64.51 to 79.48) |  | 537 (429 to 678) | 34.9 (28.14 to 44.16) | -2.45 (-2.79 to -2.11) |
| Mexico | 16910 (15104 to 17646) | 49.17 (43.38 to 51.63) |  | 38524 (32632 to 43748) | 36.93 (31.2 to 41.88) | -0.98 (-1.2 to -0.77) |
| Micronesia (Federated States of) | 68 (53 to 84) | 171.54 (133.45 to 219.17) |  | 57 (42 to 73) | 108.37 (84.05 to 136.73) | -1.62 (-1.67 to -1.56) |
| Monaco | 15 (12 to 19) | 18.69 (14.83 to 23.27) |  | 20 (16 to 23) | 17.04 (13.67 to 20.12) | -0.24 (-0.41 to -0.06) |
| Mongolia | 526 (412 to 622) | 56.55 (44.27 to 66.64) |  | 453 (357 to 608) | 27.81 (22.73 to 34.98) | -2.53 (-2.81 to -2.25) |
| Montenegro | 54 (44 to 63) | 9.76 (7.91 to 11.52) |  | 85 (69 to 100) | 9.32 (7.48 to 10.91) | 0.08 (-0.05 to 0.21) |
| Morocco | 4982 (3971 to 6517) | 43.72 (34.6 to 59.43) |  | 9373 (7233 to 11682) | 37.27 (29.08 to 46.8) | -0.66 (-1.25 to -0.06) |
| Mozambique | 2451 (1960 to 2992) | 44.61 (36.64 to 54.55) |  | 3759 (2983 to 4746) | 38.91 (31.11 to 49.16) | -0.46 (-0.6 to -0.33) |
| Myanmar | 37654 (27630 to 46073) | 193.65 (139.34 to 233.07) |  | 43284 (33261 to 50185) | 115.56 (88.11 to 133.27) | -1.79 (-1.87 to -1.72) |
| Namibia | 659 (470 to 905) | 102.83 (74.53 to 142) |  | 791 (614 to 1012) | 64.34 (49.72 to 82.23) | -1.52 (-1.65 to -1.4) |
| Nauru | 5 (4 to 6) | 149.05 (121.07 to 179.52) |  | 3 (2 to 4) | 98.54 (73.89 to 120.02) | -1.44 (-1.54 to -1.34) |
| Nepal | 19609 (15063 to 23557) | 273.02 (213.16 to 326.19) |  | 40800 (30907 to 48383) | 231.2 (175.79 to 270.35) | -0.53 (-0.58 to -0.48) |
| Netherlands | 6934 (6378 to 7449) | 33.47 (30.75 to 35.95) |  | 10766 (8586 to 12240) | 28.62 (22.8 to 32.43) | -0.56 (-0.74 to -0.38) |
| New Zealand | 1542 (1441 to 1659) | 39.27 (36.49 to 42.28) |  | 2396 (2025 to 2686) | 28.28 (24.04 to 31.59) | -1.12 (-1.37 to -0.86) |
| Nicaragua | 430 (380 to 482) | 29.16 (25.49 to 33.87) |  | 1343 (1011 to 1549) | 40.05 (29.96 to 46.03) | 1.1 (0.64 to 1.55) |
| Niger | 1973 (1518 to 2505) | 74.46 (57.15 to 98.42) |  | 3619 (2582 to 4954) | 52.11 (37.99 to 70.27) | -1.19 (-1.31 to -1.06) |
| Nigeria | 17749 (14085 to 21947) | 45.48 (36.02 to 56.61) |  | 24506 (19595 to 30356) | 33.92 (27.06 to 41.73) | -0.99 (-1.08 to -0.9) |
| Niue | 2 (2 to 3) | 95.82 (76.13 to 117.27) |  | 1 (1 to 2) | 58.07 (44.19 to 73.18) | -1.74 (-1.8 to -1.68) |
| North Macedonia | 659 (556 to 726) | 42.89 (35.31 to 47.91) |  | 652 (515 to 836) | 24.72 (19.85 to 31.66) | -2.26 (-3.04 to -1.47) |
| Northern Mariana Islands | 10 (9 to 12) | 81.71 (70.41 to 94.96) |  | 17 (15 to 21) | 49.27 (42.2 to 58.79) | -1.69 (-1.86 to -1.51) |
| Norway | 1523 (1414 to 1785) | 20.52 (19.06 to 23.97) |  | 2771 (2117 to 3026) | 25.85 (19.7 to 28.18) | 0.86 (0.72 to 1) |
| Oman | 185 (138 to 248) | 38.53 (28.77 to 51.86) |  | 244 (191 to 281) | 25.45 (19.45 to 29.63) | -1.51 (-2.02 to -1) |
| Pakistan | 64997 (52751 to 82334) | 130.64 (105.33 to 167.5) |  | 82758 (67880 to 102124) | 100.39 (83.09 to 122.7) | -0.86 (-0.92 to -0.8) |
| Palau | 12 (10 to 16) | 153 (123.52 to 193.39) |  | 17 (13 to 21) | 107.93 (87.06 to 130.08) | -1.17 (-1.23 to -1.12) |
| Palestine | 342 (265 to 448) | 44.35 (34.47 to 58.6) |  | 488 (405 to 569) | 26.93 (22.32 to 31.45) | -1.66 (-1.85 to -1.47) |
| Panama | 349 (312 to 392) | 24.17 (21.33 to 27.41) |  | 877 (661 to 1098) | 20.31 (15.31 to 25.48) | -0.55 (-0.84 to -0.25) |
| Papua New Guinea | 3338 (2739 to 4001) | 240.12 (190.92 to 286.42) |  | 7154 (5539 to 9108) | 209.49 (162.01 to 259.45) | -0.49 (-0.54 to -0.44) |
| Paraguay | 431 (375 to 552) | 20.67 (17.7 to 27.05) |  | 1148 (878 to 1464) | 22.12 (16.9 to 28.21) | 0.33 (-0.06 to 0.72) |
| Peru | 3320 (2825 to 4232) | 27 (22.75 to 36.91) |  | 6961 (5132 to 8990) | 21.31 (15.64 to 27.63) | -0.65 (-1.06 to -0.23) |
| Philippines | 20035 (17366 to 22256) | 82.95 (72.7 to 93.82) |  | 33229 (27252 to 41083) | 50.85 (41.71 to 64.72) | -1.49 (-1.75 to -1.23) |
| Poland | 14579 (13622 to 15101) | 34.65 (32.14 to 35.99) |  | 11349 (9488 to 13769) | 15.37 (12.87 to 18.69) | -2.99 (-3.22 to -2.75) |
| Portugal | 4624 (4279 to 5029) | 36.33 (33.31 to 39.27) |  | 7052 (5903 to 8062) | 23.69 (20.21 to 26.93) | -1.4 (-1.66 to -1.14) |
| Puerto Rico | 964 (863 to 1053) | 28.05 (24.87 to 30.7) |  | 1832 (1391 to 2281) | 21.92 (16.99 to 27.59) | -0.78 (-1.23 to -0.32) |
| Qatar | 21 (17 to 30) | 32.84 (25.42 to 47.51) |  | 66 (49 to 99) | 22.97 (17.71 to 32.41) | -1.24 (-1.53 to -0.95) |
| Republic of Korea | 11477 (8398 to 12518) | 62.49 (41.58 to 69.62) |  | 15506 (12626 to 17472) | 19.16 (15.35 to 21.58) | -4.07 (-4.35 to -3.78) |
| Republic of Moldova | 1830 (1501 to 1958) | 46.98 (37.85 to 50.47) |  | 971 (823 to 1200) | 16.75 (14.2 to 20.71) | -3.79 (-4.56 to -3.01) |
| Romania | 14278 (11364 to 15298) | 59.46 (46.15 to 64.04) |  | 8106 (6607 to 10101) | 20.77 (16.82 to 25.97) | -3.39 (-3.86 to -2.92) |
| Russian Federation | 57982 (47061 to 62626) | 34.55 (27.49 to 37.36) |  | 39390 (33695 to 46788) | 16.7 (14.31 to 19.87) | -2.33 (-3.17 to -1.48) |
| Rwanda | 2761 (2064 to 3390) | 97.56 (74.5 to 117.55) |  | 2520 (1928 to 3363) | 50.27 (39.13 to 66.02) | -2.35 (-2.49 to -2.21) |
| Saint Kitts and Nevis | 9 (8 to 10) | 24.76 (21.88 to 28.21) |  | 11 (9 to 13) | 20.2 (17.06 to 24.03) | -0.82 (-1.71 to 0.07) |
| Saint Lucia | 30 (27 to 33) | 35.91 (32.55 to 39.66) |  | 59 (48 to 70) | 28.94 (23.69 to 34.31) | -0.79 (-1.08 to -0.49) |
| Saint Vincent and the Grenadines | 11 (9 to 12) | 15.2 (13.41 to 17.44) |  | 20 (17 to 23) | 16.12 (13.65 to 18.48) | 0.27 (0.03 to 0.52) |
| Samoa | 104 (83 to 136) | 138.21 (110.64 to 180.52) |  | 105 (85 to 132) | 82.52 (67.1 to 103.26) | -1.74 (-1.8 to -1.67) |
| San Marino | 5 (4 to 6) | 15.82 (13.19 to 19.14) |  | 11 (7 to 14) | 13.39 (9.26 to 18.35) | -0.54 (-0.65 to -0.44) |
| Sao Tome and Principe | 60 (49 to 69) | 102.65 (84.86 to 118.29) |  | 79 (59 to 98) | 88.33 (66.73 to 108.64) | -0.48 (-0.68 to -0.29) |
| Saudi Arabia | 2561 (1938 to 3534) | 55.43 (41.43 to 75.66) |  | 3723 (2953 to 4532) | 31.74 (25.73 to 37.64) | -1.84 (-2.08 to -1.61) |
| Senegal | 1950 (1499 to 2346) | 65.76 (50.94 to 79.08) |  | 2763 (2158 to 3433) | 41.77 (33.46 to 50.91) | -1.58 (-1.97 to -1.19) |
| Serbia | 3395 (3019 to 3965) | 34.01 (30.13 to 40.16) |  | 3865 (3107 to 4773) | 25.15 (20.29 to 31.12) | -0.91 (-1.15 to -0.67) |
| Seychelles | 26 (23 to 29) | 46.9 (40.37 to 51.38) |  | 31 (26 to 35) | 32.67 (27.98 to 37.18) | -1.33 (-1.74 to -0.92) |
| Sierra Leone | 1186 (911 to 1461) | 65.07 (50.5 to 79.24) |  | 1479 (1108 to 1908) | 44.74 (34.38 to 56.56) | -1.25 (-1.36 to -1.13) |
| Singapore | 958 (824 to 1022) | 54.18 (46.25 to 58.02) |  | 750 (627 to 996) | 10.58 (8.82 to 14.07) | -5.85 (-6.17 to -5.53) |
| Slovakia | 1052 (946 to 1532) | 18.39 (16.52 to 26.51) |  | 1147 (901 to 1484) | 12.56 (9.9 to 16.2) | -1.31 (-1.57 to -1.05) |
| Slovenia | 673 (527 to 837) | 28.4 (22.3 to 35.13) |  | 639 (498 to 801) | 12.71 (9.9 to 16.07) | -2.98 (-3.34 to -2.61) |
| Solomon Islands | 187 (143 to 240) | 168.9 (131.64 to 217.12) |  | 347 (275 to 418) | 145.87 (118.53 to 169.97) | -0.5 (-0.72 to -0.28) |
| Somalia | 2126 (1435 to 3042) | 89.31 (62.19 to 133.51) |  | 3992 (2675 to 6428) | 66.97 (45.16 to 109.05) | -0.98 (-1.06 to -0.91) |
| South Africa | 12236 (10582 to 14044) | 62.79 (53.59 to 72.98) |  | 17827 (16467 to 20125) | 46.6 (42.43 to 51.97) | -0.81 (-1.19 to -0.42) |
| South Sudan | 1396 (917 to 1780) | 58.97 (40.41 to 75.45) |  | 1331 (952 to 1814) | 40.87 (29.4 to 53.86) | -1.24 (-1.29 to -1.19) |
| Spain | 22694 (20391 to 24152) | 42.29 (37.58 to 45.18) |  | 36560 (29180 to 42491) | 29.05 (24.12 to 33.19) | -1.22 (-1.42 to -1.01) |
| Sri Lanka | 9542 (8298 to 10391) | 113.98 (99.12 to 124.21) |  | 13597 (9536 to 17634) | 65.33 (46 to 83.62) | -1.53 (-2 to -1.07) |
| Sudan | 6244 (4005 to 8346) | 72.74 (45.49 to 100.4) |  | 7191 (4823 to 9718) | 45.18 (30.73 to 60.99) | -1.63 (-1.7 to -1.56) |
| Suriname | 63 (56 to 73) | 25.4 (22.74 to 29.09) |  | 117 (97 to 139) | 20.97 (17.35 to 24.94) | -1.21 (-2.82 to 0.43) |
| Sweden | 2481 (2271 to 2978) | 15.11 (13.86 to 18.05) |  | 4478 (3398 to 5046) | 17.85 (13.56 to 20.03) | 0.65 (0.58 to 0.72) |
| Switzerland | 2537 (2300 to 2744) | 22.84 (20.73 to 24.69) |  | 3074 (2563 to 3504) | 14.94 (12.58 to 16.87) | -1.45 (-1.56 to -1.35) |
| Syrian Arab Republic | 2224 (1804 to 2795) | 45.74 (36.85 to 59.76) |  | 3249 (2417 to 4430) | 35.02 (26.6 to 46.91) | -0.97 (-1.24 to -0.69) |
| Taiwan (Province of China) | 5150 (4562 to 5518) | 45.04 (39.14 to 48.76) |  | 9969 (7858 to 12656) | 24.42 (19.16 to 31.06) | -2.03 (-2.24 to -1.81) |
| Tajikistan | 1682 (1436 to 1852) | 61.52 (51.88 to 67.64) |  | 1669 (1337 to 2395) | 48.85 (38.61 to 72.69) | -0.55 (-0.81 to -0.28) |
| Thailand | 20374 (16503 to 23501) | 70.22 (56.47 to 81.16) |  | 23925 (17913 to 31300) | 24.83 (18.64 to 32.4) | -3.65 (-4.07 to -3.22) |
| Timor-Leste | 244 (184 to 311) | 99.34 (74.6 to 127.46) |  | 516 (391 to 651) | 77.66 (59.67 to 98.03) | -0.86 (-0.98 to -0.74) |
| Togo | 741 (591 to 881) | 65.75 (51.99 to 78.37) |  | 1332 (985 to 1729) | 43.17 (33.1 to 55.37) | -1.47 (-1.54 to -1.39) |
| Tokelau | 2 (1 to 2) | 124.05 (101.53 to 153.4) |  | 1 (1 to 1) | 63.5 (49.21 to 79.32) | -2.32 (-2.36 to -2.28) |
| Tonga | 36 (31 to 42) | 79.5 (66.4 to 92.07) |  | 42 (34 to 50) | 54.91 (44.13 to 65.8) | -1.12 (-1.7 to -0.54) |
| Trinidad and Tobago | 201 (188 to 218) | 25.67 (23.78 to 27.97) |  | 271 (201 to 360) | 15.58 (11.61 to 20.59) | -1.66 (-2.02 to -1.29) |
| Tunisia | 1381 (1119 to 1833) | 34.31 (27.61 to 45.45) |  | 2649 (1963 to 3507) | 24.02 (17.97 to 31.61) | -1.23 (-1.3 to -1.15) |
| Turkey | 18824 (15855 to 21343) | 60.16 (50.41 to 68.57) |  | 32981 (22668 to 40823) | 40.64 (28.01 to 50.33) | -1.38 (-1.63 to -1.12) |
| Turkmenistan | 843 (653 to 912) | 50.03 (38.3 to 54.39) |  | 442 (323 to 806) | 13.26 (9.64 to 25.01) | -5.16 (-5.82 to -4.5) |
| Tuvalu | 10 (8 to 12) | 167.79 (132.25 to 215.64) |  | 7 (6 to 10) | 87.49 (67.74 to 110.57) | -2.23 (-2.29 to -2.16) |
| Uganda | 4164 (2791 to 5910) | 68.16 (45.78 to 96.71) |  | 5480 (4037 to 7142) | 44.02 (33 to 56.25) | -1.54 (-1.62 to -1.47) |
| Ukraine | 33452 (26048 to 36237) | 49.76 (38.14 to 54.11) |  | 12181 (9742 to 18471) | 15.96 (12.78 to 24.16) | -3.91 (-4.63 to -3.19) |
| United Arab Emirates | 310 (232 to 417) | 77.58 (61.14 to 104.08) |  | 1778 (1216 to 2383) | 45.5 (35.6 to 58.31) | -2.03 (-2.27 to -1.8) |
| United Kingdom | 37657 (35478 to 43241) | 39.59 (37.28 to 45.62) |  | 51879 (43733 to 55229) | 36.54 (30.82 to 38.77) | -0.2 (-0.35 to -0.06) |
| United Republic of Tanzania | 4190 (3520 to 4867) | 41.44 (35.07 to 48.02) |  | 7001 (5759 to 8305) | 31.42 (25.67 to 36.63) | -0.94 (-1.03 to -0.85) |
| United States of America | 106098 (98990 to 124041) | 31.61 (29.57 to 36.81) |  | 224988 (181616 to 240468) | 37.72 (30.84 to 40.16) | 0.6 (0.54 to 0.67) |
| United States Virgin Islands | 14 (12 to 17) | 19.44 (16.36 to 23.27) |  | 31 (25 to 35) | 17.62 (14.45 to 20.34) | -0.23 (-0.48 to 0.03) |
| Uruguay | 1291 (1174 to 1538) | 33.38 (30.34 to 40.06) |  | 2155 (1888 to 2389) | 35.61 (31.35 to 39.19) | 0.32 (0.11 to 0.53) |
| Uzbekistan | 4929 (3868 to 5379) | 46.84 (36.58 to 51.27) |  | 3837 (3035 to 5646) | 28.98 (23.23 to 47.54) | -2.08 (-2.64 to -1.51) |
| Vanuatu | 90 (63 to 123) | 166.47 (119 to 227.78) |  | 172 (129 to 228) | 121.24 (92.27 to 156.46) | -1.13 (-1.28 to -0.99) |
| Venezuela (Bolivarian Republic of) | 2046 (1856 to 2303) | 23.29 (20.73 to 26.98) |  | 7062 (5421 to 9045) | 26.08 (20.01 to 33.27) | 0.33 (-0.17 to 0.84) |
| Viet Nam | 26078 (9883 to 33160) | 73.93 (27.59 to 93.74) |  | 36229 (14127 to 45192) | 47.62 (18.73 to 58.89) | -1.47 (-1.61 to -1.34) |
| Yemen | 3341 (2294 to 4736) | 79.73 (53.62 to 116.73) |  | 5738 (4433 to 7400) | 52.51 (41.42 to 66.84) | -1.44 (-1.51 to -1.38) |
| Zambia | 1570 (1283 to 1886) | 57.54 (47.35 to 70.37) |  | 2388 (1919 to 2919) | 41.65 (33.77 to 50.26) | -1.18 (-1.28 to -1.08) |
| Zimbabwe | 1820 (1560 to 2073) | 53.07 (45.66 to 60.13) |  | 2891 (2000 to 3774) | 49.07 (34.59 to 63.36) | -0.28 (-0.4 to -0.15) |

CRDs: chronic respiratory diseases; AAPC: average annual percent change; UI: uncertainty interval; CI: confidence interval; SDI: Socio-demographic index.

Table S9. Mortality of COPD (1990/2019) for sexes, SDI and locations, with AAPC (1990-2019)

| GBD data | 1990 | |  | 2019 | | AAPC % (95% CI)  1990-2019 |
| --- | --- | --- | --- | --- | --- | --- |
|  | Cases (95% UI) | Age-standardised mortality per  100 000 population (95% UI) |  | Cases (95% UI) | Age-standardised mortality per  100 000 population (95% UI) |  |
| **Global** | 3093755 (2583800 to 3339171) | 87.89 (73.87 to 95.1) |  | 3280636 (2902855 to 3572367) | 51.28 (45.9 to 55.51) | -1.92 (-2 to -1.84) |
| **Sex** |  |  |  |  |  |  |
| Female | 1360492 (1023327 to 1507670) | 67.84 (51.59 to 75.41) |  | 1740667 (1456918 to 1961313) | 39.73 (33.24 to 44.75) | -1.92 (-2 to -1.84) |
| Male | 1733263 (1508440 to 1884436) | 116.75 (102.61 to 126.76) |  | 2233647 (2029782 to 2452532) | 66.72 (60.55 to 73.06) | -1.97 (-2.06 to -1.89) |
| **SDI** |  |  |  |  |  |  |
| High SDI | 322191 (301748 to 352570) | 30.3 (28.3 to 33.21) |  | 525019 (453715 to 559024) | 24.64 (21.49 to 26.07) | -0.73 (-0.8 to -0.66) |
| High-middle SDI | 744067 (598458 to 810269) | 80.81 (64.52 to 87.92) |  | 651323 (580032 to 772576) | 33.22 (29.45 to 39.31) | -3.2 (-3.42 to -2.97) |
| Middle SDI | 1042205 (817157 to 1147392) | 134.26 (106.75 to 147) |  | 1206232 (1062900 to 1340546) | 59.82 (52.3 to 66.61) | -2.83 (-2.98 to -2.67) |
| Low-middle SDI | 761018 (634082 to 845278) | 160.43 (135.86 to 179.49) |  | 1226245 (1031957 to 1380038) | 107.29 (90.1 to 120.82) | -1.36 (-1.57 to -1.15) |
| Low SDI | 223276 (187251 to 253304) | 114.69 (96.34 to 133.08) |  | 363857 (310934 to 403593) | 87.82 (74.17 to 97.67) | -0.95 (-1.16 to -0.73) |
| **Region** |  |  |  |  |  |  |
| High-income Asia Pacific | 42370 (37231 to 44366) | 24 (20.85 to 25.25) |  | 72143 (59578 to 81146) | 12.31 (10.4 to 13.74) | -2.37 (-2.52 to -2.22) |
| High-income North America | 115443 (107761 to 133643) | 31.29 (29.23 to 36.15) |  | 243409 (196086 to 260316) | 36.24 (29.59 to 38.58) | 0.47 (0.41 to 0.53) |
| Western Europe | 185311 (173116 to 200615) | 31 (28.87 to 33.56) |  | 244861 (210219 to 263528) | 22.45 (19.62 to 24.03) | -1.11 (-1.21 to -1) |
| Australasia | 8508 (7953 to 9006) | 36.52 (33.93 to 38.72) |  | 13599 (11348 to 15144) | 24.82 (20.92 to 27.5) | -1.33 (-1.44 to -1.21) |
| Andean Latin America | 6889 (6131 to 7876) | 33.72 (30.1 to 40.56) |  | 14209 (11012 to 17013) | 26.84 (20.79 to 32.1) | -0.74 (-0.97 to -0.51) |
| Tropical Latin America | 40684 (37275 to 42704) | 54.94 (49.56 to 58.05) |  | 77698 (69788 to 86968) | 34.1 (30.5 to 38.25) | -1.65 (-1.81 to -1.49) |
| Central Latin America | 30365 (27299 to 31656) | 42.34 (37.85 to 44.43) |  | 74582 (62845 to 85574) | 33.69 (28.27 to 38.66) | -0.8 (-0.97 to -0.64) |
| Southern Latin America | 13966 (13014 to 15616) | 32.72 (30.22 to 37.11) |  | 27893 (24150 to 30844) | 32.56 (28.24 to 35.95) | -0.04 (-0.36 to 0.28) |
| Caribbean | 7309 (6432 to 8172) | 28.3 (24.92 to 31.68) |  | 13766 (11412 to 16209) | 26.87 (22.23 to 31.67) | -0.05 (-0.21 to 0.11) |
| Central Europe | 50505 (46626 to 52058) | 36.96 (33.94 to 38.21) |  | 42773 (37191 to 49286) | 19.12 (16.63 to 22.05) | -2.22 (-2.37 to -2.06) |
| Eastern Europe | 102468 (81695 to 109757) | 39.44 (30.85 to 42.36) |  | 56496 (49473 to 71897) | 16.23 (14.22 to 20.72) | -2.9 (-3.65 to -2.14) |
| Central Asia | 21753 (18617 to 22909) | 51.12 (43.53 to 54.07) |  | 22237 (20056 to 26149) | 39.41 (35.47 to 46.52) | -0.62 (-1.03 to -0.21) |
| North Africa and Middle East | 78174 (68863 to 87436) | 53.8 (47.11 to 61.58) |  | 128513 (110781 to 144351) | 36.1 (30.9 to 40.31) | -1.38 (-1.51 to -1.25) |
| South Asia | 748038 (644493 to 838307) | 179.62 (154.45 to 204.39) |  | 1363402 (1125162 to 1563756) | 118.75 (97.56 to 135.84) | -1.49 (-1.72 to -1.26) |
| Southeast Asia | 190752 (151873 to 213268) | 90.37 (70.75 to 102.45) |  | 267847 (233250 to 296152) | 53.72 (46.49 to 59.45) | -1.83 (-1.92 to -1.74) |
| East Asia | 1325881 (975800 to 1485484) | 221.48 (166.31 to 246.45) |  | 1126946 (969591 to 1362127) | 67.48 (57.8 to 82.27) | -4.15 (-4.49 to -3.81) |
| Oceania | 4656 (3880 to 5451) | 201.85 (164.72 to 235.52) |  | 8940 (7142 to 11103) | 166.28 (133.34 to 202.63) | -0.68 (-0.71 to -0.66) |
| Western Sub-Saharan Africa | 43327 (35835 to 50442) | 54.64 (45.14 to 63.75) |  | 64223 (54089 to 74359) | 39.14 (33.48 to 44.62) | -1.15 (-1.21 to -1.09) |
| Eastern Sub-Saharan Africa | 44814 (37876 to 51042) | 63.2 (53.59 to 75.14) |  | 60212 (51794 to 69611) | 42.4 (36.86 to 48.27) | -1.39 (-1.43 to -1.35) |
| Central Sub-Saharan Africa | 16116 (11564 to 21424) | 86.5 (60.43 to 123.44) |  | 26972 (18531 to 40753) | 65.8 (44.51 to 104.69) | -0.99 (-1.09 to -0.89) |
| Southern Sub-Saharan Africa | 16429 (14124 to 19095) | 65.65 (55.94 to 77.28) |  | 23593 (21463 to 26023) | 49.21 (44.37 to 54.24) | -0.79 (-1.11 to -0.47) |
| **Countries** |  |  |  |  |  |  |
| Afghanistan | 5945 (4809 to 7128) | 94.84 (78.02 to 112.96) |  | 7082 (5444 to 8699) | 67.82 (51.99 to 81.33) | -1.11 (-1.21 to -1) |
| Albania | 1006 (695 to 1096) | 59.1 (39.61 to 64.7) |  | 815 (608 to 1053) | 19.54 (14.61 to 25.16) | -3.72 (-4.42 to -3.03) |
| Algeria | 4410 (3378 to 5596) | 53.82 (41.75 to 66.94) |  | 7528 (6030 to 9347) | 30.36 (24.5 to 37.49) | -1.96 (-2.03 to -1.89) |
| American Samoa | 17 (15 to 19) | 96.52 (82.7 to 109.26) |  | 23 (19 to 28) | 57.84 (48.68 to 69.95) | -1.8 (-1.91 to -1.69) |
| Andorra | 16 (12 to 21) | 35.77 (27.88 to 46.21) |  | 39 (31 to 49) | 25.66 (20.11 to 32.13) | -1.17 (-1.27 to -1.08) |
| Angola | 2794 (2084 to 3626) | 82.55 (59.96 to 118.87) |  | 3934 (2886 to 5046) | 46.92 (35.19 to 62.07) | -1.99 (-2.13 to -1.85) |
| Antigua and Barbuda | 7 (6 to 8) | 11.61 (10.33 to 13.08) |  | 11 (9 to 13) | 11.93 (9.71 to 14.2) | 0.25 (-0.52 to 1.02) |
| Argentina | 9894 (9154 to 11385) | 33.02 (30.24 to 38.6) |  | 19348 (16775 to 21811) | 34.85 (30.28 to 39.29) | 0.34 (0.08 to 0.59) |
| Armenia | 1078 (902 to 1154) | 48.39 (39.39 to 52.16) |  | 1323 (1016 to 1581) | 33.77 (25.81 to 40.44) | -1.18 (-1.62 to -0.74) |
| Australia | 6966 (6488 to 7402) | 35.97 (33.28 to 38.3) |  | 11203 (9284 to 12661) | 24.18 (20.3 to 27.12) | -1.32 (-1.43 to -1.21) |
| Austria | 2524 (2339 to 2898) | 20.33 (18.81 to 23.3) |  | 3406 (2914 to 3825) | 16.92 (14.47 to 18.79) | -0.71 (-1.04 to -0.39) |
| Azerbaijan | 1934 (1728 to 2169) | 41.91 (37.35 to 47.59) |  | 2006 (1556 to 3036) | 31.94 (23.85 to 49.48) | -0.65 (-0.93 to -0.37) |
| Bahamas | 24 (21 to 27) | 16.36 (14.42 to 18.53) |  | 51 (41 to 63) | 14.58 (11.67 to 18.06) | -0.36 (-0.55 to -0.17) |
| Bahrain | 86 (74 to 98) | 78.35 (67.14 to 88.92) |  | 161 (129 to 207) | 37.15 (30.47 to 45.41) | -2.98 (-3.63 to -2.32) |
| Bangladesh | 61137 (52166 to 78436) | 147.55 (125.64 to 198.33) |  | 70893 (53727 to 117828) | 62.02 (47.21 to 106.8) | -2.74 (-3.38 to -2.1) |
| Barbados | 40 (35 to 45) | 12.9 (11.42 to 14.62) |  | 63 (50 to 76) | 12.96 (10.4 to 15.57) | -0.08 (-0.46 to 0.31) |
| Belarus | 6648 (4906 to 7316) | 53.71 (39.04 to 59.38) |  | 2438 (1838 to 3910) | 14.98 (11.29 to 24.05) | -4.49 (-5.25 to -3.72) |
| Belgium | 6494 (6017 to 7017) | 41.08 (38.01 to 44.29) |  | 7400 (6282 to 8256) | 27.63 (23.71 to 30.62) | -1.4 (-1.58 to -1.22) |
| Belize | 22 (19 to 25) | 22.42 (19.47 to 25.37) |  | 74 (62 to 87) | 28.82 (24.06 to 33.98) | 1.3 (0.69 to 1.92) |
| Benin | 1340 (1082 to 1589) | 69.2 (55.9 to 82.02) |  | 1888 (1458 to 2449) | 42.45 (34.03 to 53.78) | -1.69 (-1.79 to -1.59) |
| Bermuda | 11 (10 to 12) | 19.04 (16.91 to 21.48) |  | 18 (15 to 22) | 13.21 (10.89 to 15.97) | -1.15 (-1.31 to -0.99) |
| Bhutan | 300 (218 to 394) | 162.86 (120.78 to 216.77) |  | 571 (434 to 781) | 123.17 (94.18 to 164.64) | -0.96 (-1.03 to -0.9) |
| Bolivia (Plurinational State of) | 1856 (1475 to 2221) | 62.07 (51.29 to 73.06) |  | 3578 (2812 to 4445) | 51.07 (40.15 to 63.07) | -0.65 (-0.71 to -0.59) |
| Bosnia and Herzegovina | 1162 (997 to 1244) | 36.31 (30.79 to 39.07) |  | 1204 (953 to 1487) | 20.97 (16.59 to 25.73) | -2.11 (-2.29 to -1.92) |
| Botswana | 463 (335 to 650) | 95.12 (68.85 to 132.98) |  | 649 (481 to 847) | 58.45 (43.86 to 74) | -1.67 (-1.79 to -1.55) |
| Brazil | 40254 (36784 to 42288) | 56.04 (50.36 to 59.18) |  | 76551 (68782 to 85616) | 34.38 (30.76 to 38.59) | -1.7 (-1.85 to -1.54) |
| Brunei Darussalam | 73 (53 to 82) | 128.47 (85.53 to 147.9) |  | 93 (81 to 107) | 64.5 (54.57 to 72.9) | -2.27 (-2.59 to -1.96) |
| Bulgaria | 3786 (3436 to 4056) | 35.16 (31.47 to 37.74) |  | 3036 (2405 to 3809) | 20.51 (16.24 to 25.64) | -1.75 (-2.05 to -1.45) |
| Burkina Faso | 1658 (1350 to 1989) | 41.99 (34.73 to 50.35) |  | 2714 (2232 to 3291) | 31.75 (26.44 to 38.01) | -0.9 (-1.15 to -0.65) |
| Burundi | 2249 (1633 to 2806) | 94.5 (69.75 to 116.48) |  | 2399 (1769 to 3214) | 61.07 (45.92 to 79.19) | -1.54 (-1.67 to -1.41) |
| Cabo Verde | 133 (86 to 152) | 55.5 (36.06 to 63.02) |  | 107 (86 to 145) | 25.59 (20.74 to 34.64) | -2.82 (-3.31 to -2.31) |
| Cambodia | 2738 (2257 to 3164) | 73.54 (59.91 to 85.76) |  | 4959 (3936 to 5791) | 53.76 (42.74 to 62.11) | -1.07 (-1.11 to -1.03) |
| Cameroon | 2630 (2116 to 3139) | 67.97 (55.12 to 80.56) |  | 4166 (3053 to 5526) | 40.02 (30.06 to 51.76) | -1.84 (-1.91 to -1.76) |
| Canada | 9319 (8590 to 10076) | 28.77 (26.44 to 31.03) |  | 18385 (14506 to 20999) | 24.22 (19.52 to 27.56) | -0.62 (-0.72 to -0.53) |
| Central African Republic | 1074 (766 to 1444) | 109.22 (73.88 to 161.94) |  | 1579 (1054 to 2342) | 91.58 (58.78 to 146.99) | -0.59 (-0.66 to -0.52) |
| Chad | 1886 (1454 to 2493) | 69.78 (53.61 to 92.4) |  | 2761 (2143 to 3486) | 52.82 (40.86 to 65.42) | -0.95 (-1.03 to -0.87) |
| Chile | 2781 (2444 to 2956) | 32.28 (28.09 to 34.53) |  | 6389 (4943 to 7227) | 26.87 (20.81 to 30.37) | -0.63 (-0.82 to -0.45) |
| China | 1301224 (955216 to 1458961) | 226.43 (170.16 to 251.43) |  | 1085273 (929797 to 1320157) | 67.98 (57.8 to 83.43) | -4.2 (-4.56 to -3.84) |
| Colombia | 6211 (5414 to 6638) | 41.82 (36.49 to 44.92) |  | 17772 (12952 to 22724) | 31.9 (23.4 to 40.81) | -1.23 (-1.41 to -1.05) |
| Comoros | 129 (80 to 168) | 62.14 (41.96 to 79.47) |  | 167 (131 to 208) | 38.28 (30.52 to 47.36) | -1.6 (-1.79 to -1.41) |
| Congo | 789 (568 to 1089) | 90.3 (63.54 to 132.36) |  | 997 (723 to 1305) | 51.39 (37.85 to 68.2) | -1.88 (-2.03 to -1.74) |
| Cook Islands | 8 (6 to 9) | 71.88 (60.06 to 82.58) |  | 7 (6 to 9) | 32.74 (27.05 to 39.98) | -2.64 (-2.84 to -2.44) |
| Costa Rica | 477 (420 to 526) | 29.57 (25.9 to 32.79) |  | 1425 (1086 to 1794) | 27.59 (21.1 to 34.87) | -0.15 (-0.7 to 0.41) |
| Croatia | 1282 (1173 to 1410) | 21.97 (19.97 to 24.1) |  | 1878 (1467 to 2322) | 19.53 (15.31 to 24.14) | -0.3 (-0.73 to 0.14) |
| Cuba | 1866 (1712 to 1989) | 18.66 (17.09 to 19.98) |  | 4586 (3549 to 5603) | 23.29 (17.9 to 28.42) | 0.99 (0.71 to 1.27) |
| Cyprus | 327 (270 to 374) | 57.95 (46.17 to 66.57) |  | 541 (400 to 626) | 32.53 (22.62 to 37.96) | -1.92 (-2.24 to -1.6) |
| Czechia | 3193 (2968 to 3760) | 23.64 (21.87 to 28) |  | 4062 (3295 to 4918) | 18.4 (14.9 to 22.28) | -0.74 (-1.3 to -0.18) |
| Côte d’Ivoire | 2305 (1790 to 2764) | 67.46 (52.91 to 80.4) |  | 3642 (2763 to 4612) | 40.43 (32.05 to 49.5) | -1.8 (-1.95 to -1.66) |
| Democratic People's Republic of Korea | 19507 (14226 to 25359) | 174.31 (126.7 to 231.33) |  | 31704 (25210 to 37877) | 112.77 (90.31 to 136.52) | -1.52 (-1.59 to -1.46) |
| Democratic Republic of the Congo | 10993 (7447 to 15008) | 86.78 (58.21 to 126.81) |  | 19997 (12900 to 32274) | 71.96 (45.13 to 121.72) | -0.67 (-0.77 to -0.57) |
| Denmark | 3265 (3026 to 3486) | 38.24 (35.28 to 40.64) |  | 4572 (3610 to 5186) | 36 (28.4 to 40.8) | -0.2 (-0.41 to 0) |
| Djibouti | 60 (40 to 84) | 48.45 (32.72 to 63.64) |  | 146 (97 to 209) | 31.66 (21.13 to 44.06) | -1.52 (-1.61 to -1.42) |
| Dominica | 17 (15 to 21) | 23.28 (20.69 to 27.77) |  | 22 (17 to 27) | 24.28 (19.03 to 29.67) | 0.17 (0.08 to 0.25) |
| Dominican Republic | 915 (774 to 1029) | 24.32 (20.63 to 27.38) |  | 1937 (1436 to 2516) | 22.19 (16.46 to 28.7) | 0.01 (-0.3 to 0.31) |
| Ecuador | 1713 (1424 to 1860) | 34.95 (30.21 to 37.58) |  | 3670 (2838 to 4523) | 29.81 (22.53 to 36.57) | -0.45 (-0.78 to -0.12) |
| Egypt | 15225 (13669 to 16935) | 58.83 (53.37 to 68.33) |  | 22560 (16449 to 29261) | 43.58 (32.27 to 55.42) | -0.96 (-1.39 to -0.53) |
| El Salvador | 1042 (885 to 1134) | 33.9 (29.33 to 37.23) |  | 1514 (1116 to 1881) | 23.1 (17.08 to 28.72) | -1.31 (-1.72 to -0.9) |
| Equatorial Guinea | 160 (109 to 223) | 93.43 (61.07 to 142.33) |  | 157 (103 to 262) | 42.84 (28.57 to 74.23) | -2.72 (-2.89 to -2.56) |
| Eritrea | 704 (475 to 908) | 71.55 (49.99 to 94.29) |  | 1117 (784 to 1437) | 49.95 (34.38 to 62.92) | -1.19 (-1.32 to -1.07) |
| Estonia | 332 (308 to 369) | 16.6 (15.35 to 18.52) |  | 303 (238 to 388) | 10.27 (8.01 to 13.09) | -1.6 (-2.35 to -0.85) |
| Eswatini | 251 (185 to 345) | 97.63 (71.75 to 135.29) |  | 319 (238 to 417) | 65.53 (49.9 to 83.65) | -1.42 (-1.59 to -1.25) |
| Ethiopia | 13499 (10819 to 15548) | 71.35 (58.57 to 84.83) |  | 13727 (11713 to 15813) | 38.43 (32.63 to 44.17) | -2.18 (-2.28 to -2.09) |
| Fiji | 400 (320 to 495) | 143.05 (115.79 to 177.69) |  | 412 (321 to 534) | 73.44 (58.69 to 92.71) | -2.37 (-2.65 to -2.09) |
| Finland | 1253 (1163 to 1405) | 17.27 (15.97 to 19.48) |  | 2043 (1736 to 2286) | 14.6 (12.5 to 16.3) | -0.6 (-0.84 to -0.36) |
| France | 22050 (19530 to 24041) | 24.91 (21.98 to 27.12) |  | 22060 (18180 to 26355) | 12.76 (10.79 to 15.11) | -2.32 (-2.52 to -2.12) |
| Gabon | 306 (220 to 386) | 63.62 (44.58 to 82.04) |  | 308 (218 to 400) | 37.08 (26.2 to 47.01) | -1.83 (-1.94 to -1.72) |
| Gambia | 196 (143 to 255) | 62.56 (46.95 to 79.47) |  | 424 (325 to 536) | 48.78 (37.97 to 61.02) | -0.97 (-1.26 to -0.69) |
| Georgia | 1196 (1056 to 1596) | 22.53 (19.89 to 30.48) |  | 1222 (975 to 1675) | 19.36 (15.5 to 26.07) | -0.52 (-0.95 to -0.1) |
| Germany | 39888 (36879 to 45489) | 30.3 (27.96 to 34.48) |  | 45165 (39408 to 51730) | 20.75 (18.24 to 23.49) | -1.42 (-1.73 to -1.11) |
| Ghana | 3076 (1929 to 3784) | 55.71 (35.32 to 68.48) |  | 5938 (3587 to 7459) | 42.08 (25.74 to 51.96) | -0.89 (-0.98 to -0.81) |
| Greece | 2627 (2385 to 2863) | 18.25 (16.37 to 19.85) |  | 6399 (4869 to 7428) | 20.39 (16.17 to 23.24) | 0.23 (-0.17 to 0.64) |
| Greenland | 24 (19 to 27) | 92.77 (73 to 105.6) |  | 32 (24 to 38) | 56.34 (40.93 to 66.46) | -1.67 (-2.07 to -1.27) |
| Grenada | 18 (16 to 20) | 22.34 (19.59 to 25.35) |  | 19 (16 to 22) | 19.1 (16.42 to 21.73) | -0.74 (-1.28 to -0.19) |
| Guam | 30 (27 to 34) | 60.45 (52.33 to 68.12) |  | 53 (43 to 65) | 28.73 (23.49 to 35.23) | -2.81 (-3.29 to -2.33) |
| Guatemala | 1627 (1382 to 1819) | 45.88 (40.55 to 51.28) |  | 2529 (1988 to 3209) | 27.19 (21.66 to 34.14) | -1.91 (-2.43 to -1.38) |
| Guinea | 2215 (1776 to 2770) | 70.92 (56.81 to 89.72) |  | 2787 (2134 to 3446) | 53.06 (41.08 to 65.19) | -0.97 (-1.02 to -0.92) |
| Guinea-Bissau | 370 (293 to 452) | 96.12 (76.33 to 115.38) |  | 386 (297 to 485) | 59.15 (45.8 to 73.57) | -1.65 (-1.72 to -1.58) |
| Guyana | 90 (77 to 104) | 25.58 (22.02 to 29.55) |  | 119 (92 to 148) | 21.57 (16.89 to 26.57) | -0.48 (-1.14 to 0.2) |
| Haiti | 2420 (1642 to 3185) | 72.44 (44.93 to 105.25) |  | 3420 (2266 to 4792) | 54.34 (35 to 77.46) | -0.97 (-1.01 to -0.92) |
| Honduras | 1274 (1036 to 1464) | 57.71 (43.75 to 68.1) |  | 3536 (2229 to 4628) | 68.62 (41.59 to 88.94) | 0.96 (0.56 to 1.36) |
| Hungary | 5385 (5065 to 6151) | 39.1 (36.58 to 44.73) |  | 5936 (4888 to 7093) | 29.7 (24.36 to 35.39) | -0.91 (-1.52 to -0.29) |
| Iceland | 70 (62 to 80) | 23.04 (20.52 to 26.32) |  | 111 (89 to 127) | 17.5 (14.24 to 20.02) | -0.87 (-1.11 to -0.63) |
| India | 601995 (491310 to 683144) | 195.96 (159.48 to 226) |  | 1168381 (899260 to 1363609) | 125.11 (95.2 to 145.25) | -1.61 (-1.9 to -1.32) |
| Indonesia | 65596 (56281 to 74089) | 83.86 (71.34 to 96.81) |  | 100930 (85012 to 113831) | 61.93 (52.48 to 70.18) | -1.05 (-1.19 to -0.91) |
| Iran (Islamic Republic of) | 8207 (7169 to 9874) | 42.64 (36.06 to 52.57) |  | 16835 (14588 to 18193) | 26.93 (23.24 to 29.14) | -1.52 (-1.61 to -1.43) |
| Iraq | 2101 (1714 to 2584) | 28.16 (22.82 to 35.34) |  | 3292 (2577 to 4153) | 17.32 (13.82 to 21.98) | -1.68 (-2.16 to -1.19) |
| Ireland | 2301 (2043 to 2429) | 56.83 (50.44 to 60.09) |  | 2548 (2135 to 2887) | 32.41 (27.25 to 36.7) | -1.91 (-2.08 to -1.73) |
| Israel | 1328 (1222 to 1429) | 29.35 (26.79 to 31.79) |  | 2027 (1763 to 2482) | 16.18 (14.17 to 19.79) | -2.13 (-2.35 to -1.92) |
| Italy | 24263 (22611 to 25175) | 27.24 (25.05 to 28.33) |  | 31345 (26440 to 34246) | 16.76 (14.51 to 18.17) | -1.67 (-1.85 to -1.5) |
| Jamaica | 345 (310 to 392) | 18.45 (16.6 to 21.14) |  | 642 (494 to 800) | 20.73 (15.93 to 26.08) | 0.5 (-0.17 to 1.18) |
| Japan | 29862 (27076 to 31160) | 19.46 (17.52 to 20.39) |  | 55794 (45378 to 63876) | 11.41 (9.61 to 12.91) | -1.87 (-2.02 to -1.72) |
| Jordan | 397 (325 to 469) | 38.78 (31.73 to 46.17) |  | 856 (700 to 1028) | 17.79 (14.57 to 21.48) | -2.71 (-3.14 to -2.29) |
| Kazakhstan | 6905 (6041 to 7412) | 60.49 (52.52 to 65.11) |  | 9772 (8198 to 11527) | 68.18 (56.63 to 80.18) | 0.87 (0.25 to 1.49) |
| Kenya | 3501 (2573 to 5301) | 47.87 (34.9 to 73.98) |  | 7705 (5822 to 10391) | 42.9 (32.45 to 57.47) | -0.39 (-0.47 to -0.31) |
| Kiribati | 58 (47 to 70) | 184.25 (152.41 to 230) |  | 73 (57 to 90) | 144.1 (112.54 to 176.67) | -0.87 (-0.98 to -0.76) |
| Kuwait | 81 (70 to 91) | 17.21 (14.38 to 19.64) |  | 230 (184 to 277) | 12.22 (9.69 to 14.91) | -0.83 (-2.12 to 0.47) |
| Kyrgyzstan | 2658 (2017 to 2937) | 92.09 (69.32 to 102.1) |  | 1513 (1283 to 1889) | 41.08 (34.89 to 51.25) | -3 (-3.57 to -2.41) |
| Lao People's Democratic Republic | 2563 (1993 to 3123) | 135.08 (106.06 to 165.81) |  | 2663 (2092 to 3360) | 76.7 (61.34 to 96.08) | -1.98 (-2.04 to -1.91) |
| Latvia | 750 (695 to 803) | 21.2 (19.63 to 22.64) |  | 433 (350 to 599) | 9.92 (7.94 to 13.53) | -2.51 (-3.25 to -1.76) |
| Lebanon | 695 (564 to 865) | 37.81 (30.6 to 47.38) |  | 1185 (918 to 1499) | 23.35 (18.13 to 29.69) | -1.66 (-1.7 to -1.61) |
| Lesotho | 1000 (674 to 1438) | 114.66 (77.92 to 164.91) |  | 1116 (787 to 1526) | 104.4 (74.25 to 141.45) | -0.22 (-0.45 to 0.01) |
| Liberia | 437 (361 to 521) | 40.93 (34.27 to 49.57) |  | 534 (398 to 715) | 29.75 (23.04 to 39.56) | -0.96 (-1.36 to -0.56) |
| Libya | 559 (433 to 722) | 33.34 (25.32 to 43.46) |  | 1173 (882 to 1477) | 26.61 (19.92 to 33.35) | -0.75 (-1.32 to -0.17) |
| Lithuania | 1473 (1256 to 1568) | 32.82 (27.97 to 34.93) |  | 779 (631 to 1010) | 12.21 (9.9 to 15.8) | -3.38 (-4.1 to -2.66) |
| Luxembourg | 176 (163 to 195) | 32.59 (29.99 to 36.01) |  | 221 (183 to 260) | 19.91 (16.54 to 23.25) | -1.7 (-1.87 to -1.54) |
| Madagascar | 4165 (3657 to 4705) | 78.35 (68.98 to 90.3) |  | 6030 (4601 to 7698) | 65.01 (49.59 to 82.25) | -0.61 (-0.72 to -0.49) |
| Malawi | 1816 (1460 to 2165) | 50.14 (40.9 to 58.85) |  | 2402 (1967 to 2842) | 37.21 (30.74 to 43.72) | -1.01 (-1.07 to -0.94) |
| Malaysia | 5101 (4196 to 6029) | 65.33 (53.95 to 78.15) |  | 7473 (5862 to 10018) | 34.38 (27 to 46.19) | -2.4 (-3.27 to -1.52) |
| Maldives | 96 (73 to 113) | 143.31 (113.82 to 164.18) |  | 123 (102 to 147) | 51.64 (42.42 to 61.43) | -3.74 (-4.04 to -3.43) |
| Mali | 2894 (2199 to 3420) | 74.45 (57.59 to 87.6) |  | 4574 (3389 to 5877) | 56.29 (43.08 to 70.7) | -0.91 (-1 to -0.83) |
| Malta | 106 (96 to 117) | 26.29 (23.72 to 28.86) |  | 157 (132 to 181) | 15.4 (12.97 to 17.65) | -1.98 (-2.12 to -1.84) |
| Marshall Islands | 22 (18 to 30) | 155.34 (124.04 to 214.45) |  | 26 (19 to 35) | 104.71 (77.56 to 136.15) | -1.45 (-1.64 to -1.26) |
| Mauritania | 526 (380 to 644) | 57.35 (41.27 to 70.01) |  | 525 (402 to 670) | 28.76 (22.5 to 36.13) | -2.45 (-2.57 to -2.32) |
| Mauritius | 450 (398 to 482) | 73.83 (64.51 to 79.48) |  | 537 (429 to 678) | 34.9 (28.14 to 44.16) | -2.45 (-2.79 to -2.11) |
| Mexico | 16910 (15104 to 17646) | 49.17 (43.38 to 51.63) |  | 38524 (32632 to 43748) | 36.93 (31.2 to 41.88) | -0.98 (-1.2 to -0.77) |
| Micronesia (Federated States of) | 68 (53 to 84) | 171.54 (133.45 to 219.17) |  | 57 (42 to 73) | 108.37 (84.05 to 136.73) | -1.62 (-1.67 to -1.56) |
| Monaco | 15 (12 to 19) | 18.69 (14.83 to 23.27) |  | 20 (16 to 23) | 17.04 (13.67 to 20.12) | -0.24 (-0.41 to -0.06) |
| Mongolia | 526 (412 to 622) | 56.55 (44.27 to 66.64) |  | 453 (357 to 608) | 27.81 (22.73 to 34.98) | -2.53 (-2.81 to -2.25) |
| Montenegro | 54 (44 to 63) | 9.76 (7.91 to 11.52) |  | 85 (69 to 100) | 9.32 (7.48 to 10.91) | 0.08 (-0.05 to 0.21) |
| Morocco | 4982 (3971 to 6517) | 43.72 (34.6 to 59.43) |  | 9373 (7233 to 11682) | 37.27 (29.08 to 46.8) | -0.66 (-1.25 to -0.06) |
| Mozambique | 2451 (1960 to 2992) | 44.61 (36.64 to 54.55) |  | 3759 (2983 to 4746) | 38.91 (31.11 to 49.16) | -0.46 (-0.6 to -0.33) |
| Myanmar | 37654 (27630 to 46073) | 193.65 (139.34 to 233.07) |  | 43284 (33261 to 50185) | 115.56 (88.11 to 133.27) | -1.79 (-1.87 to -1.72) |
| Namibia | 659 (470 to 905) | 102.83 (74.53 to 142) |  | 791 (614 to 1012) | 64.34 (49.72 to 82.23) | -1.52 (-1.65 to -1.4) |
| Nauru | 5 (4 to 6) | 149.05 (121.07 to 179.52) |  | 3 (2 to 4) | 98.54 (73.89 to 120.02) | -1.44 (-1.54 to -1.34) |
| Nepal | 19609 (15063 to 23557) | 273.02 (213.16 to 326.19) |  | 40800 (30907 to 48383) | 231.2 (175.79 to 270.35) | -0.53 (-0.58 to -0.48) |
| Netherlands | 6934 (6378 to 7449) | 33.47 (30.75 to 35.95) |  | 10766 (8586 to 12240) | 28.62 (22.8 to 32.43) | -0.56 (-0.74 to -0.38) |
| New Zealand | 1542 (1441 to 1659) | 39.27 (36.49 to 42.28) |  | 2396 (2025 to 2686) | 28.28 (24.04 to 31.59) | -1.12 (-1.37 to -0.86) |
| Nicaragua | 430 (380 to 482) | 29.16 (25.49 to 33.87) |  | 1343 (1011 to 1549) | 40.05 (29.96 to 46.03) | 1.1 (0.64 to 1.55) |
| Niger | 1973 (1518 to 2505) | 74.46 (57.15 to 98.42) |  | 3619 (2582 to 4954) | 52.11 (37.99 to 70.27) | -1.19 (-1.31 to -1.06) |
| Nigeria | 17749 (14085 to 21947) | 45.48 (36.02 to 56.61) |  | 24506 (19595 to 30356) | 33.92 (27.06 to 41.73) | -0.99 (-1.08 to -0.9) |
| Niue | 2 (2 to 3) | 95.82 (76.13 to 117.27) |  | 1 (1 to 2) | 58.07 (44.19 to 73.18) | -1.74 (-1.8 to -1.68) |
| North Macedonia | 659 (556 to 726) | 42.89 (35.31 to 47.91) |  | 652 (515 to 836) | 24.72 (19.85 to 31.66) | -2.26 (-3.04 to -1.47) |
| Northern Mariana Islands | 10 (9 to 12) | 81.71 (70.41 to 94.96) |  | 17 (15 to 21) | 49.27 (42.2 to 58.79) | -1.69 (-1.86 to -1.51) |
| Norway | 1523 (1414 to 1785) | 20.52 (19.06 to 23.97) |  | 2771 (2117 to 3026) | 25.85 (19.7 to 28.18) | 0.86 (0.72 to 1) |
| Oman | 185 (138 to 248) | 38.53 (28.77 to 51.86) |  | 244 (191 to 281) | 25.45 (19.45 to 29.63) | -1.51 (-2.02 to -1) |
| Pakistan | 64997 (52751 to 82334) | 130.64 (105.33 to 167.5) |  | 82758 (67880 to 102124) | 100.39 (83.09 to 122.7) | -0.86 (-0.92 to -0.8) |
| Palau | 12 (10 to 16) | 153 (123.52 to 193.39) |  | 17 (13 to 21) | 107.93 (87.06 to 130.08) | -1.17 (-1.23 to -1.12) |
| Palestine | 342 (265 to 448) | 44.35 (34.47 to 58.6) |  | 488 (405 to 569) | 26.93 (22.32 to 31.45) | -1.66 (-1.85 to -1.47) |
| Panama | 349 (312 to 392) | 24.17 (21.33 to 27.41) |  | 877 (661 to 1098) | 20.31 (15.31 to 25.48) | -0.55 (-0.84 to -0.25) |
| Papua New Guinea | 3338 (2739 to 4001) | 240.12 (190.92 to 286.42) |  | 7154 (5539 to 9108) | 209.49 (162.01 to 259.45) | -0.49 (-0.54 to -0.44) |
| Paraguay | 431 (375 to 552) | 20.67 (17.7 to 27.05) |  | 1148 (878 to 1464) | 22.12 (16.9 to 28.21) | 0.33 (-0.06 to 0.72) |
| Peru | 3320 (2825 to 4232) | 27 (22.75 to 36.91) |  | 6961 (5132 to 8990) | 21.31 (15.64 to 27.63) | -0.65 (-1.06 to -0.23) |
| Philippines | 20035 (17366 to 22256) | 82.95 (72.7 to 93.82) |  | 33229 (27252 to 41083) | 50.85 (41.71 to 64.72) | -1.49 (-1.75 to -1.23) |
| Poland | 14579 (13622 to 15101) | 34.65 (32.14 to 35.99) |  | 11349 (9488 to 13769) | 15.37 (12.87 to 18.69) | -2.99 (-3.22 to -2.75) |
| Portugal | 4624 (4279 to 5029) | 36.33 (33.31 to 39.27) |  | 7052 (5903 to 8062) | 23.69 (20.21 to 26.93) | -1.4 (-1.66 to -1.14) |
| Puerto Rico | 964 (863 to 1053) | 28.05 (24.87 to 30.7) |  | 1832 (1391 to 2281) | 21.92 (16.99 to 27.59) | -0.78 (-1.23 to -0.32) |
| Qatar | 21 (17 to 30) | 32.84 (25.42 to 47.51) |  | 66 (49 to 99) | 22.97 (17.71 to 32.41) | -1.24 (-1.53 to -0.95) |
| Republic of Korea | 11477 (8398 to 12518) | 62.49 (41.58 to 69.62) |  | 15506 (12626 to 17472) | 19.16 (15.35 to 21.58) | -4.07 (-4.35 to -3.78) |
| Republic of Moldova | 1830 (1501 to 1958) | 46.98 (37.85 to 50.47) |  | 971 (823 to 1200) | 16.75 (14.2 to 20.71) | -3.79 (-4.56 to -3.01) |
| Romania | 14278 (11364 to 15298) | 59.46 (46.15 to 64.04) |  | 8106 (6607 to 10101) | 20.77 (16.82 to 25.97) | -3.39 (-3.86 to -2.92) |
| Russian Federation | 57982 (47061 to 62626) | 34.55 (27.49 to 37.36) |  | 39390 (33695 to 46788) | 16.7 (14.31 to 19.87) | -2.33 (-3.17 to -1.48) |
| Rwanda | 2761 (2064 to 3390) | 97.56 (74.5 to 117.55) |  | 2520 (1928 to 3363) | 50.27 (39.13 to 66.02) | -2.35 (-2.49 to -2.21) |
| Saint Kitts and Nevis | 9 (8 to 10) | 24.76 (21.88 to 28.21) |  | 11 (9 to 13) | 20.2 (17.06 to 24.03) | -0.82 (-1.71 to 0.07) |
| Saint Lucia | 30 (27 to 33) | 35.91 (32.55 to 39.66) |  | 59 (48 to 70) | 28.94 (23.69 to 34.31) | -0.79 (-1.08 to -0.49) |
| Saint Vincent and the Grenadines | 11 (9 to 12) | 15.2 (13.41 to 17.44) |  | 20 (17 to 23) | 16.12 (13.65 to 18.48) | 0.27 (0.03 to 0.52) |
| Samoa | 104 (83 to 136) | 138.21 (110.64 to 180.52) |  | 105 (85 to 132) | 82.52 (67.1 to 103.26) | -1.74 (-1.8 to -1.67) |
| San Marino | 5 (4 to 6) | 15.82 (13.19 to 19.14) |  | 11 (7 to 14) | 13.39 (9.26 to 18.35) | -0.54 (-0.65 to -0.44) |
| Sao Tome and Principe | 60 (49 to 69) | 102.65 (84.86 to 118.29) |  | 79 (59 to 98) | 88.33 (66.73 to 108.64) | -0.48 (-0.68 to -0.29) |
| Saudi Arabia | 2561 (1938 to 3534) | 55.43 (41.43 to 75.66) |  | 3723 (2953 to 4532) | 31.74 (25.73 to 37.64) | -1.84 (-2.08 to -1.61) |
| Senegal | 1950 (1499 to 2346) | 65.76 (50.94 to 79.08) |  | 2763 (2158 to 3433) | 41.77 (33.46 to 50.91) | -1.58 (-1.97 to -1.19) |
| Serbia | 3395 (3019 to 3965) | 34.01 (30.13 to 40.16) |  | 3865 (3107 to 4773) | 25.15 (20.29 to 31.12) | -0.91 (-1.15 to -0.67) |
| Seychelles | 26 (23 to 29) | 46.9 (40.37 to 51.38) |  | 31 (26 to 35) | 32.67 (27.98 to 37.18) | -1.33 (-1.74 to -0.92) |
| Sierra Leone | 1186 (911 to 1461) | 65.07 (50.5 to 79.24) |  | 1479 (1108 to 1908) | 44.74 (34.38 to 56.56) | -1.25 (-1.36 to -1.13) |
| Singapore | 958 (824 to 1022) | 54.18 (46.25 to 58.02) |  | 750 (627 to 996) | 10.58 (8.82 to 14.07) | -5.85 (-6.17 to -5.53) |
| Slovakia | 1052 (946 to 1532) | 18.39 (16.52 to 26.51) |  | 1147 (901 to 1484) | 12.56 (9.9 to 16.2) | -1.31 (-1.57 to -1.05) |
| Slovenia | 673 (527 to 837) | 28.4 (22.3 to 35.13) |  | 639 (498 to 801) | 12.71 (9.9 to 16.07) | -2.98 (-3.34 to -2.61) |
| Solomon Islands | 187 (143 to 240) | 168.9 (131.64 to 217.12) |  | 347 (275 to 418) | 145.87 (118.53 to 169.97) | -0.5 (-0.72 to -0.28) |
| Somalia | 2126 (1435 to 3042) | 89.31 (62.19 to 133.51) |  | 3992 (2675 to 6428) | 66.97 (45.16 to 109.05) | -0.98 (-1.06 to -0.91) |
| South Africa | 12236 (10582 to 14044) | 62.79 (53.59 to 72.98) |  | 17827 (16467 to 20125) | 46.6 (42.43 to 51.97) | -0.81 (-1.19 to -0.42) |
| South Sudan | 1396 (917 to 1780) | 58.97 (40.41 to 75.45) |  | 1331 (952 to 1814) | 40.87 (29.4 to 53.86) | -1.24 (-1.29 to -1.19) |
| Spain | 22694 (20391 to 24152) | 42.29 (37.58 to 45.18) |  | 36560 (29180 to 42491) | 29.05 (24.12 to 33.19) | -1.22 (-1.42 to -1.01) |
| Sri Lanka | 9542 (8298 to 10391) | 113.98 (99.12 to 124.21) |  | 13597 (9536 to 17634) | 65.33 (46 to 83.62) | -1.53 (-2 to -1.07) |
| Sudan | 6244 (4005 to 8346) | 72.74 (45.49 to 100.4) |  | 7191 (4823 to 9718) | 45.18 (30.73 to 60.99) | -1.63 (-1.7 to -1.56) |
| Suriname | 63 (56 to 73) | 25.4 (22.74 to 29.09) |  | 117 (97 to 139) | 20.97 (17.35 to 24.94) | -1.21 (-2.82 to 0.43) |
| Sweden | 2481 (2271 to 2978) | 15.11 (13.86 to 18.05) |  | 4478 (3398 to 5046) | 17.85 (13.56 to 20.03) | 0.65 (0.58 to 0.72) |
| Switzerland | 2537 (2300 to 2744) | 22.84 (20.73 to 24.69) |  | 3074 (2563 to 3504) | 14.94 (12.58 to 16.87) | -1.45 (-1.56 to -1.35) |
| Syrian Arab Republic | 2224 (1804 to 2795) | 45.74 (36.85 to 59.76) |  | 3249 (2417 to 4430) | 35.02 (26.6 to 46.91) | -0.97 (-1.24 to -0.69) |
| Taiwan (Province of China) | 5150 (4562 to 5518) | 45.04 (39.14 to 48.76) |  | 9969 (7858 to 12656) | 24.42 (19.16 to 31.06) | -2.03 (-2.24 to -1.81) |
| Tajikistan | 1682 (1436 to 1852) | 61.52 (51.88 to 67.64) |  | 1669 (1337 to 2395) | 48.85 (38.61 to 72.69) | -0.55 (-0.81 to -0.28) |
| Thailand | 20374 (16503 to 23501) | 70.22 (56.47 to 81.16) |  | 23925 (17913 to 31300) | 24.83 (18.64 to 32.4) | -3.65 (-4.07 to -3.22) |
| Timor-Leste | 244 (184 to 311) | 99.34 (74.6 to 127.46) |  | 516 (391 to 651) | 77.66 (59.67 to 98.03) | -0.86 (-0.98 to -0.74) |
| Togo | 741 (591 to 881) | 65.75 (51.99 to 78.37) |  | 1332 (985 to 1729) | 43.17 (33.1 to 55.37) | -1.47 (-1.54 to -1.39) |
| Tokelau | 2 (1 to 2) | 124.05 (101.53 to 153.4) |  | 1 (1 to 1) | 63.5 (49.21 to 79.32) | -2.32 (-2.36 to -2.28) |
| Tonga | 36 (31 to 42) | 79.5 (66.4 to 92.07) |  | 42 (34 to 50) | 54.91 (44.13 to 65.8) | -1.12 (-1.7 to -0.54) |
| Trinidad and Tobago | 201 (188 to 218) | 25.67 (23.78 to 27.97) |  | 271 (201 to 360) | 15.58 (11.61 to 20.59) | -1.66 (-2.02 to -1.29) |
| Tunisia | 1381 (1119 to 1833) | 34.31 (27.61 to 45.45) |  | 2649 (1963 to 3507) | 24.02 (17.97 to 31.61) | -1.23 (-1.3 to -1.15) |
| Turkey | 18824 (15855 to 21343) | 60.16 (50.41 to 68.57) |  | 32981 (22668 to 40823) | 40.64 (28.01 to 50.33) | -1.38 (-1.63 to -1.12) |
| Turkmenistan | 843 (653 to 912) | 50.03 (38.3 to 54.39) |  | 442 (323 to 806) | 13.26 (9.64 to 25.01) | -5.16 (-5.82 to -4.5) |
| Tuvalu | 10 (8 to 12) | 167.79 (132.25 to 215.64) |  | 7 (6 to 10) | 87.49 (67.74 to 110.57) | -2.23 (-2.29 to -2.16) |
| Uganda | 4164 (2791 to 5910) | 68.16 (45.78 to 96.71) |  | 5480 (4037 to 7142) | 44.02 (33 to 56.25) | -1.54 (-1.62 to -1.47) |
| Ukraine | 33452 (26048 to 36237) | 49.76 (38.14 to 54.11) |  | 12181 (9742 to 18471) | 15.96 (12.78 to 24.16) | -3.91 (-4.63 to -3.19) |
| United Arab Emirates | 310 (232 to 417) | 77.58 (61.14 to 104.08) |  | 1778 (1216 to 2383) | 45.5 (35.6 to 58.31) | -2.03 (-2.27 to -1.8) |
| United Kingdom | 37657 (35478 to 43241) | 39.59 (37.28 to 45.62) |  | 51879 (43733 to 55229) | 36.54 (30.82 to 38.77) | -0.2 (-0.35 to -0.06) |
| United Republic of Tanzania | 4190 (3520 to 4867) | 41.44 (35.07 to 48.02) |  | 7001 (5759 to 8305) | 31.42 (25.67 to 36.63) | -0.94 (-1.03 to -0.85) |
| United States of America | 106098 (98990 to 124041) | 31.61 (29.57 to 36.81) |  | 224988 (181616 to 240468) | 37.72 (30.84 to 40.16) | 0.6 (0.54 to 0.67) |
| United States Virgin Islands | 14 (12 to 17) | 19.44 (16.36 to 23.27) |  | 31 (25 to 35) | 17.62 (14.45 to 20.34) | -0.23 (-0.48 to 0.03) |
| Uruguay | 1291 (1174 to 1538) | 33.38 (30.34 to 40.06) |  | 2155 (1888 to 2389) | 35.61 (31.35 to 39.19) | 0.32 (0.11 to 0.53) |
| Uzbekistan | 4929 (3868 to 5379) | 46.84 (36.58 to 51.27) |  | 3837 (3035 to 5646) | 28.98 (23.23 to 47.54) | -2.08 (-2.64 to -1.51) |
| Vanuatu | 90 (63 to 123) | 166.47 (119 to 227.78) |  | 172 (129 to 228) | 121.24 (92.27 to 156.46) | -1.13 (-1.28 to -0.99) |
| Venezuela (Bolivarian Republic of) | 2046 (1856 to 2303) | 23.29 (20.73 to 26.98) |  | 7062 (5421 to 9045) | 26.08 (20.01 to 33.27) | 0.33 (-0.17 to 0.84) |
| Viet Nam | 26078 (9883 to 33160) | 73.93 (27.59 to 93.74) |  | 36229 (14127 to 45192) | 47.62 (18.73 to 58.89) | -1.47 (-1.61 to -1.34) |
| Yemen | 3341 (2294 to 4736) | 79.73 (53.62 to 116.73) |  | 5738 (4433 to 7400) | 52.51 (41.42 to 66.84) | -1.44 (-1.51 to -1.38) |
| Zambia | 1570 (1283 to 1886) | 57.54 (47.35 to 70.37) |  | 2388 (1919 to 2919) | 41.65 (33.77 to 50.26) | -1.18 (-1.28 to -1.08) |
| Zimbabwe | 1820 (1560 to 2073) | 53.07 (45.66 to 60.13) |  | 2891 (2000 to 3774) | 49.07 (34.59 to 63.36) | -0.28 (-0.4 to -0.15) |

COPD: chronic obstructive pulmonary disease; AAPC: average annual percent change; UI: uncertainty interval; CI: confidence interval; SDI: Socio-demographic index.

Table S10. Mortality of asthma (1990/2019) for sexes, SDI and locations, with AAPC (1990-2019)

| GBD data | 1990 | |  | 2019 | | AAPC % (95% CI)  1990-2019 |
| --- | --- | --- | --- | --- | --- | --- |
|  | Cases (95% UI) | Age-standardised mortality per  100 000 population (95% UI) |  | Cases (95% UI) | Age-standardised mortality per  100 000 population (95% UI) |  |
| Global | 460012 (342616 to 599601) | 11.91 (8.8 to 15.86) |  | 461069 (366580 to 559006) | 5.8 (4.62 to 7.03) | -2.47 (-2.55 to -2.39) |
| **Sex** |  |  |  |  |  |  |
| Female | 227077 (152948 to 313979) | 10.67 (7.15 to 14.8) |  | 247395 (179849 to 320088) | 5.72 (4.16 to 7.39) | -2.13 (-2.23 to -2.04) |
| Male | 232935 (168385 to 323357) | 13.76 (9.82 to 19.51) |  | 213674 (174060 to 260238) | 5.93 (4.84 to 7.21) | -2.87 (-2.98 to -2.77) |
| **SDI** |  |  |  |  |  |  |
| High SDI | 36673 (32602 to 39104) | 3.57 (3.17 to 3.81) |  | 17959 (15624 to 20181) | 0.96 (0.86 to 1.07) | -4.47 (-4.68 to -4.27) |
| High-middle SDI | 53394 (45389 to 64001) | 5.5 (4.64 to 6.65) |  | 35675 (30692 to 40377) | 1.83 (1.57 to 2.07) | -3.68 (-3.85 to -3.5) |
| Middle SDI | 118020 (92350 to 150266) | 13.26 (10.19 to 17.46) |  | 116850 (100287 to 131918) | 5.33 (4.55 to 6.02) | -3.13 (-3.18 to -3.07) |
| Low-middle SDI | 175687 (118671 to 247703) | 32.29 (21.25 to 48.01) |  | 200060 (143312 to 257378) | 16.29 (11.6 to 21.33) | -2.31 (-2.52 to -2.1) |
| Low SDI | 75923 (52687 to 102487) | 33.26 (22.16 to 49.06) |  | 90180 (65746 to 127842) | 18.94 (13.35 to 28.66) | -2 (-2.07 to -1.93) |
| **Region** |  |  |  |  |  |  |
| High-income Asia Pacific | 12381 (9710 to 13495) | 7.03 (5.44 to 7.69) |  | 4948 (3761 to 6267) | 0.83 (0.66 to 1.04) | -7.38 (-7.75 to -7.01) |
| High-income North America | 5525 (5095 to 6360) | 1.65 (1.53 to 1.88) |  | 4373 (3593 to 4701) | 0.83 (0.7 to 0.88) | -2.31 (-2.44 to -2.18) |
| Western Europe | 17558 (15989 to 19074) | 3.11 (2.83 to 3.36) |  | 6902 (5733 to 7827) | 0.7 (0.61 to 0.79) | -5.08 (-5.29 to -4.88) |
| Australasia | 979 (880 to 1057) | 4.33 (3.88 to 4.67) |  | 545 (448 to 636) | 1.14 (0.96 to 1.31) | -4.59 (-4.78 to -4.4) |
| Andean Latin America | 1291 (912 to 1615) | 4.13 (3.09 to 5.01) |  | 521 (404 to 660) | 0.95 (0.73 to 1.19) | -5.06 (-5.34 to -4.77) |
| Tropical Latin America | 3284 (2881 to 3590) | 3.45 (3.01 to 3.79) |  | 2940 (2613 to 3448) | 1.28 (1.14 to 1.5) | -3.3 (-3.51 to -3.09) |
| Central Latin America | 5747 (4899 to 6167) | 6.52 (5.64 to 7.02) |  | 3419 (2810 to 4047) | 1.49 (1.23 to 1.76) | -5.12 (-5.35 to -4.88) |
| Southern Latin America | 1256 (1102 to 1408) | 2.79 (2.45 to 3.12) |  | 884 (731 to 1006) | 1.07 (0.89 to 1.21) | -3.25 (-3.52 to -2.98) |
| Caribbean | 2394 (1877 to 2906) | 8.06 (6.35 to 10.09) |  | 2233 (1693 to 2832) | 4.54 (3.43 to 5.83) | -1.84 (-1.99 to -1.68) |
| Central Europe | 6357 (5777 to 6823) | 4.63 (4.2 to 5) |  | 1776 (1509 to 2139) | 0.81 (0.69 to 0.98) | -5.91 (-6.06 to -5.75) |
| Eastern Europe | 11364 (9335 to 12433) | 4.2 (3.43 to 4.62) |  | 2326 (1964 to 3410) | 0.7 (0.59 to 1.02) | -5.96 (-6.92 to -4.99) |
| Central Asia | 4474 (3676 to 5323) | 10.26 (8.3 to 12.43) |  | 3342 (2869 to 4053) | 5.42 (4.69 to 6.54) | -1.97 (-2.33 to -1.62) |
| North Africa and Middle East | 32419 (26002 to 42144) | 20.45 (15.9 to 28.03) |  | 32076 (26201 to 38345) | 8.39 (6.86 to 9.92) | -3.02 (-3.11 to -2.92) |
| South Asia | 185115 (118302 to 278666) | 38.82 (24.26 to 61.63) |  | 232188 (160834 to 316297) | 18.95 (12.92 to 26.43) | -2.51 (-2.7 to -2.32) |
| Southeast Asia | 72711 (55589 to 86985) | 30.93 (22.89 to 38.63) |  | 72055 (61159 to 81555) | 13.79 (11.64 to 15.64) | -2.73 (-2.8 to -2.67) |
| East Asia | 43493 (33085 to 61961) | 6.6 (4.92 to 9.65) |  | 27211 (22727 to 33033) | 1.59 (1.33 to 1.93) | -4.92 (-5.08 to -4.76) |
| Oceania | 1596 (1256 to 2119) | 67.94 (51.62 to 94.5) |  | 2556 (1891 to 3446) | 46.76 (34.38 to 63.61) | -1.3 (-1.35 to -1.26) |
| Western Sub-Saharan Africa | 19946 (15906 to 23957) | 23.04 (18.35 to 28.45) |  | 24108 (19908 to 29110) | 13.13 (10.95 to 15.72) | -1.91 (-1.96 to -1.85) |
| Eastern Sub-Saharan Africa | 18628 (14007 to 22321) | 20.73 (15.29 to 28.44) |  | 19765 (15361 to 28237) | 11.29 (8.54 to 17.52) | -2.07 (-2.11 to -2.03) |
| Central Sub-Saharan Africa | 7099 (4636 to 10330) | 31.2 (18.83 to 55.74) |  | 9792 (6176 to 17107) | 20.63 (12.04 to 41.64) | -1.42 (-1.49 to -1.35) |
| Southern Sub-Saharan Africa | 6393 (5497 to 7601) | 22.72 (19.1 to 27.97) |  | 7109 (6295 to 8293) | 13.78 (12.14 to 16.11) | -1.59 (-1.96 to -1.22) |
| **Countries** |  |  |  |  |  |  |
| Afghanistan | 2968 (1906 to 4266) | 45.35 (27.95 to 67.56) |  | 2947 (1954 to 4134) | 25.78 (15.93 to 36.64) | -1.87 (-1.96 to -1.78) |
| Albania | 204 (156 to 251) | 11.72 (8.98 to 14.84) |  | 143 (99 to 199) | 3.41 (2.36 to 4.72) | -4.28 (-4.69 to -3.86) |
| Algeria | 2122 (1569 to 2805) | 23.32 (17.09 to 30.94) |  | 2250 (1728 to 2848) | 8.53 (6.5 to 10.73) | -3.37 (-3.44 to -3.3) |
| American Samoa | 4 (3 to 5) | 21.82 (17.74 to 26.89) |  | 4 (3 to 5) | 11 (8.64 to 13.95) | -2.33 (-2.76 to -1.9) |
| Andorra | 1 (1 to 1) | 1.84 (1.34 to 2.56) |  | 1 (1 to 1) | 0.7 (0.46 to 1.02) | -3.3 (-3.45 to -3.15) |
| Angola | 1356 (953 to 1881) | 32.12 (20.81 to 54.91) |  | 1485 (1048 to 2100) | 14.54 (10.25 to 23.21) | -2.78 (-2.95 to -2.6) |
| Antigua and Barbuda | 2 (1 to 2) | 2.9 (2.52 to 3.37) |  | 2 (1 to 2) | 1.95 (1.53 to 2.39) | -1.28 (-1.81 to -0.74) |
| Argentina | 769 (654 to 896) | 2.42 (2.06 to 2.82) |  | 491 (407 to 572) | 0.92 (0.77 to 1.07) | -3.31 (-3.61 to -3.02) |
| Armenia | 59 (43 to 74) | 2.41 (1.76 to 3.08) |  | 18 (14 to 23) | 0.45 (0.35 to 0.57) | -6.16 (-7.05 to -5.26) |
| Australia | 813 (723 to 881) | 4.32 (3.83 to 4.65) |  | 468 (380 to 549) | 1.14 (0.95 to 1.32) | -4.5 (-4.77 to -4.23) |
| Austria | 408 (371 to 456) | 3.39 (3.07 to 3.77) |  | 78 (63 to 91) | 0.41 (0.34 to 0.48) | -7.19 (-7.87 to -6.5) |
| Azerbaijan | 449 (352 to 596) | 9.67 (7.43 to 13.23) |  | 364 (259 to 591) | 5.34 (3.57 to 8.5) | -1.91 (-2.18 to -1.64) |
| Bahamas | 6 (5 to 7) | 3.68 (3.14 to 4.31) |  | 8 (6 to 10) | 2.06 (1.61 to 2.62) | -1.93 (-2.25 to -1.61) |
| Bahrain | 23 (19 to 29) | 14.96 (12.21 to 19.22) |  | 31 (24 to 42) | 4.87 (3.81 to 6.53) | -4.19 (-4.67 to -3.71) |
| Bangladesh | 15262 (10819 to 22340) | 32.58 (22.28 to 49.86) |  | 10719 (7475 to 17539) | 8.83 (6.16 to 14.55) | -4.22 (-4.61 to -3.84) |
| Barbados | 13 (11 to 15) | 4.36 (3.7 to 5.02) |  | 13 (10 to 16) | 2.84 (2.23 to 3.5) | -1.5 (-2.03 to -0.97) |
| Belarus | 907 (700 to 1152) | 7.18 (5.59 to 9.14) |  | 127 (80 to 287) | 0.8 (0.5 to 1.82) | -7.4 (-8.55 to -6.24) |
| Belgium | 454 (408 to 505) | 3.12 (2.82 to 3.45) |  | 150 (119 to 176) | 0.65 (0.54 to 0.75) | -5.46 (-5.85 to -5.07) |
| Belize | 4 (4 to 5) | 3.59 (2.92 to 4.48) |  | 8 (7 to 10) | 2.7 (2.21 to 3.27) | -0.5 (-1.07 to 0.07) |
| Benin | 609 (468 to 768) | 29.09 (22.15 to 37.16) |  | 731 (541 to 985) | 14.83 (11.28 to 19.63) | -2.28 (-2.41 to -2.15) |
| Bermuda | 2 (1 to 2) | 3.3 (2.51 to 4.04) |  | 1 (1 to 1) | 0.88 (0.7 to 1.1) | -4.42 (-4.65 to -4.18) |
| Bhutan | 75 (43 to 129) | 34.27 (19.55 to 64.26) |  | 75 (52 to 113) | 15.23 (10.73 to 22.81) | -2.76 (-2.81 to -2.71) |
| Bolivia (Plurinational State of) | 431 (265 to 638) | 8.49 (5.65 to 11.41) |  | 198 (146 to 261) | 2.43 (1.77 to 3.2) | -4.21 (-4.43 to -3.98) |
| Bosnia and Herzegovina | 89 (62 to 120) | 2.75 (1.9 to 3.83) |  | 57 (43 to 74) | 0.99 (0.75 to 1.29) | -3.45 (-5.27 to -1.6) |
| Botswana | 185 (129 to 258) | 34.09 (23.8 to 48.04) |  | 202 (129 to 287) | 16 (9.95 to 22.76) | -2.62 (-2.77 to -2.46) |
| Brazil | 3226 (2838 to 3524) | 3.48 (3.04 to 3.83) |  | 2849 (2542 to 3360) | 1.27 (1.13 to 1.5) | -3.35 (-3.58 to -3.13) |
| Brunei Darussalam | 18 (14 to 21) | 30.45 (21.01 to 37.4) |  | 14 (12 to 17) | 10.94 (8.63 to 13.46) | -3.31 (-3.6 to -3.01) |
| Bulgaria | 264 (233 to 297) | 2.36 (2.07 to 2.63) |  | 52 (38 to 68) | 0.37 (0.27 to 0.48) | -6.31 (-6.79 to -5.82) |
| Burkina Faso | 789 (605 to 977) | 17.82 (13.66 to 22.3) |  | 1108 (842 to 1425) | 11.42 (8.93 to 14.71) | -1.42 (-1.61 to -1.22) |
| Burundi | 920 (593 to 1239) | 31.09 (20.06 to 44.58) |  | 815 (542 to 1326) | 16.28 (10.44 to 28.78) | -2.25 (-2.36 to -2.15) |
| Cabo Verde | 42 (25 to 52) | 17.64 (10.45 to 21.77) |  | 27 (19 to 38) | 6.34 (4.49 to 8.88) | -3.76 (-4.18 to -3.34) |
| Cambodia | 1136 (852 to 1371) | 26.22 (18.47 to 34.26) |  | 1359 (1039 to 1675) | 13.67 (10.42 to 16.71) | -2.19 (-2.28 to -2.1) |
| Cameroon | 1182 (941 to 1441) | 27.63 (21.89 to 33.8) |  | 1506 (1096 to 2033) | 13.07 (9.92 to 17.2) | -2.6 (-2.71 to -2.49) |
| Canada | 511 (459 to 565) | 1.63 (1.47 to 1.8) |  | 300 (232 to 358) | 0.45 (0.36 to 0.53) | -4.46 (-4.79 to -4.14) |
| Central African Republic | 500 (327 to 724) | 43.94 (26.1 to 77.67) |  | 710 (429 to 1225) | 35.4 (19.28 to 74.16) | -0.75 (-0.83 to -0.66) |
| Chad | 890 (642 to 1176) | 31.13 (22.2 to 42.84) |  | 1156 (876 to 1505) | 19.69 (14.94 to 25.84) | -1.55 (-1.63 to -1.48) |
| Chile | 261 (219 to 290) | 2.86 (2.37 to 3.18) |  | 270 (210 to 333) | 1.15 (0.89 to 1.41) | -2.85 (-3.33 to -2.36) |
| China | 40414 (30326 to 58426) | 6.37 (4.69 to 9.45) |  | 24750 (20245 to 30769) | 1.51 (1.23 to 1.86) | -4.96 (-5.12 to -4.8) |
| Colombia | 759 (661 to 822) | 4.21 (3.59 to 4.57) |  | 264 (192 to 347) | 0.5 (0.36 to 0.66) | -7.33 (-7.9 to -6.77) |
| Comoros | 50 (25 to 72) | 20.89 (12.06 to 30.22) |  | 50 (36 to 70) | 10.64 (7.77 to 15.19) | -2.22 (-2.53 to -1.9) |
| Congo | 325 (221 to 501) | 32.69 (20.97 to 58.25) |  | 325 (216 to 463) | 14.57 (9.84 to 22.4) | -2.7 (-2.92 to -2.49) |
| Cook Islands | 2 (1 to 2) | 15.65 (11.64 to 19.99) |  | 1 (1 to 2) | 6.02 (4.11 to 8.35) | -3.15 (-3.42 to -2.88) |
| Costa Rica | 45 (40 to 53) | 2.33 (2.01 to 2.74) |  | 51 (37 to 67) | 0.99 (0.72 to 1.3) | -2.77 (-3.47 to -2.07) |
| Croatia | 256 (229 to 284) | 4.48 (3.99 to 4.99) |  | 55 (42 to 70) | 0.59 (0.45 to 0.75) | -6.8 (-7.37 to -6.23) |
| Cuba | 260 (218 to 288) | 2.45 (2.05 to 2.71) |  | 261 (194 to 332) | 1.52 (1.16 to 1.93) | -1.51 (-2.57 to -0.45) |
| Cyprus | 44 (31 to 58) | 8.26 (5.58 to 11.33) |  | 29 (21 to 43) | 1.93 (1.31 to 3.05) | -5.02 (-5.73 to -4.3) |
| Czechia | 265 (242 to 306) | 2.02 (1.85 to 2.32) |  | 117 (89 to 146) | 0.59 (0.45 to 0.73) | -4.07 (-4.38 to -3.76) |
| Côte d’Ivoire | 1096 (830 to 1366) | 27.34 (20.67 to 33.81) |  | 1410 (1054 to 1869) | 13.73 (10.78 to 17.63) | -2.36 (-2.55 to -2.16) |
| Democratic People's Republic of Korea | 1294 (858 to 1868) | 10.24 (6.58 to 15.41) |  | 1636 (1073 to 2524) | 5.69 (3.66 to 8.99) | -2.03 (-2.16 to -1.89) |
| Democratic Republic of the Congo | 4736 (2913 to 7208) | 30.26 (17.09 to 55.72) |  | 7141 (4144 to 13525) | 22.48 (11.83 to 48.44) | -1.01 (-1.1 to -0.93) |
| Denmark | 229 (198 to 263) | 2.94 (2.57 to 3.33) |  | 70 (57 to 84) | 0.63 (0.52 to 0.73) | -5.2 (-5.69 to -4.7) |
| Djibouti | 29 (19 to 41) | 17.09 (11.05 to 23.43) |  | 50 (29 to 72) | 8.89 (4.76 to 12.31) | -2.26 (-2.37 to -2.14) |
| Dominica | 5 (4 to 5) | 6.34 (5.32 to 7.66) |  | 4 (3 to 6) | 5.05 (3.8 to 6.49) | -0.78 (-0.93 to -0.63) |
| Dominican Republic | 486 (375 to 569) | 10.6 (8.21 to 12.13) |  | 477 (346 to 633) | 5.17 (3.75 to 6.84) | -2.18 (-2.98 to -1.37) |
| Ecuador | 339 (192 to 437) | 4.21 (2.85 to 5.42) |  | 94 (74 to 125) | 0.7 (0.55 to 0.92) | -6.34 (-6.81 to -5.88) |
| Egypt | 7950 (6154 to 9827) | 28.54 (22.47 to 36.04) |  | 7490 (5033 to 10491) | 13.66 (9.43 to 18.75) | -2.3 (-2.4 to -2.19) |
| El Salvador | 351 (253 to 423) | 9.86 (7.52 to 12.09) |  | 174 (124 to 230) | 2.75 (1.95 to 3.64) | -4.43 (-4.94 to -3.92) |
| Equatorial Guinea | 73 (45 to 117) | 38.01 (21.65 to 72.17) |  | 45 (26 to 83) | 10.51 (6.07 to 20.36) | -4.42 (-4.64 to -4.21) |
| Eritrea | 363 (224 to 482) | 28.31 (18.11 to 41.66) |  | 422 (282 to 599) | 15.18 (9.86 to 21.64) | -2.08 (-2.29 to -1.86) |
| Estonia | 81 (70 to 95) | 4.02 (3.49 to 4.68) |  | 30 (22 to 41) | 1.06 (0.8 to 1.45) | -4.75 (-5.8 to -3.7) |
| Eswatini | 102 (74 to 139) | 34.58 (24.6 to 48.53) |  | 108 (78 to 146) | 20.02 (14.78 to 26.49) | -1.9 (-2.08 to -1.71) |
| Ethiopia | 5394 (3644 to 6747) | 22.71 (15.51 to 31.92) |  | 3699 (2744 to 5827) | 8.54 (6.21 to 14.61) | -3.37 (-3.52 to -3.22) |
| Fiji | 268 (214 to 335) | 95.03 (76.38 to 119.1) |  | 243 (188 to 315) | 43.33 (34.22 to 54.76) | -2.74 (-2.99 to -2.49) |
| Finland | 111 (95 to 134) | 1.63 (1.4 to 1.95) |  | 110 (85 to 135) | 0.78 (0.62 to 0.94) | -2.57 (-2.85 to -2.29) |
| France | 2290 (2030 to 2508) | 2.8 (2.51 to 3.05) |  | 1138 (898 to 1477) | 0.73 (0.6 to 0.95) | -4.7 (-4.96 to -4.45) |
| Gabon | 110 (80 to 150) | 21.21 (15.16 to 31.27) |  | 87 (62 to 116) | 9.55 (6.88 to 12.86) | -2.68 (-2.8 to -2.57) |
| Gambia | 90 (64 to 120) | 25.97 (18.73 to 34.23) |  | 153 (112 to 205) | 16.48 (12.06 to 22.24) | -1.61 (-1.87 to -1.36) |
| Georgia | 376 (309 to 446) | 6.65 (5.49 to 7.89) |  | 116 (91 to 156) | 1.94 (1.54 to 2.54) | -3.68 (-5.48 to -1.85) |
| Germany | 6378 (5424 to 7365) | 5.08 (4.34 to 5.8) |  | 1243 (1046 to 1468) | 0.64 (0.55 to 0.75) | -7.08 (-7.49 to -6.68) |
| Ghana | 1484 (941 to 1838) | 24.02 (15.91 to 29.95) |  | 2120 (1404 to 2750) | 13.97 (9.32 to 17.77) | -1.84 (-2 to -1.67) |
| Greece | 177 (157 to 200) | 1.24 (1.1 to 1.39) |  | 67 (51 to 82) | 0.26 (0.21 to 0.31) | -5.59 (-6.48 to -4.69) |
| Greenland | 2 (2 to 3) | 6.65 (5.32 to 8.75) |  | 1 (1 to 2) | 1.93 (1.48 to 2.65) | -4.25 (-4.61 to -3.88) |
| Grenada | 3 (3 to 4) | 4.52 (3.76 to 5.42) |  | 2 (2 to 3) | 2.03 (1.65 to 2.4) | -2.81 (-3.18 to -2.44) |
| Guam | 5 (4 to 5) | 9.87 (7.79 to 11.89) |  | 7 (6 to 10) | 4.03 (3.1 to 5.18) | -3.88 (-5.01 to -2.73) |
| Guatemala | 732 (532 to 857) | 14.35 (11.18 to 17.53) |  | 327 (247 to 438) | 2.97 (2.27 to 3.95) | -5.63 (-6.44 to -4.8) |
| Guinea | 1068 (803 to 1363) | 31.92 (24.07 to 42.67) |  | 1136 (840 to 1471) | 19.9 (14.76 to 26.13) | -1.59 (-1.69 to -1.49) |
| Guinea-Bissau | 182 (138 to 231) | 43.23 (32.99 to 53.56) |  | 162 (121 to 210) | 22.44 (16.74 to 28.95) | -2.25 (-2.34 to -2.16) |
| Guyana | 36 (31 to 42) | 8.55 (7.21 to 10.14) |  | 29 (22 to 38) | 4.55 (3.43 to 5.94) | -2.14 (-2.45 to -1.83) |
| Haiti | 1156 (748 to 1641) | 26.29 (15.46 to 47.01) |  | 1094 (674 to 1665) | 13.08 (7.57 to 22.31) | -2.37 (-2.44 to -2.31) |
| Honduras | 383 (266 to 497) | 11.82 (8.62 to 15.57) |  | 372 (240 to 527) | 6.19 (4 to 8.71) | -1.96 (-2.37 to -1.55) |
| Hungary | 326 (301 to 375) | 2.32 (2.14 to 2.63) |  | 110 (85 to 137) | 0.58 (0.46 to 0.72) | -4.82 (-5.48 to -4.14) |
| Iceland | 6 (6 to 7) | 2.2 (1.94 to 2.53) |  | 3 (2 to 3) | 0.5 (0.39 to 0.59) | -5.12 (-5.34 to -4.9) |
| India | 149455 (90391 to 227979) | 41.98 (23.9 to 67.5) |  | 198799 (129622 to 271916) | 20.05 (12.9 to 28.19) | -2.61 (-2.85 to -2.36) |
| Indonesia | 24274 (19536 to 30695) | 26.58 (21.16 to 35.81) |  | 26458 (21282 to 32820) | 14.87 (11.99 to 18.55) | -1.99 (-2.08 to -1.91) |
| Iran (Islamic Republic of) | 3806 (3075 to 4777) | 18.52 (14 to 24.95) |  | 3606 (3095 to 4030) | 5.61 (4.77 to 6.25) | -4.08 (-4.21 to -3.95) |
| Iraq | 1166 (904 to 1469) | 14.93 (11.56 to 19.32) |  | 1157 (891 to 1474) | 5.71 (4.5 to 7.32) | -3.59 (-4.23 to -2.95) |
| Ireland | 136 (117 to 155) | 3.46 (3.02 to 3.95) |  | 50 (41 to 62) | 0.7 (0.58 to 0.86) | -5.35 (-5.63 to -5.06) |
| Israel | 207 (178 to 240) | 4.43 (3.82 to 5.12) |  | 134 (110 to 178) | 1.11 (0.92 to 1.46) | -4.7 (-5.19 to -4.21) |
| Italy | 1962 (1799 to 2134) | 2.26 (2.06 to 2.46) |  | 537 (428 to 609) | 0.32 (0.27 to 0.37) | -6.73 (-7.06 to -6.39) |
| Jamaica | 76 (66 to 88) | 3.86 (3.35 to 4.55) |  | 77 (58 to 101) | 2.54 (1.92 to 3.32) | -1.1 (-2.14 to -0.04) |
| Japan | 6103 (5440 to 6539) | 4.04 (3.59 to 4.32) |  | 2215 (1592 to 3000) | 0.46 (0.36 to 0.62) | -7.47 (-7.84 to -7.11) |
| Jordan | 113 (90 to 137) | 9.28 (7.24 to 11.65) |  | 143 (116 to 178) | 2.61 (2.09 to 3.28) | -4.39 (-4.79 to -3.99) |
| Kazakhstan | 1333 (1015 to 1737) | 11.44 (8.66 to 15.07) |  | 1259 (929 to 1612) | 8.44 (6.2 to 10.86) | -0.55 (-1.35 to 0.25) |
| Kenya | 1195 (808 to 1926) | 13.3 (8.54 to 23.7) |  | 2171 (1448 to 3749) | 10.17 (6.69 to 18.61) | -0.9 (-0.99 to -0.82) |
| Kiribati | 33 (27 to 40) | 105.34 (84.79 to 140.79) |  | 40 (30 to 51) | 80.5 (59.22 to 104.19) | -0.94 (-1 to -0.87) |
| Kuwait | 33 (28 to 38) | 5.57 (4.5 to 6.62) |  | 33 (26 to 42) | 1.46 (1.14 to 1.85) | -3.99 (-5.25 to -2.72) |
| Kyrgyzstan | 156 (122 to 191) | 5.13 (3.99 to 6.3) |  | 57 (46 to 81) | 1.32 (1.08 to 1.85) | -5.02 (-5.91 to -4.12) |
| Lao People's Democratic Republic | 1185 (865 to 1491) | 53.4 (38.29 to 71.76) |  | 821 (591 to 1126) | 21.11 (15.24 to 29.06) | -3.19 (-3.24 to -3.14) |
| Latvia | 169 (144 to 198) | 4.76 (4.06 to 5.54) |  | 35 (27 to 54) | 0.86 (0.65 to 1.3) | -6.18 (-7.33 to -5.02) |
| Lebanon | 292 (217 to 409) | 15.24 (11.15 to 21.34) |  | 279 (177 to 376) | 5.45 (3.47 to 7.37) | -3.49 (-3.6 to -3.38) |
| Lesotho | 341 (215 to 544) | 36.09 (22.52 to 58.36) |  | 356 (232 to 526) | 29.85 (19.61 to 46.08) | -0.51 (-0.79 to -0.23) |
| Liberia | 206 (161 to 257) | 17.84 (14.16 to 22.57) |  | 200 (139 to 284) | 10.08 (7.14 to 14.08) | -1.82 (-2.19 to -1.45) |
| Libya | 253 (177 to 359) | 14.4 (9.77 to 21.02) |  | 350 (241 to 471) | 7.67 (5.33 to 10.4) | -1.95 (-2.3 to -1.59) |
| Lithuania | 123 (102 to 146) | 2.75 (2.27 to 3.24) |  | 37 (28 to 52) | 0.63 (0.48 to 0.88) | -5.03 (-5.57 to -4.48) |
| Luxembourg | 23 (20 to 26) | 4.35 (3.84 to 4.87) |  | 10 (8 to 12) | 0.98 (0.78 to 1.21) | -5.15 (-5.34 to -4.96) |
| Madagascar | 1949 (1563 to 2343) | 27.99 (21.82 to 37.2) |  | 2361 (1642 to 3241) | 19.29 (13.32 to 26.64) | -1.22 (-1.37 to -1.08) |
| Malawi | 799 (557 to 1043) | 17.33 (12.31 to 22.62) |  | 818 (586 to 1089) | 10.8 (7.51 to 15.25) | -1.6 (-1.66 to -1.53) |
| Malaysia | 1510 (1220 to 1809) | 16.75 (13.5 to 20.54) |  | 939 (681 to 1412) | 3.83 (2.77 to 5.79) | -5.12 (-5.76 to -4.47) |
| Maldives | 20 (13 to 27) | 23.37 (15.56 to 33.94) |  | 10 (8 to 13) | 4.2 (3.31 to 5.24) | -5.94 (-6.24 to -5.64) |
| Mali | 1406 (977 to 1748) | 33.92 (23.45 to 42.63) |  | 1794 (1240 to 2473) | 20.15 (14.01 to 26.86) | -1.72 (-1.83 to -1.62) |
| Malta | 9 (8 to 11) | 2.32 (2.04 to 2.67) |  | 5 (4 to 6) | 0.53 (0.43 to 0.66) | -5.01 (-5.17 to -4.85) |
| Marshall Islands | 7 (5 to 10) | 47.22 (34.45 to 71.35) |  | 7 (5 to 10) | 27.2 (18.48 to 37.53) | -1.93 (-2.14 to -1.73) |
| Mauritania | 237 (164 to 300) | 24.75 (17.04 to 31.45) |  | 174 (124 to 239) | 8.93 (6.4 to 11.96) | -3.53 (-3.66 to -3.39) |
| Mauritius | 243 (208 to 266) | 38.43 (32.58 to 42.19) |  | 147 (113 to 197) | 9.55 (7.38 to 12.72) | -5.17 (-5.79 to -4.54) |
| Mexico | 2871 (2521 to 3052) | 7.36 (6.36 to 7.84) |  | 1655 (1342 to 1931) | 1.52 (1.23 to 1.77) | -5.42 (-5.73 to -5.11) |
| Micronesia (Federated States of) | 21 (16 to 29) | 53.47 (38.58 to 80.2) |  | 14 (10 to 20) | 27.69 (20.23 to 37.7) | -2.25 (-2.3 to -2.2) |
| Monaco | 1 (0 to 1) | 0.83 (0.63 to 1.06) |  | 1 (0 to 1) | 0.55 (0.42 to 0.68) | -1.37 (-1.48 to -1.26) |
| Mongolia | 153 (113 to 199) | 16.78 (12.42 to 22.24) |  | 105 (78 to 154) | 6.31 (4.7 to 9.05) | -3.45 (-3.7 to -3.19) |
| Montenegro | 3 (2 to 4) | 0.53 (0.42 to 0.75) |  | 3 (2 to 4) | 0.34 (0.25 to 0.44) | -1.33 (-1.53 to -1.12) |
| Morocco | 2515 (1828 to 3698) | 21.05 (14.89 to 33.03) |  | 3054 (2255 to 4073) | 11.76 (8.62 to 15.83) | -1.96 (-2.63 to -1.28) |
| Mozambique | 1124 (765 to 1444) | 16.45 (11.7 to 21.64) |  | 1389 (1004 to 1836) | 11.92 (8.36 to 16.19) | -1.11 (-1.24 to -0.98) |
| Myanmar | 16004 (10752 to 20423) | 74.11 (47.22 to 98.38) |  | 11043 (7905 to 13820) | 27.8 (19.51 to 34.4) | -3.35 (-3.44 to -3.27) |
| Namibia | 240 (169 to 347) | 35.19 (24.91 to 51.78) |  | 210 (156 to 281) | 15.94 (11.86 to 21.26) | -2.63 (-2.76 to -2.49) |
| Nauru | 1 (1 to 2) | 36.89 (27.7 to 46.32) |  | 1 (0 to 1) | 21.13 (13.06 to 28.16) | -1.9 (-2.07 to -1.73) |
| Nepal | 5145 (3022 to 7572) | 63.26 (35.89 to 101.19) |  | 6901 (4301 to 9793) | 36.4 (22.45 to 52.15) | -1.85 (-1.96 to -1.74) |
| Netherlands | 235 (210 to 259) | 1.21 (1.09 to 1.34) |  | 116 (86 to 139) | 0.35 (0.27 to 0.41) | -4.03 (-4.51 to -3.55) |
| New Zealand | 165 (150 to 182) | 4.4 (3.98 to 4.83) |  | 77 (65 to 88) | 1.12 (0.98 to 1.26) | -4.64 (-5.14 to -4.13) |
| Nicaragua | 149 (122 to 179) | 7.09 (5.94 to 8.34) |  | 101 (77 to 138) | 2.59 (1.98 to 3.5) | -3.74 (-4.2 to -3.28) |
| Niger | 1034 (740 to 1319) | 34.08 (24.1 to 46.73) |  | 1556 (1059 to 2241) | 19.47 (13.42 to 28.3) | -1.88 (-1.97 to -1.79) |
| Nigeria | 7818 (5952 to 9992) | 18.6 (13.95 to 24.21) |  | 8746 (6849 to 11135) | 10.72 (8.46 to 13.61) | -1.89 (-2.01 to -1.78) |
| Niue | 1 (0 to 1) | 23.89 (16.67 to 30.47) |  | 0 (0 to 0) | 11.04 (6.24 to 15.55) | -2.65 (-2.7 to -2.59) |
| North Macedonia | 137 (111 to 166) | 8.85 (6.94 to 10.99) |  | 58 (44 to 74) | 2.16 (1.64 to 2.75) | -4.93 (-5.54 to -4.32) |
| Northern Mariana Islands | 2 (1 to 2) | 13.73 (10.91 to 17.03) |  | 2 (2 to 3) | 6.65 (5.24 to 8.47) | -2.25 (-3.29 to -1.19) |
| Norway | 349 (321 to 399) | 4.95 (4.56 to 5.63) |  | 97 (76 to 110) | 0.92 (0.71 to 1.04) | -5.95 (-6.34 to -5.57) |
| Oman | 11 (7 to 17) | 1.87 (1.24 to 3.09) |  | 7 (6 to 9) | 0.64 (0.48 to 0.84) | -3.35 (-3.84 to -2.86) |
| Pakistan | 15178 (10101 to 24483) | 28.09 (18.24 to 47.46) |  | 15694 (12143 to 21426) | 16.39 (12.59 to 22.93) | -1.82 (-1.89 to -1.75) |
| Palau | 2 (1 to 2) | 22.64 (17.22 to 30.22) |  | 2 (1 to 2) | 12.81 (10 to 15.92) | -1.9 (-2.02 to -1.77) |
| Palestine | 107 (77 to 140) | 13.09 (9.35 to 17.52) |  | 99 (76 to 120) | 5.21 (3.93 to 6.33) | -3.3 (-3.72 to -2.89) |
| Panama | 64 (53 to 73) | 3.57 (3.05 to 4.11) |  | 61 (44 to 78) | 1.45 (1.06 to 1.87) | -3.14 (-3.37 to -2.91) |
| Papua New Guinea | 1038 (767 to 1453) | 72.76 (51.51 to 107.82) |  | 1940 (1370 to 2753) | 55.81 (39.05 to 80.31) | -0.92 (-0.98 to -0.87) |
| Paraguay | 58 (49 to 70) | 2.41 (2.01 to 2.97) |  | 90 (64 to 119) | 1.67 (1.19 to 2.19) | -1.23 (-1.79 to -0.66) |
| Peru | 521 (370 to 690) | 2.94 (2.2 to 3.64) |  | 229 (159 to 313) | 0.7 (0.49 to 0.97) | -5.14 (-5.83 to -4.44) |
| Philippines | 9191 (8097 to 10256) | 32.01 (27.71 to 36.72) |  | 11776 (9712 to 14703) | 16.36 (13.54 to 21.18) | -2.12 (-2.36 to -1.88) |
| Poland | 2957 (2751 to 3108) | 7.05 (6.54 to 7.44) |  | 524 (428 to 691) | 0.73 (0.6 to 0.96) | -7.69 (-8.15 to -7.22) |
| Portugal | 499 (443 to 553) | 3.98 (3.51 to 4.4) |  | 148 (113 to 181) | 0.54 (0.43 to 0.66) | -6.66 (-6.9 to -6.42) |
| Puerto Rico | 162 (144 to 186) | 4.6 (4.11 to 5.26) |  | 93 (70 to 120) | 1.44 (1.09 to 1.87) | -4.06 (-4.86 to -3.24) |
| Qatar | 5 (4 to 7) | 4.66 (3.06 to 8.7) |  | 8 (6 to 12) | 1.36 (1.02 to 1.94) | -4.04 (-4.57 to -3.51) |
| Republic of Korea | 6133 (3870 to 7153) | 35.6 (20.81 to 42.09) |  | 2674 (2002 to 3282) | 3.48 (2.56 to 4.31) | -7.9 (-8.29 to -7.51) |
| Republic of Moldova | 136 (105 to 164) | 3.21 (2.49 to 3.86) |  | 31 (25 to 41) | 0.53 (0.43 to 0.73) | -6.47 (-7.65 to -5.28) |
| Romania | 1098 (738 to 1390) | 4.41 (2.91 to 5.55) |  | 254 (199 to 329) | 0.65 (0.51 to 0.84) | -6.29 (-6.63 to -5.95) |
| Russian Federation | 8677 (7315 to 9434) | 4.91 (4.11 to 5.34) |  | 1786 (1499 to 2443) | 0.78 (0.66 to 1.07) | -6.17 (-7.08 to -5.26) |
| Rwanda | 1154 (765 to 1539) | 32.56 (21.52 to 46.75) |  | 760 (516 to 1217) | 12.58 (8.5 to 21.48) | -3.32 (-3.45 to -3.19) |
| Saint Kitts and Nevis | 2 (2 to 2) | 5.04 (4.28 to 5.89) |  | 1 (1 to 1) | 1.94 (1.51 to 2.43) | -3 (-3.45 to -2.54) |
| Saint Lucia | 8 (7 to 9) | 8.79 (7.65 to 10.06) |  | 8 (6 to 10) | 4.15 (3.21 to 5.14) | -2.44 (-2.87 to -2.01) |
| Saint Vincent and the Grenadines | 4 (3 to 4) | 4.71 (4.02 to 5.48) |  | 4 (3 to 5) | 3.02 (2.42 to 3.65) | -1.56 (-2.04 to -1.09) |
| Samoa | 29 (21 to 43) | 39.02 (28.38 to 57.65) |  | 25 (19 to 33) | 19.86 (15.35 to 25.5) | -2.4 (-2.56 to -2.24) |
| San Marino | 0 (0 to 0) | 0.86 (0.67 to 1.07) |  | 0 (0 to 1) | 0.58 (0.37 to 0.85) | -1.32 (-1.41 to -1.23) |
| Sao Tome and Principe | 25 (20 to 30) | 41.06 (32.35 to 49.68) |  | 25 (17 to 33) | 26.13 (17.66 to 34.16) | -1.44 (-1.61 to -1.28) |
| Saudi Arabia | 853 (603 to 1236) | 15.94 (11.16 to 23.98) |  | 906 (684 to 1225) | 6.15 (4.73 to 8.03) | -3.2 (-3.36 to -3.04) |
| Senegal | 897 (657 to 1128) | 27.49 (20.28 to 35) |  | 1010 (772 to 1316) | 14.22 (10.97 to 18.62) | -2.23 (-2.46 to -2.01) |
| Serbia | 603 (471 to 770) | 6.04 (4.66 to 7.79) |  | 331 (257 to 417) | 2.18 (1.7 to 2.7) | -3.28 (-3.58 to -2.98) |
| Seychelles | 7 (6 to 10) | 13 (10.2 to 16.77) |  | 6 (4 to 8) | 6.59 (4.46 to 8.95) | -2.38 (-2.75 to -2) |
| Sierra Leone | 533 (382 to 683) | 27.31 (19.58 to 35.23) |  | 574 (415 to 771) | 15.67 (11.56 to 20.77) | -1.9 (-1.99 to -1.81) |
| Singapore | 127 (104 to 139) | 5.62 (4.59 to 6.21) |  | 45 (37 to 67) | 0.62 (0.5 to 0.93) | -7.54 (-7.96 to -7.12) |
| Slovakia | 103 (86 to 141) | 1.78 (1.5 to 2.4) |  | 52 (39 to 71) | 0.59 (0.45 to 0.79) | -3.55 (-4 to -3.09) |
| Slovenia | 51 (40 to 66) | 2.18 (1.71 to 2.82) |  | 20 (15 to 27) | 0.42 (0.32 to 0.57) | -5.73 (-6.38 to -5.09) |
| Solomon Islands | 57 (41 to 76) | 49.12 (34.66 to 69.36) |  | 91 (65 to 119) | 36.46 (25.23 to 46.75) | -1.01 (-1.12 to -0.91) |
| Somalia | 1049 (595 to 1502) | 34.1 (18.94 to 52.93) |  | 1815 (1080 to 3081) | 24.56 (13.93 to 45.57) | -1.1 (-1.19 to -1.01) |
| South Africa | 4567 (3936 to 5452) | 20.75 (17.62 to 26.03) |  | 4669 (4215 to 5348) | 11.61 (10.37 to 13.16) | -1.85 (-2.36 to -1.33) |
| South Sudan | 602 (386 to 807) | 20.41 (12.53 to 28.53) |  | 501 (327 to 775) | 12.48 (7.82 to 20.84) | -1.64 (-1.72 to -1.57) |
| Spain | 1191 (1063 to 1324) | 2.27 (2.03 to 2.51) |  | 1122 (798 to 1444) | 0.92 (0.69 to 1.15) | -3.05 (-3.29 to -2.82) |
| Sri Lanka | 6682 (5605 to 7565) | 80.95 (67.77 to 91.19) |  | 8229 (5744 to 10723) | 40.31 (27.9 to 51.84) | -2.34 (-2.8 to -1.87) |
| Sudan | 3196 (1949 to 4946) | 34.68 (19.7 to 60.74) |  | 2484 (1552 to 3791) | 14.72 (8.97 to 23.46) | -2.91 (-2.95 to -2.87) |
| Suriname | 13 (11 to 15) | 4.68 (4.02 to 5.42) |  | 15 (12 to 18) | 2.52 (1.98 to 3.12) | -2.14 (-2.78 to -1.5) |
| Sweden | 384 (346 to 456) | 2.56 (2.33 to 3) |  | 153 (116 to 184) | 0.65 (0.51 to 0.77) | -4.65 (-4.94 to -4.37) |
| Switzerland | 281 (235 to 324) | 2.65 (2.23 to 3.05) |  | 110 (86 to 133) | 0.54 (0.43 to 0.64) | -5.6 (-5.89 to -5.31) |
| Syrian Arab Republic | 1168 (909 to 1480) | 22.36 (16.66 to 29.66) |  | 1172 (874 to 1539) | 12.38 (9.29 to 15.95) | -2.14 (-2.44 to -1.84) |
| Taiwan (Province of China) | 1785 (1562 to 1928) | 14.5 (12.57 to 15.84) |  | 825 (619 to 1104) | 2.06 (1.54 to 2.76) | -6.52 (-6.95 to -6.08) |
| Tajikistan | 380 (254 to 554) | 14.15 (9.38 to 20.87) |  | 250 (191 to 327) | 9.39 (6.74 to 12.16) | -1.48 (-1.64 to -1.32) |
| Thailand | 4631 (3346 to 5834) | 15.02 (10.5 to 19.31) |  | 4194 (3053 to 5582) | 4.41 (3.21 to 5.83) | -4.14 (-4.63 to -3.65) |
| Timor-Leste | 120 (81 to 160) | 36.83 (25.08 to 53.61) |  | 145 (98 to 203) | 19.75 (13.47 to 27.95) | -2.17 (-2.29 to -2.04) |
| Togo | 358 (275 to 453) | 28.28 (21.29 to 35.72) |  | 517 (373 to 703) | 15.31 (11.26 to 20.82) | -2.1 (-2.25 to -1.96) |
| Tokelau | 0 (0 to 1) | 35.61 (26.94 to 47.94) |  | 0 (0 to 0) | 14.23 (10.29 to 18.75) | -3.13 (-3.17 to -3.09) |
| Tonga | 9 (7 to 12) | 19.84 (15.41 to 26.82) |  | 9 (7 to 12) | 11.9 (8.93 to 15.17) | -1.6 (-2.1 to -1.1) |
| Trinidad and Tobago | 74 (66 to 81) | 8.64 (7.79 to 9.6) |  | 55 (40 to 75) | 3.25 (2.35 to 4.4) | -3.29 (-3.76 to -2.81) |
| Tunisia | 612 (475 to 839) | 14.25 (10.84 to 19.59) |  | 687 (485 to 917) | 6.11 (4.32 to 8.18) | -2.9 (-2.99 to -2.82) |
| Turkey | 3309 (2565 to 4596) | 9.83 (7.37 to 14.24) |  | 2666 (1982 to 3588) | 3.32 (2.46 to 4.5) | -3.57 (-4.18 to -2.96) |
| Turkmenistan | 206 (146 to 260) | 10.64 (7.56 to 13.73) |  | 49 (33 to 93) | 1.31 (0.87 to 2.55) | -7.82 (-8.95 to -6.68) |
| Tuvalu | 3 (2 to 4) | 52.16 (36.08 to 76.39) |  | 2 (1 to 2) | 21.37 (15.23 to 28.49) | -3 (-3.09 to -2.91) |
| Uganda | 1629 (976 to 2377) | 21.29 (12.82 to 33.99) |  | 1802 (1236 to 2674) | 11.68 (8.03 to 18.76) | -2.04 (-2.19 to -1.9) |
| Ukraine | 1271 (715 to 1658) | 1.97 (1.09 to 2.53) |  | 280 (211 to 521) | 0.41 (0.31 to 0.74) | -5.19 (-6.06 to -4.32) |
| United Arab Emirates | 155 (96 to 251) | 33.65 (22.32 to 52.87) |  | 550 (313 to 1012) | 12.24 (7.58 to 21.41) | -3.49 (-3.88 to -3.09) |
| United Kingdom | 2171 (2037 to 2593) | 2.67 (2.51 to 3.16) |  | 1524 (1284 to 1714) | 1.22 (1.06 to 1.36) | -2.68 (-2.95 to -2.41) |
| United Republic of Tanzania | 1663 (1269 to 2095) | 13.42 (9.94 to 17.93) |  | 2316 (1684 to 3132) | 8.61 (6.11 to 12.3) | -1.52 (-1.65 to -1.39) |
| United States of America | 5012 (4619 to 5804) | 1.66 (1.54 to 1.9) |  | 4072 (3353 to 4367) | 0.87 (0.74 to 0.92) | -2.15 (-2.27 to -2.03) |
| United States Virgin Islands | 4 (3 to 4) | 4.44 (3.67 to 5.31) |  | 4 (3 to 5) | 2.54 (1.99 to 3.11) | -1.98 (-2.14 to -1.82) |
| Uruguay | 226 (194 to 265) | 5.97 (5.14 to 6.99) |  | 123 (101 to 144) | 2.2 (1.83 to 2.55) | -3.47 (-4.04 to -2.89) |
| Uzbekistan | 1364 (925 to 1716) | 12.57 (8.27 to 16.04) |  | 1123 (886 to 1641) | 7.22 (5.73 to 11.59) | -1.99 (-2.48 to -1.49) |
| Vanuatu | 27 (17 to 42) | 47.53 (30.15 to 79.43) |  | 45 (31 to 66) | 31.55 (21.84 to 47.19) | -1.42 (-1.55 to -1.29) |
| Venezuela (Bolivarian Republic of) | 392 (334 to 443) | 3.27 (2.81 to 3.77) |  | 415 (305 to 554) | 1.47 (1.08 to 1.96) | -2.77 (-3.2 to -2.34) |
| Viet Nam | 7610 (2722 to 10854) | 20.56 (7.09 to 30.2) |  | 6834 (2814 to 9200) | 8.68 (3.55 to 11.87) | -2.92 (-3 to -2.83) |
| Yemen | 1739 (1115 to 2638) | 38.29 (23.34 to 64.6) |  | 2125 (1547 to 3039) | 18.04 (13.12 to 26.32) | -2.6 (-2.69 to -2.51) |
| Zambia | 694 (522 to 876) | 19.5 (14.74 to 25.51) |  | 780 (595 to 1011) | 10.87 (8.12 to 14.18) | -2.04 (-2.12 to -1.96) |
| Zimbabwe | 959 (795 to 1139) | 24.97 (20.57 to 30.86) |  | 1565 (1054 to 2159) | 23.65 (15.65 to 33.15) | -0.21 (-0.34 to -0.07) |

AAPC: average annual percent change; UI: uncertainty interval; CI: confidence interval; SDI: Socio-demographic index.

Table S11. Mortality of ILD&PS (1990/2019) for sexes, SDI and locations, with AAPC (1990-2019)

| GBD data | 1990 | |  | 2019 | | AAPC % (95% CI)  1990-2019 |
| --- | --- | --- | --- | --- | --- | --- |
|  | Cases (95% UI) | Age-standardised mortality per  100 000 population (95% UI) |  | Cases (95% UI) | Age-standardised mortality per  100 000 population (95% UI) |  |
| Global | 63697 (50958 to 80625) | 1.76 (1.41 to 2.22) |  | 169833 (118756 to 204802) | 2.17 (1.5 to 2.62) | 0.73 (0.61 to 0.84) |
| **Sex** |  |  |  |  |  |  |
| Female | 27210 (20775 to 38024) | 1.34 (1.02 to 1.87) |  | 76869 (48378 to 94366) | 1.76 (1.11 to 2.16) | 1 (0.93 to 1.08) |
| Male | 36487 (24519 to 50833) | 2.34 (1.6 to 3.18) |  | 92964 (57581 to 120738) | 2.72 (1.66 to 3.55) | 0.53 (0.43 to 0.64) |
| **SDI** |  |  |  |  |  |  |
| High SDI | 19866 (17059 to 27310) | 1.88 (1.61 to 2.57) |  | 56666 (30161 to 74336) | 2.72 (1.52 to 3.5) | 1.35 (1.19 to 1.51) |
| High-middle SDI | 9818 (8474 to 12954) | 1 (0.85 to 1.33) |  | 22488 (16187 to 26416) | 1.14 (0.81 to 1.34) | 0.49 (0.39 to 0.59) |
| Middle SDI | 10593 (8240 to 14597) | 1.25 (0.98 to 1.73) |  | 31111 (24950 to 38137) | 1.44 (1.14 to 1.74) | 0.49 (0.4 to 0.57) |
| Low-middle SDI | 17693 (11188 to 27613) | 3.46 (2.21 to 5.27) |  | 45510 (30801 to 62865) | 3.75 (2.51 to 5.15) | 0.29 (0.16 to 0.42) |
| Low SDI | 5704 (3301 to 9191) | 2.86 (1.65 to 4.53) |  | 13999 (8922 to 19095) | 3.24 (2.04 to 4.42) | 0.53 (0.28 to 0.78) |
| **Region** |  |  |  |  |  |  |
| High-income Asia Pacific | 3986 (2730 to 4652) | 2.12 (1.45 to 2.49) |  | 15701 (7049 to 22438) | 2.87 (1.39 to 3.97) | 1.02 (0.87 to 1.18) |
| High-income North America | 9064 (7425 to 13653) | 2.49 (2.06 to 3.74) |  | 23714 (13349 to 29541) | 3.59 (2.08 to 4.43) | 1.33 (1.23 to 1.43) |
| Western Europe | 7784 (6279 to 12165) | 1.3 (1.06 to 2.01) |  | 20520 (10757 to 27206) | 2 (1.08 to 2.6) | 1.64 (1.42 to 1.87) |
| Australasia | 294 (213 to 487) | 1.25 (0.9 to 2.07) |  | 1334 (589 to 1961) | 2.46 (1.1 to 3.58) | 2.6 (2.27 to 2.93) |
| Andean Latin America | 1346 (1017 to 2118) | 7.4 (5.58 to 11.88) |  | 5167 (3564 to 6605) | 9.73 (6.72 to 12.47) | 1.08 (0.85 to 1.31) |
| Tropical Latin America | 863 (743 to 1190) | 1.02 (0.86 to 1.45) |  | 3311 (1950 to 4166) | 1.44 (0.84 to 1.81) | 1.28 (1.13 to 1.43) |
| Central Latin America | 1151 (985 to 1571) | 1.46 (1.23 to 2) |  | 4974 (2948 to 6632) | 2.18 (1.29 to 2.91) | 1.46 (1.37 to 1.55) |
| Southern Latin America | 1252 (975 to 1912) | 2.87 (2.22 to 4.43) |  | 3610 (2460 to 4328) | 4.22 (2.88 to 5.05) | 1.46 (1.35 to 1.57) |
| Caribbean | 199 (165 to 252) | 0.77 (0.64 to 0.98) |  | 555 (372 to 738) | 1.08 (0.73 to 1.44) | 1.21 (1.08 to 1.33) |
| Central Europe | 1555 (1327 to 1957) | 1.1 (0.93 to 1.37) |  | 1700 (1364 to 2112) | 0.8 (0.64 to 0.99) | -1.11 (-1.26 to -0.96) |
| Eastern Europe | 1914 (1232 to 2372) | 0.73 (0.46 to 0.89) |  | 1110 (866 to 1639) | 0.33 (0.26 to 0.49) | -2.66 (-2.95 to -2.37) |
| Central Asia | 822 (646 to 1019) | 1.91 (1.5 to 2.4) |  | 932 (749 to 1152) | 1.5 (1.18 to 1.86) | -0.73 (-0.94 to -0.52) |
| North Africa and Middle East | 1739 (1079 to 3119) | 1.18 (0.72 to 2.18) |  | 4728 (3427 to 7703) | 1.24 (0.88 to 2.04) | 0.16 (-0.04 to 0.36) |
| South Asia | 23962 (14452 to 37801) | 5.27 (3.22 to 8.18) |  | 65459 (43015 to 90348) | 5.35 (3.48 to 7.33) | 0.09 (-0.16 to 0.35) |
| Southeast Asia | 1673 (981 to 3232) | 0.76 (0.44 to 1.49) |  | 3978 (2234 to 7834) | 0.74 (0.41 to 1.48) | -0.14 (-0.22 to -0.06) |
| East Asia | 3520 (2681 to 5765) | 0.47 (0.36 to 0.76) |  | 8627 (6157 to 10749) | 0.46 (0.32 to 0.58) | -0.02 (-0.16 to 0.13) |
| Oceania | 96 (67 to 143) | 2.6 (1.81 to 3.8) |  | 223 (154 to 330) | 2.74 (1.87 to 3.88) | 0.15 (0.08 to 0.22) |
| Western Sub-Saharan Africa | 839 (356 to 1365) | 1.07 (0.45 to 1.74) |  | 1112 (719 to 1511) | 0.66 (0.42 to 0.9) | -1.68 (-1.95 to -1.41) |
| Eastern Sub-Saharan Africa | 731 (252 to 1222) | 1.09 (0.37 to 1.81) |  | 1447 (606 to 2329) | 0.99 (0.4 to 1.63) | -0.36 (-0.43 to -0.3) |
| Central Sub-Saharan Africa | 306 (102 to 562) | 1.7 (0.54 to 3.16) |  | 654 (245 to 1263) | 1.53 (0.55 to 3.15) | -0.37 (-0.49 to -0.25) |
| Southern Sub-Saharan Africa | 602 (373 to 805) | 2.48 (1.51 to 3.36) |  | 977 (783 to 1299) | 2.01 (1.6 to 2.66) | -0.71 (-0.88 to -0.53) |
| **Countries** |  |  |  |  |  |  |
| Afghanistan | 2185 (1821 to 2633) | 26.53 (21.98 to 31.8) |  | 6938 (5705 to 8350) | 32.85 (27.37 to 38.97) | 0.85 (0.71 to 0.99) |
| Albania | 1189 (1004 to 1420) | 47.44 (40.34 to 55.66) |  | 1957 (1643 to 2345) | 54.15 (45.31 to 64.94) | 0.53 (0.45 to 0.61) |
| Algeria | 4807 (3997 to 5750) | 28.98 (24.07 to 34.35) |  | 14675 (12162 to 17646) | 37.87 (31.73 to 44.85) | 0.98 (0.9 to 1.07) |
| American Samoa | 16 (14 to 19) | 43.41 (37.11 to 50.57) |  | 24 (20 to 28) | 45.24 (38.45 to 52.45) | 0.18 (0.11 to 0.25) |
| Andorra | 25 (22 to 29) | 43.39 (37.37 to 49.96) |  | 70 (59 to 82) | 51.37 (43.64 to 59.6) | 0.59 (0.53 to 0.64) |
| Angola | 1379 (1154 to 1642) | 22.59 (19.15 to 26.3) |  | 4743 (3983 to 5662) | 27.63 (23.52 to 32.32) | 0.71 (0.65 to 0.77) |
| Antigua and Barbuda | 9 (7 to 11) | 16.98 (13.76 to 20.36) |  | 22 (19 to 27) | 21.55 (18.19 to 25.49) | 0.85 (0.77 to 0.94) |
| Argentina | 17502 (15351 to 19801) | 53.64 (47.07 to 60.63) |  | 43007 (38338 to 47633) | 80.74 (71.97 to 89.02) | 1.47 (1.4 to 1.54) |
| Armenia | 1575 (1384 to 1791) | 55.28 (48.76 to 62.01) |  | 2117 (1850 to 2399) | 53.93 (47 to 60.89) | -0.05 (-0.19 to 0.09) |
| Australia | 8070 (7035 to 9178) | 41.57 (36.24 to 47.34) |  | 26346 (22881 to 29785) | 65.23 (56.86 to 73.11) | 1.57 (1.49 to 1.65) |
| Austria | 5477 (4727 to 6318) | 53.94 (46.26 to 62.96) |  | 7744 (6662 to 8958) | 52.14 (44.62 to 60.47) | 0.11 (-0.11 to 0.32) |
| Azerbaijan | 1665 (1406 to 1976) | 29 (24.74 to 34) |  | 3350 (2760 to 4055) | 31.34 (26.24 to 36.94) | 0.34 (0.22 to 0.46) |
| Bahamas | 50 (43 to 57) | 28.43 (24.8 to 32.49) |  | 138 (121 to 158) | 34.08 (30.08 to 38.43) | 0.64 (0.55 to 0.74) |
| Bahrain | 119 (99 to 145) | 41.24 (35.82 to 47.46) |  | 760 (637 to 912) | 62.4 (55.07 to 70.39) | 1.45 (1.33 to 1.57) |
| Bangladesh | 25813 (22848 to 28907) | 47.54 (42.23 to 53.11) |  | 87502 (77765 to 97842) | 63.53 (56.57 to 70.82) | 0.98 (0.91 to 1.04) |
| Barbados | 60 (52 to 70) | 22.37 (19.19 to 26.31) |  | 126 (110 to 144) | 28.19 (24.6 to 32.32) | 0.8 (0.75 to 0.85) |
| Belarus | 5639 (4747 to 6712) | 46.37 (38.97 to 55.27) |  | 5755 (4589 to 7313) | 44.68 (35.68 to 56.21) | -0.03 (-0.14 to 0.09) |
| Belgium | 5106 (4439 to 5861) | 35.82 (30.9 to 41.2) |  | 9846 (8575 to 11216) | 48.64 (42 to 55.75) | 1.36 (1.06 to 1.67) |
| Belize | 45 (40 to 50) | 42.76 (37.91 to 47.84) |  | 213 (190 to 237) | 68.62 (61.51 to 75.87) | 1.63 (1.43 to 1.83) |
| Benin | 641 (541 to 760) | 21.9 (18.46 to 25.86) |  | 1756 (1451 to 2136) | 21.87 (18.15 to 26.39) | 0.03 (-0.03 to 0.09) |
| Bermuda | 30 (26 to 33) | 47.16 (41.49 to 52.81) |  | 77 (68 to 85) | 63.25 (56.14 to 70.56) | 0.92 (0.8 to 1.04) |
| Bhutan | 121 (107 to 136) | 40.7 (36.49 to 45.35) |  | 379 (336 to 426) | 63.22 (56.29 to 70.4) | 1.54 (1.47 to 1.61) |
| Bolivia (Plurinational State of) | 2147 (1881 to 2413) | 64.6 (56.56 to 72.41) |  | 7870 (7009 to 8716) | 90.28 (80.86 to 99.58) | 1.14 (1.05 to 1.24) |
| Bosnia and Herzegovina | 1964 (1627 to 2366) | 43.66 (36.75 to 51.89) |  | 2353 (1953 to 2837) | 49.37 (40.73 to 59.89) | 0.36 (0.09 to 0.64) |
| Botswana | 319 (274 to 369) | 42 (36.54 to 48.22) |  | 830 (703 to 981) | 47.09 (40.74 to 53.89) | 0.45 (0.32 to 0.59) |
| Brazil | 33167 (27311 to 39699) | 31.49 (26.05 to 37.61) |  | 48743 (40574 to 57598) | 20.67 (17.23 to 24.37) | -1.36 (-1.57 to -1.14) |
| Brunei Darussalam | 182 (159 to 208) | 159.96 (140.43 to 179.95) |  | 443 (382 to 501) | 135.43 (118.72 to 151.33) | -0.57 (-0.62 to -0.53) |
| Bulgaria | 4535 (3736 to 5479) | 40.94 (33.84 to 49.61) |  | 4725 (3821 to 5817) | 46.64 (37.65 to 58.27) | 0.53 (0.41 to 0.66) |
| Burkina Faso | 1184 (978 to 1425) | 18.42 (15.15 to 22.28) |  | 3108 (2527 to 3813) | 20.51 (16.61 to 25.06) | 0.41 (0.32 to 0.51) |
| Burundi | 708 (597 to 840) | 21.25 (17.96 to 24.95) |  | 1569 (1313 to 1894) | 21.94 (18.45 to 26.06) | 0.15 (0.1 to 0.2) |
| Cabo Verde | 49 (41 to 58) | 19.84 (16.48 to 24.06) |  | 112 (91 to 139) | 21.95 (17.89 to 26.89) | 0.42 (0.32 to 0.52) |
| Cambodia | 571 (466 to 692) | 9.9 (8.08 to 12) |  | 1856 (1526 to 2237) | 14.35 (11.86 to 17.23) | 1.29 (1.19 to 1.4) |
| Cameroon | 1630 (1400 to 1910) | 25.77 (22.14 to 29.96) |  | 4444 (3710 to 5328) | 23.36 (19.63 to 27.85) | -0.32 (-0.38 to -0.27) |
| Canada | 28019 (24473 to 31722) | 88.05 (76.78 to 99.68) |  | 73315 (64337 to 81998) | 116.47 (102.28 to 130.92) | 0.98 (0.9 to 1.06) |
| Central African Republic | 398 (338 to 471) | 23.91 (20.56 to 28.05) |  | 801 (667 to 955) | 24.2 (20.63 to 28.27) | 0.07 (0 to 0.14) |
| Chad | 824 (695 to 974) | 21.38 (18.01 to 25.22) |  | 2069 (1713 to 2508) | 21.55 (17.88 to 26.06) | 0.06 (-0.02 to 0.15) |
| Chile | 8394 (7525 to 9310) | 82.09 (73.83 to 91.04) |  | 33978 (30069 to 37950) | 141.67 (125.61 to 158.11) | 1.8 (1.68 to 1.92) |
| China | 282517 (226776 to 346000) | 30.06 (24.12 to 36.53) |  | 722410 (586951 to 872704) | 35.62 (29.2 to 42.12) | 0.74 (0.39 to 1.09) |
| Colombia | 5606 (4564 to 6807) | 26.08 (21.23 to 31.76) |  | 17509 (14949 to 20677) | 33.65 (28.8 to 39.42) | 0.92 (0.79 to 1.06) |
| Comoros | 63 (53 to 73) | 21.25 (18.03 to 24.97) |  | 132 (110 to 159) | 22.51 (18.98 to 26.79) | 0.25 (0.17 to 0.33) |
| Congo | 371 (315 to 433) | 25.24 (21.68 to 29.3) |  | 1028 (862 to 1225) | 28.64 (24.58 to 33.3) | 0.49 (0.42 to 0.55) |
| Cook Islands | 8 (6 to 9) | 47.42 (40.67 to 54.67) |  | 12 (10 to 14) | 57.06 (49.24 to 65.57) | 0.69 (0.62 to 0.75) |
| Costa Rica | 998 (888 to 1112) | 53.41 (47.53 to 59.61) |  | 3581 (3196 to 3984) | 70.25 (62.84 to 78.1) | 0.97 (0.9 to 1.04) |
| Croatia | 2842 (2291 to 3510) | 47.45 (38.55 to 58.89) |  | 3764 (3079 to 4504) | 60.68 (50.01 to 72.62) | 1.09 (0.88 to 1.3) |
| Cuba | 1705 (1367 to 2090) | 16.34 (12.98 to 20.13) |  | 3027 (2442 to 3779) | 18.44 (15.03 to 22.57) | 0.48 (0.39 to 0.57) |
| Cyprus | 481 (414 to 552) | 56.36 (49.03 to 63.89) |  | 1314 (1130 to 1500) | 67.02 (58.01 to 76.25) | 0.44 (0.13 to 0.75) |
| Czechia | 4679 (3879 to 5616) | 37.67 (31.23 to 45.42) |  | 10963 (9403 to 12748) | 63.48 (54.29 to 74.8) | 2.06 (1.82 to 2.29) |
| Côte d’Ivoire | 1558 (1294 to 1881) | 21.52 (18.13 to 25.63) |  | 3850 (3144 to 4737) | 20.94 (17.28 to 25.43) | -0.03 (-0.13 to 0.07) |
| Democratic People's Republic of Korea | 4026 (3377 to 4763) | 24.46 (20.81 to 28.49) |  | 7616 (6366 to 8892) | 23.6 (19.86 to 27.38) | -0.19 (-0.29 to -0.09) |
| Democratic Republic of the Congo | 5665 (4796 to 6707) | 25.17 (21.41 to 29.42) |  | 14276 (11961 to 16977) | 27.51 (23.37 to 31.96) | 0.35 (0.29 to 0.42) |
| Denmark | 2939 (2577 to 3341) | 38.9 (34.05 to 44.39) |  | 4911 (4328 to 5487) | 45.97 (40.4 to 51.78) | 0.61 (0.37 to 0.85) |
| Djibouti | 57 (48 to 69) | 22.51 (19.23 to 26.24) |  | 212 (175 to 260) | 24.23 (20.46 to 28.59) | 0.31 (0.22 to 0.4) |
| Dominica | 13 (11 to 15) | 19.74 (16.8 to 23.25) |  | 21 (18 to 25) | 25.16 (21.77 to 29.06) | 0.86 (0.83 to 0.9) |
| Dominican Republic | 717 (581 to 873) | 14.58 (11.72 to 17.96) |  | 1829 (1509 to 2203) | 18.18 (15.03 to 21.8) | 0.82 (0.74 to 0.9) |
| Ecuador | 2263 (1967 to 2568) | 39.36 (34.13 to 44.56) |  | 12893 (11533 to 14252) | 86.51 (77.18 to 95.71) | 3.06 (2.76 to 3.36) |
| Egypt | 11562 (9613 to 13747) | 29.74 (24.87 to 35.36) |  | 34492 (29065 to 40876) | 44.75 (37.72 to 52.51) | 1.42 (1.34 to 1.5) |
| El Salvador | 999 (866 to 1156) | 29.79 (25.87 to 34.34) |  | 2776 (2437 to 3132) | 46.73 (40.97 to 52.69) | 1.6 (1.46 to 1.74) |
| Equatorial Guinea | 56 (47 to 66) | 20.32 (17.33 to 23.86) |  | 259 (219 to 304) | 36.53 (31.35 to 42.34) | 2.13 (1.98 to 2.28) |
| Eritrea | 342 (284 to 408) | 20.09 (17.01 to 23.76) |  | 972 (810 to 1173) | 23.16 (19.73 to 27.32) | 0.56 (0.46 to 0.65) |
| Estonia | 838 (699 to 992) | 45.01 (37.5 to 53.43) |  | 916 (766 to 1093) | 49.42 (40.98 to 60.29) | 0.38 (0.29 to 0.47) |
| Eswatini | 169 (144 to 198) | 39.68 (34.35 to 45.59) |  | 318 (269 to 374) | 40.72 (34.91 to 46.94) | 0.08 (-0.04 to 0.2) |
| Ethiopia | 5879 (4846 to 7117) | 17.53 (14.55 to 21.15) |  | 13859 (11415 to 16850) | 19.74 (16.29 to 23.65) | 0.48 (0.31 to 0.64) |
| Fiji | 221 (182 to 267) | 35.88 (29.99 to 43.18) |  | 370 (306 to 443) | 41.09 (34.21 to 48.73) | 0.55 (0.39 to 0.72) |
| Finland | 3069 (2660 to 3498) | 44.95 (38.62 to 51.58) |  | 6588 (5715 to 7473) | 59.51 (51.47 to 68.05) | 1 (0.88 to 1.12) |
| France | 27422 (23609 to 31459) | 35.68 (30.56 to 41.46) |  | 51135 (44653 to 58206) | 42.78 (36.97 to 49.18) | 0.64 (0.62 to 0.66) |
| Gabon | 177 (151 to 206) | 26.15 (22.48 to 30.32) |  | 377 (317 to 449) | 28.69 (24.47 to 33.54) | 0.35 (0.31 to 0.39) |
| Gambia | 128 (106 to 153) | 21.84 (18.25 to 25.84) |  | 321 (265 to 390) | 21.33 (17.62 to 25.9) | -0.05 (-0.12 to 0.03) |
| Georgia | 1842 (1549 to 2176) | 30.33 (25.54 to 35.57) |  | 1426 (1263 to 1611) | 27.45 (24.33 to 30.94) | -0.41 (-0.65 to -0.18) |
| Germany | 44911 (39190 to 51593) | 37.98 (33.04 to 43.69) |  | 78111 (68261 to 88130) | 45.95 (40.09 to 52.39) | 1.11 (0.55 to 1.66) |
| Ghana | 2000 (1665 to 2416) | 20.18 (16.77 to 24.31) |  | 5284 (4338 to 6445) | 22.21 (18.49 to 27.03) | 0.36 (0.29 to 0.42) |
| Greece | 3324 (2723 to 4019) | 23.21 (19.02 to 28.04) |  | 8090 (6937 to 9285) | 38.42 (32.9 to 44.42) | 1.84 (1.73 to 1.94) |
| Greenland | 41 (35 to 46) | 110.98 (97.08 to 123.91) |  | 84 (73 to 95) | 125.53 (108.98 to 141.1) | 0.43 (0.41 to 0.45) |
| Grenada | 14 (12 to 16) | 20.39 (17.37 to 23.61) |  | 33 (28 to 38) | 28.96 (25.35 to 32.95) | 1.2 (1.15 to 1.25) |
| Guam | 120 (107 to 133) | 113.03 (101.15 to 124.15) |  | 196 (175 to 216) | 108.32 (97.21 to 119.21) | -0.2 (-0.26 to -0.14) |
| Guatemala | 1593 (1403 to 1804) | 36.63 (32.61 to 41.12) |  | 5271 (4657 to 5940) | 44.48 (39.32 to 50.12) | 0.69 (0.59 to 0.79) |
| Guinea | 899 (758 to 1067) | 20.83 (17.6 to 24.68) |  | 1774 (1461 to 2154) | 21.33 (17.65 to 25.74) | 0.13 (0.05 to 0.2) |
| Guinea-Bissau | 132 (111 to 158) | 21.12 (17.78 to 25.1) |  | 264 (217 to 322) | 20.79 (17.23 to 25.24) | 0 (-0.08 to 0.07) |
| Guyana | 93 (79 to 109) | 19.21 (16.44 to 22.38) |  | 174 (149 to 202) | 25.34 (22 to 29.12) | 0.97 (0.93 to 1) |
| Haiti | 714 (601 to 837) | 17.43 (14.81 to 20.25) |  | 1909 (1638 to 2226) | 22.46 (19.53 to 25.69) | 0.9 (0.85 to 0.94) |
| Honduras | 921 (810 to 1041) | 37.26 (33.04 to 42.04) |  | 3099 (2712 to 3506) | 46.8 (40.86 to 52.7) | 0.79 (0.74 to 0.84) |
| Hungary | 6129 (5208 to 7213) | 46.37 (39.28 to 54.61) |  | 8806 (7612 to 10231) | 58.3 (49.92 to 68.81) | 0.78 (0.66 to 0.9) |
| Iceland | 106 (90 to 122) | 38.69 (32.78 to 45.44) |  | 266 (230 to 302) | 51.96 (44.65 to 59.32) | 1.31 (1.04 to 1.58) |
| India | 388385 (323103 to 458851) | 77.23 (63.56 to 91.41) |  | 881289 (731880 to 1042779) | 73.6 (60.95 to 87.18) | -0.15 (-0.21 to -0.1) |
| Indonesia | 16558 (13732 to 19801) | 14.21 (11.61 to 17.01) |  | 38799 (31602 to 46626) | 16.3 (13.29 to 19.55) | 0.49 (0.47 to 0.5) |
| Iran (Islamic Republic of) | 10892 (8945 to 13142) | 27.75 (22.71 to 33.77) |  | 28589 (23241 to 35098) | 33.17 (27.29 to 39.91) | 0.7 (0.47 to 0.92) |
| Iraq | 3067 (2560 to 3661) | 27.87 (23.23 to 33.24) |  | 11280 (9346 to 13511) | 36.41 (30.59 to 43.11) | 1.01 (0.9 to 1.12) |
| Ireland | 2055 (1812 to 2301) | 50.1 (44.19 to 56.18) |  | 6028 (5225 to 6837) | 81.5 (70.88 to 92.18) | 1.62 (1.51 to 1.73) |
| Israel | 1477 (1280 to 1696) | 30.73 (26.48 to 35.5) |  | 3869 (3379 to 4415) | 35.22 (30.57 to 40.6) | 0.5 (0.46 to 0.55) |
| Italy | 41914 (33587 to 50263) | 53.65 (42.89 to 64.57) |  | 79985 (66255 to 94155) | 66.94 (56.15 to 77.95) | 0.87 (0.41 to 1.33) |
| Jamaica | 315 (265 to 370) | 17.19 (14.28 to 20.46) |  | 616 (516 to 728) | 20.87 (17.55 to 24.57) | 0.67 (0.61 to 0.72) |
| Japan | 257742 (208631 to 311593) | 153.45 (125.33 to 183.66) |  | 511281 (419252 to 614079) | 162.61 (135.95 to 190.47) | 0.27 (0.17 to 0.36) |
| Jordan | 1016 (891 to 1141) | 58.86 (52.13 to 65.66) |  | 5452 (4780 to 6218) | 71.45 (63.39 to 80.01) | 0.67 (0.62 to 0.72) |
| Kazakhstan | 3799 (3194 to 4504) | 26.6 (22.5 to 31.22) |  | 5830 (4825 to 6992) | 30.98 (25.93 to 36.83) | 0.56 (0.4 to 0.72) |
| Kenya | 3185 (2665 to 3779) | 25.06 (21.06 to 29.66) |  | 8284 (6893 to 9915) | 24.97 (20.92 to 29.61) | 0.05 (-0.01 to 0.1) |
| Kiribati | 24 (20 to 28) | 40.52 (34.98 to 46.22) |  | 44 (38 to 52) | 43.06 (37.14 to 50) | 0.27 (0.19 to 0.35) |
| Kuwait | 448 (376 to 535) | 47.53 (41.67 to 53.38) |  | 2054 (1723 to 2466) | 60.99 (53.97 to 68.45) | 0.85 (0.72 to 0.99) |
| Kyrgyzstan | 883 (742 to 1064) | 25.81 (21.56 to 31) |  | 1569 (1242 to 1970) | 27.38 (21.81 to 33.92) | 0.33 (0.17 to 0.5) |
| Lao People's Democratic Republic | 274 (227 to 325) | 11 (9.14 to 13.03) |  | 831 (689 to 992) | 17.08 (14.14 to 20.27) | 1.56 (1.47 to 1.65) |
| Latvia | 1972 (1695 to 2305) | 60.49 (51.63 to 70.52) |  | 1407 (1163 to 1704) | 52.82 (43.08 to 64.99) | -0.35 (-0.97 to 0.28) |
| Lebanon | 794 (664 to 953) | 30.48 (25.46 to 36.26) |  | 2258 (1889 to 2644) | 42.79 (35.96 to 50.11) | 1.21 (1.15 to 1.26) |
| Lesotho | 444 (382 to 514) | 36.51 (31.55 to 42.27) |  | 615 (522 to 735) | 38.21 (32.93 to 44.7) | 0.22 (0.14 to 0.3) |
| Liberia | 274 (227 to 333) | 18.98 (15.6 to 23.16) |  | 717 (578 to 894) | 20.4 (16.6 to 25.3) | 0.32 (0.18 to 0.47) |
| Libya | 817 (686 to 967) | 32.2 (27.13 to 37.84) |  | 2400 (1987 to 2910) | 38.45 (32.38 to 45.37) | 0.63 (0.53 to 0.72) |
| Lithuania | 1459 (1183 to 1789) | 35.05 (28.53 to 43.13) |  | 1491 (1207 to 1817) | 38.23 (30.84 to 47.32) | 0.52 (0.27 to 0.77) |
| Luxembourg | 169 (145 to 197) | 32.28 (27.56 to 37.8) |  | 407 (351 to 467) | 43.15 (37.25 to 49.5) | 1.07 (0.92 to 1.23) |
| Madagascar | 1689 (1444 to 1961) | 24.02 (20.56 to 27.79) |  | 4139 (3492 to 4954) | 25.63 (22 to 29.86) | 0.29 (0.19 to 0.39) |
| Malawi | 1146 (956 to 1382) | 19.62 (16.46 to 23.72) |  | 2489 (2068 to 3008) | 22.42 (18.73 to 26.5) | 0.49 (0.43 to 0.56) |
| Malaysia | 1697 (1450 to 1977) | 16.48 (14.1 to 19) |  | 6657 (5714 to 7647) | 23.91 (20.71 to 27.26) | 1.26 (1.2 to 1.32) |
| Maldives | 89 (78 to 101) | 89.64 (78.3 to 101.63) |  | 382 (340 to 426) | 122.45 (108.29 to 136.58) | 0.96 (0.75 to 1.17) |
| Mali | 1284 (1090 to 1510) | 22.46 (19.07 to 26.38) |  | 3217 (2703 to 3822) | 24.54 (20.74 to 28.94) | 0.33 (0.26 to 0.4) |
| Malta | 222 (196 to 249) | 51.38 (45.33 to 57.5) |  | 677 (592 to 766) | 75.89 (67.04 to 85.23) | 1.41 (1.33 to 1.5) |
| Marshall Islands | 17 (14 to 20) | 54.22 (47.89 to 61.05) |  | 29 (25 to 33) | 57.84 (50.89 to 65.29) | 0.21 (0.17 to 0.25) |
| Mauritania | 306 (258 to 362) | 22.41 (18.9 to 26.43) |  | 644 (532 to 781) | 22.36 (18.53 to 27.11) | 0.03 (-0.04 to 0.09) |
| Mauritius | 333 (289 to 376) | 44.65 (38.93 to 50.47) |  | 1548 (1357 to 1750) | 87.75 (77.12 to 98.76) | 2.27 (2.17 to 2.38) |
| Mexico | 27677 (23077 to 32587) | 57.6 (47.34 to 68.65) |  | 67392 (56512 to 79179) | 57.26 (47.86 to 67.55) | -0.07 (-0.14 to 0.01) |
| Micronesia (Federated States of) | 40 (34 to 46) | 51.91 (45.64 to 58.86) |  | 53 (46 to 60) | 57.69 (50.63 to 64.87) | 0.31 (0.18 to 0.44) |
| Monaco | 29 (26 to 33) | 46.79 (40.69 to 53.33) |  | 42 (36 to 47) | 49.8 (43.34 to 56.8) | 0.21 (0.19 to 0.23) |
| Mongolia | 463 (403 to 529) | 33.89 (29.71 to 38.26) |  | 1003 (841 to 1196) | 34.02 (29.17 to 39.55) | 0.03 (0 to 0.06) |
| Montenegro | 265 (212 to 331) | 40.84 (32.86 to 50.46) |  | 388 (311 to 489) | 47.6 (38.05 to 59.76) | 0.61 (0.44 to 0.78) |
| Morocco | 4870 (4045 to 5911) | 26.65 (22.06 to 32.2) |  | 12459 (10290 to 14961) | 35.78 (30.03 to 42.49) | 1.09 (0.99 to 1.18) |
| Mozambique | 1588 (1313 to 1926) | 18.11 (15.02 to 21.83) |  | 3784 (3153 to 4565) | 21.96 (18.41 to 26.36) | 0.7 (0.57 to 0.84) |
| Myanmar | 4214 (3500 to 4937) | 16.47 (13.61 to 19.33) |  | 14850 (12190 to 17566) | 31.21 (25.73 to 36.72) | 2.23 (2.13 to 2.33) |
| Namibia | 354 (305 to 411) | 39.15 (33.82 to 45.18) |  | 750 (633 to 881) | 42.96 (36.79 to 49.74) | 0.42 (0.32 to 0.51) |
| Nauru | 4 (3 to 4) | 51.38 (44.73 to 58.89) |  | 4 (4 to 5) | 53.52 (46.42 to 60.83) | 0.19 (0.13 to 0.25) |
| Nepal | 5009 (4441 to 5619) | 47.67 (42.39 to 53.04) |  | 14807 (13218 to 16471) | 63.64 (57.04 to 70.52) | 0.87 (0.7 to 1.03) |
| Netherlands | 5964 (5035 to 7064) | 31.63 (26.56 to 37.74) |  | 15471 (13208 to 17837) | 48.95 (42.01 to 56.42) | 1.55 (1.49 to 1.62) |
| New Zealand | 2006 (1625 to 2429) | 51.78 (42.31 to 62.2) |  | 3986 (3365 to 4718) | 52.58 (44.76 to 61.44) | 0.11 (0.02 to 0.21) |
| Nicaragua | 522 (432 to 626) | 25.28 (20.7 to 30.47) |  | 1631 (1391 to 1912) | 32.85 (28.19 to 38.12) | 0.95 (0.76 to 1.15) |
| Niger | 1053 (887 to 1256) | 23.09 (19.49 to 27.1) |  | 2941 (2434 to 3538) | 22.22 (18.59 to 26.77) | -0.09 (-0.17 to -0.02) |
| Nigeria | 14901 (12398 to 17731) | 24.25 (20.32 to 28.82) |  | 32721 (26810 to 39838) | 22.1 (18.14 to 26.73) | -0.25 (-0.36 to -0.13) |
| Niue | 1 (1 to 1) | 48.03 (41.74 to 55.28) |  | 1 (1 to 1) | 56.82 (48.82 to 65.9) | 0.58 (0.51 to 0.65) |
| North Macedonia | 794 (639 to 988) | 38.6 (31.27 to 47.85) |  | 1340 (1080 to 1675) | 46.67 (37.58 to 57.98) | 0.73 (0.56 to 0.89) |
| Northern Mariana Islands | 36 (32 to 41) | 108.56 (97.22 to 120.36) |  | 50 (44 to 57) | 97.7 (86.59 to 108.46) | -0.49 (-0.64 to -0.34) |
| Norway | 4815 (3919 to 5736) | 81.37 (66.5 to 96.53) |  | 7041 (5728 to 8349) | 84.36 (69.44 to 99.63) | 0.78 (0.12 to 1.44) |
| Oman | 342 (279 to 422) | 28.43 (23.63 to 34.27) |  | 1303 (1051 to 1644) | 40.43 (33.75 to 47.83) | 1.24 (1.18 to 1.31) |
| Pakistan | 24785 (20836 to 29041) | 37.81 (31.35 to 44.58) |  | 45837 (38615 to 54104) | 33.05 (27.7 to 39.17) | -0.41 (-0.55 to -0.28) |
| Palau | 14 (13 to 16) | 106.74 (96.15 to 118.29) |  | 27 (24 to 30) | 123.9 (111.21 to 137.39) | 0.51 (0.44 to 0.59) |
| Palestine | 807 (713 to 903) | 80.98 (71.15 to 90.49) |  | 2636 (2331 to 2969) | 97.73 (86.59 to 109.18) | 0.65 (0.5 to 0.8) |
| Panama | 537 (464 to 622) | 32.43 (27.98 to 37.47) |  | 2232 (1983 to 2525) | 53.8 (47.72 to 60.71) | 1.84 (1.75 to 1.93) |
| Papua New Guinea | 2152 (1903 to 2449) | 74.57 (66.68 to 82.51) |  | 7308 (6506 to 8196) | 96.83 (86.84 to 106.75) | 0.87 (0.81 to 0.93) |
| Paraguay | 377 (318 to 447) | 14.63 (12.36 to 17.34) |  | 1163 (997 to 1343) | 20.09 (17.29 to 23.07) | 1.15 (1.08 to 1.21) |
| Peru | 10886 (9700 to 12036) | 89.18 (79.5 to 98.9) |  | 48180 (43197 to 52658) | 150.54 (134.76 to 164.83) | 1.83 (1.59 to 2.08) |
| Philippines | 4155 (3384 to 5065) | 10.52 (8.48 to 12.93) |  | 9819 (7872 to 12049) | 10.54 (8.47 to 12.93) | 0.07 (-0.02 to 0.15) |
| Poland | 24070 (20070 to 28559) | 56.79 (47.67 to 67.17) |  | 30816 (26803 to 34957) | 54.4 (47.71 to 61.41) | 0.07 (-0.36 to 0.51) |
| Portugal | 3965 (3374 to 4592) | 29.29 (24.87 to 33.87) |  | 10520 (9213 to 11803) | 48.24 (42.09 to 54.72) | 1.75 (1.65 to 1.84) |
| Puerto Rico | 1122 (985 to 1271) | 30.76 (26.89 to 34.97) |  | 2843 (2497 to 3210) | 45.12 (39.71 to 50.95) | 1.33 (1.21 to 1.45) |
| Qatar | 91 (72 to 119) | 29.84 (25.35 to 35.29) |  | 870 (688 to 1108) | 39.21 (33.2 to 46.58) | 0.97 (0.9 to 1.05) |
| Republic of Korea | 18295 (15213 to 21369) | 52.88 (44.99 to 60.7) |  | 89113 (76325 to 101212) | 102.19 (87.52 to 115.41) | 2.36 (2.2 to 2.51) |
| Republic of Moldova | 1794 (1427 to 2252) | 38.73 (31.1 to 48.58) |  | 2101 (1666 to 2642) | 42.74 (34 to 53.65) | 0.45 (0.3 to 0.61) |
| Romania | 21369 (18906 to 23816) | 77.59 (68.77 to 86.71) |  | 19241 (16419 to 22173) | 64.22 (54.94 to 74.02) | -0.71 (-0.82 to -0.6) |
| Russian Federation | 68125 (54998 to 84516) | 39.55 (31.94 to 49.1) |  | 75727 (60169 to 94696) | 39.96 (31.99 to 50.08) | 0.13 (-0.06 to 0.31) |
| Rwanda | 892 (754 to 1050) | 21.07 (17.88 to 24.57) |  | 2111 (1777 to 2521) | 25.38 (21.66 to 29.76) | 0.63 (0.55 to 0.7) |
| Saint Kitts and Nevis | 9 (8 to 10) | 24.74 (21.52 to 28.39) |  | 21 (18 to 25) | 31.18 (27.35 to 35.46) | 0.78 (0.73 to 0.84) |
| Saint Lucia | 24 (21 to 27) | 24.86 (21.62 to 28.47) |  | 76 (66 to 86) | 35.4 (31.21 to 39.84) | 1.19 (1.12 to 1.27) |
| Saint Vincent and the Grenadines | 13 (11 to 16) | 16.74 (13.97 to 20.02) |  | 30 (25 to 35) | 22.46 (19.21 to 26.26) | 1.03 (0.99 to 1.07) |
| Samoa | 67 (58 to 77) | 53.29 (46.72 to 59.86) |  | 100 (86 to 115) | 55.21 (47.76 to 62.75) | 0.13 (0.09 to 0.17) |
| San Marino | 8 (6 to 10) | 26.14 (20.8 to 31.96) |  | 14 (11 to 17) | 26.39 (21.12 to 32.52) | 0.11 (-0.02 to 0.23) |
| Sao Tome and Principe | 22 (19 to 25) | 28.24 (24.39 to 32.56) |  | 44 (38 to 52) | 31.53 (27.31 to 36.23) | 0.36 (0.31 to 0.41) |
| Saudi Arabia | 5021 (4388 to 5703) | 69.38 (61.24 to 77.25) |  | 24568 (21466 to 27974) | 114.44 (101.06 to 127.24) | 1.72 (1.65 to 1.79) |
| Senegal | 1009 (840 to 1202) | 21.34 (18.02 to 25.27) |  | 2362 (1954 to 2844) | 22 (18.29 to 26.52) | 0.16 (0.09 to 0.22) |
| Serbia | 3712 (3024 to 4507) | 33.41 (27.6 to 40.47) |  | 5107 (4227 to 6044) | 41.47 (34.22 to 49.81) | 1.11 (0.73 to 1.49) |
| Seychelles | 7 (6 to 9) | 12.5 (10.24 to 15.01) |  | 18 (14 to 22) | 15.67 (12.9 to 18.76) | 0.77 (0.73 to 0.81) |
| Sierra Leone | 517 (431 to 624) | 20.03 (16.7 to 24.16) |  | 1185 (971 to 1446) | 20.61 (16.9 to 24.98) | 0.16 (0.09 to 0.24) |
| Singapore | 2643 (2124 to 3229) | 94.82 (76.62 to 115.24) |  | 8492 (6891 to 10247) | 109.1 (90.04 to 129.76) | 0.55 (0.26 to 0.84) |
| Slovakia | 2369 (1954 to 2856) | 41.37 (34.13 to 50.07) |  | 4328 (3671 to 5090) | 55.16 (46.61 to 65.34) | 1.04 (0.98 to 1.11) |
| Slovenia | 1107 (908 to 1354) | 48.09 (39.54 to 58.67) |  | 2111 (1784 to 2497) | 64.92 (54.11 to 78.1) | 1.26 (0.99 to 1.54) |
| Solomon Islands | 97 (81 to 114) | 41.14 (35.14 to 47.25) |  | 240 (201 to 281) | 48.43 (41.95 to 55.71) | 0.61 (0.49 to 0.73) |
| Somalia | 876 (733 to 1060) | 21.07 (17.86 to 24.77) |  | 2408 (1978 to 2920) | 20.73 (17.38 to 24.66) | 0.01 (-0.08 to 0.09) |
| South Africa | 15245 (12904 to 17742) | 62.79 (52.55 to 73.96) |  | 26248 (22087 to 30972) | 53.23 (44.69 to 62.95) | -0.55 (-0.6 to -0.5) |
| South Sudan | 770 (653 to 905) | 22.6 (19.19 to 26.44) |  | 1307 (1097 to 1574) | 22.95 (19.54 to 27.02) | 0.08 (0.04 to 0.13) |
| Spain | 28142 (24859 to 31674) | 52.43 (46.22 to 59.13) |  | 66343 (58783 to 73780) | 73.34 (64.99 to 82.05) | 1.14 (1.09 to 1.19) |
| Sri Lanka | 1696 (1411 to 2010) | 13.71 (11.42 to 16.1) |  | 5231 (4407 to 6168) | 20.11 (17.05 to 23.46) | 1.27 (1.22 to 1.33) |
| Sudan | 3519 (2926 to 4223) | 27.21 (22.78 to 32.55) |  | 9471 (7908 to 11379) | 36.04 (30.41 to 42.55) | 1.03 (0.94 to 1.11) |
| Suriname | 60 (52 to 71) | 20.78 (17.8 to 24.27) |  | 162 (140 to 187) | 26.49 (23.02 to 30.33) | 0.83 (0.79 to 0.88) |
| Sweden | 7691 (6168 to 9377) | 55.26 (44.89 to 66.67) |  | 11557 (9244 to 14178) | 59.69 (48.43 to 72.1) | 0.36 (0.26 to 0.47) |
| Switzerland | 3950 (3421 to 4508) | 40.69 (35.01 to 46.98) |  | 6979 (6077 to 7960) | 44.15 (38.34 to 50.69) | 0.46 (0.26 to 0.66) |
| Syrian Arab Republic | 2419 (2057 to 2823) | 33.49 (28.46 to 39.14) |  | 6043 (5049 to 7157) | 44.62 (37.83 to 52.25) | 1.01 (0.97 to 1.04) |
| Taiwan (Province of China) | 2594 (2094 to 3151) | 15.38 (12.55 to 18.48) |  | 11777 (10037 to 13597) | 30.65 (26.27 to 35.24) | 2.44 (2.2 to 2.69) |
| Tajikistan | 2594 (2304 to 2881) | 88.9 (78.52 to 99.83) |  | 3678 (3233 to 4153) | 64.2 (56.86 to 71.96) | -1.15 (-1.31 to -1) |
| Thailand | 4757 (3897 to 5747) | 11.48 (9.49 to 13.7) |  | 13547 (10819 to 16738) | 13.55 (11 to 16.46) | 0.59 (0.57 to 0.61) |
| Timor-Leste | 43 (35 to 52) | 11.02 (9.11 to 13.13) |  | 145 (120 to 171) | 16.13 (13.39 to 19) | 1.41 (1.29 to 1.53) |
| Togo | 469 (390 to 564) | 22.2 (18.75 to 26.3) |  | 1245 (1022 to 1528) | 21.48 (17.7 to 26.05) | -0.06 (-0.13 to 0.01) |
| Tokelau | 1 (0 to 1) | 39.81 (34.18 to 45.98) |  | 1 (1 to 1) | 50.08 (43.03 to 57.45) | 0.83 (0.79 to 0.88) |
| Tonga | 34 (29 to 40) | 44.32 (38.36 to 51.01) |  | 48 (41 to 55) | 52.63 (45.77 to 60.23) | 0.65 (0.58 to 0.73) |
| Trinidad and Tobago | 242 (211 to 276) | 26.23 (23 to 29.91) |  | 595 (519 to 680) | 32.82 (28.7 to 37.27) | 0.78 (0.69 to 0.87) |
| Tunisia | 1829 (1523 to 2178) | 29.39 (24.46 to 34.9) |  | 5123 (4270 to 6088) | 39.78 (33.33 to 46.95) | 1.09 (0.97 to 1.21) |
| Turkey | 14627 (12430 to 17205) | 33.04 (28.36 to 38.69) |  | 45938 (39521 to 53113) | 50.91 (44.28 to 58.48) | 1.7 (1.48 to 1.92) |
| Turkmenistan | 671 (575 to 786) | 27.67 (23.79 to 32.41) |  | 1336 (1067 to 1643) | 28.24 (22.83 to 34.31) | 0.19 (0.02 to 0.37) |
| Tuvalu | 3 (3 to 4) | 39.86 (34.59 to 45.85) |  | 5 (5 to 6) | 48.96 (42.38 to 55.93) | 0.76 (0.7 to 0.81) |
| Uganda | 2080 (1760 to 2464) | 21.71 (18.57 to 25.49) |  | 5542 (4645 to 6585) | 25.43 (21.44 to 29.88) | 0.57 (0.54 to 0.61) |
| Ukraine | 44887 (37065 to 53547) | 67.38 (56.21 to 79.7) |  | 22098 (17905 to 27233) | 37.55 (30.3 to 46.3) | -1.94 (-2.13 to -1.75) |
| United Arab Emirates | 405 (329 to 500) | 35.58 (30.18 to 41.38) |  | 3932 (3196 to 4960) | 44.34 (38.03 to 51.36) | 0.79 (0.71 to 0.87) |
| United Kingdom | 58468 (47633 to 71307) | 67.8 (55.94 to 81.69) |  | 102511 (85399 to 121245) | 87.01 (72.96 to 102.04) | 0.87 (0.81 to 0.92) |
| United Republic of Tanzania | 3132 (2615 to 3741) | 19.71 (16.44 to 23.52) |  | 8084 (6711 to 9735) | 22.56 (18.76 to 26.88) | 0.51 (0.44 to 0.58) |
| United States of America | 357828 (296866 to 424682) | 117.3 (98.1 to 138.53) |  | 654842 (566536 to 745855) | 126 (110 to 142.04) | 0.34 (0.09 to 0.6) |
| United States Virgin Islands | 27 (24 to 31) | 29.7 (25.98 to 33.59) |  | 78 (69 to 88) | 45.1 (39.98 to 50.42) | 1.38 (1.13 to 1.64) |
| Uruguay | 1665 (1419 to 1918) | 44.3 (37.75 to 51.25) |  | 3155 (2800 to 3515) | 62.46 (55.16 to 70.1) | 1.22 (1.09 to 1.35) |
| Uzbekistan | 8506 (7571 to 9440) | 68.18 (60.83 to 75.58) |  | 11107 (9518 to 13028) | 44.3 (38.83 to 50.38) | -1.51 (-1.64 to -1.39) |
| Vanuatu | 51 (44 to 60) | 46.12 (40.32 to 52.22) |  | 119 (102 to 137) | 48.72 (42.34 to 55.28) | 0.19 (0.1 to 0.28) |
| Venezuela (Bolivarian Republic of) | 3449 (2943 to 4029) | 29.92 (25.53 to 34.86) |  | 10149 (8834 to 11681) | 34.51 (30.26 to 39.44) | 0.55 (0.43 to 0.67) |
| Viet Nam | 5362 (4463 to 6367) | 11.98 (9.93 to 14.2) |  | 18572 (15299 to 21976) | 19.69 (16.26 to 23.11) | 1.72 (1.67 to 1.77) |
| Yemen | 2110 (1757 to 2539) | 27.3 (22.72 to 32.61) |  | 6674 (5540 to 8080) | 33.48 (28.14 to 39.75) | 0.77 (0.69 to 0.86) |
| Zambia | 927 (775 to 1102) | 20.8 (17.67 to 24.35) |  | 2541 (2123 to 3050) | 24.02 (20.33 to 28.23) | 0.56 (0.47 to 0.66) |
| Zimbabwe | 2026 (1686 to 2430) | 32.67 (27.26 to 39.31) |  | 3471 (2854 to 4249) | 33.16 (27.41 to 40.33) | 0.18 (0.01 to 0.36) |

ILD&PS: Interstitial lung disease and pulmonary sarcoidosis; AAPC: average annual percent change; UI: uncertainty interval; CI: confidence interval; SDI: Socio-demographic index.

Table S12. Mortality of PNE (1990/2019) for sexes, SDI and locations, with AAPC (1990-2019)

| GBD data | 1990 | |  | 2019 | | AAPC % (95% CI)  1990-2019 |
| --- | --- | --- | --- | --- | --- | --- |
|  | Cases (95% UI) | Age-standardised mortality per  100 000 population (95% UI) |  | Cases (95% UI) | Age-standardised mortality per  100 000 population (95% UI) |  |
| Global | 23720 (18392 to 27667) | 0.62 (0.49 to 0.72) |  | 23015 (20348 to 26159) | 0.29 (0.26 to 0.33) | -2.6 (-2.74 to -2.46) |
| **Sex** |  |  |  |  |  |  |
| Female | 1376 (1067 to 2108) | 0.07 (0.05 to 0.1) |  | 2043 (1699 to 2388) | 0.05 (0.04 to 0.05) | -1.14 (-1.29 to -0.99) |
| Male | 22344 (17092 to 26335) | 1.36 (1.07 to 1.58) |  | 20972 (18482 to 24033) | 0.6 (0.53 to 0.69) | -2.77 (-2.93 to -2.61) |
| **SDI** |  |  |  |  |  |  |
| High SDI | 5855 (5542 to 6121) | 0.54 (0.51 to 0.57) |  | 5698 (4998 to 6448) | 0.26 (0.23 to 0.3) | -2.47 (-2.68 to -2.26) |
| High-middle SDI | 6386 (5326 to 7276) | 0.62 (0.52 to 0.7) |  | 4537 (3884 to 6336) | 0.23 (0.19 to 0.32) | -3.43 (-3.72 to -3.14) |
| Middle SDI | 7426 (5053 to 9464) | 0.74 (0.52 to 0.94) |  | 7685 (6289 to 9407) | 0.33 (0.27 to 0.4) | -2.75 (-2.98 to -2.51) |
| Low-middle SDI | 3188 (1664 to 4207) | 0.57 (0.3 to 0.75) |  | 3886 (2825 to 4923) | 0.31 (0.22 to 0.38) | -2.16 (-2.26 to -2.05) |
| Low SDI | 860 (298 to 1260) | 0.41 (0.14 to 0.59) |  | 1204 (542 to 1721) | 0.26 (0.12 to 0.37) | -1.57 (-1.71 to -1.42) |
| **Region** |  |  |  |  |  |  |
| High-income Asia Pacific | 1218 (1105 to 1333) | 0.61 (0.55 to 0.67) |  | 1565 (1249 to 1938) | 0.28 (0.23 to 0.35) | -2.81 (-3.12 to -2.51) |
| High-income North America | 1282 (1211 to 1345) | 0.34 (0.32 to 0.36) |  | 1263 (1135 to 1354) | 0.19 (0.17 to 0.2) | -2.01 (-2.25 to -1.78) |
| Western Europe | 4248 (4025 to 4452) | 0.69 (0.66 to 0.73) |  | 2891 (2551 to 3210) | 0.26 (0.23 to 0.29) | -3.41 (-3.64 to -3.19) |
| Australasia | 47 (42 to 52) | 0.19 (0.17 to 0.22) |  | 180 (147 to 216) | 0.32 (0.27 to 0.39) | 1.84 (1.55 to 2.13) |
| Andean Latin America | 95 (72 to 117) | 0.46 (0.35 to 0.58) |  | 121 (88 to 161) | 0.22 (0.16 to 0.3) | -1.98 (-4.06 to 0.14) |
| Tropical Latin America | 205 (191 to 219) | 0.22 (0.2 to 0.24) |  | 502 (460 to 546) | 0.21 (0.19 to 0.23) | -0.04 (-0.24 to 0.17) |
| Central Latin America | 246 (230 to 260) | 0.32 (0.29 to 0.33) |  | 260 (220 to 307) | 0.11 (0.1 to 0.13) | -3.82 (-4.13 to -3.51) |
| Southern Latin America | 142 (127 to 157) | 0.32 (0.28 to 0.35) |  | 167 (143 to 200) | 0.2 (0.17 to 0.23) | -1.48 (-1.98 to -0.97) |
| Caribbean | 18 (12 to 23) | 0.07 (0.05 to 0.09) |  | 21 (14 to 31) | 0.04 (0.03 to 0.06) | -1.79 (-2.12 to -1.46) |
| Central Europe | 809 (760 to 860) | 0.55 (0.51 to 0.58) |  | 250 (212 to 289) | 0.11 (0.09 to 0.13) | -5.37 (-5.6 to -5.14) |
| Eastern Europe | 515 (410 to 664) | 0.19 (0.15 to 0.24) |  | 279 (239 to 340) | 0.08 (0.07 to 0.1) | -3.42 (-3.88 to -2.96) |
| Central Asia | 81 (63 to 99) | 0.18 (0.14 to 0.21) |  | 96 (75 to 119) | 0.15 (0.12 to 0.19) | 0.09 (-1.01 to 1.21) |
| North Africa and Middle East | 147 (103 to 187) | 0.09 (0.06 to 0.11) |  | 289 (244 to 344) | 0.07 (0.06 to 0.09) | -0.61 (-0.75 to -0.47) |
| South Asia | 2598 (844 to 3830) | 0.54 (0.18 to 0.78) |  | 3389 (1941 to 4784) | 0.27 (0.16 to 0.37) | -2.37 (-2.58 to -2.15) |
| Southeast Asia | 87 (64 to 106) | 0.04 (0.03 to 0.05) |  | 122 (101 to 154) | 0.02 (0.02 to 0.03) | -1.95 (-2.07 to -1.83) |
| East Asia | 11367 (7996 to 14368) | 1.36 (0.97 to 1.71) |  | 10708 (8559 to 14012) | 0.54 (0.44 to 0.71) | -3.02 (-3.33 to -2.72) |
| Oceania | 7 (4 to 10) | 0.36 (0.18 to 0.54) |  | 13 (8 to 19) | 0.28 (0.15 to 0.4) | -0.92 (-1.01 to -0.83) |
| Western Sub-Saharan Africa | 40 (21 to 57) | 0.05 (0.03 to 0.07) |  | 93 (66 to 117) | 0.04 (0.03 to 0.05) | -0.52 (-0.7 to -0.34) |
| Eastern Sub-Saharan Africa | 320 (112 to 483) | 0.46 (0.16 to 0.68) |  | 417 (166 to 625) | 0.28 (0.11 to 0.41) | -1.7 (-1.79 to -1.61) |
| Central Sub-Saharan Africa | 101 (56 to 151) | 0.52 (0.3 to 0.76) |  | 169 (90 to 274) | 0.37 (0.21 to 0.59) | -1.18 (-1.32 to -1.03) |
| Southern Sub-Saharan Africa | 146 (121 to 177) | 0.58 (0.48 to 0.7) |  | 219 (195 to 246) | 0.44 (0.39 to 0.49) | -0.54 (-0.97 to -0.11) |
| **Countries** |  |  |  |  |  |  |
| Afghanistan | 106 (84 to 132) | 1.41 (1.14 to 1.74) |  | 592 (442 to 778) | 2.88 (2.28 to 3.56) | 2.48 (2.12 to 2.85) |
| Albania | 310 (252 to 387) | 14.29 (11.66 to 17.5) |  | 700 (553 to 897) | 16.45 (13.14 to 20.94) | 0.61 (0.2 to 1.03) |
| Algeria | 52 (41 to 66) | 0.41 (0.34 to 0.51) |  | 231 (192 to 280) | 0.72 (0.61 to 0.87) | 2.03 (1.85 to 2.2) |
| American Samoa | 1 (1 to 1) | 3.96 (3.16 to 5.06) |  | 3 (2 to 4) | 6.29 (4.86 to 7.98) | 1.58 (1.47 to 1.69) |
| Andorra | 0 (0 to 0) | 0.15 (0.12 to 0.2) |  | 0 (0 to 0) | 0.12 (0.1 to 0.16) | -1.15 (-1.76 to -0.53) |
| Angola | 115 (97 to 135) | 3.31 (2.84 to 3.87) |  | 350 (294 to 410) | 3.7 (3.11 to 4.33) | 0.31 (0.16 to 0.47) |
| Antigua and Barbuda | 1 (0 to 1) | 1.05 (0.83 to 1.33) |  | 1 (1 to 1) | 0.88 (0.69 to 1.09) | -0.72 (-1.03 to -0.41) |
| Argentina | 720 (559 to 918) | 2.24 (1.75 to 2.84) |  | 2077 (1626 to 2683) | 3.74 (2.94 to 4.83) | 1.76 (1.7 to 1.83) |
| Armenia | 100 (83 to 120) | 3.74 (3.1 to 4.5) |  | 204 (165 to 256) | 4.89 (3.99 to 6.15) | 0.94 (0.85 to 1.02) |
| Australia | 404 (334 to 485) | 2.01 (1.68 to 2.41) |  | 1164 (926 to 1596) | 2.58 (2.06 to 3.52) | 0.96 (0.46 to 1.47) |
| Austria | 66 (53 to 81) | 0.56 (0.45 to 0.69) |  | 140 (110 to 178) | 0.78 (0.62 to 0.99) | 1.2 (0.84 to 1.57) |
| Azerbaijan | 99 (76 to 126) | 2.08 (1.6 to 2.68) |  | 300 (235 to 381) | 3.53 (2.77 to 4.54) | 1.88 (1.76 to 1.99) |
| Bahamas | 1 (1 to 1) | 0.68 (0.54 to 0.86) |  | 3 (2 to 3) | 0.7 (0.55 to 0.86) | 0.01 (-0.15 to 0.18) |
| Bahrain | 3 (2 to 4) | 1.01 (0.84 to 1.21) |  | 14 (11 to 18) | 1.26 (1.05 to 1.5) | 0.3 (-1.08 to 1.71) |
| Bangladesh | 1612 (1330 to 1954) | 3.52 (2.89 to 4.25) |  | 5475 (4381 to 6748) | 4.39 (3.51 to 5.37) | 0.77 (0.53 to 1.02) |
| Barbados | 2 (1 to 2) | 0.54 (0.42 to 0.69) |  | 3 (2 to 4) | 0.63 (0.49 to 0.81) | 0.49 (0.41 to 0.57) |
| Belarus | 768 (624 to 938) | 6.02 (4.89 to 7.34) |  | 1177 (944 to 1444) | 7.67 (6.17 to 9.46) | 0.84 (0.79 to 0.89) |
| Belgium | 355 (300 to 416) | 2.22 (1.88 to 2.61) |  | 110 (90 to 134) | 0.42 (0.35 to 0.51) | -6.32 (-7.11 to -5.53) |
| Belize | 1 (1 to 1) | 0.72 (0.59 to 0.89) |  | 3 (2 to 3) | 0.85 (0.69 to 1.04) | 0.46 (0.27 to 0.65) |
| Benin | 20 (17 to 24) | 0.91 (0.76 to 1.1) |  | 53 (43 to 63) | 0.96 (0.81 to 1.14) | 0.18 (0.12 to 0.24) |
| Bermuda | 1 (1 to 1) | 1.17 (0.98 to 1.41) |  | 1 (1 to 1) | 0.77 (0.62 to 0.96) | -1.56 (-1.68 to -1.43) |
| Bhutan | 6 (5 to 8) | 2.69 (2.23 to 3.31) |  | 21 (17 to 26) | 3.97 (3.23 to 4.81) | 1.24 (1.1 to 1.37) |
| Bolivia (Plurinational State of) | 89 (71 to 122) | 2.77 (2.2 to 3.8) |  | 388 (307 to 495) | 4.61 (3.66 to 5.88) | 1.67 (1.41 to 1.93) |
| Bosnia and Herzegovina | 231 (184 to 289) | 5.5 (4.46 to 6.78) |  | 541 (428 to 674) | 9.45 (7.57 to 11.78) | 1.93 (1.81 to 2.04) |
| Botswana | 60 (49 to 77) | 10.72 (8.84 to 13.65) |  | 149 (123 to 177) | 11.61 (9.68 to 13.73) | -0.05 (-0.41 to 0.3) |
| Brazil | 22221 (18797 to 27665) | 21.41 (18 to 26.55) |  | 49744 (41788 to 60362) | 20.6 (17.33 to 24.95) | -0.37 (-0.83 to 0.1) |
| Brunei Darussalam | 3 (2 to 4) | 3.71 (2.99 to 4.65) |  | 9 (8 to 12) | 4.16 (3.31 to 5.28) | 0.51 (0.35 to 0.67) |
| Bulgaria | 2128 (1761 to 2588) | 16.68 (13.85 to 20.16) |  | 1833 (1499 to 2211) | 13.13 (10.76 to 15.91) | -0.99 (-1.36 to -0.62) |
| Burkina Faso | 48 (39 to 58) | 1.01 (0.84 to 1.22) |  | 86 (70 to 104) | 0.86 (0.72 to 1.03) | -0.6 (-0.7 to -0.49) |
| Burundi | 91 (77 to 106) | 3.87 (3.28 to 4.51) |  | 154 (127 to 185) | 3.83 (3.14 to 4.58) | -0.3 (-0.6 to 0.01) |
| Cabo Verde | 1 (1 to 2) | 0.55 (0.45 to 0.66) |  | 3 (2 to 3) | 0.64 (0.52 to 0.77) | 0.41 (0.25 to 0.57) |
| Cambodia | 116 (86 to 153) | 2.35 (1.73 to 3.08) |  | 494 (363 to 652) | 3.9 (2.9 to 5.11) | 1.82 (1.73 to 1.92) |
| Cameroon | 66 (54 to 82) | 1.3 (1.08 to 1.58) |  | 175 (143 to 212) | 1.24 (1.04 to 1.48) | -0.23 (-0.33 to -0.13) |
| Canada | 3069 (2538 to 3723) | 9.29 (7.72 to 11.15) |  | 5705 (4761 to 6855) | 8.3 (6.97 to 9.9) | -0.41 (-0.54 to -0.27) |
| Central African Republic | 39 (33 to 45) | 3.65 (3.11 to 4.23) |  | 74 (63 to 87) | 3.94 (3.37 to 4.59) | 0.12 (0.01 to 0.22) |
| Chad | 27 (22 to 32) | 0.88 (0.73 to 1.06) |  | 55 (45 to 67) | 0.9 (0.74 to 1.07) | 0.04 (0 to 0.08) |
| Chile | 1155 (938 to 1427) | 11.97 (9.75 to 14.76) |  | 4018 (3168 to 5129) | 16.61 (13.1 to 21.2) | 0.86 (0.32 to 1.4) |
| China | 1330860 (1083676 to 1672573) | 143.1 (117.11 to 178.64) |  | 2587176 (2153538 to 3082660) | 123.12 (102.96 to 146.36) | -0.59 (-0.96 to -0.21) |
| Colombia | 940 (779 to 1126) | 5.12 (4.28 to 6.07) |  | 2737 (2231 to 3398) | 5.2 (4.23 to 6.45) | 0.02 (-0.14 to 0.18) |
| Comoros | 6 (6 to 8) | 2.98 (2.54 to 3.47) |  | 15 (13 to 18) | 3.23 (2.72 to 3.81) | 0.11 (-0.09 to 0.31) |
| Congo | 40 (34 to 47) | 3.9 (3.37 to 4.5) |  | 103 (88 to 120) | 4.4 (3.77 to 5.14) | 0.42 (0.22 to 0.62) |
| Cook Islands | 1 (0 to 1) | 4.09 (3.16 to 5.3) |  | 2 (1 to 2) | 6.08 (4.66 to 7.82) | 1.43 (1.29 to 1.58) |
| Costa Rica | 62 (50 to 78) | 3.24 (2.59 to 4.08) |  | 221 (179 to 277) | 4.29 (3.46 to 5.38) | 0.96 (0.92 to 1) |
| Croatia | 596 (490 to 726) | 9.39 (7.76 to 11.41) |  | 915 (742 to 1104) | 11.46 (9.38 to 13.86) | 0.69 (0.64 to 0.74) |
| Cuba | 58 (45 to 73) | 0.56 (0.43 to 0.7) |  | 119 (92 to 152) | 0.65 (0.51 to 0.83) | 0.52 (0.47 to 0.56) |
| Cyprus | 1 (1 to 1) | 0.12 (0.1 to 0.16) |  | 1 (1 to 2) | 0.09 (0.07 to 0.12) | -1.28 (-2.21 to -0.34) |
| Czechia | 804 (633 to 1063) | 5.92 (4.66 to 7.76) |  | 1711 (1372 to 2094) | 8.78 (6.98 to 10.95) | 1.38 (1.04 to 1.72) |
| Côte d’Ivoire | 63 (50 to 78) | 1.24 (1.01 to 1.5) |  | 156 (125 to 190) | 1.22 (1.02 to 1.45) | -0.05 (-0.17 to 0.08) |
| Democratic People's Republic of Korea | 6928 (5440 to 12604) | 38.36 (30.45 to 67.58) |  | 20522 (16087 to 28148) | 61.5 (48.34 to 83.93) | 1.07 (0.54 to 1.6) |
| Democratic Republic of the Congo | 461 (390 to 541) | 3.35 (2.84 to 3.9) |  | 1249 (1064 to 1452) | 4.08 (3.48 to 4.76) | 0.58 (0.42 to 0.73) |
| Denmark | 15 (12 to 18) | 0.18 (0.14 to 0.23) |  | 11 (8 to 14) | 0.1 (0.07 to 0.14) | -2.38 (-2.89 to -1.87) |
| Djibouti | 4 (3 to 4) | 2.93 (2.46 to 3.45) |  | 19 (16 to 23) | 3.64 (3.01 to 4.33) | 0.56 (0.38 to 0.75) |
| Dominica | 0 (0 to 0) | 0.55 (0.43 to 0.7) |  | 1 (0 to 1) | 0.66 (0.52 to 0.84) | 0.64 (0.6 to 0.68) |
| Dominican Republic | 22 (17 to 28) | 0.53 (0.41 to 0.68) |  | 59 (46 to 74) | 0.62 (0.49 to 0.79) | 0.52 (0.5 to 0.54) |
| Ecuador | 113 (92 to 143) | 2.1 (1.69 to 2.64) |  | 510 (415 to 624) | 3.44 (2.79 to 4.21) | 1.87 (1.64 to 2.11) |
| Egypt | 148 (117 to 183) | 0.47 (0.38 to 0.56) |  | 616 (494 to 746) | 0.96 (0.79 to 1.14) | 2.54 (2.33 to 2.76) |
| El Salvador | 100 (80 to 125) | 3.21 (2.54 to 4.02) |  | 236 (190 to 298) | 3.95 (3.17 to 5.01) | 0.73 (0.68 to 0.77) |
| Equatorial Guinea | 6 (5 to 7) | 3.43 (2.96 to 3.99) |  | 14 (12 to 17) | 3.44 (2.93 to 4.04) | -0.11 (-0.32 to 0.09) |
| Eritrea | 33 (28 to 39) | 3.24 (2.75 to 3.8) |  | 89 (75 to 105) | 3.48 (2.94 to 4.07) | 0.13 (0.01 to 0.26) |
| Estonia | 106 (85 to 131) | 5.3 (4.27 to 6.56) |  | 181 (143 to 225) | 7.59 (6.01 to 9.55) | 1.26 (1.17 to 1.35) |
| Eswatini | 42 (35 to 53) | 14.92 (12.46 to 18.88) |  | 84 (70 to 102) | 14.86 (12.54 to 18.1) | 0.04 (-0.44 to 0.51) |
| Ethiopia | 895 (758 to 1056) | 4.15 (3.53 to 4.88) |  | 1180 (1002 to 1399) | 2.88 (2.45 to 3.42) | -1.34 (-1.41 to -1.26) |
| Fiji | 15 (11 to 19) | 3.97 (3.11 to 5.16) |  | 44 (33 to 57) | 5.76 (4.38 to 7.39) | 1.31 (1.22 to 1.39) |
| Finland | 20 (16 to 24) | 0.28 (0.23 to 0.34) |  | 30 (24 to 37) | 0.25 (0.2 to 0.31) | 0.07 (-0.73 to 0.87) |
| France | 797 (666 to 959) | 0.92 (0.77 to 1.1) |  | 563 (455 to 693) | 0.36 (0.29 to 0.44) | -4.21 (-5.62 to -2.77) |
| Gabon | 17 (15 to 20) | 3.19 (2.74 to 3.73) |  | 37 (31 to 43) | 3.82 (3.23 to 4.5) | 0.52 (0.44 to 0.59) |
| Gambia | 4 (3 to 5) | 1.03 (0.85 to 1.24) |  | 11 (9 to 13) | 1.05 (0.89 to 1.25) | -0.02 (-0.15 to 0.12) |
| Georgia | 126 (98 to 163) | 2.07 (1.62 to 2.63) |  | 242 (197 to 298) | 3.99 (3.26 to 4.86) | 2.41 (2.25 to 2.58) |
| Germany | 623 (500 to 775) | 0.48 (0.38 to 0.59) |  | 609 (479 to 770) | 0.28 (0.22 to 0.34) | -2.45 (-3.48 to -1.41) |
| Ghana | 116 (92 to 143) | 1.53 (1.26 to 1.87) |  | 179 (145 to 216) | 0.99 (0.83 to 1.17) | -1.55 (-1.74 to -1.35) |
| Greece | 9 (6 to 12) | 0.07 (0.05 to 0.1) |  | 11 (8 to 15) | 0.07 (0.04 to 0.09) | -0.11 (-0.47 to 0.25) |
| Greenland | 3 (3 to 4) | 10.92 (8.51 to 13.9) |  | 6 (4 to 7) | 8.49 (6.65 to 10.72) | -1.14 (-1.96 to -0.31) |
| Grenada | 1 (1 to 1) | 0.92 (0.77 to 1.1) |  | 1 (1 to 1) | 0.84 (0.69 to 1.04) | -0.51 (-0.73 to -0.29) |
| Guam | 3 (2 to 4) | 3.99 (3.18 to 5.11) |  | 12 (9 to 15) | 6.05 (4.63 to 7.72) | 1.44 (1.32 to 1.57) |
| Guatemala | 153 (122 to 188) | 3.75 (3.05 to 4.62) |  | 478 (384 to 590) | 4.03 (3.23 to 4.96) | 0.12 (-0.14 to 0.37) |
| Guinea | 36 (29 to 43) | 1.01 (0.84 to 1.22) |  | 71 (58 to 85) | 1.16 (0.97 to 1.37) | 0.42 (0.35 to 0.5) |
| Guinea-Bissau | 8 (6 to 10) | 1.65 (1.35 to 1.99) |  | 13 (10 to 16) | 1.42 (1.18 to 1.72) | -0.51 (-0.59 to -0.43) |
| Guyana | 3 (2 to 4) | 0.7 (0.56 to 0.87) |  | 5 (4 to 6) | 0.73 (0.59 to 0.92) | 0.11 (0.04 to 0.19) |
| Haiti | 25 (20 to 31) | 0.7 (0.58 to 0.86) |  | 62 (50 to 77) | 0.82 (0.67 to 1.02) | 0.48 (0.37 to 0.6) |
| Honduras | 83 (68 to 102) | 3.67 (3.02 to 4.49) |  | 284 (228 to 349) | 4.44 (3.62 to 5.48) | 0.62 (0.54 to 0.69) |
| Hungary | 1380 (1114 to 1681) | 9.47 (7.66 to 11.53) |  | 2283 (1846 to 2807) | 12.57 (10.16 to 15.45) | 0.9 (0.48 to 1.32) |
| Iceland | 0 (0 to 0) | 0.07 (0.04 to 0.1) |  | 0 (0 to 0) | 0.06 (0.03 to 0.09) | -0.65 (-0.9 to -0.39) |
| India | 35898 (29665 to 43411) | 7.98 (6.65 to 9.56) |  | 79358 (66736 to 96189) | 6.87 (5.82 to 8.3) | -0.55 (-0.65 to -0.45) |
| Indonesia | 7827 (5933 to 10751) | 6.66 (5.08 to 8.87) |  | 18865 (15028 to 24300) | 7.74 (6.21 to 9.78) | 0.6 (0.2 to 1) |
| Iran (Islamic Republic of) | 575 (457 to 711) | 1.75 (1.46 to 2.08) |  | 5749 (4497 to 7201) | 6.93 (5.57 to 8.46) | 4.7 (4.12 to 5.28) |
| Iraq | 121 (98 to 147) | 1.3 (1.08 to 1.55) |  | 249 (202 to 300) | 1.05 (0.89 to 1.25) | -0.96 (-1.25 to -0.68) |
| Ireland | 3 (2 to 5) | 0.09 (0.06 to 0.12) |  | 6 (4 to 8) | 0.08 (0.06 to 0.12) | -0.33 (-0.74 to 0.08) |
| Israel | 3 (2 to 5) | 0.07 (0.05 to 0.1) |  | 6 (4 to 9) | 0.06 (0.04 to 0.09) | -0.45 (-0.61 to -0.3) |
| Italy | 58800 (45508 to 78288) | 63.64 (49.34 to 85.01) |  | 26559 (22575 to 31730) | 18.06 (15.36 to 21.21) | -4.5 (-4.89 to -4.1) |
| Jamaica | 16 (13 to 20) | 0.87 (0.7 to 1.09) |  | 19 (15 to 25) | 0.63 (0.48 to 0.81) | -1.48 (-2.18 to -0.78) |
| Japan | 60886 (48912 to 77310) | 35.77 (28.87 to 45.19) |  | 52224 (44054 to 61549) | 12.79 (10.81 to 15.2) | -4.02 (-4.55 to -3.49) |
| Jordan | 20 (15 to 26) | 1.02 (0.83 to 1.23) |  | 59 (47 to 73) | 0.78 (0.64 to 0.94) | -0.97 (-1.16 to -0.77) |
| Kazakhstan | 647 (527 to 794) | 5.31 (4.32 to 6.52) |  | 1152 (934 to 1463) | 7.02 (5.65 to 8.84) | 0.54 (0.03 to 1.05) |
| Kenya | 297 (255 to 348) | 3.76 (3.2 to 4.41) |  | 770 (659 to 901) | 3.64 (3.11 to 4.3) | -0.26 (-0.48 to -0.03) |
| Kiribati | 2 (2 to 3) | 5.36 (4.2 to 7.13) |  | 5 (4 to 7) | 7.84 (6.07 to 10.49) | 1.19 (1.03 to 1.36) |
| Kuwait | 5 (4 to 7) | 0.55 (0.45 to 0.66) |  | 33 (25 to 41) | 0.91 (0.75 to 1.09) | 1.47 (0.95 to 2) |
| Kyrgyzstan | 62 (49 to 80) | 2.09 (1.62 to 2.7) |  | 147 (116 to 188) | 3.49 (2.75 to 4.53) | 1.83 (1.71 to 1.94) |
| Lao People's Democratic Republic | 58 (42 to 79) | 2.56 (1.89 to 3.46) |  | 211 (154 to 281) | 4.49 (3.3 to 6.01) | 1.99 (1.92 to 2.07) |
| Latvia | 290 (225 to 388) | 8.22 (6.37 to 10.98) |  | 258 (205 to 322) | 7.45 (5.77 to 9.35) | -0.32 (-0.44 to -0.21) |
| Lebanon | 12 (10 to 15) | 0.53 (0.43 to 0.63) |  | 36 (30 to 43) | 0.69 (0.58 to 0.83) | 1 (0.76 to 1.24) |
| Lesotho | 131 (108 to 173) | 13.51 (11.2 to 17.63) |  | 262 (216 to 324) | 20.64 (17.17 to 25.41) | 1.23 (0.96 to 1.49) |
| Liberia | 10 (8 to 12) | 0.87 (0.72 to 1.04) |  | 21 (17 to 25) | 0.91 (0.76 to 1.08) | 0.21 (0.05 to 0.37) |
| Libya | 10 (8 to 13) | 0.48 (0.4 to 0.59) |  | 61 (49 to 74) | 1.06 (0.89 to 1.27) | 2.78 (2.6 to 2.97) |
| Lithuania | 355 (297 to 421) | 7.98 (6.7 to 9.48) |  | 492 (406 to 596) | 9.09 (7.5 to 10.94) | 0.27 (0.09 to 0.45) |
| Luxembourg | 2 (1 to 2) | 0.27 (0.21 to 0.35) |  | 1 (1 to 2) | 0.13 (0.1 to 0.17) | -3.17 (-4.08 to -2.25) |
| Madagascar | 162 (134 to 193) | 3.41 (2.82 to 4.05) |  | 346 (282 to 420) | 3.81 (3.12 to 4.62) | 0.23 (-0.06 to 0.51) |
| Malawi | 97 (81 to 114) | 2.66 (2.25 to 3.15) |  | 216 (180 to 256) | 3.16 (2.63 to 3.79) | 0.45 (0.28 to 0.62) |
| Malaysia | 256 (191 to 343) | 2.55 (1.92 to 3.39) |  | 1337 (965 to 1766) | 4.85 (3.52 to 6.37) | 2.24 (2.17 to 2.31) |
| Maldives | 4 (3 to 6) | 4.18 (3.28 to 5.4) |  | 19 (14 to 25) | 5.4 (3.95 to 7.14) | 0.92 (0.85 to 0.99) |
| Mali | 48 (39 to 59) | 1.07 (0.89 to 1.3) |  | 77 (64 to 93) | 0.85 (0.72 to 1.02) | -0.8 (-0.92 to -0.69) |
| Malta | 0 (0 to 1) | 0.12 (0.09 to 0.15) |  | 1 (1 to 1) | 0.1 (0.07 to 0.13) | -1.01 (-1.54 to -0.47) |
| Marshall Islands | 1 (1 to 1) | 4.15 (3.31 to 5.25) |  | 2 (2 to 3) | 6.43 (5.05 to 8.08) | 1.52 (1.46 to 1.58) |
| Mauritania | 15 (12 to 18) | 1.32 (1.1 to 1.59) |  | 21 (17 to 25) | 0.95 (0.79 to 1.12) | -1.17 (-1.37 to -0.97) |
| Mauritius | 18 (14 to 24) | 2.3 (1.72 to 3.02) |  | 79 (56 to 106) | 4.4 (3.18 to 5.93) | 2.3 (2.22 to 2.38) |
| Mexico | 13241 (11018 to 15758) | 29.75 (24.58 to 35.98) |  | 24062 (20355 to 28617) | 19.98 (16.9 to 23.91) | -1.37 (-1.71 to -1.03) |
| Micronesia (Federated States of) | 2 (2 to 2) | 4.18 (3.37 to 5.24) |  | 4 (3 to 6) | 5.98 (4.67 to 7.53) | 1.25 (1.21 to 1.29) |
| Monaco | 0 (0 to 0) | 0.38 (0.3 to 0.47) |  | 0 (0 to 1) | 0.39 (0.31 to 0.48) | -0.46 (-1.67 to 0.76) |
| Mongolia | 35 (28 to 44) | 3.3 (2.62 to 4.14) |  | 84 (68 to 104) | 4.12 (3.33 to 5.12) | 0.78 (0.68 to 0.89) |
| Montenegro | 37 (31 to 46) | 5.93 (4.88 to 7.25) |  | 90 (72 to 110) | 9.44 (7.58 to 11.54) | 1.67 (1.59 to 1.76) |
| Morocco | 65 (51 to 81) | 0.44 (0.36 to 0.53) |  | 254 (206 to 308) | 0.84 (0.69 to 0.99) | 2.23 (2.05 to 2.41) |
| Mozambique | 153 (128 to 181) | 2.73 (2.29 to 3.22) |  | 343 (278 to 427) | 3.39 (2.74 to 4.24) | 0.61 (0.42 to 0.79) |
| Myanmar | 837 (659 to 1066) | 3.14 (2.48 to 3.98) |  | 2124 (1614 to 2747) | 4.32 (3.26 to 5.55) | 1.13 (1.02 to 1.23) |
| Namibia | 79 (67 to 93) | 11 (9.3 to 12.8) |  | 150 (127 to 175) | 10.98 (9.26 to 12.8) | -0.03 (-0.16 to 0.09) |
| Nauru | 0 (0 to 0) | 4.01 (3.15 to 5.22) |  | 0 (0 to 0) | 5.65 (4.39 to 7.11) | 1.28 (1.18 to 1.38) |
| Nepal | 306 (244 to 381) | 3.43 (2.78 to 4.22) |  | 890 (728 to 1086) | 4.23 (3.48 to 5.13) | 0.67 (0.49 to 0.86) |
| Netherlands | 118 (93 to 150) | 0.56 (0.44 to 0.71) |  | 31 (24 to 40) | 0.1 (0.08 to 0.14) | -6.03 (-6.74 to -5.31) |
| New Zealand | 262 (214 to 317) | 6.49 (5.34 to 7.82) |  | 495 (418 to 583) | 6.17 (5.25 to 7.24) | -0.3 (-0.92 to 0.32) |
| Nicaragua | 59 (48 to 73) | 3.5 (2.81 to 4.29) |  | 192 (152 to 242) | 4.1 (3.26 to 5.19) | 0.5 (0.4 to 0.59) |
| Niger | 30 (25 to 36) | 0.93 (0.78 to 1.11) |  | 68 (56 to 81) | 0.81 (0.69 to 0.96) | -0.49 (-0.61 to -0.37) |
| Nigeria | 686 (564 to 830) | 1.37 (1.15 to 1.64) |  | 889 (741 to 1055) | 0.92 (0.77 to 1.08) | -1.37 (-1.58 to -1.15) |
| Niue | 0 (0 to 0) | 3.48 (2.72 to 4.51) |  | 0 (0 to 0) | 5.68 (4.35 to 7.24) | 1.77 (1.68 to 1.87) |
| North Macedonia | 175 (140 to 243) | 9.16 (7.35 to 12.57) |  | 298 (239 to 369) | 9.44 (7.63 to 11.58) | -0.04 (-0.2 to 0.12) |
| Northern Mariana Islands | 1 (1 to 1) | 4.35 (3.44 to 5.61) |  | 3 (2 to 4) | 6.05 (4.6 to 7.8) | 1.19 (0.97 to 1.41) |
| Norway | 728 (587 to 895) | 10.67 (8.67 to 13.04) |  | 699 (571 to 861) | 8.08 (6.62 to 10.07) | -1.09 (-1.28 to -0.9) |
| Oman | 5 (4 to 7) | 0.57 (0.47 to 0.69) |  | 18 (14 to 23) | 0.79 (0.66 to 0.94) | 1.03 (0.82 to 1.23) |
| Pakistan | 2008 (1634 to 2434) | 3.58 (2.91 to 4.36) |  | 3251 (2681 to 3897) | 3 (2.49 to 3.6) | -0.7 (-0.81 to -0.59) |
| Palau | 1 (1 to 1) | 10.28 (8.3 to 12.57) |  | 3 (3 to 4) | 15.02 (12.01 to 18.4) | 0.94 (0.41 to 1.48) |
| Palestine | 5 (4 to 6) | 0.5 (0.41 to 0.6) |  | 14 (11 to 18) | 0.54 (0.45 to 0.66) | 0.36 (0.16 to 0.56) |
| Panama | 53 (43 to 66) | 3.31 (2.65 to 4.07) |  | 176 (141 to 222) | 4.24 (3.4 to 5.35) | 0.89 (0.84 to 0.94) |
| Papua New Guinea | 90 (71 to 113) | 4.84 (3.87 to 5.95) |  | 329 (256 to 416) | 6.94 (5.46 to 8.64) | 1.23 (1.14 to 1.33) |
| Paraguay | 341 (281 to 453) | 13.64 (11.26 to 18.56) |  | 884 (740 to 1061) | 14.88 (12.47 to 17.75) | -0.04 (-0.46 to 0.38) |
| Peru | 479 (390 to 615) | 4.03 (3.25 to 5.19) |  | 1832 (1450 to 2275) | 5.82 (4.59 to 7.24) | 0.99 (0.4 to 1.59) |
| Philippines | 830 (641 to 1074) | 2.5 (1.94 to 3.26) |  | 3170 (2373 to 4217) | 3.73 (2.84 to 4.98) | 1.5 (1.31 to 1.69) |
| Poland | 11240 (9073 to 14610) | 25.73 (20.74 to 33.5) |  | 8970 (7678 to 10485) | 13.44 (11.57 to 15.64) | -2.39 (-2.68 to -2.08) |
| Portugal | 182 (145 to 225) | 1.25 (1 to 1.55) |  | 144 (112 to 186) | 0.59 (0.46 to 0.77) | -3.43 (-4.77 to -2.07) |
| Puerto Rico | 20 (16 to 26) | 0.56 (0.44 to 0.7) |  | 43 (33 to 57) | 0.65 (0.51 to 0.84) | 0.52 (0.48 to 0.57) |
| Qatar | 1 (1 to 1) | 0.44 (0.36 to 0.53) |  | 8 (6 to 11) | 0.62 (0.51 to 0.77) | 1.32 (0.9 to 1.74) |
| Republic of Korea | 2317 (1953 to 2800) | 8.33 (7.03 to 9.98) |  | 6701 (5537 to 8273) | 7.51 (6.23 to 9.26) | -0.69 (-1.28 to -0.1) |
| Republic of Moldova | 230 (182 to 290) | 5.22 (4.18 to 6.51) |  | 432 (343 to 547) | 7.68 (6.06 to 9.77) | 1.44 (1.33 to 1.54) |
| Romania | 3587 (2989 to 4332) | 12.73 (10.66 to 15.22) |  | 4059 (3255 to 4959) | 11.63 (9.35 to 14.3) | -0.49 (-0.74 to -0.24) |
| Russian Federation | 9361 (7623 to 11552) | 5.28 (4.33 to 6.5) |  | 19273 (15919 to 23343) | 8.47 (7 to 10.26) | 1.66 (1.42 to 1.91) |
| Rwanda | 100 (82 to 119) | 3.51 (2.88 to 4.19) |  | 177 (144 to 228) | 3.26 (2.65 to 4.17) | -0.3 (-0.51 to -0.09) |
| Saint Kitts and Nevis | 0 (0 to 0) | 0.68 (0.53 to 0.86) |  | 0 (0 to 1) | 0.68 (0.53 to 0.87) | -0.03 (-0.12 to 0.05) |
| Saint Lucia | 1 (1 to 1) | 0.86 (0.69 to 1.08) |  | 1 (1 to 2) | 0.71 (0.56 to 0.92) | -0.77 (-0.95 to -0.6) |
| Saint Vincent and the Grenadines | 1 (1 to 1) | 0.89 (0.71 to 1.11) |  | 1 (1 to 2) | 1.08 (0.86 to 1.38) | 0.42 (-0.02 to 0.86) |
| Samoa | 3 (3 to 4) | 3.92 (3.08 to 5.05) |  | 9 (7 to 11) | 5.92 (4.54 to 7.57) | 1.43 (1.33 to 1.54) |
| San Marino | 0 (0 to 0) | 0.12 (0.09 to 0.16) |  | 0 (0 to 0) | 0.11 (0.08 to 0.14) | -1 (-1.55 to -0.44) |
| Sao Tome and Principe | 0 (0 to 1) | 0.72 (0.6 to 0.86) |  | 1 (1 to 1) | 0.95 (0.8 to 1.12) | 0.91 (0.81 to 1.02) |
| Saudi Arabia | 29 (22 to 36) | 0.41 (0.34 to 0.51) |  | 130 (102 to 164) | 0.67 (0.55 to 0.82) | 1.63 (1.53 to 1.74) |
| Senegal | 36 (29 to 44) | 1 (0.83 to 1.2) |  | 93 (76 to 111) | 1.12 (0.94 to 1.33) | 0.41 (0.28 to 0.53) |
| Serbia | 556 (450 to 684) | 4.93 (4.01 to 6.04) |  | 1561 (1255 to 1923) | 10.38 (8.43 to 12.69) | 2.61 (2.54 to 2.69) |
| Seychelles | 1 (1 to 2) | 2.37 (1.77 to 3.14) |  | 5 (4 to 7) | 4.51 (3.25 to 6.04) | 2.3 (2.21 to 2.38) |
| Sierra Leone | 16 (14 to 20) | 0.81 (0.68 to 0.97) |  | 41 (34 to 49) | 1.03 (0.86 to 1.21) | 0.86 (0.79 to 0.93) |
| Singapore | 82 (68 to 101) | 4.18 (3.44 to 5.12) |  | 369 (294 to 463) | 5.02 (4.01 to 6.31) | 0.65 (0.54 to 0.75) |
| Slovakia | 678 (538 to 993) | 11.38 (9.09 to 16.45) |  | 1141 (907 to 1409) | 12.71 (10.18 to 15.74) | 0.29 (0.09 to 0.5) |
| Slovenia | 331 (267 to 421) | 13.61 (11.01 to 17.25) |  | 559 (451 to 692) | 14.58 (11.68 to 17.86) | 0.18 (0.02 to 0.34) |
| Solomon Islands | 6 (5 to 8) | 4.6 (3.67 to 5.84) |  | 19 (15 to 24) | 6.29 (4.96 to 7.93) | 1.11 (0.98 to 1.23) |
| Somalia | 80 (66 to 99) | 3.5 (2.86 to 4.35) |  | 217 (176 to 268) | 3.55 (2.88 to 4.42) | -0.04 (-0.44 to 0.37) |
| South Africa | 3812 (3247 to 4448) | 18.31 (15.6 to 21.23) |  | 9745 (8278 to 11372) | 21.5 (18.38 to 25.02) | 0.36 (0.06 to 0.66) |
| South Sudan | 63 (53 to 75) | 2.77 (2.32 to 3.29) |  | 101 (86 to 120) | 3.01 (2.56 to 3.53) | 0.23 (0.11 to 0.35) |
| Spain | 543 (424 to 691) | 0.96 (0.75 to 1.22) |  | 350 (279 to 433) | 0.31 (0.25 to 0.39) | -4.74 (-5.98 to -3.48) |
| Sri Lanka | 346 (264 to 454) | 2.92 (2.22 to 3.85) |  | 1171 (850 to 1567) | 4.51 (3.28 to 6) | 1.53 (1.49 to 1.58) |
| Sudan | 43 (33 to 55) | 0.42 (0.34 to 0.52) |  | 156 (123 to 194) | 0.72 (0.6 to 0.87) | 1.93 (1.81 to 2.05) |
| Suriname | 4 (3 to 5) | 1.59 (1.28 to 2) |  | 6 (5 to 8) | 1.05 (0.82 to 1.36) | -1.65 (-2.41 to -0.89) |
| Sweden | 126 (92 to 250) | 0.75 (0.57 to 1.42) |  | 70 (56 to 90) | 0.34 (0.28 to 0.43) | -2.77 (-3.65 to -1.89) |
| Switzerland | 59 (49 to 72) | 0.53 (0.44 to 0.64) |  | 43 (34 to 54) | 0.25 (0.19 to 0.31) | -2.1 (-2.85 to -1.34) |
| Syrian Arab Republic | 39 (30 to 49) | 0.61 (0.5 to 0.73) |  | 87 (70 to 106) | 0.75 (0.62 to 0.89) | 0.69 (0.49 to 0.89) |
| Taiwan (Province of China) | 6141 (5164 to 7288) | 38.09 (32.23 to 45) |  | 9756 (8614 to 11037) | 24.68 (21.78 to 27.91) | -2.17 (-3.1 to -1.23) |
| Tajikistan | 63 (49 to 80) | 2.31 (1.79 to 2.98) |  | 168 (131 to 213) | 3.83 (2.95 to 4.91) | 1.8 (1.67 to 1.93) |
| Thailand | 1106 (844 to 1467) | 2.75 (2.12 to 3.65) |  | 4828 (3563 to 6472) | 4.65 (3.46 to 6.21) | 1.75 (1.64 to 1.85) |
| Timor-Leste | 9 (6 to 12) | 2.66 (1.95 to 3.61) |  | 39 (28 to 51) | 4.66 (3.45 to 6.11) | 2.01 (1.92 to 2.09) |
| Togo | 16 (13 to 20) | 1.09 (0.91 to 1.3) |  | 42 (34 to 51) | 1.02 (0.86 to 1.21) | -0.24 (-0.32 to -0.16) |
| Tokelau | 0 (0 to 0) | 3.52 (2.78 to 4.47) |  | 0 (0 to 0) | 6.12 (4.69 to 7.88) | 2 (1.89 to 2.1) |
| Tonga | 2 (2 to 3) | 3.99 (3.17 to 5.12) |  | 5 (4 to 6) | 5.71 (4.45 to 7.25) | 1.27 (1.15 to 1.39) |
| Trinidad and Tobago | 5 (4 to 6) | 0.53 (0.41 to 0.68) |  | 11 (9 to 14) | 0.62 (0.48 to 0.8) | 0.51 (0.31 to 0.71) |
| Tunisia | 20 (16 to 25) | 0.4 (0.32 to 0.48) |  | 80 (67 to 97) | 0.69 (0.57 to 0.82) | 1.94 (1.78 to 2.09) |
| Turkey | 2051 (1469 to 2760) | 4.59 (3.44 to 6.02) |  | 3859 (3022 to 4822) | 4.32 (3.42 to 5.37) | -0.47 (-0.83 to -0.11) |
| Turkmenistan | 38 (29 to 49) | 2.08 (1.61 to 2.68) |  | 120 (95 to 152) | 3.37 (2.65 to 4.24) | 1.69 (1.54 to 1.83) |
| Tuvalu | 0 (0 to 0) | 3.24 (2.55 to 4.19) |  | 1 (0 to 1) | 5.62 (4.36 to 7.29) | 1.97 (1.86 to 2.08) |
| Uganda | 175 (145 to 208) | 2.87 (2.4 to 3.42) |  | 377 (317 to 444) | 2.96 (2.47 to 3.49) | -0.01 (-0.21 to 0.2) |
| Ukraine | 13678 (11147 to 17414) | 19.05 (15.61 to 24.17) |  | 6605 (5407 to 8069) | 8.99 (7.35 to 11.01) | -2.83 (-3.25 to -2.41) |
| United Arab Emirates | 4 (3 to 5) | 0.49 (0.4 to 0.59) |  | 38 (29 to 48) | 0.91 (0.76 to 1.09) | 2.08 (1.93 to 2.22) |
| United Kingdom | 6679 (5735 to 7803) | 7.26 (6.26 to 8.51) |  | 8777 (7587 to 10089) | 6.79 (5.89 to 7.75) | -0.51 (-0.73 to -0.29) |
| United Republic of Tanzania | 263 (218 to 314) | 2.5 (2.1 to 2.98) |  | 659 (553 to 787) | 2.88 (2.42 to 3.43) | 0.42 (0.33 to 0.52) |
| United States of America | 37580 (31804 to 44364) | 11.72 (9.94 to 13.83) |  | 46307 (40897 to 52301) | 8.34 (7.39 to 9.38) | -1.31 (-1.55 to -1.07) |
| United States Virgin Islands | 1 (1 to 1) | 0.86 (0.7 to 1.07) |  | 2 (1 to 2) | 1.02 (0.84 to 1.25) | 0.59 (0.39 to 0.8) |
| Uruguay | 78 (58 to 104) | 1.93 (1.46 to 2.55) |  | 196 (146 to 263) | 3.4 (2.52 to 4.56) | 1.96 (1.85 to 2.07) |
| Uzbekistan | 278 (224 to 343) | 2.55 (2.04 to 3.14) |  | 828 (655 to 1045) | 4.77 (3.83 to 5.93) | 2.13 (1.98 to 2.28) |
| Vanuatu | 3 (2 to 4) | 4.4 (3.49 to 5.63) |  | 11 (8 to 14) | 6.32 (4.97 to 8.11) | 1.33 (1.21 to 1.44) |
| Venezuela (Bolivarian Republic of) | 370 (300 to 455) | 3.41 (2.78 to 4.16) |  | 1235 (1008 to 1517) | 4.23 (3.46 to 5.18) | 0.73 (0.7 to 0.76) |
| Viet Nam | 927 (694 to 1245) | 2.22 (1.66 to 2.99) |  | 4071 (2986 to 5444) | 4.08 (3.01 to 5.41) | 2.17 (2.09 to 2.25) |
| Yemen | 27 (21 to 34) | 0.47 (0.39 to 0.57) |  | 176 (136 to 220) | 1.03 (0.84 to 1.22) | 2.79 (2.62 to 2.95) |
| Zambia | 89 (76 to 105) | 3.29 (2.81 to 3.88) |  | 240 (204 to 278) | 3.84 (3.27 to 4.46) | 0.47 (0.3 to 0.63) |
| Zimbabwe | 192 (154 to 241) | 4.91 (3.99 to 6.07) |  | 396 (327 to 471) | 6.05 (5.06 to 7.15) | 0.69 (0.58 to 0.8) |

AAPC: average annual percent change; UI: uncertainty interval; CI: confidence interval; SDI: Socio-demographic index.

Table S13. Mortality of other CRDs (1990/2019) for sexes, SDI and locations, with AAPC (1990-2019)

| GBD data | 1990 | |  | 2019 | | AAPC % (95% CI)  1990-2019 |
| --- | --- | --- | --- | --- | --- | --- |
|  | Cases (95% UI) | Age-standardised mortality per  100 000 population (95% UI) |  | Cases (95% UI) | Age-standardised mortality per  100 000 population (95% UI) |  |
| Global | 26107 (20542 to 32806) | 0.62 (0.49 to 0.76) |  | 39761 (31085 to 46581) | 0.5 (0.39 to 0.59) | -0.65 (-0.8 to -0.49) |
| **Sex** |  |  |  |  |  |  |
| Female | 9944 (6705 to 13920) | 0.44 (0.31 to 0.61) |  | 15059 (10342 to 18924) | 0.36 (0.25 to 0.45) | -0.6 (-0.75 to -0.45) |
| Male | 16163 (11763 to 20311) | 0.84 (0.62 to 1.04) |  | 24702 (18384 to 30308) | 0.66 (0.49 to 0.8) | -0.77 (-0.95 to -0.59) |
| **SDI** |  |  |  |  |  |  |
| High SDI | 2899 (2540 to 3909) | 0.31 (0.26 to 0.41) |  | 5191 (4005 to 6261) | 0.31 (0.24 to 0.37) | 0.16 (-0.03 to 0.34) |
| High-middle SDI | 6642 (3910 to 7632) | 0.71 (0.37 to 0.82) |  | 4960 (4314 to 6130) | 0.27 (0.24 to 0.34) | -3.25 (-3.42 to -3.08) |
| Middle SDI | 5768 (4639 to 8363) | 0.46 (0.38 to 0.67) |  | 10290 (7923 to 12113) | 0.45 (0.34 to 0.52) | -0.09 (-0.17 to 0) |
| Low-middle SDI | 7265 (4580 to 10413) | 1 (0.64 to 1.4) |  | 12552 (9054 to 15827) | 0.88 (0.63 to 1.1) | -0.42 (-0.46 to -0.38) |
| Low SDI | 3511 (1883 to 5709) | 1.02 (0.61 to 1.52) |  | 6732 (4636 to 8760) | 0.95 (0.65 to 1.2) | -0.28 (-0.38 to -0.19) |
| **Region** |  |  |  |  |  |  |
| High-income Asia Pacific | 437 (360 to 517) | 0.23 (0.19 to 0.28) |  | 625 (496 to 913) | 0.17 (0.14 to 0.23) | -1.18 (-1.28 to -1.08) |
| High-income North America | 1067 (931 to 1633) | 0.33 (0.29 to 0.5) |  | 2624 (1644 to 2925) | 0.47 (0.3 to 0.52) | 1.34 (1.09 to 1.6) |
| Western Europe | 4027 (1607 to 4625) | 0.72 (0.3 to 0.83) |  | 2087 (1742 to 3048) | 0.24 (0.21 to 0.35) | -3.7 (-3.94 to -3.45) |
| Australasia | 93 (67 to 107) | 0.43 (0.31 to 0.5) |  | 142 (114 to 176) | 0.34 (0.27 to 0.42) | -0.87 (-1.04 to -0.7) |
| Andean Latin America | 474 (255 to 775) | 1.27 (0.74 to 1.9) |  | 339 (253 to 455) | 0.57 (0.42 to 0.77) | -2.77 (-3.02 to -2.52) |
| Tropical Latin America | 679 (559 to 849) | 0.57 (0.48 to 0.74) |  | 1276 (957 to 1533) | 0.55 (0.42 to 0.66) | -0.08 (-0.21 to 0.05) |
| Central Latin America | 808 (629 to 934) | 0.61 (0.48 to 0.71) |  | 1576 (1051 to 1961) | 0.67 (0.44 to 0.84) | 0.37 (-0.08 to 0.82) |
| Southern Latin America | 410 (317 to 473) | 0.9 (0.68 to 1.04) |  | 546 (410 to 657) | 0.7 (0.55 to 0.84) | -0.85 (-0.99 to -0.7) |
| Caribbean | 216 (114 to 401) | 0.65 (0.38 to 1.12) |  | 306 (209 to 453) | 0.65 (0.43 to 0.99) | -0.02 (-0.14 to 0.11) |
| Central Europe | 391 (279 to 433) | 0.3 (0.21 to 0.34) |  | 248 (201 to 354) | 0.14 (0.11 to 0.2) | -2.65 (-2.87 to -2.43) |
| Eastern Europe | 1074 (532 to 1254) | 0.41 (0.2 to 0.48) |  | 422 (350 to 615) | 0.14 (0.12 to 0.21) | -3.68 (-4.21 to -3.15) |
| Central Asia | 574 (476 to 670) | 1.13 (0.87 to 1.32) |  | 645 (537 to 809) | 0.88 (0.68 to 1.06) | -0.77 (-0.98 to -0.56) |
| North Africa and Middle East | 695 (467 to 1273) | 0.28 (0.2 to 0.48) |  | 1500 (1104 to 1862) | 0.32 (0.23 to 0.39) | 0.39 (0.08 to 0.69) |
| South Asia | 7718 (4411 to 11651) | 1.2 (0.71 to 1.75) |  | 13636 (9340 to 17850) | 0.96 (0.67 to 1.24) | -0.76 (-0.89 to -0.64) |
| Southeast Asia | 1792 (1285 to 3248) | 0.52 (0.39 to 0.82) |  | 2688 (2086 to 4096) | 0.41 (0.32 to 0.62) | -0.8 (-0.86 to -0.73) |
| East Asia | 2553 (1754 to 4736) | 0.29 (0.2 to 0.57) |  | 4909 (3102 to 6110) | 0.29 (0.18 to 0.36) | 0 (-0.18 to 0.17) |
| Oceania | 144 (68 to 288) | 3.82 (1.75 to 6.91) |  | 363 (177 to 644) | 4.35 (2.08 to 7.04) | 0.45 (0.38 to 0.53) |
| Western Sub-Saharan Africa | 1454 (978 to 2099) | 1 (0.69 to 1.33) |  | 3416 (2313 to 4545) | 0.98 (0.67 to 1.28) | -0.02 (-0.17 to 0.12) |
| Eastern Sub-Saharan Africa | 914 (287 to 1764) | 0.67 (0.29 to 1.05) |  | 1508 (836 to 2191) | 0.56 (0.33 to 0.82) | -0.65 (-0.76 to -0.55) |
| Central Sub-Saharan Africa | 285 (105 to 571) | 0.8 (0.38 to 1.27) |  | 520 (283 to 797) | 0.69 (0.37 to 1.14) | -0.54 (-0.6 to -0.49) |
| Southern Sub-Saharan Africa | 303 (218 to 372) | 0.78 (0.58 to 0.94) |  | 386 (319 to 543) | 0.59 (0.49 to 0.82) | -0.95 (-1.12 to -0.77) |
| **Countries** |  |  |  |  |  |  |
| Afghanistan | 23 (8 to 69) | 0.28 (0.11 to 0.72) |  | 69 (38 to 133) | 0.35 (0.2 to 0.59) | 0.86 (0.78 to 0.95) |
| Albania | 34 (21 to 43) | 1.63 (0.83 to 2.14) |  | 26 (17 to 35) | 0.69 (0.48 to 0.94) | -3.18 (-4.17 to -2.18) |
| Algeria | 26 (12 to 69) | 0.17 (0.08 to 0.38) |  | 71 (50 to 102) | 0.22 (0.15 to 0.31) | 0.97 (0.86 to 1.08) |
| American Samoa | 1 (1 to 1) | 3.31 (2.36 to 4.96) |  | 1 (1 to 2) | 3 (2.26 to 4.05) | -0.47 (-0.82 to -0.11) |
| Andorra | 0 (0 to 0) | 0.45 (0.26 to 0.71) |  | 0 (0 to 1) | 0.34 (0.21 to 0.55) | -1.01 (-1.09 to -0.93) |
| Angola | 54 (14 to 146) | 0.76 (0.28 to 1.48) |  | 86 (46 to 134) | 0.49 (0.28 to 0.71) | -1.57 (-1.72 to -1.42) |
| Antigua and Barbuda | 0 (0 to 0) | 0.73 (0.58 to 0.86) |  | 1 (1 to 1) | 0.79 (0.62 to 1.02) | 0.18 (0.01 to 0.35) |
| Argentina | 348 (276 to 408) | 1.12 (0.86 to 1.31) |  | 495 (363 to 605) | 0.96 (0.73 to 1.15) | -0.53 (-0.69 to -0.36) |
| Armenia | 4 (3 to 5) | 0.15 (0.12 to 0.18) |  | 3 (2 to 4) | 0.08 (0.07 to 0.11) | -2.15 (-2.57 to -1.73) |
| Australia | 70 (53 to 85) | 0.39 (0.3 to 0.47) |  | 116 (93 to 147) | 0.32 (0.26 to 0.41) | -0.6 (-0.77 to -0.42) |
| Austria | 18 (15 to 25) | 0.18 (0.15 to 0.23) |  | 25 (20 to 35) | 0.16 (0.12 to 0.21) | -0.24 (-0.69 to 0.21) |
| Azerbaijan | 46 (36 to 65) | 0.74 (0.57 to 1.06) |  | 44 (30 to 72) | 0.45 (0.32 to 0.71) | -1.58 (-1.81 to -1.35) |
| Bahamas | 2 (1 to 2) | 0.9 (0.71 to 1.11) |  | 3 (3 to 5) | 0.88 (0.65 to 1.15) | -0.09 (-0.52 to 0.33) |
| Bahrain | 2 (1 to 2) | 0.54 (0.32 to 0.73) |  | 3 (2 to 5) | 0.28 (0.19 to 0.44) | -2.49 (-2.95 to -2.02) |
| Bangladesh | 725 (373 to 1173) | 1.19 (0.6 to 1.87) |  | 896 (542 to 1377) | 0.66 (0.4 to 1.01) | -1.99 (-2.16 to -1.83) |
| Barbados | 1 (1 to 1) | 0.36 (0.29 to 0.45) |  | 2 (2 to 3) | 0.47 (0.36 to 0.6) | 0.79 (0.5 to 1.08) |
| Belarus | 59 (24 to 79) | 0.48 (0.2 to 0.65) |  | 16 (11 to 24) | 0.12 (0.08 to 0.17) | -4.91 (-5.33 to -4.49) |
| Belgium | 50 (39 to 61) | 0.37 (0.29 to 0.44) |  | 66 (50 to 83) | 0.31 (0.24 to 0.39) | -0.56 (-1.35 to 0.24) |
| Belize | 1 (1 to 1) | 0.63 (0.48 to 0.78) |  | 2 (2 to 3) | 0.65 (0.51 to 0.84) | 0.12 (-0.21 to 0.45) |
| Benin | 44 (28 to 66) | 1.22 (0.8 to 1.74) |  | 112 (64 to 174) | 1.12 (0.67 to 1.7) | -0.28 (-0.4 to -0.17) |
| Bermuda | 0 (0 to 0) | 0.5 (0.34 to 0.62) |  | 0 (0 to 0) | 0.31 (0.24 to 0.43) | -1.64 (-1.94 to -1.34) |
| Bhutan | 4 (2 to 8) | 1.24 (0.63 to 2.2) |  | 6 (3 to 9) | 0.99 (0.58 to 1.55) | -0.75 (-0.84 to -0.67) |
| Bolivia (Plurinational State of) | 93 (40 to 210) | 1.52 (0.77 to 2.91) |  | 89 (60 to 131) | 0.89 (0.61 to 1.29) | -1.85 (-1.9 to -1.79) |
| Bosnia and Herzegovina | 5 (3 to 6) | 0.12 (0.07 to 0.16) |  | 4 (2 to 7) | 0.09 (0.05 to 0.12) | -1.2 (-1.53 to -0.86) |
| Botswana | 8 (5 to 12) | 1.02 (0.65 to 1.5) |  | 16 (10 to 25) | 0.86 (0.57 to 1.3) | -0.57 (-0.7 to -0.43) |
| Brazil | 669 (551 to 842) | 0.58 (0.48 to 0.75) |  | 1263 (939 to 1525) | 0.56 (0.42 to 0.67) | -0.07 (-0.2 to 0.07) |
| Brunei Darussalam | 2 (1 to 3) | 1.48 (0.99 to 2.14) |  | 3 (2 to 4) | 0.89 (0.68 to 1.2) | -1.7 (-1.79 to -1.61) |
| Bulgaria | 37 (30 to 56) | 0.36 (0.3 to 0.54) |  | 32 (22 to 54) | 0.28 (0.2 to 0.48) | -0.67 (-1.17 to -0.17) |
| Burkina Faso | 61 (32 to 108) | 0.72 (0.43 to 1.1) |  | 177 (93 to 303) | 0.85 (0.5 to 1.28) | 0.63 (0.33 to 0.94) |
| Burundi | 44 (10 to 102) | 0.99 (0.33 to 1.95) |  | 57 (25 to 99) | 0.72 (0.36 to 1.19) | -1.12 (-1.53 to -0.7) |
| Cabo Verde | 6 (3 to 8) | 2.33 (1.35 to 3.08) |  | 5 (4 to 7) | 0.92 (0.66 to 1.31) | -3.09 (-3.35 to -2.83) |
| Cambodia | 22 (10 to 59) | 0.31 (0.17 to 0.67) |  | 42 (27 to 70) | 0.31 (0.21 to 0.52) | 0.01 (-0.2 to 0.22) |
| Cameroon | 95 (64 to 139) | 1.28 (0.81 to 1.9) |  | 264 (164 to 385) | 1.16 (0.71 to 1.69) | -0.3 (-0.52 to -0.07) |
| Canada | 89 (69 to 111) | 0.29 (0.23 to 0.37) |  | 171 (114 to 210) | 0.28 (0.2 to 0.34) | -0.18 (-0.32 to -0.04) |
| Central African Republic | 16 (5 to 31) | 0.91 (0.37 to 1.55) |  | 26 (11 to 50) | 0.8 (0.39 to 1.34) | -0.47 (-0.54 to -0.4) |
| Chad | 52 (32 to 81) | 1.12 (0.7 to 1.64) |  | 156 (96 to 239) | 1.27 (0.77 to 1.87) | 0.46 (0.31 to 0.6) |
| Chile | 35 (25 to 41) | 0.31 (0.22 to 0.36) |  | 38 (30 to 50) | 0.18 (0.14 to 0.24) | -1.96 (-2.08 to -1.84) |
| China | 2398 (1609 to 4556) | 0.29 (0.19 to 0.57) |  | 4666 (2892 to 5856) | 0.29 (0.18 to 0.36) | 0.07 (-0.11 to 0.25) |
| Colombia | 121 (103 to 170) | 0.54 (0.45 to 0.71) |  | 596 (224 to 850) | 1.11 (0.45 to 1.58) | 3.43 (2.22 to 4.65) |
| Comoros | 2 (1 to 4) | 0.61 (0.24 to 1.02) |  | 3 (1 to 4) | 0.49 (0.25 to 0.76) | -0.97 (-2.13 to 0.21) |
| Congo | 12 (5 to 22) | 0.86 (0.39 to 1.39) |  | 19 (11 to 29) | 0.54 (0.32 to 0.83) | -1.45 (-1.75 to -1.16) |
| Cook Islands | 0 (0 to 0) | 1.87 (1.12 to 3.45) |  | 0 (0 to 0) | 1.2 (0.77 to 1.96) | -1.42 (-1.75 to -1.1) |
| Costa Rica | 3 (3 to 5) | 0.13 (0.11 to 0.2) |  | 12 (7 to 16) | 0.23 (0.15 to 0.31) | 2.25 (1.17 to 3.34) |
| Croatia | 8 (6 to 10) | 0.15 (0.1 to 0.17) |  | 4 (3 to 7) | 0.06 (0.05 to 0.1) | -2.8 (-3.33 to -2.25) |
| Cuba | 26 (17 to 31) | 0.25 (0.17 to 0.29) |  | 27 (19 to 36) | 0.17 (0.12 to 0.23) | -1.24 (-1.54 to -0.95) |
| Cyprus | 4 (3 to 6) | 0.56 (0.38 to 0.83) |  | 6 (5 to 8) | 0.37 (0.28 to 0.47) | -1.47 (-1.67 to -1.27) |
| Czechia | 19 (15 to 23) | 0.16 (0.13 to 0.19) |  | 22 (14 to 29) | 0.13 (0.09 to 0.18) | -0.46 (-0.87 to -0.05) |
| Côte d’Ivoire | 107 (67 to 155) | 1.29 (0.84 to 1.86) |  | 226 (136 to 336) | 1.08 (0.67 to 1.62) | -0.68 (-0.85 to -0.5) |
| Democratic People's Republic of Korea | 82 (53 to 127) | 0.51 (0.33 to 0.83) |  | 143 (93 to 212) | 0.5 (0.32 to 0.75) | -0.04 (-0.14 to 0.06) |
| Democratic Republic of the Congo | 196 (75 to 403) | 0.8 (0.39 to 1.34) |  | 380 (202 to 612) | 0.76 (0.38 to 1.34) | -0.22 (-0.3 to -0.14) |
| Denmark | 8 (7 to 12) | 0.12 (0.1 to 0.16) |  | 14 (10 to 17) | 0.14 (0.11 to 0.17) | 0.38 (0.06 to 0.71) |
| Djibouti | 2 (1 to 3) | 0.5 (0.23 to 0.8) |  | 3 (2 to 6) | 0.39 (0.2 to 0.67) | -0.89 (-1.17 to -0.62) |
| Dominica | 1 (0 to 1) | 0.88 (0.66 to 1.14) |  | 1 (1 to 1) | 1.07 (0.73 to 1.5) | 0.67 (0.53 to 0.81) |
| Dominican Republic | 29 (14 to 48) | 0.41 (0.24 to 0.62) |  | 40 (24 to 59) | 0.4 (0.23 to 0.57) | 0.13 (-0.32 to 0.58) |
| Ecuador | 75 (46 to 110) | 0.81 (0.52 to 1.11) |  | 61 (46 to 89) | 0.39 (0.3 to 0.56) | -2.69 (-3.04 to -2.34) |
| Egypt | 88 (38 to 227) | 0.2 (0.1 to 0.47) |  | 205 (134 to 315) | 0.28 (0.18 to 0.44) | 1.26 (0.81 to 1.71) |
| El Salvador | 49 (25 to 77) | 0.93 (0.48 to 1.37) |  | 17 (12 to 25) | 0.28 (0.19 to 0.41) | -4.33 (-4.74 to -3.92) |
| Equatorial Guinea | 2 (1 to 5) | 0.78 (0.32 to 1.39) |  | 4 (2 to 7) | 0.54 (0.27 to 1.02) | -1.29 (-1.57 to -1.01) |
| Eritrea | 16 (4 to 37) | 0.72 (0.28 to 1.37) |  | 26 (12 to 44) | 0.57 (0.29 to 0.9) | -0.81 (-0.95 to -0.67) |
| Estonia | 3 (2 to 4) | 0.18 (0.11 to 0.23) |  | 1 (1 to 2) | 0.08 (0.05 to 0.11) | -3.29 (-3.9 to -2.67) |
| Eswatini | 5 (3 to 7) | 1.05 (0.71 to 1.5) |  | 8 (5 to 11) | 0.91 (0.6 to 1.35) | -0.49 (-0.64 to -0.34) |
| Ethiopia | 252 (61 to 576) | 0.7 (0.23 to 1.36) |  | 308 (173 to 497) | 0.47 (0.27 to 0.81) | -1.41 (-1.47 to -1.34) |
| Fiji | 11 (8 to 15) | 2.42 (1.69 to 3.27) |  | 15 (11 to 21) | 1.95 (1.4 to 2.74) | -0.84 (-1.19 to -0.49) |
| Finland | 10 (7 to 13) | 0.16 (0.11 to 0.19) |  | 18 (10 to 22) | 0.16 (0.09 to 0.19) | 0.29 (-0.45 to 1.04) |
| France | 380 (308 to 482) | 0.51 (0.4 to 0.62) |  | 493 (389 to 733) | 0.39 (0.32 to 0.54) | -0.96 (-1.08 to -0.83) |
| Gabon | 4 (2 to 7) | 0.64 (0.3 to 0.99) |  | 5 (3 to 9) | 0.43 (0.26 to 0.68) | -1.44 (-1.57 to -1.32) |
| Gambia | 8 (5 to 11) | 1.12 (0.69 to 1.69) |  | 20 (13 to 30) | 1.19 (0.74 to 1.76) | 0.01 (-1.42 to 1.47) |
| Georgia | 35 (27 to 44) | 0.59 (0.45 to 0.74) |  | 34 (25 to 44) | 0.66 (0.5 to 0.85) | 0.39 (-0.52 to 1.31) |
| Germany | 218 (171 to 298) | 0.19 (0.15 to 0.26) |  | 292 (220 to 360) | 0.17 (0.13 to 0.21) | -0.49 (-0.81 to -0.17) |
| Ghana | 127 (72 to 186) | 1.17 (0.63 to 1.75) |  | 401 (153 to 604) | 1.58 (0.59 to 2.41) | 1.11 (0.97 to 1.26) |
| Greece | 16 (9 to 20) | 0.12 (0.07 to 0.14) |  | 11 (9 to 19) | 0.06 (0.05 to 0.09) | -2.71 (-3.48 to -1.93) |
| Greenland | 0 (0 to 0) | 0.18 (0.13 to 0.27) |  | 0 (0 to 0) | 0.18 (0.11 to 0.25) | -0.03 (-0.32 to 0.27) |
| Grenada | 1 (1 to 1) | 1.36 (1.05 to 1.7) |  | 1 (1 to 2) | 1.21 (0.98 to 1.51) | -0.54 (-0.87 to -0.21) |
| Guam | 1 (1 to 1) | 0.9 (0.64 to 1.42) |  | 2 (1 to 2) | 0.84 (0.63 to 1.18) | -0.46 (-0.99 to 0.07) |
| Guatemala | 129 (74 to 180) | 1.77 (1.05 to 2.3) |  | 93 (69 to 129) | 0.62 (0.46 to 0.88) | -3.62 (-4.49 to -2.75) |
| Guinea | 64 (38 to 104) | 1.21 (0.76 to 1.75) |  | 137 (81 to 215) | 1.33 (0.8 to 2) | 0.42 (0.27 to 0.57) |
| Guinea-Bissau | 12 (6 to 20) | 1.68 (0.89 to 2.68) |  | 21 (13 to 33) | 1.51 (0.91 to 2.38) | -0.35 (-0.45 to -0.25) |
| Guyana | 3 (2 to 4) | 0.68 (0.41 to 0.86) |  | 4 (3 to 6) | 0.58 (0.42 to 0.79) | -0.45 (-0.64 to -0.26) |
| Haiti | 109 (33 to 272) | 1.83 (0.77 to 3.71) |  | 165 (82 to 300) | 1.59 (0.87 to 2.68) | -0.47 (-0.56 to -0.38) |
| Honduras | 65 (26 to 115) | 1.3 (0.59 to 1.99) |  | 74 (35 to 119) | 0.95 (0.46 to 1.56) | -1.04 (-1.13 to -0.96) |
| Hungary | 61 (36 to 72) | 0.49 (0.29 to 0.58) |  | 41 (29 to 54) | 0.29 (0.2 to 0.38) | -2.14 (-2.71 to -1.56) |
| Iceland | 1 (0 to 1) | 0.29 (0.11 to 0.36) |  | 0 (0 to 1) | 0.09 (0.07 to 0.12) | -4.33 (-4.86 to -3.81) |
| India | 6142 (3578 to 9378) | 1.24 (0.75 to 1.83) |  | 11229 (7540 to 14710) | 0.99 (0.67 to 1.29) | -0.77 (-0.92 to -0.62) |
| Indonesia | 679 (386 to 1534) | 0.48 (0.29 to 0.97) |  | 1101 (737 to 1923) | 0.45 (0.3 to 0.78) | -0.2 (-0.27 to -0.13) |
| Iran (Islamic Republic of) | 53 (35 to 102) | 0.16 (0.12 to 0.23) |  | 233 (78 to 329) | 0.33 (0.1 to 0.47) | 2.52 (1.77 to 3.27) |
| Iraq | 96 (61 to 140) | 0.83 (0.54 to 1.15) |  | 227 (165 to 304) | 0.8 (0.58 to 1.09) | -0.2 (-0.35 to -0.04) |
| Ireland | 23 (19 to 28) | 0.61 (0.49 to 0.74) |  | 23 (17 to 40) | 0.34 (0.26 to 0.57) | -2.03 (-2.39 to -1.68) |
| Israel | 16 (13 to 23) | 0.33 (0.27 to 0.49) |  | 25 (20 to 37) | 0.23 (0.19 to 0.33) | -1.44 (-2.81 to -0.05) |
| Italy | 1144 (251 to 1362) | 1.35 (0.31 to 1.62) |  | 244 (179 to 484) | 0.16 (0.12 to 0.35) | -7.43 (-8.36 to -6.5) |
| Jamaica | 9 (6 to 12) | 0.43 (0.31 to 0.53) |  | 14 (10 to 19) | 0.46 (0.34 to 0.64) | 0.27 (-0.2 to 0.74) |
| Japan | 326 (273 to 433) | 0.22 (0.18 to 0.29) |  | 520 (401 to 794) | 0.18 (0.16 to 0.27) | -0.64 (-0.72 to -0.55) |
| Jordan | 2 (1 to 2) | 0.08 (0.05 to 0.11) |  | 5 (3 to 6) | 0.06 (0.04 to 0.08) | -0.78 (-1.05 to -0.5) |
| Kazakhstan | 145 (103 to 179) | 1.24 (0.77 to 1.61) |  | 210 (138 to 285) | 1.43 (0.77 to 2.06) | 0.97 (0.32 to 1.64) |
| Kenya | 74 (33 to 117) | 0.55 (0.28 to 0.95) |  | 193 (110 to 317) | 0.6 (0.35 to 1.03) | 0.3 (0.18 to 0.42) |
| Kiribati | 3 (2 to 5) | 5.72 (3.62 to 9.82) |  | 4 (2 to 6) | 4.72 (3.02 to 7.34) | -0.69 (-0.83 to -0.56) |
| Kuwait | 1 (1 to 2) | 0.12 (0.09 to 0.15) |  | 3 (3 to 5) | 0.11 (0.08 to 0.14) | -0.39 (-1.56 to 0.78) |
| Kyrgyzstan | 14 (6 to 18) | 0.39 (0.16 to 0.49) |  | 10 (7 to 13) | 0.16 (0.12 to 0.21) | -3.43 (-4.18 to -2.67) |
| Lao People's Democratic Republic | 21 (8 to 57) | 0.66 (0.31 to 1.55) |  | 30 (17 to 54) | 0.53 (0.32 to 0.92) | -0.82 (-0.87 to -0.78) |
| Latvia | 6 (5 to 9) | 0.18 (0.15 to 0.28) |  | 4 (3 to 6) | 0.14 (0.1 to 0.19) | -1.17 (-2.45 to 0.12) |
| Lebanon | 3 (2 to 7) | 0.14 (0.08 to 0.28) |  | 10 (6 to 16) | 0.19 (0.12 to 0.3) | 0.96 (0.87 to 1.05) |
| Lesotho | 14 (8 to 23) | 1.1 (0.63 to 1.83) |  | 18 (10 to 31) | 1.14 (0.62 to 1.9) | 0.16 (0 to 0.32) |
| Liberia | 17 (9 to 33) | 0.89 (0.54 to 1.44) |  | 34 (19 to 61) | 0.8 (0.46 to 1.3) | -0.55 (-0.77 to -0.32) |
| Libya | 4 (2 to 8) | 0.14 (0.07 to 0.28) |  | 12 (8 to 18) | 0.21 (0.14 to 0.31) | 1.52 (1.31 to 1.73) |
| Lithuania | 12 (6 to 15) | 0.29 (0.15 to 0.36) |  | 5 (4 to 7) | 0.11 (0.08 to 0.16) | -3.24 (-3.89 to -2.58) |
| Luxembourg | 1 (0 to 1) | 0.2 (0.1 to 0.24) |  | 1 (0 to 1) | 0.07 (0.05 to 0.12) | -3.66 (-3.99 to -3.32) |
| Madagascar | 106 (35 to 199) | 1.05 (0.47 to 1.59) |  | 180 (97 to 300) | 0.91 (0.52 to 1.41) | -0.57 (-0.64 to -0.5) |
| Malawi | 38 (12 to 80) | 0.53 (0.24 to 0.85) |  | 57 (30 to 89) | 0.49 (0.27 to 0.81) | -0.26 (-0.32 to -0.19) |
| Malaysia | 65 (42 to 89) | 0.52 (0.32 to 0.7) |  | 141 (96 to 196) | 0.48 (0.33 to 0.66) | -0.24 (-0.53 to 0.05) |
| Maldives | 3 (1 to 7) | 1.51 (0.71 to 2.98) |  | 2 (2 to 3) | 0.49 (0.37 to 0.67) | -3.87 (-4.42 to -3.31) |
| Mali | 85 (51 to 128) | 1.45 (0.85 to 2.14) |  | 229 (134 to 348) | 1.63 (0.85 to 2.57) | 0.39 (0.08 to 0.69) |
| Malta | 2 (1 to 2) | 0.4 (0.19 to 0.5) |  | 1 (1 to 2) | 0.12 (0.09 to 0.2) | -3.99 (-4.27 to -3.72) |
| Marshall Islands | 1 (0 to 1) | 3.48 (1.75 to 6.26) |  | 1 (1 to 2) | 3.21 (1.83 to 5.35) | -0.38 (-0.56 to -0.19) |
| Mauritania | 15 (9 to 22) | 0.98 (0.57 to 1.42) |  | 20 (12 to 30) | 0.65 (0.38 to 0.97) | -1.35 (-1.61 to -1.08) |
| Mauritius | 9 (6 to 10) | 0.96 (0.69 to 1.15) |  | 9 (7 to 15) | 0.66 (0.47 to 1.02) | -1.11 (-1.84 to -0.38) |
| Mexico | 396 (334 to 515) | 0.62 (0.53 to 0.81) |  | 712 (576 to 908) | 0.61 (0.49 to 0.76) | 0.16 (-0.28 to 0.6) |
| Micronesia (Federated States of) | 2 (1 to 5) | 3.95 (1.97 to 7.25) |  | 3 (1 to 4) | 3.31 (1.91 to 5.48) | -0.62 (-0.63 to -0.61) |
| Monaco | 0 (0 to 1) | 0.63 (0.4 to 0.88) |  | 1 (0 to 1) | 0.59 (0.37 to 0.81) | -0.16 (-0.24 to -0.07) |
| Mongolia | 20 (12 to 29) | 1.03 (0.71 to 1.39) |  | 13 (9 to 23) | 0.49 (0.34 to 0.72) | -2.6 (-2.72 to -2.48) |
| Montenegro | 1 (1 to 1) | 0.15 (0.11 to 0.19) |  | 1 (1 to 1) | 0.12 (0.09 to 0.17) | -0.47 (-0.53 to -0.4) |
| Morocco | 22 (9 to 57) | 0.14 (0.06 to 0.3) |  | 71 (49 to 101) | 0.24 (0.17 to 0.34) | 1.9 (1.63 to 2.18) |
| Mozambique | 43 (14 to 105) | 0.42 (0.19 to 0.73) |  | 84 (41 to 143) | 0.44 (0.24 to 0.7) | 0.21 (-0.12 to 0.54) |
| Myanmar | 251 (115 to 657) | 0.83 (0.43 to 1.77) |  | 364 (234 to 618) | 0.74 (0.48 to 1.23) | -0.4 (-0.51 to -0.29) |
| Namibia | 9 (6 to 14) | 1.04 (0.68 to 1.57) |  | 16 (10 to 24) | 0.89 (0.58 to 1.32) | -0.37 (-0.58 to -0.16) |
| Nauru | 0 (0 to 0) | 3.89 (1.9 to 7.7) |  | 0 (0 to 0) | 3.18 (1.87 to 5.28) | -0.7 (-0.96 to -0.44) |
| Nepal | 191 (87 to 354) | 1.83 (0.89 to 3.25) |  | 372 (206 to 549) | 1.73 (0.95 to 2.59) | -0.16 (-0.22 to -0.09) |
| Netherlands | 40 (34 to 53) | 0.22 (0.18 to 0.29) |  | 68 (49 to 84) | 0.22 (0.16 to 0.27) | -0.16 (-0.65 to 0.33) |
| New Zealand | 23 (14 to 26) | 0.65 (0.39 to 0.75) |  | 26 (20 to 31) | 0.43 (0.32 to 0.5) | -1.61 (-1.96 to -1.26) |
| Nicaragua | 5 (2 to 10) | 0.13 (0.09 to 0.2) |  | 13 (6 to 21) | 0.22 (0.11 to 0.34) | 2.11 (1.78 to 2.45) |
| Niger | 84 (42 to 163) | 1.31 (0.72 to 2.05) |  | 210 (110 to 364) | 1.15 (0.64 to 1.8) | -0.4 (-0.62 to -0.18) |
| Nigeria | 546 (333 to 880) | 0.79 (0.49 to 1.15) |  | 1113 (721 to 1596) | 0.7 (0.46 to 1) | -0.46 (-0.65 to -0.28) |
| Niue | 0 (0 to 0) | 2.61 (1.52 to 4.65) |  | 0 (0 to 0) | 2.19 (1.37 to 3.46) | -0.64 (-0.71 to -0.57) |
| North Macedonia | 3 (1 to 3) | 0.14 (0.08 to 0.19) |  | 2 (1 to 3) | 0.07 (0.05 to 0.11) | -2.64 (-3.26 to -2.01) |
| Northern Mariana Islands | 0 (0 to 1) | 1.11 (0.64 to 2.06) |  | 1 (0 to 1) | 1.23 (0.88 to 1.77) | 0.2 (-0.21 to 0.61) |
| Norway | 6 (5 to 9) | 0.09 (0.08 to 0.14) |  | 10 (7 to 12) | 0.11 (0.08 to 0.13) | 0.77 (0.44 to 1.11) |
| Oman | 12 (3 to 20) | 0.96 (0.29 to 1.7) |  | 18 (5 to 30) | 0.47 (0.19 to 0.76) | -2.33 (-3.11 to -1.54) |
| Pakistan | 657 (360 to 994) | 1 (0.55 to 1.43) |  | 1133 (722 to 1720) | 0.85 (0.55 to 1.26) | -0.5 (-0.59 to -0.41) |
| Palau | 2 (2 to 3) | 20.3 (15.28 to 27.69) |  | 3 (2 to 4) | 15.73 (11.64 to 21.14) | -0.88 (-1.02 to -0.74) |
| Palestine | 3 (2 to 5) | 0.29 (0.13 to 0.47) |  | 15 (4 to 25) | 0.77 (0.12 to 1.34) | 3.7 (2.73 to 4.67) |
| Panama | 6 (4 to 8) | 0.28 (0.22 to 0.37) |  | 14 (10 to 19) | 0.33 (0.23 to 0.45) | 0.59 (-0.07 to 1.25) |
| Papua New Guinea | 100 (36 to 218) | 4.26 (1.49 to 8.25) |  | 287 (118 to 532) | 5.11 (2.02 to 8.48) | 0.64 (0.53 to 0.75) |
| Paraguay | 11 (6 to 16) | 0.27 (0.18 to 0.36) |  | 13 (8 to 18) | 0.2 (0.13 to 0.27) | -0.58 (-1.31 to 0.16) |
| Peru | 306 (161 to 498) | 1.41 (0.8 to 2.1) |  | 189 (128 to 271) | 0.56 (0.38 to 0.8) | -3.24 (-3.7 to -2.77) |
| Philippines | 153 (107 to 217) | 0.37 (0.27 to 0.47) |  | 278 (213 to 364) | 0.29 (0.22 to 0.38) | -0.72 (-0.86 to -0.57) |
| Poland | 123 (74 to 136) | 0.32 (0.19 to 0.36) |  | 51 (35 to 119) | 0.09 (0.06 to 0.21) | -4.48 (-5.18 to -3.77) |
| Portugal | 215 (39 to 282) | 1.84 (0.31 to 2.44) |  | 62 (48 to 81) | 0.25 (0.2 to 0.36) | -6.53 (-7.83 to -5.21) |
| Puerto Rico | 16 (11 to 19) | 0.45 (0.32 to 0.53) |  | 18 (13 to 26) | 0.34 (0.25 to 0.48) | -1.07 (-1.57 to -0.56) |
| Qatar | 1 (1 to 1) | 0.66 (0.31 to 1.04) |  | 5 (3 to 7) | 0.59 (0.28 to 0.97) | -0.31 (-1.16 to 0.55) |
| Republic of Korea | 97 (47 to 144) | 0.26 (0.13 to 0.39) |  | 88 (67 to 132) | 0.11 (0.08 to 0.16) | -3.12 (-3.48 to -2.76) |
| Republic of Moldova | 6 (5 to 8) | 0.14 (0.11 to 0.17) |  | 5 (4 to 7) | 0.1 (0.07 to 0.14) | -1.04 (-1.3 to -0.78) |
| Romania | 66 (33 to 88) | 0.28 (0.13 to 0.37) |  | 24 (18 to 34) | 0.08 (0.06 to 0.11) | -4.28 (-4.79 to -3.77) |
| Russian Federation | 698 (267 to 829) | 0.41 (0.16 to 0.48) |  | 292 (234 to 418) | 0.14 (0.12 to 0.21) | -4.05 (-4.64 to -3.45) |
| Rwanda | 56 (14 to 130) | 1.02 (0.34 to 1.99) |  | 65 (34 to 121) | 0.7 (0.39 to 1.27) | -1.4 (-1.74 to -1.06) |
| Saint Kitts and Nevis | 1 (0 to 1) | 1.51 (1.08 to 1.87) |  | 1 (0 to 1) | 1.11 (0.8 to 1.49) | -0.84 (-1.2 to -0.49) |
| Saint Lucia | 1 (1 to 1) | 0.95 (0.72 to 1.13) |  | 2 (1 to 2) | 0.92 (0.71 to 1.22) | -0.12 (-0.38 to 0.13) |
| Saint Vincent and the Grenadines | 0 (0 to 1) | 0.56 (0.38 to 0.69) |  | 1 (1 to 1) | 0.57 (0.44 to 0.73) | 0.1 (-0.08 to 0.27) |
| Samoa | 4 (2 to 7) | 3.41 (1.93 to 6.38) |  | 4 (3 to 7) | 2.6 (1.74 to 4.06) | -0.93 (-0.96 to -0.9) |
| San Marino | 0 (0 to 0) | 0.97 (0.64 to 1.33) |  | 1 (0 to 1) | 0.87 (0.53 to 1.36) | -0.25 (-0.72 to 0.22) |
| Sao Tome and Principe | 2 (1 to 3) | 1.96 (1.21 to 2.86) |  | 4 (2 to 6) | 2.22 (1.4 to 3.38) | 0.52 (0.3 to 0.74) |
| Saudi Arabia | 26 (16 to 37) | 0.3 (0.2 to 0.42) |  | 69 (41 to 102) | 0.25 (0.16 to 0.35) | -0.66 (-0.85 to -0.47) |
| Senegal | 66 (42 to 99) | 1.17 (0.75 to 1.72) |  | 127 (78 to 180) | 1.05 (0.65 to 1.5) | -0.4 (-1.32 to 0.53) |
| Serbia | 16 (12 to 26) | 0.15 (0.11 to 0.25) |  | 26 (14 to 39) | 0.2 (0.11 to 0.29) | 0.96 (0.66 to 1.25) |
| Seychelles | 0 (0 to 0) | 0.3 (0.18 to 0.59) |  | 0 (0 to 1) | 0.28 (0.17 to 0.5) | -0.23 (-0.42 to -0.05) |
| Sierra Leone | 35 (20 to 58) | 1.15 (0.69 to 1.72) |  | 82 (49 to 127) | 1.2 (0.71 to 1.82) | 0.17 (0 to 0.34) |
| Singapore | 12 (6 to 14) | 0.47 (0.24 to 0.56) |  | 14 (11 to 20) | 0.19 (0.15 to 0.26) | -2.81 (-3.47 to -2.15) |
| Slovakia | 15 (11 to 22) | 0.28 (0.19 to 0.39) |  | 13 (8 to 22) | 0.17 (0.11 to 0.28) | -1.47 (-1.89 to -1.06) |
| Slovenia | 3 (2 to 4) | 0.12 (0.08 to 0.16) |  | 2 (2 to 3) | 0.06 (0.04 to 0.09) | -1.99 (-2.74 to -1.22) |
| Solomon Islands | 6 (3 to 12) | 3.42 (1.59 to 6.32) |  | 15 (9 to 25) | 3.87 (2.38 to 6.07) | 0.45 (0.3 to 0.6) |
| Somalia | 40 (11 to 94) | 0.79 (0.32 to 1.54) |  | 81 (30 to 175) | 0.65 (0.31 to 1.2) | -0.64 (-0.75 to -0.53) |
| South Africa | 228 (150 to 281) | 0.77 (0.53 to 0.92) |  | 265 (213 to 379) | 0.54 (0.44 to 0.75) | -1.21 (-1.46 to -0.97) |
| South Sudan | 29 (9 to 62) | 0.66 (0.3 to 1.09) |  | 30 (15 to 51) | 0.49 (0.26 to 0.78) | -1.03 (-1.2 to -0.86) |
| Spain | 1524 (264 to 1888) | 2.87 (0.54 to 3.55) |  | 296 (219 to 510) | 0.29 (0.21 to 0.52) | -7.82 (-8.48 to -7.15) |
| Sri Lanka | 181 (106 to 260) | 1.23 (0.75 to 1.76) |  | 178 (107 to 266) | 0.74 (0.47 to 1.08) | -1.6 (-2.05 to -1.14) |
| Sudan | 34 (12 to 101) | 0.24 (0.11 to 0.52) |  | 80 (50 to 122) | 0.32 (0.19 to 0.48) | 1.02 (0.91 to 1.14) |
| Suriname | 5 (3 to 6) | 1.46 (1.06 to 1.83) |  | 8 (6 to 10) | 1.31 (1 to 1.74) | -0.69 (-0.82 to -0.55) |
| Sweden | 19 (15 to 25) | 0.14 (0.11 to 0.18) |  | 32 (21 to 39) | 0.15 (0.11 to 0.18) | 0.29 (-0.08 to 0.67) |
| Switzerland | 22 (18 to 30) | 0.23 (0.19 to 0.31) |  | 37 (28 to 48) | 0.22 (0.17 to 0.28) | -0.22 (-0.56 to 0.12) |
| Syrian Arab Republic | 12 (6 to 21) | 0.17 (0.08 to 0.29) |  | 22 (12 to 32) | 0.26 (0.1 to 0.45) | 1.38 (0.93 to 1.84) |
| Taiwan (Province of China) | 73 (46 to 85) | 0.46 (0.28 to 0.54) |  | 101 (72 to 148) | 0.28 (0.2 to 0.42) | -1.59 (-2.25 to -0.92) |
| Tajikistan | 113 (85 to 146) | 3.48 (2.43 to 4.75) |  | 141 (103 to 196) | 2.69 (1.77 to 3.67) | -0.83 (-1.09 to -0.57) |
| Thailand | 240 (147 to 322) | 0.52 (0.32 to 0.69) |  | 227 (155 to 364) | 0.27 (0.19 to 0.43) | -2.48 (-3.12 to -1.83) |
| Timor-Leste | 3 (1 to 8) | 0.52 (0.26 to 1.06) |  | 5 (3 to 9) | 0.52 (0.31 to 0.91) | -0.03 (-0.39 to 0.34) |
| Togo | 30 (20 to 44) | 1.13 (0.77 to 1.6) |  | 76 (48 to 111) | 1.16 (0.72 to 1.73) | 0.1 (0 to 0.21) |
| Tokelau | 0 (0 to 0) | 2.58 (1.31 to 4.83) |  | 0 (0 to 0) | 2.06 (1.32 to 3.46) | -0.72 (-0.79 to -0.65) |
| Tonga | 1 (1 to 2) | 2.02 (1.01 to 3.85) |  | 2 (1 to 3) | 1.99 (1.21 to 3.38) | -0.03 (-0.37 to 0.3) |
| Trinidad and Tobago | 3 (2 to 4) | 0.29 (0.22 to 0.35) |  | 5 (3 to 6) | 0.28 (0.19 to 0.4) | 0.2 (-0.36 to 0.77) |
| Tunisia | 8 (4 to 17) | 0.13 (0.08 to 0.27) |  | 22 (13 to 34) | 0.19 (0.11 to 0.29) | 1.23 (1.07 to 1.39) |
| Turkey | 256 (164 to 411) | 0.5 (0.33 to 0.79) |  | 253 (190 to 356) | 0.3 (0.23 to 0.43) | -1.69 (-1.82 to -1.56) |
| Turkmenistan | 19 (13 to 24) | 0.68 (0.49 to 0.88) |  | 11 (7 to 20) | 0.22 (0.15 to 0.4) | -4.35 (-5.17 to -3.52) |
| Tuvalu | 0 (0 to 1) | 3.76 (1.9 to 6.78) |  | 0 (0 to 0) | 2.78 (1.76 to 4.54) | -1.02 (-1.06 to -0.99) |
| Uganda | 88 (31 to 179) | 0.73 (0.33 to 1.21) |  | 158 (82 to 260) | 0.62 (0.34 to 0.97) | -0.77 (-1.06 to -0.49) |
| Ukraine | 289 (213 to 379) | 0.45 (0.34 to 0.59) |  | 98 (75 to 157) | 0.16 (0.12 to 0.26) | -3.55 (-4.07 to -3.02) |
| United Arab Emirates | 5 (2 to 11) | 0.37 (0.17 to 0.8) |  | 53 (27 to 97) | 0.5 (0.3 to 0.91) | 1.05 (0.83 to 1.27) |
| United Kingdom | 305 (255 to 446) | 0.41 (0.33 to 0.54) |  | 360 (297 to 535) | 0.31 (0.26 to 0.45) | -0.86 (-1.77 to 0.06) |
| United Republic of Tanzania | 89 (34 to 164) | 0.48 (0.24 to 0.72) |  | 196 (102 to 347) | 0.47 (0.27 to 0.78) | 0 (-0.13 to 0.13) |
| United States of America | 977 (854 to 1532) | 0.33 (0.29 to 0.51) |  | 2453 (1536 to 2725) | 0.49 (0.31 to 0.54) | 1.47 (1.2 to 1.75) |
| United States Virgin Islands | 1 (1 to 1) | 0.8 (0.56 to 1.05) |  | 1 (1 to 1) | 0.68 (0.51 to 0.94) | -0.52 (-0.72 to -0.32) |
| Uruguay | 27 (15 to 32) | 0.79 (0.44 to 0.94) |  | 14 (11 to 18) | 0.34 (0.28 to 0.43) | -3.35 (-3.8 to -2.9) |
| Uzbekistan | 179 (130 to 241) | 1.34 (0.96 to 1.84) |  | 179 (131 to 303) | 0.7 (0.53 to 1.16) | -2.08 (-2.75 to -1.4) |
| Vanuatu | 3 (1 to 6) | 3.76 (1.68 to 6.93) |  | 8 (4 to 13) | 3.84 (2.06 to 6.24) | 0.09 (-0.15 to 0.34) |
| Venezuela (Bolivarian Republic of) | 34 (22 to 43) | 0.22 (0.15 to 0.27) |  | 44 (31 to 64) | 0.16 (0.11 to 0.23) | -0.96 (-1.32 to -0.6) |
| Viet Nam | 164 (102 to 289) | 0.36 (0.21 to 0.66) |  | 306 (147 to 521) | 0.32 (0.16 to 0.54) | -0.47 (-0.65 to -0.3) |
| Yemen | 18 (6 to 58) | 0.22 (0.1 to 0.55) |  | 52 (31 to 91) | 0.29 (0.18 to 0.46) | 0.94 (0.86 to 1.02) |
| Zambia | 36 (11 to 78) | 0.63 (0.28 to 1.03) |  | 66 (37 to 101) | 0.57 (0.33 to 0.86) | -0.33 (-0.48 to -0.18) |
| Zimbabwe | 39 (27 to 60) | 0.73 (0.53 to 1.07) |  | 64 (39 to 102) | 0.7 (0.45 to 1.07) | -0.17 (-0.32 to -0.03) |

CRDs: chronic respiratory diseases; AAPC: average annual percent change; UI: uncertainty interval; CI: confidence interval; SDI: Socio-demographic index.

Table S14. DALY of specific CRDs in 2019 for sexes, SDI and regions, with AAPC (1990-2019).

|  | CRDs | |  | COPD | |  | Asthma | |  | ILD&PS | |  | PNE | |  | Other CRDs | |
| --- | --- | --- | --- | --- | --- | --- | --- | --- | --- | --- | --- | --- | --- | --- | --- | --- | --- |
| GBD data | Age-standardised DALY per 100000 population (95% UI) | AAPC % (95% CI) 1990-2019 |  | Age-standardised DALY per 100000 population (95% UI) | AAPC % (95% CI) 1990-2019 |  | Age-standardised DALY per 100000 population (95% UI) | AAPC % (95% CI) 1990-2019 |  | Age-standardised DALY per 100000 population (95% UI) | AAPC % (95% CI) 1990-2019 |  | Age-standardised DALY per 100000 population (95% UI) | AAPC % (95% CI) 1990-2019 |  | Age-standardised DALY per 100000 population (95% UI) | AAPC % (95% CI) 1990-2019 |
| Global | 1293.74 (1182.99 to 1403.57) | -1.72 (-1.78 to -1.65) |  | 926.08 (848.76 to 997.67) | -1.82 (-1.9 to -1.74) |  | 273.63 (216.71 to 343.38) | -1.94 (-2.07 to -1.81) |  | 46.45 (35.12 to 54.98) | 0.44 (0.3 to 0.58) |  | 11.1 (9.23 to 13.45) | -2.03 (-2.19 to -1.87) |  | 36.48 (31.41 to 41.1) | 0.45 (0.4 to 0.51) |
| **Sex** |  |  |  |  |  |  |  |  |  |  |  |  |  |  |  |  |  |
| Female | 1093.03 (965.72 to 1208.96) | -1.63 (-1.7 to -1.56) |  | 744.12 (652.18 to 822.2) | -1.75 (-1.83 to -1.67) |  | 275.24 (214.89 to 349.63) | -1.8 (-1.88 to -1.73) |  | 38.99 (28.09 to 46.8) | 0.62 (0.54 to 0.7) |  | 1.71 (1.43 to 2.09) | -1.22 (-1.35 to -1.09) |  | 32.97 (27.69 to 38.3) | 0.68 (0.63 to 0.73) |
| Male | 1538.69 (1399.47 to 1690.3) | -1.82 (-1.9 to -1.74) |  | 1149.12 (1050.15 to 1257.26) | -1.88 (-1.98 to -1.79) |  | 272.16 (216.88 to 340.92) | -2.13 (-2.27 to -1.99) |  | 55.48 (36.9 to 70.33) | 0.24 (0.12 to 0.35) |  | 21.68 (18.03 to 26.13) | -2.16 (-2.3 to -2.01) |  | 40.24 (33.52 to 46.68) | 0.26 (0.19 to 0.32) |
| **SDI** |  |  |  |  |  |  |  |  |  |  |  |  |  |  |  |  |  |
| High SDI | 924.07 (797.2 to 1067.03) | -0.63 (-0.68 to -0.57) |  | 543.53 (499.18 to 582.88) | -0.49 (-0.54 to -0.44) |  | 293.69 (200.72 to 419.44) | -1.18 (-1.45 to -0.92) |  | 58.79 (39.65 to 71.48) | 0.89 (0.79 to 0.99) |  | 5.67 (5 to 6.44) | -2.85 (-3.03 to -2.67) |  | 22.39 (19.42 to 25) | 0.84 (0.76 to 0.92) |
| High-middle SDI | 837 (750.92 to 948.78) | -2.67 (-2.88 to -2.46) |  | 617.69 (562.78 to 708.9) | -2.99 (-3.2 to -2.79) |  | 158.33 (114 to 220.25) | -2 (-2.22 to -1.79) |  | 26 (20.53 to 30.04) | 0.04 (-0.03 to 0.12) |  | 11.88 (9.26 to 15.09) | -2.23 (-2.45 to -2.01) |  | 23.1 (19.96 to 26.23) | -0.61 (-0.68 to -0.53) |
| Middle SDI | 1316.93 (1199.96 to 1449.4) | -2.53 (-2.65 to -2.42) |  | 1007.2 (915.7 to 1112.45) | -2.75 (-2.91 to -2.59) |  | 230.64 (183.79 to 290.27) | -2.06 (-2.14 to -1.98) |  | 31.2 (26.09 to 37.34) | 0.36 (0.26 to 0.46) |  | 15.52 (12.39 to 19.44) | -2.16 (-2.35 to -1.97) |  | 32.37 (28.37 to 36.43) | 0.49 (0.43 to 0.56) |
| Low-middle SDI | 2314.24 (2029.67 to 2562.08) | -1.44 (-1.68 to -1.2) |  | 1728.64 (1488.81 to 1923.71) | -1.3 (-1.41 to -1.19) |  | 445.9 (348.53 to 546.29) | -2.26 (-2.57 to -1.95) |  | 78.56 (54.31 to 107.04) | 0.15 (-0.12 to 0.43) |  | 9.24 (7.17 to 11.26) | -1.92 (-2.04 to -1.8) |  | 51.9 (43.21 to 59.92) | 0.32 (0.22 to 0.43) |
| Low SDI | 2048.37 (1802.01 to 2247.14) | -1.05 (-1.21 to -0.89) |  | 1364.11 (1201.64 to 1522.56) | -0.71 (-0.9 to -0.53) |  | 553.9 (434.74 to 726.78) | -1.9 (-1.98 to -1.82) |  | 66.6 (44.1 to 89.25) | 0.27 (0.08 to 0.46) |  | 6.17 (3.09 to 8.6) | -1.57 (-1.67 to -1.46) |  | 57.58 (45.79 to 69.12) | 0.4 (0.36 to 0.44) |
| **Region** |  |  |  |  |  |  |  |  |  |  |  |  |  |  |  |  |  |
| High-income Asia Pacific | 468.37 (392.53 to 561.9) | -2.07 (-2.19 to -1.95) |  | 224.49 (193.59 to 258.41) | -1.48 (-1.61 to -1.35) |  | 160.29 (105.89 to 237.58) | -3.35 (-3.59 to -3.11) |  | 62.5 (41.53 to 79.52) | 0.51 (0.42 to 0.59) |  | 6.09 (5.07 to 7.35) | -3.81 (-4.1 to -3.52) |  | 14.99 (12.56 to 17.57) | 0.12 (0.03 to 0.2) |
| High-income North America | 1374 (1180.95 to 1570.24) | 0.16 (0.03 to 0.3) |  | 848.43 (765.9 to 903.04) | 0.14 (0.1 to 0.18) |  | 412.95 (282.52 to 584.39) | 0.25 (-0.22 to 0.73) |  | 78.28 (53.48 to 92.3) | 0.74 (0.66 to 0.82) |  | 4.09 (3.62 to 4.56) | -2.13 (-2.29 to -1.97) |  | 30.24 (24.99 to 33.3) | 1.21 (1 to 1.43) |
| Western Europe | 769.63 (660.74 to 900.99) | -1.06 (-1.16 to -0.97) |  | 460.78 (418.27 to 499.58) | -0.73 (-0.8 to -0.67) |  | 245.62 (162.85 to 361.91) | -1.79 (-2.14 to -1.45) |  | 41.51 (26.17 to 51.19) | 1.15 (0.99 to 1.31) |  | 4.39 (3.97 to 4.82) | -4.25 (-4.47 to -4.03) |  | 17.34 (14.61 to 20.55) | -0.88 (-0.98 to -0.77) |
| Australasia | 946.08 (793.37 to 1128.11) | -1.31 (-1.38 to -1.24) |  | 512.07 (455.06 to 564.8) | -1.26 (-1.38 to -1.14) |  | 359.99 (239.04 to 527.64) | -1.77 (-1.97 to -1.56) |  | 46.67 (25.34 to 62.94) | 2.05 (1.79 to 2.31) |  | 5.11 (4.33 to 6) | 0.99 (0.71 to 1.27) |  | 22.24 (19.14 to 26.16) | 0.28 (0.19 to 0.37) |
| Andean Latin America | 680.12 (559.51 to 811.82) | -1.45 (-1.6 to -1.29) |  | 281.86 (236.05 to 335.07) | -1.26 (-1.45 to -1.08) |  | 192.28 (124.91 to 291.95) | -2.59 (-2.78 to -2.39) |  | 168.85 (122.93 to 212.42) | 0.69 (0.46 to 0.92) |  | 5.34 (4.09 to 6.98) | -3.12 (-4.38 to -1.84) |  | 31.78 (25.06 to 40.78) | -2.97 (-3.17 to -2.78) |
| Tropical Latin America | 909.91 (808.6 to 1037.21) | -1.47 (-1.6 to -1.33) |  | 613.23 (563.89 to 674.01) | -1.55 (-1.68 to -1.42) |  | 227.28 (154.5 to 339.89) | -1.42 (-1.52 to -1.32) |  | 31.31 (20.31 to 37.85) | 0.66 (0.54 to 0.78) |  | 8.08 (7.01 to 9.44) | -0.54 (-0.7 to -0.38) |  | 30.02 (25.71 to 34.61) | 0.09 (0.01 to 0.17) |
| Central Latin America | 784.43 (686.77 to 890.61) | -0.97 (-1.1 to -0.83) |  | 529.96 (461.49 to 605.57) | -0.52 (-0.7 to -0.34) |  | 166.15 (116.28 to 237.45) | -2.52 (-2.7 to -2.33) |  | 50.61 (32.92 to 64.75) | 1.2 (1.12 to 1.29) |  | 4.16 (3.43 to 5.01) | -2.75 (-2.97 to -2.52) |  | 33.54 (27.48 to 39.18) | 0.16 (-0.09 to 0.4) |
| Southern Latin America | 942.1 (817.88 to 1086.82) | -0.2 (-0.3 to -0.1) |  | 544.81 (493.05 to 601.24) | -0.09 (-0.2 to 0.01) |  | 275.77 (188.18 to 399.41) | -0.67 (-0.76 to -0.58) |  | 84.66 (62.31 to 97.99) | 1.12 (1.03 to 1.21) |  | 4.5 (3.91 to 5.17) | -1.49 (-1.85 to -1.13) |  | 32.37 (28.07 to 37.75) | -0.91 (-1.29 to -0.53) |
| Caribbean | 960.2 (797.86 to 1118.46) | -0.35 (-0.42 to -0.28) |  | 463.2 (400.14 to 527.69) | 0.5 (0.41 to 0.58) |  | 422.79 (315.68 to 553.54) | -1.22 (-1.3 to -1.15) |  | 28.96 (20.66 to 38.26) | 1.19 (1.09 to 1.28) |  | 1.11 (0.74 to 1.63) | -1.3 (-1.56 to -1.03) |  | 44.15 (29.1 to 70.89) | 0.42 (0.32 to 0.52) |
| Central Europe | 678.01 (583.53 to 787.39) | -1.57 (-1.64 to -1.5) |  | 452.77 (402.69 to 504.13) | -1.41 (-1.52 to -1.29) |  | 180.45 (119.9 to 263.92) | -2.06 (-2.23 to -1.89) |  | 23.87 (19.78 to 28.54) | -0.97 (-1.07 to -0.87) |  | 3.93 (3.24 to 4.72) | -4.3 (-4.49 to -4.1) |  | 16.99 (14.03 to 19.97) | 0.03 (-0.04 to 0.11) |
| Eastern Europe | 537.15 (467.11 to 633.55) | -2.4 (-2.85 to -1.94) |  | 381.51 (336.85 to 448.43) | -2.34 (-3.06 to -1.62) |  | 124.09 (84.15 to 182.59) | -2.85 (-3.08 to -2.61) |  | 12.43 (10.03 to 16.67) | -1.89 (-2.11 to -1.66) |  | 2.88 (2.37 to 3.48) | -2.75 (-3.16 to -2.35) |  | 16.24 (13.48 to 19.59) | -0.74 (-1.05 to -0.44) |
| Central Asia | 937.22 (847.05 to 1056.72) | -0.99 (-1.24 to -0.73) |  | 662.79 (602.45 to 747.77) | -0.82 (-1.13 to -0.52) |  | 197.95 (160.01 to 246.02) | -1.61 (-1.79 to -1.43) |  | 35.79 (29.4 to 43.19) | -0.7 (-0.83 to -0.58) |  | 3.79 (3.06 to 4.63) | -0.23 (-1.27 to 0.83) |  | 36.9 (31.34 to 46.14) | -0.5 (-0.62 to -0.37) |
| North Africa and Middle East | 1033.42 (906.68 to 1149.27) | -1.03 (-1.13 to -0.94) |  | 649.08 (574.58 to 717.67) | -0.43 (-0.5 to -0.37) |  | 324.16 (259.23 to 397.53) | -2.21 (-2.27 to -2.16) |  | 30.01 (22.72 to 45.25) | 0.36 (0.3 to 0.42) |  | 1.99 (1.68 to 2.34) | -0.77 (-0.93 to -0.61) |  | 28.19 (23.91 to 32.49) | 1.26 (1.19 to 1.33) |
| South Asia | 2559.27 (2206.93 to 2879.03) | -1.4 (-1.55 to -1.24) |  | 1915.92 (1617.03 to 2171.73) | -1.2 (-1.37 to -1.03) |  | 472 (350.52 to 601.23) | -2.35 (-2.74 to -1.96) |  | 109.24 (74.08 to 148.96) | -0.04 (-0.33 to 0.25) |  | 6.33 (3.99 to 8.66) | -2.31 (-2.47 to -2.14) |  | 55.78 (45.84 to 65.65) | 0.18 (0.12 to 0.25) |
| Southeast Asia | 1383.12 (1235.32 to 1511.65) | -1.57 (-1.64 to -1.51) |  | 895.56 (785.02 to 981.38) | -1.19 (-1.26 to -1.11) |  | 433.23 (365.45 to 509.73) | -2.33 (-2.39 to -2.26) |  | 16.96 (10.47 to 31.01) | -0.07 (-0.16 to 0.01) |  | 1.29 (0.98 to 1.69) | -0.37 (-0.47 to -0.27) |  | 36.08 (30.19 to 45.45) | 0.5 (0.43 to 0.57) |
| East Asia | 1270.89 (1120.48 to 1470.61) | -3.87 (-4.1 to -3.63) |  | 1100.02 (964.12 to 1299.38) | -4.08 (-4.35 to -3.81) |  | 106.42 (75.33 to 152.05) | -2.23 (-2.63 to -1.84) |  | 12.49 (9.96 to 15.35) | -0.14 (-0.27 to -0.01) |  | 29.23 (22.87 to 37.14) | -2.1 (-2.22 to -1.97) |  | 22.72 (18.72 to 26.68) | 0.12 (0.05 to 0.2) |
| Oceania | 3677.62 (3020.87 to 4477.19) | -0.72 (-0.77 to -0.67) |  | 2309.94 (1847.48 to 2874.57) | -0.47 (-0.55 to -0.39) |  | 1102.21 (863.7 to 1431.32) | -1.34 (-1.4 to -1.27) |  | 97.9 (69.85 to 142.17) | 0.24 (-0.01 to 0.5) |  | 5.77 (3.67 to 7.85) | -0.64 (-0.73 to -0.55) |  | 161.79 (90.64 to 280.06) | 0.55 (0.47 to 0.62) |
| Western Sub-Saharan Africa | 1128.76 (986.27 to 1272.71) | -1.02 (-1.1 to -0.95) |  | 619.02 (542.83 to 692.7) | -0.6 (-0.68 to -0.51) |  | 425.22 (354.05 to 508.79) | -1.75 (-1.83 to -1.66) |  | 16.49 (11.37 to 21.83) | -1.48 (-1.61 to -1.35) |  | 1.41 (1.05 to 1.75) | 0.34 (0.11 to 0.56) |  | 66.62 (51.32 to 81.69) | 0.59 (0.45 to 0.73) |
| Eastern Sub-Saharan Africa | 1231.52 (1082.84 to 1392.41) | -1.37 (-1.41 to -1.32) |  | 707.54 (622.49 to 801.74) | -1.04 (-1.11 to -0.97) |  | 450.86 (356.42 to 582.77) | -1.94 (-1.99 to -1.9) |  | 23.57 (11.37 to 36.17) | -0.33 (-0.43 to -0.23) |  | 6.73 (2.96 to 9.91) | -1.73 (-1.82 to -1.64) |  | 42.82 (31.94 to 53.53) | 0.28 (0.19 to 0.36) |
| Central Sub-Saharan Africa | 1625 (1229.32 to 2246.8) | -1.01 (-1.11 to -0.91) |  | 964.51 (743.67 to 1321.27) | -0.62 (-0.68 to -0.56) |  | 572.95 (389.25 to 907.95) | -1.65 (-1.71 to -1.58) |  | 33.28 (14.24 to 61.26) | -0.37 (-0.44 to -0.3) |  | 8.54 (4.77 to 13.47) | -1.17 (-1.31 to -1.03) |  | 45.72 (33.74 to 58.79) | 0.3 (0.21 to 0.38) |
| Southern Sub-Saharan Africa | 1387.64 (1263.76 to 1520.81) | -0.94 (-1.13 to -0.74) |  | 838.57 (772.56 to 907.81) | -0.55 (-0.89 to -0.21) |  | 446.49 (378.51 to 530.69) | -1.54 (-1.94 to -1.15) |  | 43.53 (36.12 to 56.35) | -0.73 (-0.91 to -0.55) |  | 11.57 (10.22 to 13.17) | -0.88 (-1.34 to -0.41) |  | 47.48 (40.96 to 57.21) | 0 (-0.13 to 0.13) |

DALY: Disability-Adjusted Life Year; CRDs: chronic respiratory diseases; COPD: chronic obstructive pulmonary disease; ILD&PS: Interstitial lung disease and pulmonary sarcoidosis; PNE: pneumoconiosis; AAPC: average annual percent change; UI: uncertainty interval; CI: confidence interval; SDI: Socio-demographic index.

Table S15. DALY of CRDs (1990/2019) for sexes, SDI and locations, with AAPC (1990-2019)

| GBD data | 1990 | |  | 2019 | | AAPC % (95% CI)  1990-2019 |
| --- | --- | --- | --- | --- | --- | --- |
| Cases (95% UI) | Age-standardised DALY per  100 000 population (95% UI) |  | Cases (95% UI) | Age-standardised DALY per  100 000 population (95% UI) |
| **Global** | 85701654 (74882447 to 92675585) | 2107.59 (1836.22 to 2266.19) |  | 103533107 (94792077 to 112266452) | 1293.74 (1182.99 to 1403.57) | -1.72 (-1.78 to -1.65) |
| **Sex** |  |  |  |  |  |  |
| Female | 38051582 (30884229 to 42090584) | 1726.15 (1395.38 to 1896.69) |  | 46526316 (41119608 to 51370913) | 1093.03 (965.72 to 1208.96) | -1.63 (-1.7 to -1.56) |
| Male | 47650072 (41972025 to 51907609) | 2601.44 (2288.63 to 2817.61) |  | 57006791 (51895441 to 62641862) | 1538.69 (1399.47 to 1690.3) | -1.82 (-1.9 to -1.74) |
| **SDI** |  |  |  |  |  |  |
| High SDI | 10863283 (9549816 to 12375949) | 1109.21 (961.09 to 1283.53) |  | 14764858 (13146962 to 16366860) | 924.07 (797.2 to 1067.03) | -0.63 (-0.68 to -0.57) |
| High-middle SDI | 18281363 (15646583 to 19951921) | 1767.21 (1507.45 to 1926.22) |  | 15743868 (14247302 to 17869088) | 837 (750.92 to 948.78) | -2.67 (-2.88 to -2.46) |
| Middle SDI | 27366629 (22629047 to 29928439) | 2712.23 (2230.86 to 2951.54) |  | 30297497 (27620574 to 33345421) | 1316.93 (1199.96 to 1449.4) | -2.53 (-2.65 to -2.42) |
| Low-middle SDI | 21470643 (18532330 to 23437402) | 3504.76 (2997.35 to 3836.69) |  | 30792248 (27029274 to 34056139) | 2314.24 (2029.67 to 2562.08) | -1.44 (-1.68 to -1.2) |
| Low SDI | 7683083 (6649910 to 8553763) | 2749.84 (2370.45 to 3068.74) |  | 11882206 (10516270 to 13218432) | 2048.37 (1802.01 to 2247.14) | -1.05 (-1.21 to -0.89) |
| **Region** |  |  |  |  |  |  |
| High-income Asia Pacific | 1560914 (1332053 to 1825283) | 837.64 (713.06 to 984.42) |  | 1746166 (1535587 to 1955982) | 468.37 (392.53 to 561.9) | -2.07 (-2.19 to -1.95) |
| High-income North America | 4312309 (3807518 to 4869085) | 1309.32 (1138.88 to 1514.62) |  | 7451116 (6536326 to 8268824) | 1374 (1180.95 to 1570.24) | 0.16 (0.03 to 0.3) |
| Western Europe | 5448330 (4829714 to 6171813) | 1034.21 (899.3 to 1205.75) |  | 5951210 (5260861 to 6622182) | 769.63 (660.74 to 900.99) | -1.06 (-1.16 to -0.97) |
| Australasia | 305649 (264599 to 351338) | 1376.88 (1179.57 to 1606.72) |  | 391851 (340881 to 445018) | 946.08 (793.37 to 1128.11) | -1.31 (-1.38 to -1.24) |
| Andean Latin America | 348007 (286044 to 410810) | 1035.12 (903.56 to 1176.3) |  | 393916 (323481 to 472210) | 680.12 (559.51 to 811.82) | -1.45 (-1.6 to -1.29) |
| Tropical Latin America | 1456836 (1271255 to 1697408) | 1366.72 (1238.72 to 1516.07) |  | 2082958 (1870585 to 2347497) | 909.91 (808.6 to 1037.21) | -1.47 (-1.6 to -1.33) |
| Central Latin America | 1131583 (987165 to 1310849) | 1030.45 (936.74 to 1132.59) |  | 1831475 (1599897 to 2086032) | 784.43 (686.77 to 890.61) | -0.97 (-1.1 to -0.83) |
| Southern Latin America | 470413 (414337 to 537764) | 1006.41 (889.66 to 1149.56) |  | 739935 (648037 to 840683) | 942.1 (817.88 to 1086.82) | -0.2 (-0.3 to -0.1) |
| Caribbean | 347478 (286973 to 414111) | 1082.25 (917.66 to 1261.62) |  | 465876 (389501 to 539116) | 960.2 (797.86 to 1118.46) | -0.35 (-0.42 to -0.28) |
| Central Europe | 1511302 (1366472 to 1674472) | 1072.36 (963.33 to 1197.13) |  | 1240537 (1091708 to 1395421) | 678.01 (583.53 to 787.39) | -1.57 (-1.64 to -1.5) |
| Eastern Europe | 2974077 (2537563 to 3275914) | 1111.67 (943.64 to 1234.04) |  | 1612662 (1425088 to 1898858) | 537.15 (467.11 to 633.55) | -2.4 (-2.85 to -1.94) |
| Central Asia | 639076 (569393 to 692146) | 1290.81 (1147.5 to 1380.62) |  | 675503 (604277 to 764579) | 937.22 (847.05 to 1056.72) | -0.99 (-1.24 to -0.73) |
| North Africa and Middle East | 2964169 (2615504 to 3338882) | 1403.74 (1252.64 to 1560.45) |  | 4759606 (4142498 to 5361709) | 1033.42 (906.68 to 1149.27) | -1.03 (-1.13 to -0.94) |
| South Asia | 21027290 (18425676 to 23151814) | 3830.22 (3350.24 to 4261.91) |  | 34548257 (29879421 to 38832883) | 2559.27 (2206.93 to 2879.03) | -1.4 (-1.55 to -1.24) |
| Southeast Asia | 6257860 (5435239 to 6867517) | 2163.45 (1822.55 to 2373.96) |  | 8177396 (7253985 to 8958447) | 1383.12 (1235.32 to 1511.65) | -1.57 (-1.64 to -1.51) |
| East Asia | 29644067 (22556379 to 33046668) | 3845.41 (2938.76 to 4279.44) |  | 23441600 (20675834 to 27335346) | 1270.89 (1120.48 to 1470.61) | -3.87 (-4.1 to -3.63) |
| Oceania | 153816 (131582 to 180254) | 4495.51 (3837.12 to 5182.71) |  | 283407 (230257 to 352567) | 3677.62 (3020.87 to 4477.19) | -0.72 (-0.77 to -0.67) |
| Western Sub-Saharan Africa | 1747750 (1506295 to 1992185) | 1524.38 (1310.15 to 1739.37) |  | 2929955 (2498580 to 3377354) | 1128.76 (986.27 to 1272.71) | -1.02 (-1.1 to -0.95) |
| Eastern Sub-Saharan Africa | 2120442 (1807690 to 2414021) | 1822.22 (1588.45 to 2047.46) |  | 2913833 (2462881 to 3464028) | 1231.52 (1082.84 to 1392.41) | -1.37 (-1.41 to -1.32) |
| Central Sub-Saharan Africa | 660311 (507263 to 803590) | 2152.87 (1630.09 to 2792.55) |  | 1063506 (826873 to 1368014) | 1625 (1229.32 to 2246.8) | -1.01 (-1.11 to -0.91) |
| Southern Sub-Saharan Africa | 619977 (548657 to 691134) | 1861.61 (1647.57 to 2094.22) |  | 832342 (752858 to 922070) | 1387.64 (1263.76 to 1520.81) | -0.94 (-1.13 to -0.74) |
| **Countries** |  |  |  |  |  |  |
| Afghanistan | 191923 (160093 to 226504) | 2417.04 (2022.96 to 2856.97) |  | 306361 (250838 to 369967) | 1754.03 (1422.52 to 2077.07) | -1.05 (-1.15 to -0.94) |
| Albania | 24582 (19699 to 27109) | 1179.92 (906.55 to 1288.31) |  | 19951 (16193 to 24255) | 527.32 (430.71 to 642.15) | -2.73 (-2.96 to -2.49) |
| Algeria | 165365 (135560 to 199553) | 1175.93 (960.21 to 1417.9) |  | 267335 (226065 to 317522) | 788.59 (668.69 to 929.8) | -1.35 (-1.39 to -1.31) |
| American Samoa | 556 (491 to 628) | 2119.32 (1866.76 to 2360.45) |  | 646 (552 to 771) | 1370.04 (1177.28 to 1643.94) | -1.5 (-1.61 to -1.39) |
| Andorra | 546 (444 to 669) | 1031.39 (851.66 to 1251.41) |  | 1034 (861 to 1218) | 830.99 (679.8 to 993.13) | -0.74 (-0.81 to -0.67) |
| Angola | 128386 (97462 to 166379) | 2194.06 (1705.3 to 2779.55) |  | 189114 (153309 to 226499) | 1248.5 (1016.23 to 1527.07) | -1.98 (-2.12 to -1.84) |
| Antigua and Barbuda | 282 (227 to 357) | 477.73 (391.06 to 595.16) |  | 435 (354 to 534) | 501.45 (398.66 to 628.48) | 0.1 (-0.05 to 0.25) |
| Argentina | 341074 (298946 to 392042) | 1057 (927.6 to 1211.3) |  | 529348 (461244 to 602800) | 1031.28 (893.04 to 1194.09) | -0.09 (-0.2 to 0.01) |
| Armenia | 28691 (25357 to 31103) | 1089.63 (949.69 to 1174.05) |  | 28248 (24106 to 32467) | 727.73 (619.41 to 836.92) | -1.5 (-1.91 to -1.1) |
| Australia | 248171 (215375 to 283528) | 1345.83 (1152.48 to 1561.33) |  | 328077 (283933 to 374683) | 945.42 (786.39 to 1138.96) | -1.23 (-1.29 to -1.16) |
| Austria | 80706 (69820 to 93342) | 763.56 (646.99 to 909.3) |  | 96019 (82912 to 109715) | 651.41 (543.85 to 777.17) | -0.58 (-0.7 to -0.45) |
| Azerbaijan | 58855 (53533 to 64425) | 1092.61 (996.32 to 1190.98) |  | 65300 (54935 to 85407) | 758.76 (636.6 to 1026.08) | -1.15 (-1.34 to -0.97) |
| Bahamas | 1329 (1092 to 1658) | 634.76 (539.75 to 749.99) |  | 2211 (1822 to 2689) | 602.2 (491.01 to 746.09) | -0.18 (-0.28 to -0.08) |
| Bahrain | 3496 (3048 to 4029) | 1719.55 (1532.53 to 1932.05) |  | 7571 (6382 to 9052) | 875.79 (751.56 to 1030.56) | -2.39 (-3.1 to -1.68) |
| Bangladesh | 1730648 (1505307 to 2053533) | 3434.42 (2989 to 4181.58) |  | 1942679 (1593316 to 2790908) | 1499.65 (1228.5 to 2192.86) | -2.78 (-3.44 to -2.11) |
| Barbados | 1536 (1262 to 1850) | 570.34 (461.06 to 699.11) |  | 2059 (1703 to 2482) | 576.69 (457.07 to 731.58) | 0 (-0.17 to 0.17) |
| Belarus | 166780 (137666 to 184151) | 1336.23 (1099.21 to 1481.99) |  | 70914 (57600 to 94487) | 517.73 (413.95 to 669.25) | -3.38 (-3.86 to -2.9) |
| Belgium | 172781 (156122 to 191834) | 1208.83 (1073.49 to 1371.05) |  | 178071 (157984 to 198309) | 878.54 (763.04 to 1011.86) | -1.19 (-1.35 to -1.02) |
| Belize | 1247 (1022 to 1530) | 804.34 (689.78 to 931.21) |  | 3128 (2620 to 3733) | 935.94 (793.63 to 1092.21) | 0.7 (0.42 to 0.99) |
| Benin | 49497 (41198 to 57766) | 1841.12 (1542.65 to 2124.84) |  | 86780 (70383 to 107612) | 1244.48 (1030.23 to 1517.17) | -1.36 (-1.45 to -1.27) |
| Bermuda | 381 (326 to 445) | 650.54 (550.43 to 772.73) |  | 473 (399 to 561) | 544.71 (435.41 to 681.55) | -0.58 (-0.63 to -0.52) |
| Bhutan | 8996 (6735 to 11533) | 3547.71 (2710.15 to 4520.09) |  | 13059 (10397 to 17104) | 2436.44 (1953.52 to 3190) | -1.32 (-1.36 to -1.27) |
| Bolivia (Plurinational State of) | 86366 (63562 to 108920) | 1619.83 (1317.49 to 1905.41) |  | 98267 (79671 to 118546) | 1063.53 (870.16 to 1274.71) | -1.44 (-1.47 to -1.41) |
| Bosnia and Herzegovina | 37307 (32454 to 41834) | 956.12 (834.57 to 1062.25) |  | 33919 (28376 to 40118) | 662.32 (551.63 to 786.31) | -1.36 (-1.84 to -0.87) |
| Botswana | 15417 (11746 to 20252) | 2340.52 (1789.23 to 3114.17) |  | 24044 (19002 to 29777) | 1589.19 (1266.81 to 1956.25) | -1.35 (-1.46 to -1.23) |
| Brazil | 1436485 (1254143 to 1671651) | 1387.29 (1257.26 to 1538.71) |  | 2042184 (1835929 to 2300990) | 915.42 (813.67 to 1043.39) | -1.5 (-1.64 to -1.36) |
| Brunei Darussalam | 2280 (1887 to 2587) | 2401.96 (1859.45 to 2691.4) |  | 3171 (2761 to 3657) | 1292.76 (1146.28 to 1451.17) | -2.13 (-2.34 to -1.91) |
| Bulgaria | 111059 (100061 to 121998) | 975.71 (876.51 to 1082.37) |  | 83661 (70107 to 99653) | 692.69 (575.69 to 818.51) | -1.12 (-1.24 to -1) |
| Burkina Faso | 72472 (59101 to 86609) | 1183.41 (997.84 to 1370.14) |  | 139722 (114073 to 168460) | 992.47 (844.37 to 1155.76) | -0.53 (-0.68 to -0.38) |
| Burundi | 96798 (71433 to 120785) | 2696 (2050.63 to 3261.29) |  | 109117 (85848 to 144244) | 1675.53 (1324.13 to 2118.8) | -1.71 (-1.88 to -1.55) |
| Cabo Verde | 3924 (2850 to 4395) | 1552.88 (1102.67 to 1724.71) |  | 3659 (3162 to 4414) | 779.55 (675.18 to 939.7) | -2.58 (-2.87 to -2.29) |
| Cambodia | 96700 (81427 to 111358) | 1732.93 (1475.95 to 1972.2) |  | 150283 (125987 to 172225) | 1264.58 (1062.21 to 1439.33) | -1.08 (-1.12 to -1.04) |
| Cameroon | 100022 (85165 to 116074) | 1804.48 (1519.81 to 2091.54) |  | 195154 (155381 to 240340) | 1208.68 (975.49 to 1491.33) | -1.37 (-1.46 to -1.27) |
| Canada | 269100 (239674 to 302712) | 867.97 (763.01 to 993.29) |  | 446332 (388801 to 499640) | 744.36 (640.9 to 860.93) | -0.52 (-0.6 to -0.44) |
| Central African Republic | 42125 (32682 to 52085) | 2805.54 (2101.05 to 3690.39) |  | 63887 (47596 to 84358) | 2323.42 (1683.25 to 3319.25) | -0.64 (-0.7 to -0.57) |
| Chad | 64860 (52434 to 80819) | 1813.07 (1456.23 to 2315.09) |  | 118887 (97016 to 144239) | 1433.32 (1165.05 to 1745.24) | -0.78 (-0.86 to -0.7) |
| Chile | 90726 (78355 to 104594) | 867.05 (756.8 to 982.71) |  | 160293 (136186 to 184274) | 721.04 (609.75 to 845.92) | -0.68 (-0.85 to -0.5) |
| China | 28992086 (22016545 to 32387823) | 3910.13 (2991.37 to 4343.33) |  | 22520549 (19719779 to 26416880) | 1269.95 (1114 to 1473.89) | -3.93 (-4.17 to -3.68) |
| Colombia | 221546 (192564 to 259032) | 1051.5 (935.26 to 1167.54) |  | 388120 (318756 to 468217) | 755.57 (620.82 to 916.63) | -1.31 (-1.52 to -1.09) |
| Comoros | 5011 (3119 to 6449) | 1687.06 (1108.38 to 2125.52) |  | 6125 (5018 to 7320) | 1117.08 (929.59 to 1338.07) | -1.37 (-1.69 to -1.06) |
| Congo | 30065 (23633 to 37234) | 2296.31 (1751.8 to 2988.05) |  | 41550 (32629 to 50463) | 1366.85 (1078.72 to 1657.67) | -1.76 (-1.94 to -1.58) |
| Cook Islands | 251 (218 to 283) | 1858.19 (1627.8 to 2078.91) |  | 237 (206 to 272) | 1056.56 (910.17 to 1218.17) | -1.87 (-2.01 to -1.73) |
| Costa Rica | 19793 (16010 to 24528) | 868.95 (742.05 to 1017.37) |  | 35890 (29338 to 43173) | 736.98 (601.24 to 891.75) | -0.52 (-0.83 to -0.2) |
| Croatia | 43882 (38232 to 50018) | 739.08 (635.6 to 849.86) |  | 48168 (40609 to 56093) | 632.04 (523.86 to 747.77) | -0.52 (-0.56 to -0.47) |
| Cuba | 81812 (67823 to 98774) | 777.63 (645.23 to 940.95) |  | 130377 (105524 to 154175) | 852.63 (683.81 to 1038.33) | 0.4 (0.27 to 0.54) |
| Cyprus | 9000 (7592 to 10499) | 1235.1 (1042.66 to 1425.41) |  | 14574 (12169 to 17064) | 864.13 (706.84 to 1027.72) | -1.25 (-1.42 to -1.08) |
| Czechia | 93750 (85082 to 104446) | 731.97 (658.15 to 820.93) |  | 113490 (96194 to 130424) | 628.4 (528.72 to 728.31) | -0.31 (-0.52 to -0.1) |
| Côte d’Ivoire | 103762 (84357 to 122857) | 1782.18 (1463.58 to 2095.61) |  | 176611 (141403 to 213456) | 1180.16 (969 to 1416.73) | -1.46 (-1.6 to -1.32) |
| Democratic People's Republic of Korea | 492235 (373486 to 607475) | 3444.24 (2634.15 to 4285.68) |  | 720735 (597373 to 834077) | 2369.28 (1964.03 to 2733.79) | -1.3 (-1.37 to -1.23) |
| Democratic Republic of the Congo | 443580 (331043 to 558209) | 2102.43 (1518.79 to 2817.96) |  | 750175 (563758 to 1015026) | 1741.07 (1252.3 to 2575.29) | -0.68 (-0.77 to -0.58) |
[truncated: 249,494 more chars]
